# Diagnosis and Treatment of Leishmaniasis: Clinical Practice Guidelines by the Infectious Diseases Society of America (IDSA) and the American Society of Tropical Medicine and Hygiene (ASTMH)*

**DOI:** 10.4269/ajtmh.16-84256

**Published:** 2017-01-11

**Authors:** Naomi Aronson, Barbara L. Herwaldt, Michael Libman, Richard Pearson, Rogelio Lopez-Velez, Peter Weina, Edgar Carvalho, Moshe Ephros, Selma Jeronimo, Alan Magill

**Affiliations:** 1 Uniformed Services University of the Health Sciences, Bethesda, MD.; 2 Center for Disease Control and Prevention, Atlanta, GA.; 3 McGill University Health Centre, Montreal, Que, Canada.; 4 University of Virginia School of Medicine, Charlottesville, VA.; 5 University of Alcalá, Madrid, Spain.; 6 Walter Reed Army Institute of Research, Silver Spring, MD.; 7 Universidade Federal da Bahia, Salvador, BA, Brazil.; 8 Carmel Medical Center, Haifa, Israel.; 9 Federal University of Rio Grande do Norte, Natal, RN, Brazil.; 10 Bill and Melinda Gates Foundation, Seattle, WA.

## Executive Summary

**Figure 1:**
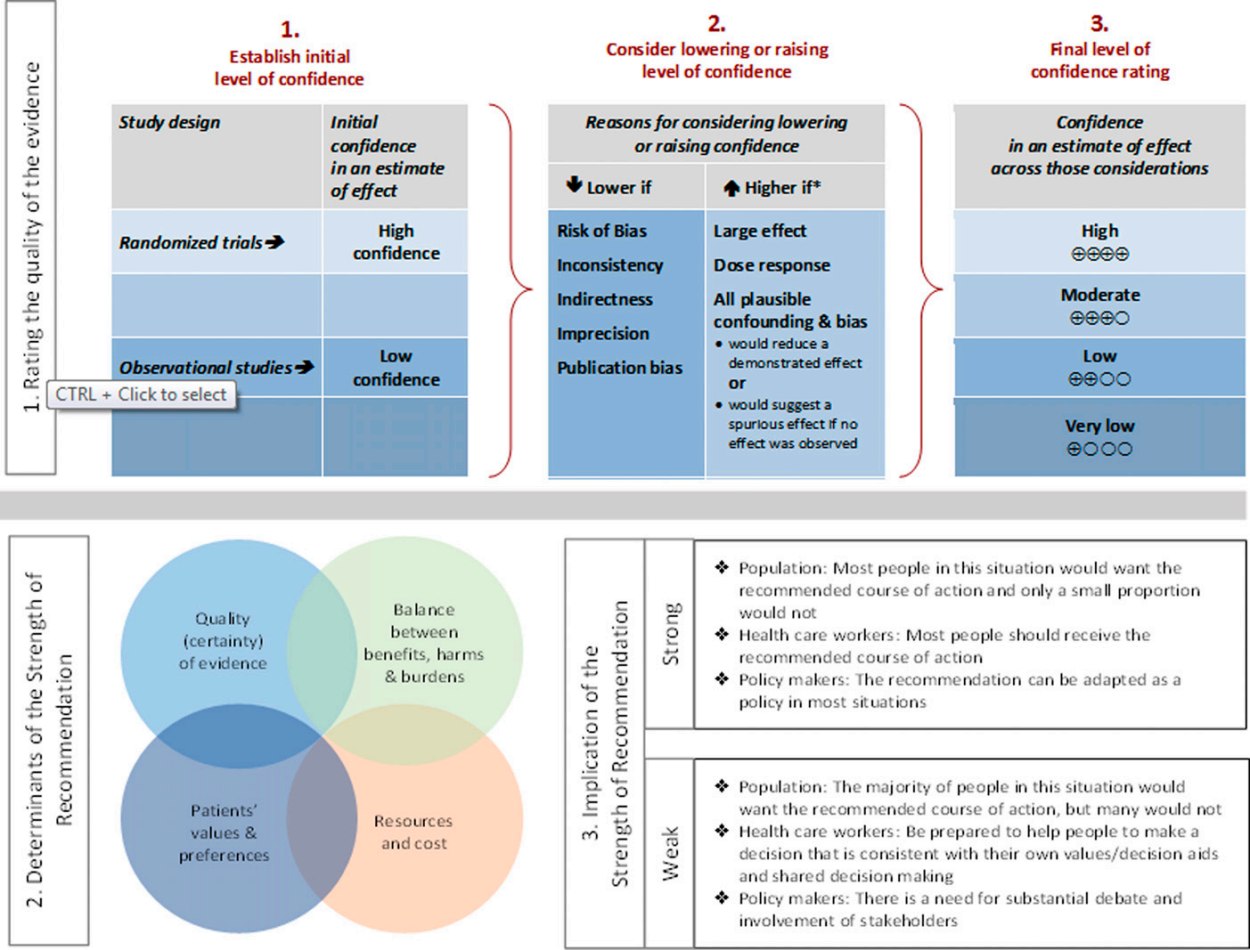
Approach and implications to rating the quality of evidence and strength of recommendations using the GRADE methodology (unrestricted use of the figure granted by the U.S. GRADE Network) [1]

Guidelines for the clinical management of persons with leishmaniasis were prepared by a Panel of the Infectious Diseases Society of America (IDSA) and the American Society of Tropical Medicine and Hygiene (ASTMH). The guidelines are intended for internists, pediatricians, family practitioners, and dermatologists, as well as infectious disease specialists, practicing in the United States and Canada (for simplicity, referred to here as North America). The Panel followed a guideline development process that has been adopted by IDSA, which includes a systematic method of grading both the quality of evidence (very low, low, moderate, or high) and the strength of the recommendation (weak or strong) [1] (Figure 1

).

In these guidelines, we describe our approaches to the diagnosis and management of cases of cutaneous, mucosal, and visceral leishmaniasis, the three main clinical syndromes caused by infection with *Leishmania* parasites. Less common or rare syndromes that may require specialized expertise are beyond the scope of these guidelines. Whenever possible, our recommendations are based on randomized clinical trials. However, because of the diversity encompassed by leishmaniasis, which includes a spectrum of diseases caused by >20 *Leishmania* species found in many areas of the world, many of the recommendations are based on observational studies, anecdotal data, or expert opinion. Although there may be disagreement with some of our recommendations and suggestions, the approaches we describe have been both useful and feasible in North America.

Cutaneous leishmaniasis (CL) is the most common leishmanial syndrome worldwide and the one most likely to be encountered in patients in North America. The skin lesions of CL are usually painless and chronic, often occurring at sites of infected sand fly bites. Slow spontaneous healing as cell-mediated immunity develops is the usual natural history, accelerated by antileishmanial therapy. A minority of cutaneous infections caused by *Leishmania* (*Viannia*) *braziliensis* (*L.* [*V.*] *braziliensis*) and related species in the *Viannia* subgenus, including *L.* (*V.*) *panamensis* and *L.* (*V.*) *guyanensis*, are associated with concomitant or late mucosal leishmaniasis (ML), which can cause destructive lesions of the naso-oropharyngeal/laryngeal mucosa. No universally applicable treatment has been identified for CL; the choice of agent, dose, and duration of therapy should be individualized. Parasite and host factors must be considered, as well as clinical characteristics (Table 1).

Visceral leishmaniasis (VL), which reflects dissemination of *Leishmania* parasites throughout the reticuloendothelial system, is potentially life threatening without treatment. VL is an opportunistic infection in persons with HIV/AIDS or other causes of cell-mediated immunosuppression.

The primary goals of therapy for VL and CL/ML are to prevent mortality and morbidity, respectively. The only Food and Drug Administration (FDA)-approved medications for the treatment of leishmaniasis are intravenous liposomal amphotericin B (L-AmB) for VL and oral miltefosine for CL, ML, and VL caused by particular species. For prevention of leishmaniasis in travelers, no vaccines or chemoprophylaxis currently are available; personal protective measures to minimize exposure to sand fly bites are recommended.

Our recommendations for the diagnosis and clinical management of leishmaniasis are listed below. Background information about leishmaniasis, a description of our methods, and the evidence summaries that support our recommendations can be found online in the full text, tables, figures, and appendix of the guidelines.

## Recommendations for the Diagnosis of Leishmaniasis (Cutaneous, Mucosal, and Visceral)

**I. In a person with a compatible skin lesion(s) and exposure history, what specimen(s) should be collected for diagnostic testing for CL?**

*Recommendations*

1.  Tissue specimens should be collected from a lesion(s) when a clinical suspicion for CL exists. Full-thickness skin biopsy specimens allow for simultaneous testing for other diagnoses, such as by histopathology and cultures [Strong, moderate].
2.  Obtain a sample from a cleansed lesion, from which cellular debris and eschar/exudates have been removed [Strong, very low].

**II. In a person with manifestations suggestive of New World mucosal leishmaniasis (ML), what types of specimens should be obtained for diagnostic testing?**

*Recommendations*

3.  The initial and most prominent mucosal manifestations typically are nasal (e.g., chronic unexplained congestion/secretions). Oral/palatal, pharyngeal, and laryngeal involvement may develop as ML progresses or, in some persons, may be the first or the only noted abnormalities. The clinical signs, which may evolve over time, may include erythema, edema, hyperemia, infiltration, nodules, erosion, ulceration, and tissue destruction (e.g., perforation of the nasal septum) [FACT, no grade].
4.  Mucosal areas that have macroscopic abnormalities are recommended for specimen collection; biopsy specimens, obtained by an otolaryngologist, are useful for confirming the diagnosis by molecular and traditional methods and for excluding other etiologies [Strong, low].

**III. During the initial and subsequent evaluations of persons with CL acquired in Central or South America who may have or be at risk for mucosal leishmaniasis (ML), what should be done to assess the possibility of mucosal disease?**

*Recommendations*

5.  All persons at risk for ML—on the basis of the etiologic agent of the *Leishmania* infection, if known, and the region in the New World in which infection was acquired—should be questioned about and examined for mucosal symptoms and signs, respectively, even during the initial evaluation [Strong, low].
6.  During all evaluations (i.e., initial and subsequent), persons at risk for ML should be questioned explicitly about the development, evolution, and other characteristics of mucosal symptoms; and they should have a thorough examination of the naso-oropharyngeal mucosa even if they do not have any mucosal symptoms [Strong, low].
7.  Persons at risk for ML should be educated and provided personalized documentation about the importance of seeking medical attention for possible ML if they ever develop persistent, atypical (unusual for the person) naso-oropharyngeal/laryngeal manifestations that do not have a clear etiology [Strong, low].
8.  Persons at risk for ML who have persistent mucosal symptom(s) or compatible abnormalities of the naso-oropharyngeal mucosa should be referred to a specialist for an otorhinolaryngologic examination, which typically should include fiberoptic endoscopy [Strong, low].
9.  Clinicians might refer some at-risk persons without documented mucosal symptoms or signs to an otolaryngologist, especially if it was not possible to conduct a thorough review of systems and mucosal examination or if the assessments may not have been adequate or reliable [Weak, very low].

**IV. In a person with a compatible clinical course and epidemiologic context, what types of samples should be collected to evaluate for the diagnosis of VL?**

*Recommendations*

10.  We recommend the collection of tissue aspirates or biopsy specimens for smears, histopathology, parasite culture, and molecular testing [Strong, low].
11.  Bone marrow aspiration is the preferred first source of a diagnostic sample. Liver, enlarged lymph nodes, and whole blood (buffy coat) are other potential sources of tissue specimens [Strong, low].
12.  Serum should be collected for detection of antileishmanial antibodies (see VIII) [Strong, moderate].
13.  In immunocompromised persons, blood should be collected for buffy coat examination, *in vitro* culture, and molecular analyses [Strong, very low].

**V. What laboratory tests should be used to diagnose leishmaniasis?**

*Recommendations*

14.  We recommend using multiple diagnostic approaches to maximize the likelihood of a positive *Leishmania* result, using methods such as visualization of the characteristic amastigote in smears or tissue (histopathology); parasite isolation by *in vitro* culture; molecular detection of parasite DNA; and, for VL, serologic testing (see VI–VIII and Table 2). Simultaneous testing for other diagnoses (e.g., by histopathology and culture) should be considered [Strong, low].
15.  We recommend attempting parasite isolation with the assistance of reference laboratories. We recommend that clinicians contact their leishmaniasis reference laboratory before collecting specimens (Table 2). If *Leishmania* parasites are isolated in culture, reference laboratories can identify the species by DNA-based assays or isoenzyme analysis [Strong, low].
16.  Molecular amplification assays typically should be performed because they are the most sensitive *Leishmania* tests currently available (see VII) [Strong, moderate].
17.  *Leishmania* skin testing is not recommended or available in the United States or Canada; there are no standardized, approved, or commercially available skin-test products in North America [Strong, very low].

**VI. In a person with leishmaniasis, why could it be helpful to identify the infecting *Leishmania* species?**

*Recommendation*

18.  We suggest that identification of the infecting parasite to the species level be attempted in cases of suspected CL. Species identification may help inform clinical management decisions for individual persons (e.g., whether and how to treat) [Weak, moderate].

**VII. What is the role of DNA-based assays in the diagnosis of leishmaniasis?**

*Recommendation*

19.  DNA-based assays should be performed, especially if other diagnostic testing is unrevealing. They are emerging as the most sensitive assays for the diagnosis of leishmaniasis [Strong, moderate].

**VIII. What is the role of serologic testing in the diagnosis of leishmaniasis?**

*Recommendations*

20.  Serologic testing is recommended for persons with suspected VL in whom definitive diagnostic tests for the parasite (microscopic identification, culture, and molecular tests for parasite DNA) cannot be conducted or have negative results. The sensitivity and specificity of serologic tests depend on the assay and antigens used, as well as host factors. Serologic tests cannot be used to assess the response to treatment. Antileishmanial antibodies can be detected years after clinically successful therapy in some persons [Strong, moderate].
21.  We suggest that tests for antileishmanial antibodies not be performed as the sole diagnostic assay. Antibodies may be undetectable or present at low levels in persons with VL who are immunocompromised because of concurrent HIV/AIDS or other reasons. The potential for false-negative test results limits the utility of serologic assays in this setting [Weak, low].
22.  Serologic testing is not recommended as part of the diagnostic evaluation for CL. The currently available serologic assays are neither sensitive nor specific for the diagnosis of CL [Strong, moderate].

## Recommendations for the Treatment of Leishmaniasis

### Cutaneous Leishmaniasis

**IX. In a person with a consistent travel history and compatible skin lesion(s), is it necessary to obtain parasitologic confirmation of the diagnosis of leishmaniasis before starting treatment?**

*Recommendation*

23.  After a careful diagnostic evaluation in which neither leishmaniasis nor another diagnosis is confirmed, empiric treatment may be indicated on the basis of an individualized risk-benefit assessment [Weak, very low]. *Remark: This should be discussed with the patient and reevaluated periodically, taking into account the clinical evolution.*

**X. Is treatment of clinically manifest cutaneous infection (CL) always indicated?**

*Recommendations*

**Figure 2:**
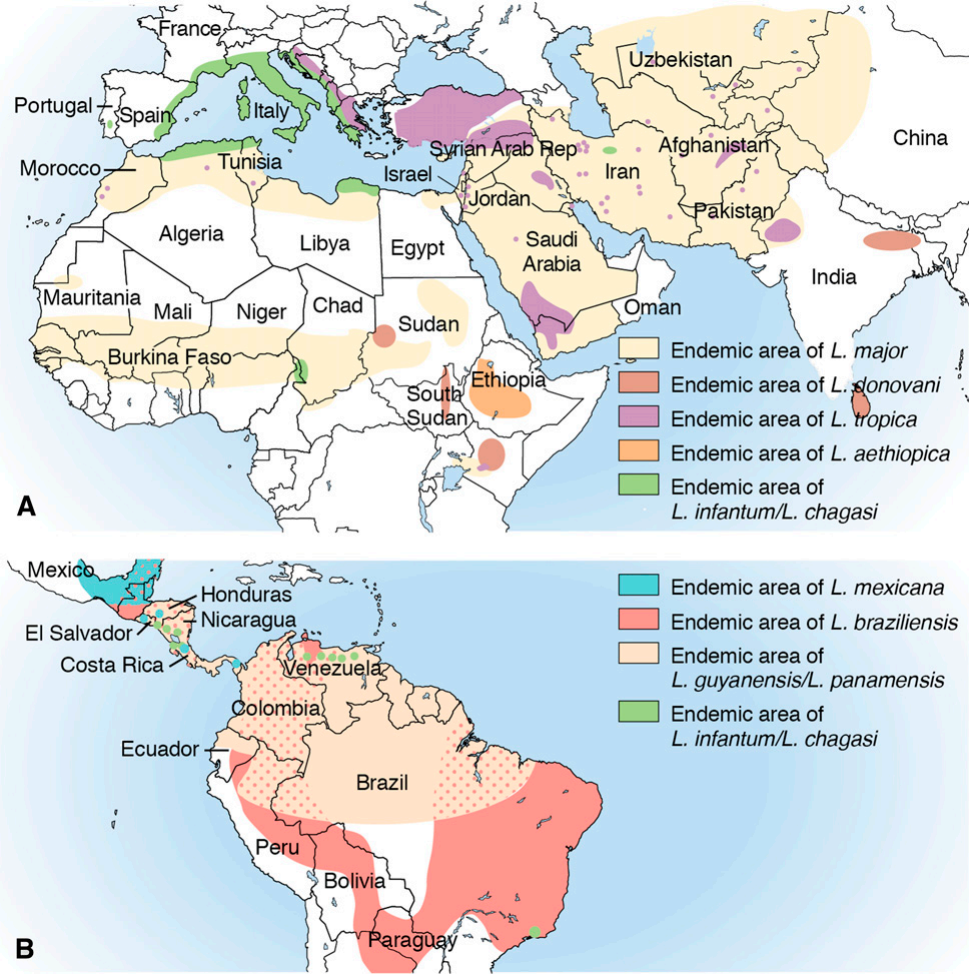
Maps of the Geographic Distribution of Cutaneous Leishmaniasis (CL). Notes: ***Adapted and modified from Chapter 277, Leishmania species. Principles and Practice of Infectious Diseases [31]*** ^1^In Guatemala, the reported cases of CL have been acquired in the northern departments (particularly, El Petén and Alta Verapaz but also Izabal, El Quiché, Baja Verapaz, and Jalapa). ^2^The etiologic agents of CL in Israel primarily include *L. major* and *L. tropica* but also *L. infantum-chagasi*. ^3^The species *L.* (*Leishmania*) *martiniquensis*, which was formally named in 2014, has been identified as the etiologic agent of cutaneous and visceral leishmaniasis in the French West Indies (Martinique Island) and Thailand, where it previously was referred to as “*L. siamenensis*” (not considered a taxonomically valid name). ^4^In Sri Lanka, *L. donovani* has been identified as the etiologic agent of cutaneous and visceral leishmaniasis. ^5^Not all *Leishmania* species that cause CL are included in this map (eg, *L. amazonensis* in South America).

24.  We recommend that immunocompetent persons with skin lesions that are caused by infection with *Leishmania* species that are not associated with increased risk for ML, that are defined as clinically simple lesions (Table 1), and that are healing spontaneously may be observed without treatment if the patient concurs with this management [Strong, moderate].
25.  For persons with CL when the *Leishmania* species is not known but the infection was not acquired in an increased ML-risk region (Table 1, Figure 2

), treatment of clinically simple or healing skin lesions is not required in an immunocompetent patient who concurs with this management [Strong, low; E.C. dissents, recommending that all persons with NWCL receive treatment]. *Remark: See XXIV and XXV regarding the management of CL in immunocompromised persons*.
26.  We suggest that systemic treatment be offered for persons even with healing/recently healed CL lesions caused by increased ML-risk species or when the species is unknown but the infection was acquired in an increased ML-risk region. Risks and benefits of such treatment should be discussed with the patient [Weak, low]. *Remark: In some cases, watchful waiting, with vigilance for signs and symptoms of ML, may be a reasonable approach.*
27.  We recommend that any decision to observe a patient with CL without treatment should be reevaluated periodically, and the decision not to treat should be reconsidered if healing does not progress as anticipated [Strong, very low].
28.  In all cases of CL, wound care, individualized documentation of lesion evolution, and patient education regarding the manifestations and detection of local therapeutic failure/relapse and ML should be routine components of management (see III and XV) [Strong, low].

**XI. In a person with CL, what could be the consequences of no treatment or suboptimal therapy, and how should persons who received no or suboptimal therapy be monitored?**

*Recommendations*

29.  Potential consequences of inadequate treatment include poor cosmetic outcome due to scarring or superinfection, the persistence of a chronic wound(s), and, with some *Leishmania* species, destructive and disfiguring ML. In immunocompromised persons, cutaneous, mucosal, and visceral dissemination may occur [FACT, no grade].
30.  Persons with CL should be actively monitored by clinical appearance, including by performing a careful nasal and oropharyngeal examination periodically up to 1 year, or at least 2 years if at increased risk for ML. They should be educated about the signs and symptoms of relapse and ML and instructed to seek medical attention anytime these appear [Strong, low].
31.  Symptoms such as chronic nasal stuffiness, epistaxis, or hoarseness or findings such as septal perforation that occur anytime in a person with a prior or current diagnosis of CL or a scar consistent with prior CL should prompt evaluation for ML, including fiberoptic examination of the affected area if relevant (see II and III) [Strong, moderate].

**XII. In a person with CL, what factors should prompt consideration of use of a systemic (oral or parenteral) agent for initial therapy?**

*Recommendations*

32.  Systemic treatment is recommended for persons with complex CL as defined in Table 1 [Strong, moderate].
33.  Initial systemic therapy (see XIII) may be used in persons with CL in whom it is not practical to use local therapy or (possibly) if more rapid healing of large, cosmetically or functionally concerning lesions is preferred [Weak, very low].
34.  Less common cutaneous syndromes, such as leishmaniasis recidivans (caused by *L. tropica* and occasionally other species), diffuse cutaneous leishmaniasis (caused by *L. mexicana*, *L. amazonensis*, and *L. aethiopica*), and disseminated cutaneous leishmaniasis (caused by *L.* [*V.*] *braziliensis*), usually require systemic therapy [Strong, low].

**XIII. What systemic treatment options are available in North America for CL, and what factors should be considered when selecting a medication for an individual patient?**

*Recommendations*

35.  The parenteral options for systemic therapy currently available in North America include **conventional amphotericin B deoxycholate, lipid formulations of amphotericin B, pentavalent antimonial (Sb^V^) compounds, and pentamidine (listed in alphabetical order). Oral options include miltefosine and the “azole” antifungal compounds, including ketoconazole (if potential benefits outweigh risks for hepatotoxicity and QT prolongation) and fluconazole [FACT, no grade].**
36.  To maximize effectiveness and to minimize toxicity, the choice of agent, dose, and duration of therapy should be individualized [Strong, moderate]. *Remarks: No ideal or universally applicable therapy for CL has been identified. Some therapies/regimens appear highly effective only against certain Leishmania species/strains in certain areas of the world. Both the parasite species and host factors (e.g., comorbid conditions and immunologic status) should be considered.*
37.  Factors that should be considered when selecting CL treatment for an individual patient include the risk for ML; the *Leishmania* strain/species and published response rates for antileishmanial agents in the pertinent geographic region; the potential for adverse events; age extremes; childbearing competence and pregnancy; obesity; hepatic, pancreatic, renal, and cardiac comorbid conditions; preference for and convenience of various routes of administration; the rapidity with which one wishes to control the infection; the impact of lesions on daily activities and patient self-confidence; the patient/provider comfort level with logistics (e.g., Investigational New Drug protocols); and other practical issues (e.g., drug availability, various types of cost, insurance reimbursement) (see XII and XXVI; Tables 3 and 4) [Strong, low].

**XIV. In which clinical settings can local therapy be used effectively in a person with CL?**

*Recommendations*

38.  Local therapy is preferred for treatment of OWCL lesions defined as clinically simple (Table 1) and may be useful for localized NWCL caused by *Leishmania* species not associated with increased risk for ML [Strong, moderate]. *Remark: Local therapy includes heat and cryotherapy, topical ointments/creams with paromomycin and other ingredients, intralesional injections of pentavalent antimonial drugs (±cryotherapy), and photodynamic or laser treatment.*
39.  Eschar(s) overlying ulcers should be debrided before administration of local therapy and any secondary infection managed to maximize treatment effect [Strong, very low].

**XV. What are the recommended timeframes and findings to assess response to treatment in a person with CL?**

*Recommendations*

40.  Response to treatment is assessed by clinical criteria; repeat parasitologic testing is not recommended if the skin lesion appears to be healing [Strong, low]. *Remark: The healing process may continue after the treatment course is completed especially for large ulcerative lesions.*
41.  Persons with CL should have their skin lesions monitored for 6–12 months after treatment for clinical evidence of therapeutic failure, which is initially seen at the border of a healed lesion [Strong, low]. *Remark: The first sign of healing is usually flattening of the skin lesion. By 4–6 weeks after treatment, the lesion size should have decreased by >50%, ulcerative lesions should be reepithelializing, and no new lesions should be appearing. Ulcerative lesions are generally fully reepithelialized and clinically healed by approximately 3 months after treatment.*

**XVI. What are the recommended approaches for additional management in a person with CL that does not respond to therapy?**

*Recommendations*

42.  Additional therapy is recommended (but not necessarily always with a different agent or approach) when there is development of new skin lesions or worsening of existing lesions. Additional therapy is also recommended if there is incomplete healing by 3 months after completion of the treatment course [Strong, low].
43.  We recommend that therapeutic failure be assessed by physical appearance. Relatively little improvement or worsening while on therapy suggests an inadequate response and an alternate treatment approach should be planned [Strong, low]. *Remark: A paradoxical increase in the local inflammatory response may be seen in the first 2–3 weeks of treatment and can be difficult to differentiate from therapeutic failure.*
44.  Consultation with a leishmaniasis expert about other treatment options is recommended for management of persons' lesions associated with therapeutic failure [Strong, very low].

### Mucosal Leishmaniasis

**XVII. What are the treatment options for American (New World) mucosal leishmaniasis (ML)?**

*Recommendations*

45.  All persons with clinically manifest, metastatic, American ML should receive systemic antileishmanial therapy, with the goals of preventing morbidity (e.g., disfigurement) and mortality (e.g., from aspiration pneumonia or respiratory obstruction) [Strong, low].
46.  Before treatment is initiated, a complete examination of the naso-oropharyngeal/laryngeal mucosa should be conducted by a specialist to assess the anatomic extension and clinical severity of the mucosal disease, which have prognostic implications [Strong, moderate].
47.  We recommend inpatient monitoring and prophylactic corticosteroid therapy for persons with laryngeal/pharyngeal disease and increased risk for respiratory obstruction, as indicated by symptoms and otolaryngologic/radiologic examinations, because of the potential for inflammatory reactions after initiation of antileishmanial therapy [Strong, low].
48.  The choice of antileishmanial agent, dose, and duration of therapy for persons with ML should be individualized (Table 3) [Strong, moderate]. *Remarks: The traditional options for ML include treatment with a pentavalent antimonial (Sb^V^) compound (20 mg Sb^V^/kg daily, IV or IM, for 28–30 days) or with amphotericin B deoxycholate (0.5–1.0 mg/kg per dose, IV, daily or every other day, for a cumulative total of ∼20–45 mg/kg). More recently, on the basis of comparatively limited data, the armamentarium has expanded to include lipid formulations of amphotericin B (typically, liposomal amphotericin B [L-AmB], with a cumulative total dose ranging widely from ∼20–60 mg/kg), as well as the oral agent miltefosine (∼2.5 mg/kg per day [maximum, 150 mg/day] for 28 days).*

### Visceral Leishmaniasis

**XVIII. In what circumstances should a person with visceral *Leishmania* infection be treated?**

*Recommendations*

49.  We recommend that persons with clinical abnormalities compatible with VL and laboratory evidence of VL be treated (Table 3) [Strong, moderate].
50.  We suggest that clinicians closely monitor persons with asymptomatic visceral infection and generally initiate therapy only if clinical manifestations of VL develop [Weak, very low].

**XIX. What is the optimal treatment for VL in a symptomatic immunocompetent person (person without an identified immune defect) in North America?**

*Recommendations*

51.  For an immunocompetent person with VL, treatment with liposomal amphotericin B (L-AmB) is recommended. The FDA-approved dosage regimen is 3 mg/kg/day IV on days 1–5, 14, and 21 (total dose, 21 mg/kg) (Table 3) [Strong, high]. *Remarks: Multiple regimens in which the total L-AmB dose is 18–21 mg/kg have been used effectively in regions other than East Africa. Doses of 40 mg/kg or more may be necessary in persons with VL acquired in East Africa. Other lipid-associated formulations of amphotericin B, such as amphotericin B lipid complex and amphotericin B colloidal dispersion, are not generally recommended: they have not been approved by FDA for treatment of VL; and they have been less well studied in VL treatment trials (i.e., bioequivalence has not been established).*
52.  For an immunocompetent person with VL caused by *L. donovani*, acquired in the Indian subcontinent (South Asia), who is ≥12 years of age, weighs ≥30 kg, and is not pregnant or breastfeeding, treatment with the oral agent miltefosine, 2.5 mg/kg per day (maximum, 150 mg, in 3 divided doses) for 28 days, is a possible alternative to L-AmB, particularly in persons weighing <75 kg (see XXVI and Table 3) [Strong, moderate].

**XX. What alternative agent(s) can be used for a person with VL who cannot tolerate liposomal amphotericin B or miltefosine or in whom these agents otherwise are contraindicated?**

*Recommendations*

53.  Pentavalent antimonial therapy (20 mg Sb^V^/kg/day IV or IM for 28 days) is a recommended therapy for immunocompetent persons with VL acquired in areas where the prevalence of antimony-resistant *Leishmania* species is low (<10%) [Strong, high].
54.  We do not recommend switching to amphotericin B deoxycholate in persons with contraindications to, or substantial toxicity with, L-AmB, with the exception of persons who develop liposome-induced complement activation-related pseudoallergy (CARPA). Amphotericin B lipid complex is a consideration in this situation [Strong, low].

**XXI. In persons with VL, what parameters should be used to assess the clinical response to treatment?**

*Recommendations*

55.  Clinical parameters correlate well with parasitologic responses to VL treatment and should be used to monitor the response [Strong, low].
56.  Parasitologic confirmation of response (such as by repeat bone marrow aspiration for microscopy and culture after treatment) is not recommended in a patient showing a timely clinical response. Antibody levels fall but over many months or longer [Strong, moderate].

**XXII. How should a person with VL be treated who does not respond to initial therapy as assessed by these parameters [or who has a relapse]?**

*Recommendations*

57.  Immunocompetent persons with VL who do not respond to therapy with L-AmB should be treated with an alternative drug or with a higher dose or a longer course of L-AmB [Strong, low].
58.  Immunocompetent persons with VL who do not respond to initial therapy with miltefosine or a pentavalent antimonial compound should be treated with L-AmB or an alternative drug if L-AmB is unavailable [Strong, low].
59.  Immunocompetent persons with VL who respond to initial therapy but subsequently have a relapse should be treated with an alternative drug or with another, potentially longer, course of therapy with the initial drug. If L-AmB was the drug used for initial therapy, use of a higher dose can be considered [Strong, low].
60.  Combination therapies may be considered but have not been well studied after therapeutic failure in persons with VL [Weak, low].

## Leishmaniasis in Immunocompromised Hosts

**XXIII. How should HIV/AIDS-associated visceral leishmaniasis (VL) be treated in persons in North America, and what other management issues should be considered?**

*Recommendations*

61.  Liposomal amphotericin B (L-AmB) is recommended for the treatment of VL in immunocompromised persons in North America (Table 3) [Strong, low]. *Remark: The FDA-approved dosage regimen of L-AmB for such persons, including those with concurrent HIV/AIDS, is 4 mg/kg/day IV, on days 1–5, 10, 17, 24, 31, and 38 (10 doses over a 38-day period), for a total dose of 40 mg/kg.*
62.  Combination therapy (e.g., L-AmB plus miltefosine) might be considered, especially for persons with refractory cases of VL [Weak, very low]. *Remark: The efficacy and optimal duration of miltefosine monotherapy (and combination therapy) for HIV/AIDS-associated VL have not been established.*
63.  Because of the importance of effective immune reconstitution in HIV-VL-coinfected persons, antiretroviral therapy (ART) should be initiated or optimized as soon as the person is sufficiently stable to tolerate it (e.g., either during or soon after the initial course of therapy for VL) [Strong, low].
64.  *Leishmania* infection that becomes clinically manifest or worsens after initiation of ART should be treated with antileishmanial (and, if indicated, corticosteroid) therapy; leishmaniasis-associated immune reconstitution inflammatory syndrome (IRIS) reactions after initiation of ART have been reported occasionally [Strong, very low].
65.  We recommend administering secondary prophylaxis (chronic maintenance therapy) to decrease the risk for posttreatment relapse of VL in persons with HIV/AIDS-associated immunosuppression (e.g., CD4 T-lymphocyte cell counts <200 cells/mm^3^) [Strong, moderate].
66.  Persons with VL-HIV/AIDS coinfection should be monitored indefinitely (until effective immune reconstitution) for evidence of posttreatment relapse; ART and secondary prophylaxis provide only partial protection against relapse. Antileishmanial treatment is indicated for persons who have clinical and parasitologic evidence of recurrence [Strong, low].

**XXIV. How should HIV/AIDS-associated CL or ML be treated in persons in North America who do not have evidence of VL, and what other management issues should be considered?**

*Recommendations*

67.  In HIV/AIDS-associated CL/ML, systemic antileishmanial therapy is recommended, particularly in persons who are moderately to severely immunosuppressed (e.g., have CD4+ T-lymphocyte cell counts <200–350 cells/mm^3^), who may be at increased risk for suboptimal therapeutic responses, for posttreatment relapses, and for cutaneous, mucosal, or visceral dissemination [Strong, very low].
68.  The systemic regimens used for CL/ML in otherwise comparable immunocompetent persons typically are recommended for the initial treatment of coinfected persons, taking into account the potentials for drug interactions and toxicity (Tables 3 and 4) [Strong, very low]. *Remark: Whether coinfected persons who experience multiple posttreatment relapses of CL/ML would benefit from secondary prophylaxis (chronic maintenance therapy) has not yet been established.*
69.  Antiretroviral therapy (ART) should be initiated or optimized in accordance with standard practice for HIV/AIDS; no evidence-based, CL/ML-specific recommendations regarding ART have been established [Strong, low].

**XXV. What is the preferred treatment of visceral/cutaneous leishmaniasis in immunocompromised hosts with solid organ transplant, persons with lymphatic-hematologic malignancies, or persons receiving immunosuppressive therapy for other reasons?**

*Recommendations*

**Figure 3:**
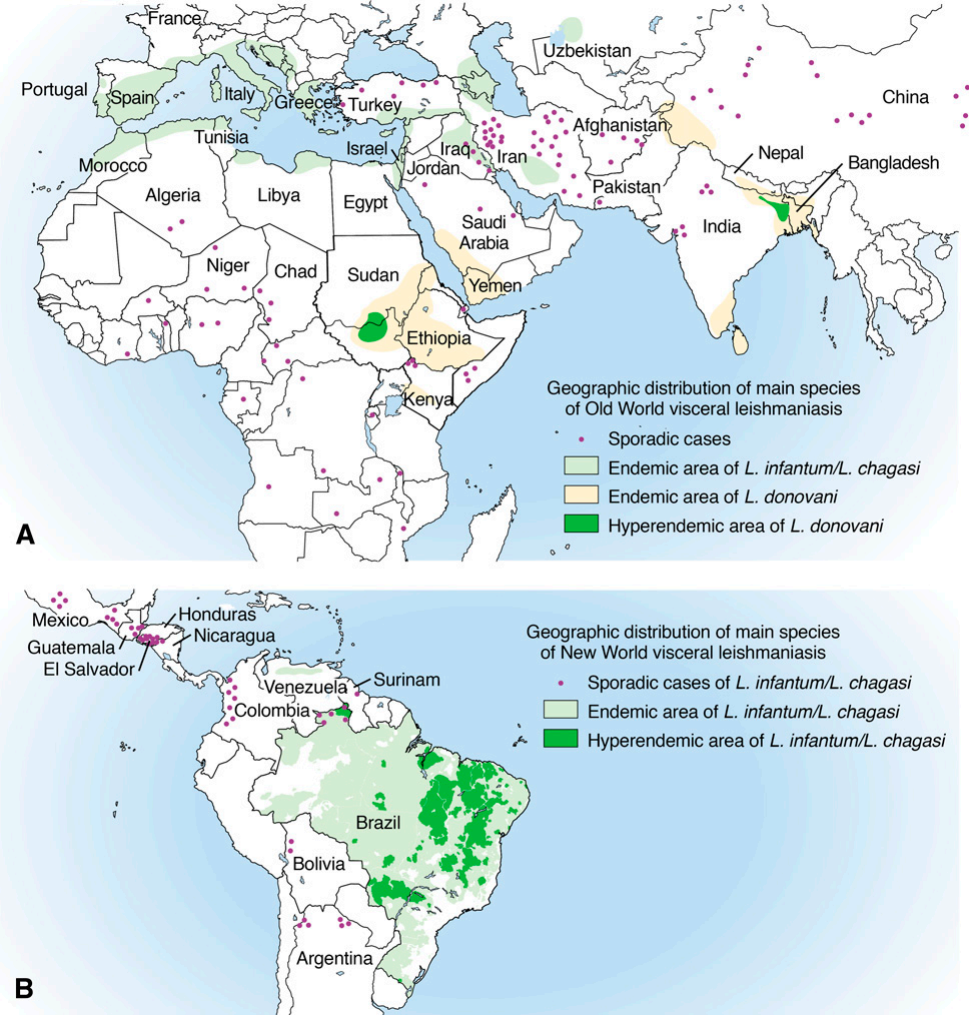
Maps of the Geographic Distribution of Visceral Leishmaniasis (VL)

70.  Liposomal amphotericin B (L-AmB) is recommended as the drug of choice for immunosuppressed persons with VL (Table 3) [Strong, low]. *Remarks: The FDA-approved regimen is 4 mg/kg/day IV on days 1–5, 10, 17, 24, 31, and 38 (total dose of 40 mg/kg). Higher doses and longer durations of therapy may be needed depending in part on the level of immunosuppression.*
71.  Doses of immunosuppressive drugs should be decreased in persons with VL during antileishmanial therapy whenever possible [Strong, very low].
72.  Secondary prophylaxis is not recommended for initial management in persons with VL who have not manifested a relapse [Weak, low]. *Remark: Immunosuppressed persons with VL who are not coinfected with HIV typically have higher response rates to initial treatment and lower recurrence rates than HIV-coinfected persons.*
73.  Routine serologic screening of organ donors from leishmaniasis-endemic areas is not recommended. If an available donor is known to be seropositive, it is advisable to perform clinical and laboratory monitoring of the recipient in the post-transplant period rather than to reject the organ for transplant [Strong, low].
74.  We suggest that clinicians not routinely perform diagnostic testing to assess persons for evidence of asymptomatic visceral infection, including persons who have lived or traveled in leishmaniasis-endemic regions (Figure 3

) and are considering organ transplantation or initiation of therapy with biologic immunomodulating agents. Immunosuppressed persons known or found to be asymptomatically infected and those with a history of VL warrant close monitoring. Neither preemptive treatment nor primary prophylaxis for VL in asymptomatically infected persons is suggested [Weak, very low].
75.  Immunosuppressed persons with VL who are not coinfected with HIV should be monitored for a minimum of 1 year (ideally lifelong or until effective immune reconstitution) to assess for posttreatment relapse. During clinical follow-up, assess for symptoms and, if present, pursue parasitologic confirmation of relapse [Strong, very low].
76.  Confirmed VL therapeutic failure typically can be managed by retreatment using L-AmB at the same or a higher total dose [Strong, very low]. *Remark: Pentavalent antimonials could be used in some persons with VL under close follow-up.*
77.  We suggest that CL/ML associated with the use of tumor necrosis factor (TNF)-alpha antagonist therapy be managed with systemic therapy and standard drug regimens for the pertinent setting/species (e.g., geographic area where the infection was acquired) [Weak, very low]. *Remark: Withdrawal of TNF-α antagonists during antileishmanial therapy may be appropriate: the risks, benefits, and feasibility of this action should be assessed on a case-by-case basis.*

## Special Populations and Leishmaniasis

**XXVI. How should the treatment of leishmaniasis be modified in persons who are pregnant or lactating; are young children or older adults; or have comorbidities such as renal, hepatic, or cardiac dysfunction?**

*Recommendations*

78.  In general, clinically manifest cases otf VL and ML should be treated even in these special populations of persons because the benefits of treatment typically outweigh the risks. However, patient-specific factors, including the presence of comorbid conditions, should be considered in the selection of the antileishmanial therapy, dosage regimen, and monitoring approach (Table 4) [Strong, low].
79.  Decisions regarding whether and how to treat cases of CL in persons with special characteristics or comorbid conditions should take into account the potential risks and benefits of various approaches, such as initially observing without antileishmanial therapy, deferring treatment (e.g., until after pregnancy/delivery), and using local (vs systemic) therapy as the sole approach or as a temporizing measure, if otherwise appropriate and feasible [Strong, very low].

## Supplementary Material

Supplemental Guidelines.

## Figures and Tables

**Table 1:**
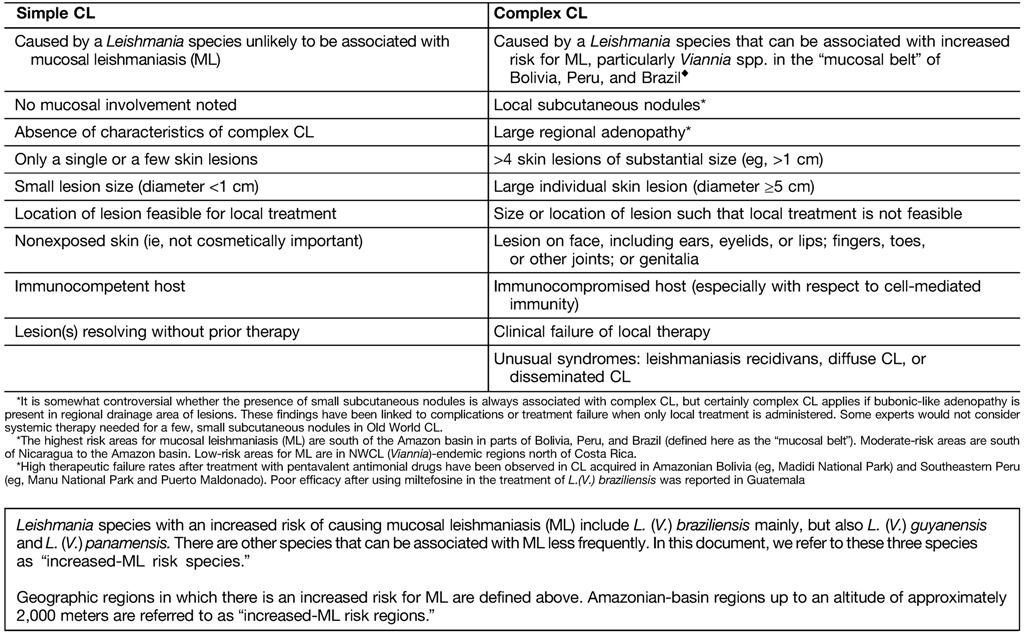
Clinical Characteristics of Cutaneous Leishmaniasis (CL) that may Modify Management in North America

**Table 2:**
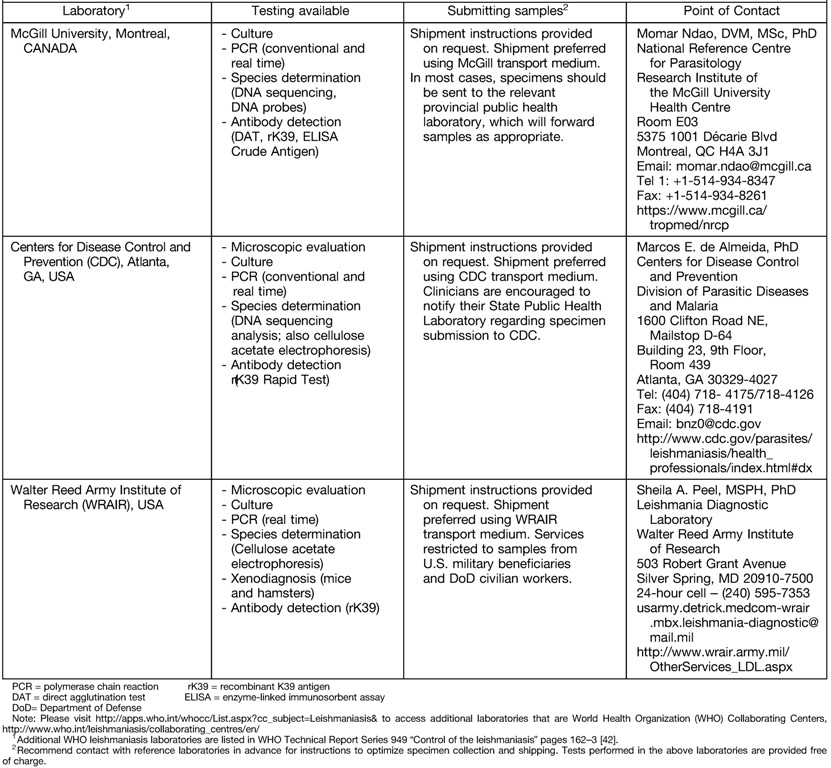
Leishmaniasis Reference Diagnostic Laboratories in North America

**Table 3:**
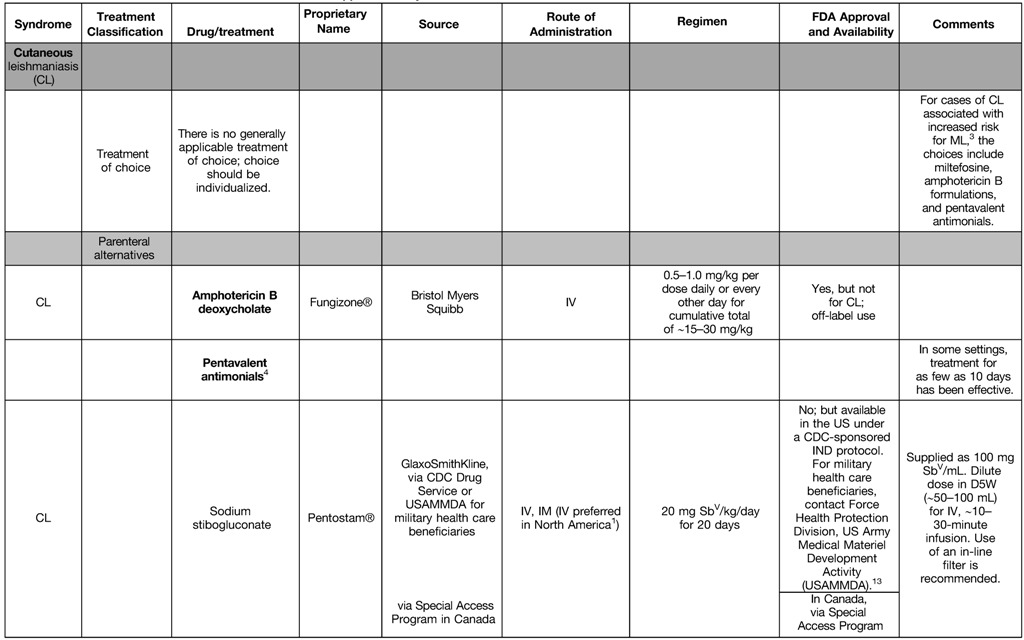

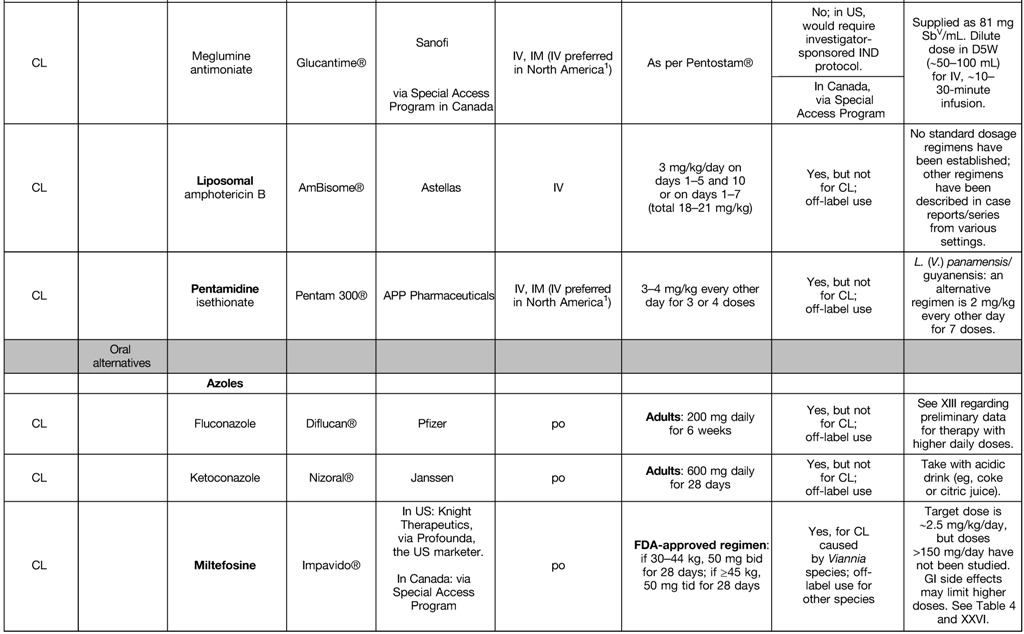

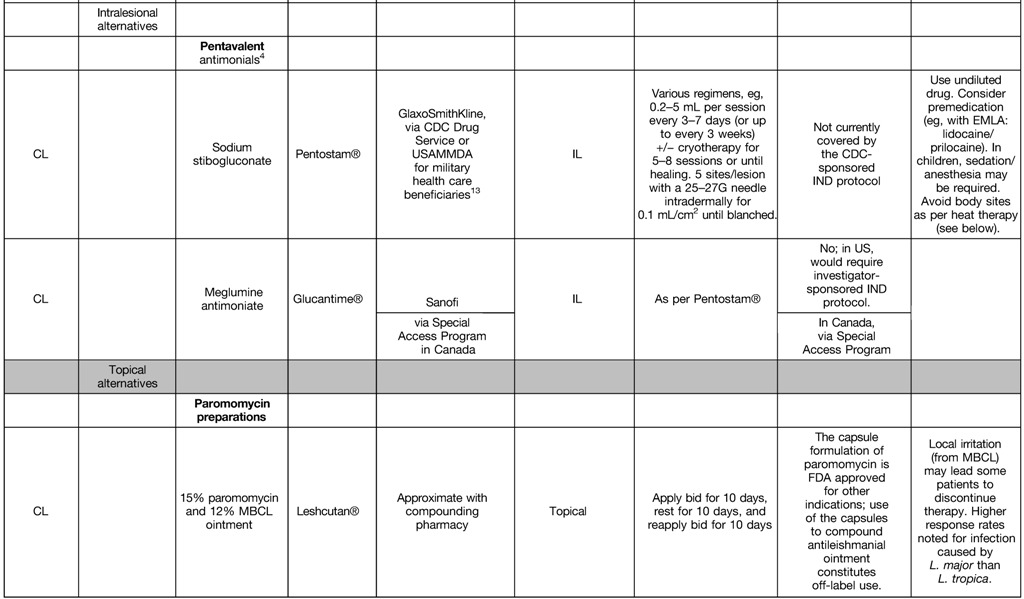

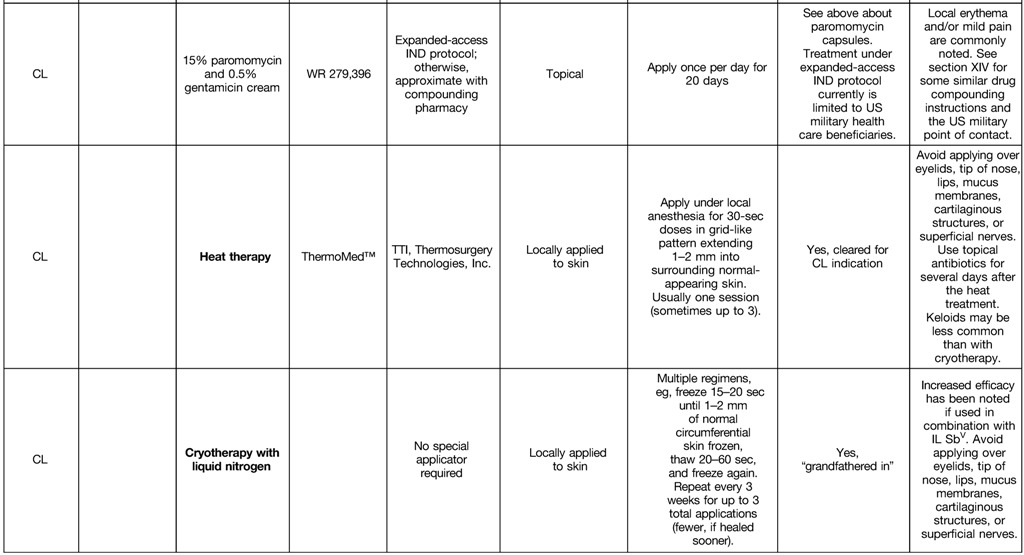

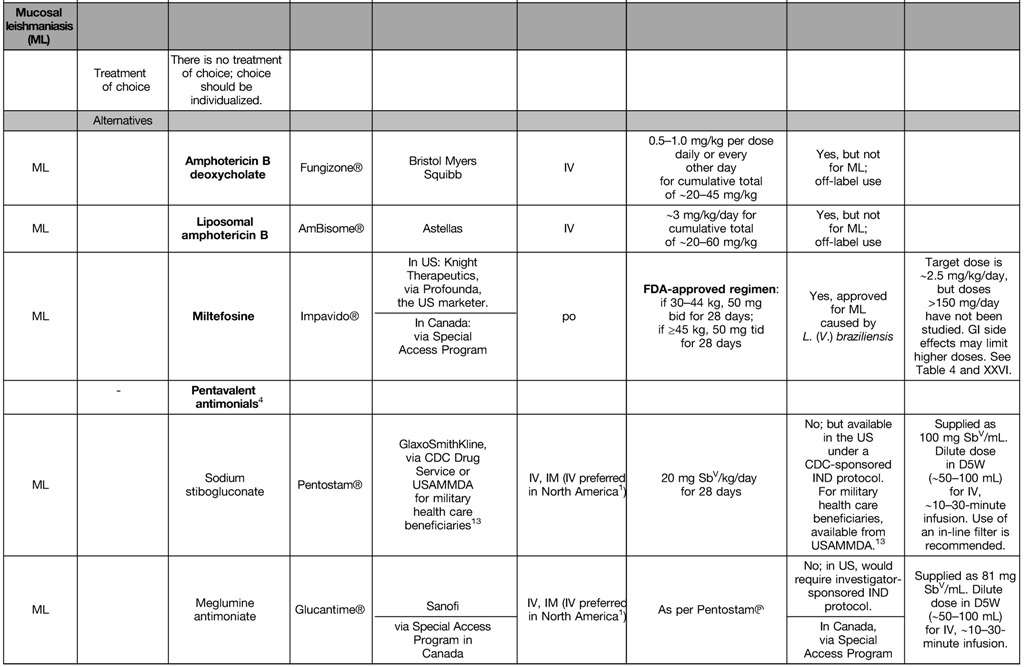

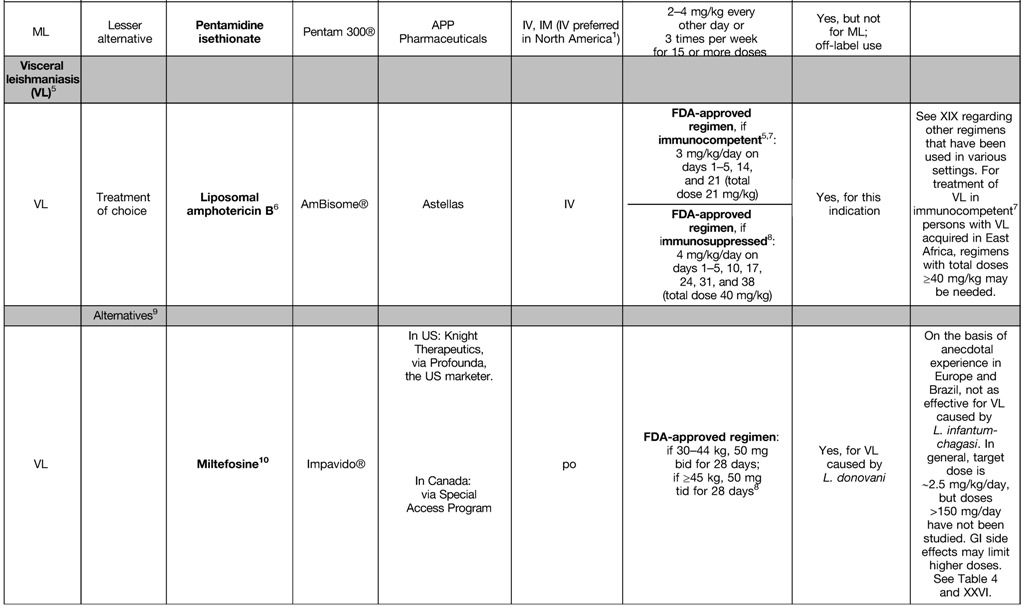

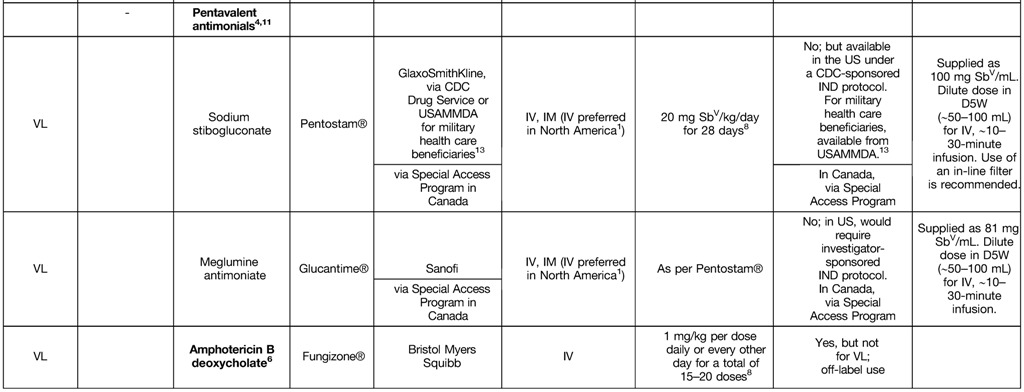

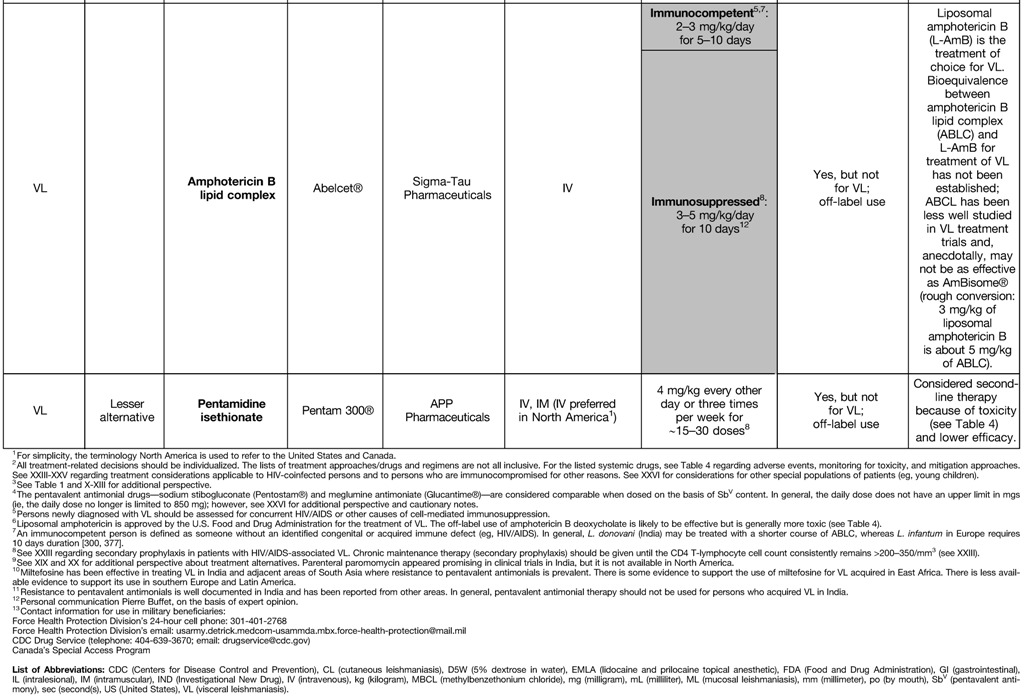
Approach to Syndromic Treatment of Leishmaniasis in North America^1,2^

**Table 4:**
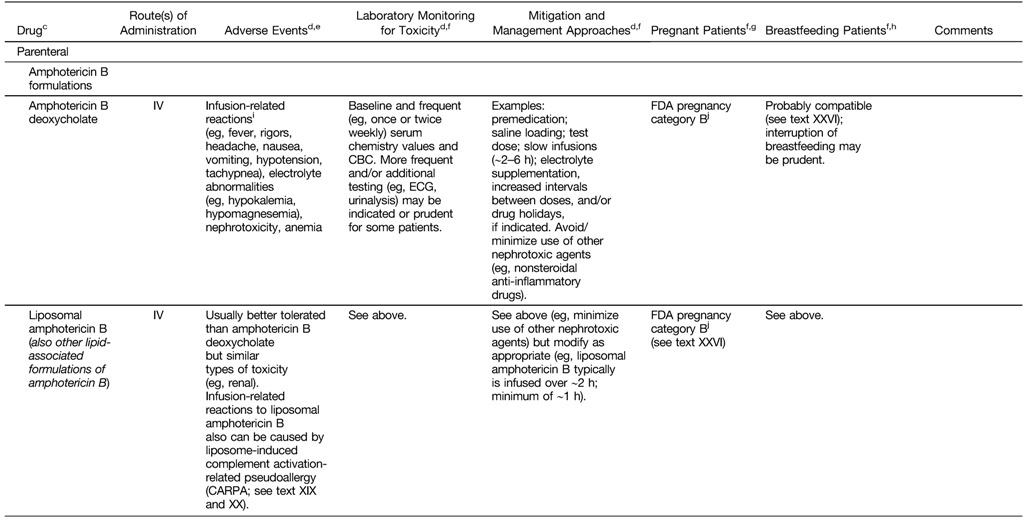

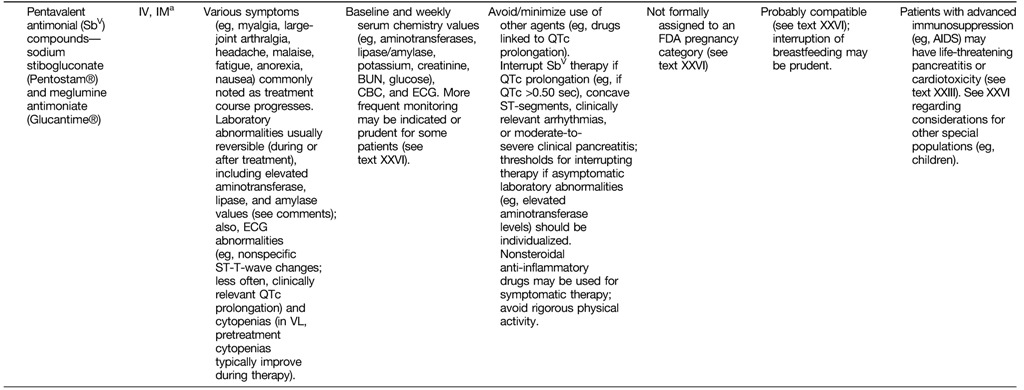

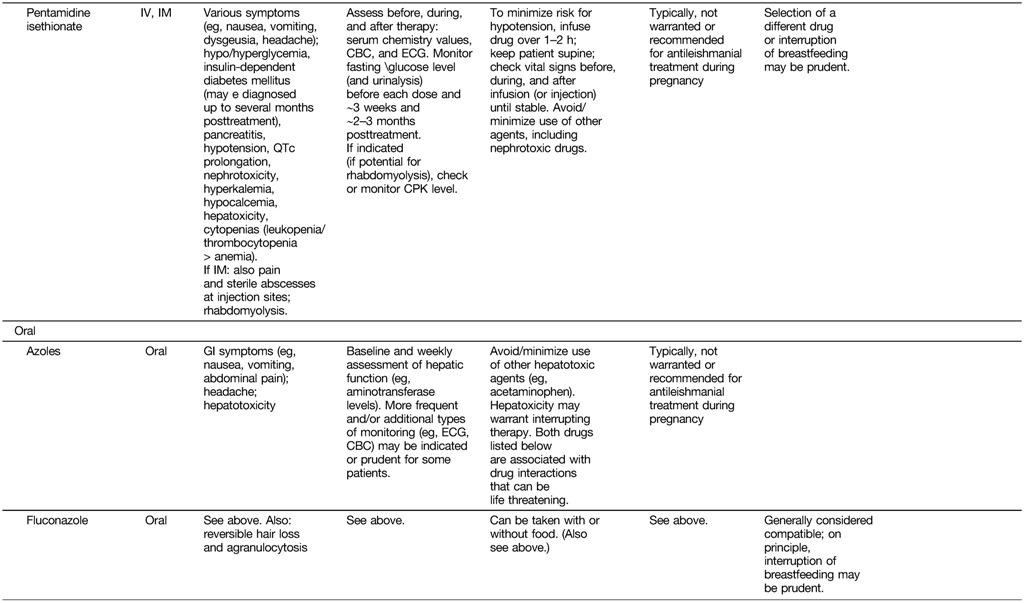

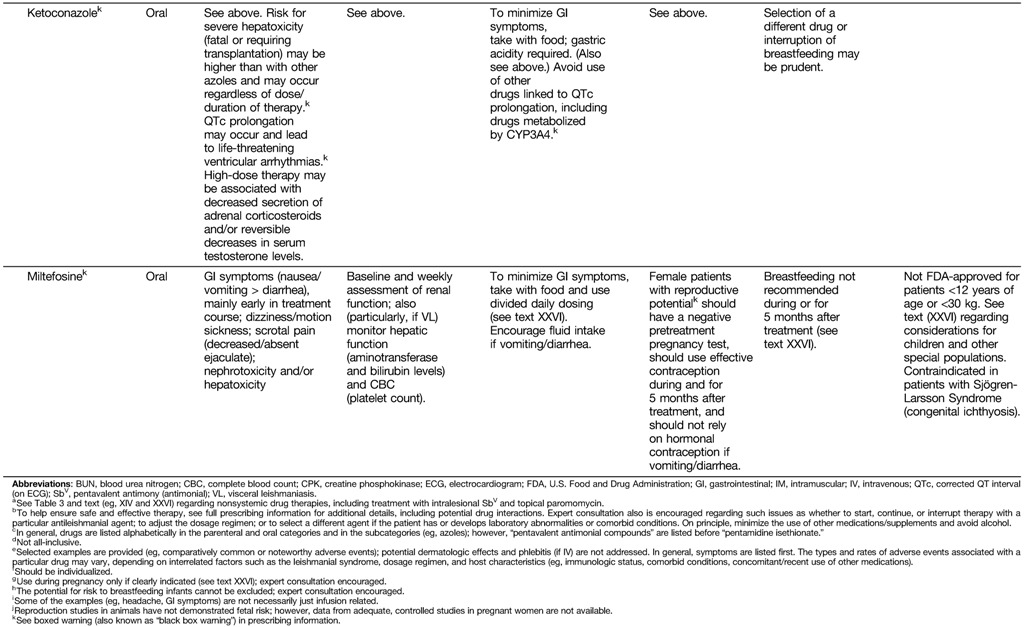
Drugs Used in North America for Systemic^a^ Antileishmanial Therapy: Adverse Events, Monitoring for Toxicity, and Mitigation Approaches^b^
