# Supplementary material for: Diagnosis and Treatment of Leishmaniasis: Clinical Practice Guidelines by the Infectious Diseases Society of America (IDSA) and the American Society of Tropical Medicine and Hygiene (ASTMH)
Source: Am J Trop Med Hyg. 2017 Jan 11;96(1):24–45. doi: 10.4269/ajtmh.16-84256 (PMC5239701; doi:10.4269/ajtmh.16-84256)
Supplement: Supplementary file 1 [file SD1.pdf]

*Am. J. Trop. Med. Hyg.*, 96(1), 2017
doi:10.4269/ajtmh.16-84256

## Guidelines

# Diagnosis and Treatment of Leishmaniasis: Clinical Practice Guidelines by the Infectious Diseases Society of America (IDSA) and the American Society of Tropical Medicine and Hygiene (ASTMH)*

**Naomi Aronson,[1] Barbara L. Herwaldt,[2] Michael Libman,[3] Richard Pearson,[4] Rogelio Lopez-Velez,[5] Peter Weina,[6] Edgar Carvalho,[7] Moshe Ephros,[8] Selma Jeronimo,[9] and Alan Magill[10]**

[1]Uniformed Services University of the Health Sciences, Bethesda, MD; [2]Center for Disease Control and Prevention, Atlanta, GA; [3]McGill University Health Centre, Montreal, Que, Canada; [4]University of Virginia School of Medicine, Charlottesville, VA; [5]University of Alcalá, Madrid, Spain; [6]Walter Reed Army Institute of Research, Silver Spring, MD; [7]Universidade Federal da Bahia, Salvador, BA, Brazil; [8]Carmel Medical Center, Haifa, Israel; [9]Federal University of Rio Grande do Norte, Natal, RN, Brazil; [10]Bill and Melinda Gates Foundation, Seattle, WA

## EXECUTIVE SUMMARY

Guidelines for the clinical management of persons with leishmaniasis were prepared by a Panel of the Infectious Diseases Society of America (IDSA) and the American Society of Tropical Medicine and Hygiene (ASTMH). The guidelines are intended for internists, pediatricians, family practitioners, and dermatologists, as well as infectious disease specialists, practicing in the United States and Canada (for simplicity, referred to here as North America). The Panel followed a guideline development process that has been adopted by IDSA, which includes a systematic method of grading both the quality of evidence (very low, low, moderate, or high) and the strength of the recommendation (weak or strong) [1] (Figure 1).

In these guidelines, we describe our approaches to the diagnosis and management of cases of cutaneous, mucosal, and visceral leishmaniasis, the three main clinical syndromes caused by infection with *Leishmania* parasites. Less common or rare syndromes that may require specialized expertise are beyond the scope of these guidelines. Whenever possible, our recommendations are based on randomized clinical trials. However, because of the diversity encompassed by leishmaniasis, which includes a spectrum of diseases caused by >20 *Leishmania* species found in many areas of the world, many of the recommendations are based on observational studies, anecdotal data, or expert opinion. Although there may be disagreement with some of our recommendations and suggestions, the approaches we describe have been both useful and feasible in North America.

Cutaneous leishmaniasis (CL) is the most common leishmanial syndrome worldwide and the one most likely to be encountered in patients in North America. The skin lesions of CL are usually painless and chronic, often occurring at sites of infected sand fly bites. Slow spontaneous healing as cell-mediated immunity develops is the usual natural history, accelerated by antileishmanial therapy. A minority of cutaneous infections caused by *Leishmania* (*Viannia*) *braziliensis* (*L.* [*V.*] *braziliensis*) and related species in the *Viannia* subgenus, including *L.* (*V.*) *panamensis* and *L.* (*V.*) *guyanensis*, are associated with concomitant or late mucosal leishmaniasis (ML), which can cause destructive lesions of the naso-oropharyngeal/laryngeal mucosa. No universally applicable treatment has been identified for CL; the choice of agent, dose, and duration of therapy should be individualized. Parasite and host factors must be considered, as well as clinical characteristics (Table 1).

Visceral leishmaniasis (VL), which reflects dissemination of *Leishmania* parasites throughout the reticuloendothelial system, is potentially life threatening without treatment. VL is an opportunistic infection in persons with HIV/AIDS or other causes of cell-mediated immunosuppression.

The primary goals of therapy for VL and CL/ML are to prevent mortality and morbidity, respectively. The only Food and Drug Administration (FDA)-approved medications for the treatment of leishmaniasis are intravenous liposomal amphotericin B (L-AmB) for VL and oral miltefosine for CL, ML, and VL caused by particular species. For prevention of leishmaniasis in travelers, no vaccines or chemoprophylaxis currently are available; personal protective measures to minimize exposure to sand fly bites are recommended.

Our recommendations for the diagnosis and clinical management of leishmaniasis are listed below. Background information about leishmaniasis, a description of our methods, and the evidence summaries that support our recommendations can be found online in the full text, tables, figures, and appendix of the guidelines.

## RECOMMENDATIONS FOR THE DIAGNOSIS OF LEISHMANIASIS (CUTANEOUS, MUCOSAL, AND VISCERAL)

### I. In a person with a compatible skin lesion(s) and exposure history, what specimen(s) should be collected for diagnostic testing for CL?

*The content and views expressed in this document are the sole responsibility of the authors and do not necessarily reflect the views or policies of the U.S. Department of Defense, the U.S. Department of Health and Human Services, or the Centers for Disease Control and Prevention. It is important to realize that guidelines cannot always account for individual variation among patients. They are not intended to supplant physician judgment with respect to particular patients or special clinical situations. The IDSA and ASTMH consider adherence to these guidelines to be voluntary, with the ultimate determinations regarding their application to be made by the physician in the light of each patient's individual circumstances.

**Corresponding author:**
Naomi Aronson
Uniformed Services University of the Health Services
Infectious Diseases Division
4301 Jones Bridge Road, Room A3060
Bethesda, MD 20814
TEL: 301 295 3621
FAX: 301 295 3557
e-mail: naomi.aronson@usuhs.edu

**Dedication: The Panel dedicates these guidelines to Alan Magill**

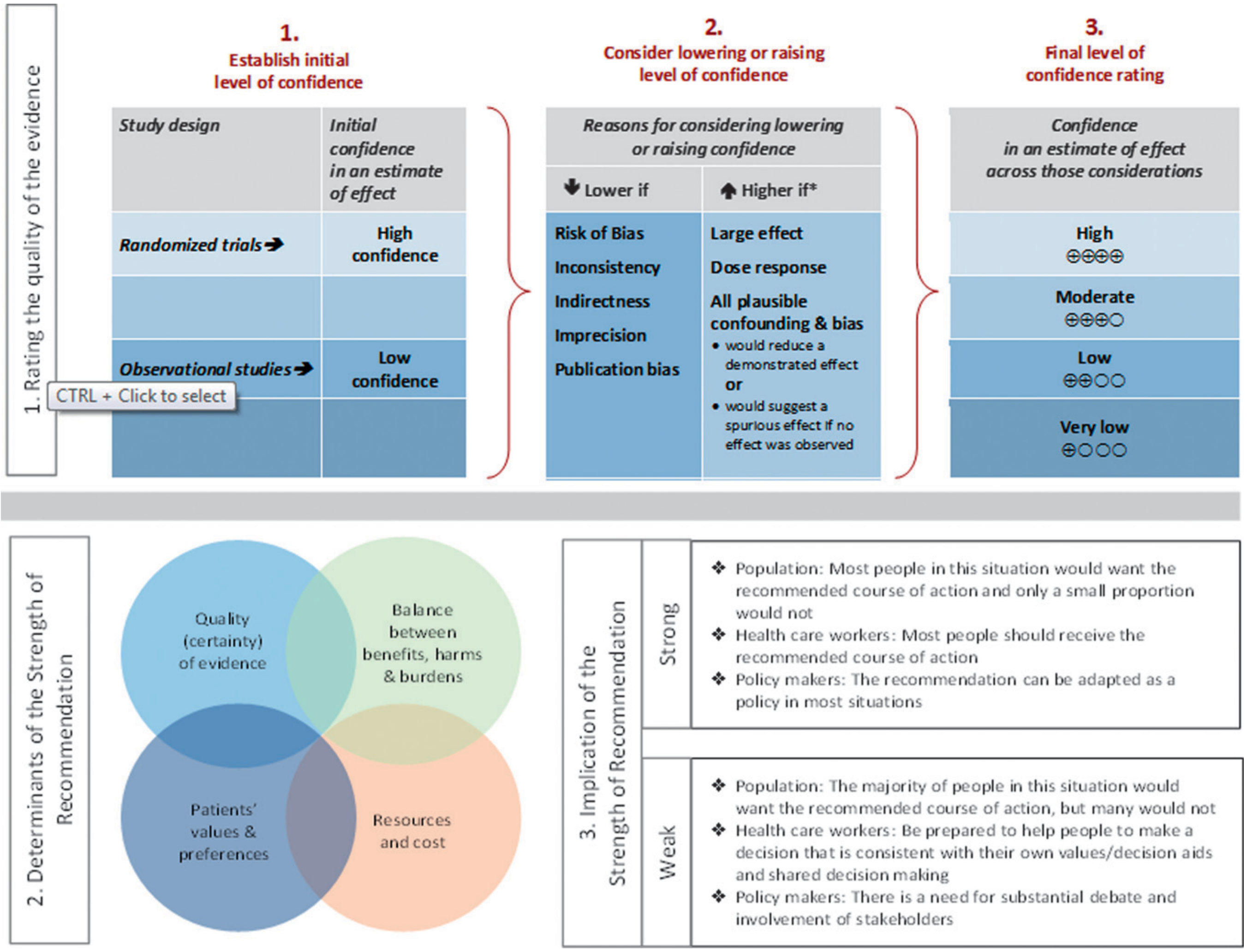


Figure 1: Approach and implications to rating the quality of evidence and strength of recommendations using the GRADE methodology (unrestricted use of the figure granted by the U.S. GRADE Network) [1]

*Recommendations*

1. Tissue specimens should be collected from a lesion(s) when a clinical suspicion for CL exists. Full-thickness skin biopsy specimens allow for simultaneous testing for other diagnoses, such as by histopathology and cultures [Strong, moderate].
2. Obtain a sample from a cleansed lesion, from which cellular debris and eschar/exudates have been removed [Strong, very low].

**II. In a person with manifestations suggestive of New World mucosal leishmaniasis (ML), what types of specimens should be obtained for diagnostic testing?**

*Recommendations*

3. The initial and most prominent mucosal manifestations typically are nasal (e.g., chronic unexplained congestion/secretions). Oral/palatal, pharyngeal, and laryngeal involvement may develop as ML progresses or, in some persons, may be the first or the only noted abnormalities. The clinical signs, which may evolve over time, may include erythema, edema, hyperemia, infiltration, nodules, erosion, ulceration, and tissue destruction (e.g., perforation of the nasal septum) [FACT, no grade].
4. Mucosal areas that have macroscopic abnormalities are recommended for specimen collection; biopsy specimens, obtained by an otolaryngologist, are useful for confirming the diagnosis by molecular and traditional methods and for excluding other etiologies [Strong, low].

**III. During the initial and subsequent evaluations of persons with CL acquired in Central or South America who may have or be at risk for mucosal leishmaniasis (ML), what should be done to assess the possibility of mucosal disease?**

*Recommendations*

5. All persons at risk for ML—on the basis of the etiologic agent of the *Leishmania* infection, if known, and the region in the New World in which infection was acquired—should be questioned about and examined for mucosal symptoms and signs, respectively, even during the initial evaluation [Strong, low].
6. During all evaluations (i.e., initial and subsequent), persons at risk for ML should be questioned explicitly about the development, evolution, and other characteristics of

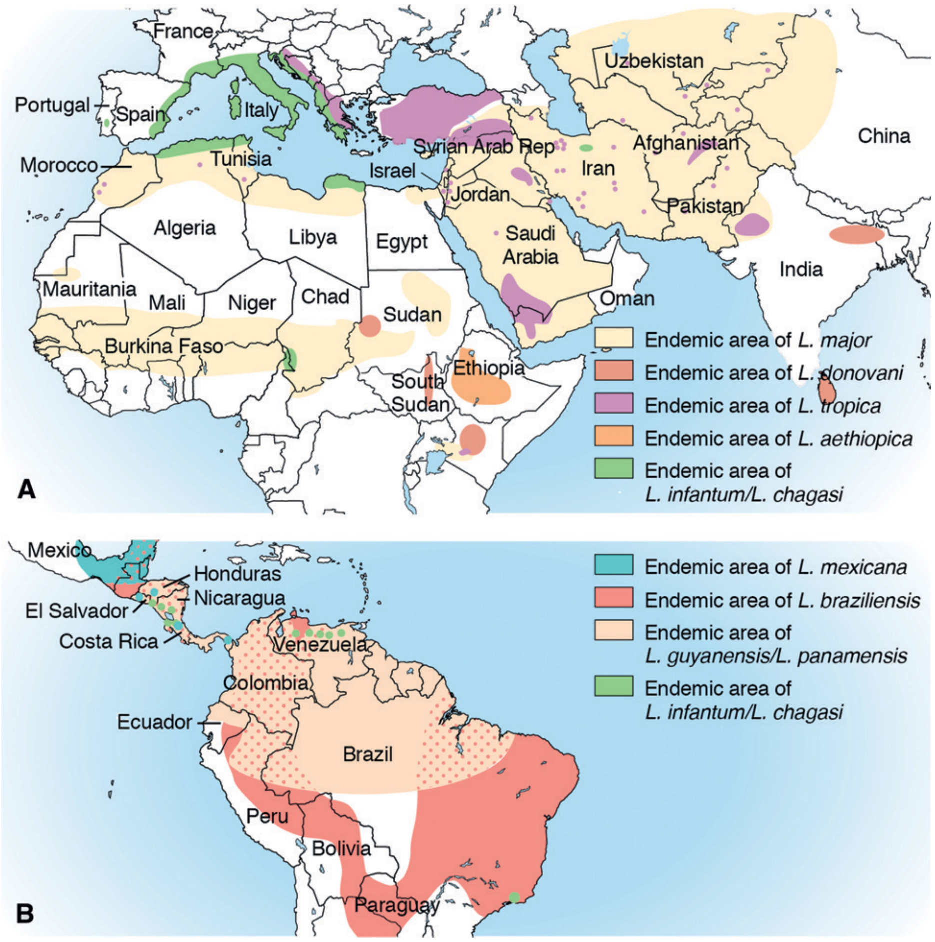


Figure 2: Maps of the Geographic Distribution of Cutaneous Leishmaniasis (CL). Notes: ***Adapted and modified from Chapter 277, Leishmania species. Principles and Practice of Infectious Diseases [31]*** [1]In Guatemala, the reported cases of CL have been acquired in the northern departments (particularly, El Petén and Alta Verapaz but also Izabal, El Quiché, Baja Verapaz, and Jalapa). [2]The etiologic agents of CL in Israel primarily include *L. major* and *L. tropica* but also *L. infantum-chagasi*. [3]The species *L.* (*Leishmania*) *martiniquensis*, which was formally named in 2014, has been identified as the etiologic agent of cutaneous and visceral leishmaniasis in the French West Indies (Martinique Island) and Thailand, where it previously was referred to as "*L. siamenensis*" (not considered a taxonomically valid name). [4]In Sri Lanka, *L. donovani* has been identified as the etiologic agent of cutaneous and visceral leishmaniasis. [5]Not all *Leishmania* species that cause CL are included in this map (eg, *L. amazonensis* in South America).

mucosal symptoms; and they should have a thorough examination of the naso-oropharyngeal mucosa even if they do not have any mucosal symptoms [Strong, low].

7. Persons at risk for ML should be educated and provided personalized documentation about the importance of seeking medical attention for possible ML if they ever develop persistent, atypical (unusual for the person) naso-oropharyngeal/laryngeal manifestations that do not have a clear etiology [Strong, low].
8. Persons at risk for ML who have persistent mucosal symptom(s) or compatible abnormalities of the naso-oropharyngeal mucosa should be referred to a specialist for an otorhinolaryngologic examination, which typically should include fiberoptic endoscopy [Strong, low].
9. Clinicians might refer some at-risk persons without documented mucosal symptoms or signs to an otolaryngologist, especially if it was not possible to conduct a thorough review of systems and mucosal examination or if the assessments may not have been adequate or reliable [Weak, very low].

## IV. In a person with a compatible clinical course and epidemiologic context, what types of samples should be collected to evaluate for the diagnosis of VL?

*Recommendations*

10. We recommend the collection of tissue aspirates or biopsy specimens for smears, histopathology, parasite culture, and molecular testing [Strong, low].
11. Bone marrow aspiration is the preferred first source of a diagnostic sample. Liver, enlarged lymph nodes, and whole blood (buffy coat) are other potential sources of tissue specimens [Strong, low].
12. Serum should be collected for detection of anti-leishmanial antibodies (see VIII) [Strong, moderate].
13. In immunocompromised persons, blood should be collected for buffy coat examination, *in vitro* culture, and molecular analyses [Strong, very low].

**Table 1: Clinical Characteristics of Cutaneous Leishmaniasis (CL) that may Modify Management in North America**

| Simple CL | Complex CL |
|---|---|
| Caused by a *Leishmania* species unlikely to be associated with mucosal leishmaniasis (ML) | Caused by a *Leishmania* species that can be associated with increased risk for ML, particularly *Viannia* spp. in the "mucosal belt" of Bolivia, Peru, and Brazil◆ |
| No mucosal involvement noted | Local subcutaneous nodules* |
| Absence of characteristics of complex CL | Large regional adenopathy* |
| Only a single or a few skin lesions | >4 skin lesions of substantial size (eg, >1 cm) |
| Small lesion size (diameter <1 cm) | Large individual skin lesion (diameter ≥5 cm) |
| Location of lesion feasible for local treatment | Size or location of lesion such that local treatment is not feasible |
| Nonexposed skin (ie, not cosmetically important) | Lesion on face, including ears, eyelids, or lips; fingers, toes, or other joints; or genitalia |
| Immunocompetent host | Immunocompromised host (especially with respect to cell-mediated immunity) |
| Lesion(s) resolving without prior therapy | Clinical failure of local therapy |
| | Unusual syndromes: leishmaniasis recidivans, diffuse CL, or disseminated CL |

*It is somewhat controversial whether the presence of small subcutaneous nodules is always associated with complex CL, but certainly complex CL applies if bubonic-like adenopathy is present in regional drainage area of lesions. These findings have been linked to complications or treatment failure when only local treatment is administered. Some experts would not consider systemic therapy needed for a few, small subcutaneous nodules in Old World CL.

*The highest risk areas for mucosal leishmaniasis (ML) are south of the Amazon basin in parts of Bolivia, Peru, and Brazil (defined here as the "mucosal belt"). Moderate-risk areas are south of Nicaragua to the Amazon basin. Low-risk areas for ML are in NWCL (*Viannia*)-endemic regions north of Costa Rica.

*High therapeutic failure rates after treatment with pentavalent antimonial drugs have been observed in CL acquired in Amazonian Bolivia (eg, Madidi National Park) and Southeastern Peru (eg, Manu National Park and Puerto Maldonado). Poor efficacy after using miltefosine in the treatment of *L.(V.) braziliensis* was reported in Guatemala

*Leishmania* species with an increased risk of causing mucosal leishmaniasis (ML) include *L.* (*V.*) *braziliensis* mainly, but also *L.* (*V.*) *guyanensis* and *L.* (*V.*) *panamensis.* There are other species that can be associated with ML less frequently. In this document, we refer to these three species as "increased-ML risk species."

Geographic regions in which there is an increased risk for ML are defined above. Amazonian-basin regions up to an altitude of approximately 2,000 meters are referred to as "increased-ML risk regions."

## V. What laboratory tests should be used to diagnose leishmaniasis?

*Recommendations*

14. We recommend using multiple diagnostic approaches to maximize the likelihood of a positive *Leishmania* result, using methods such as visualization of the characteristic amastigote in smears or tissue (histopathology); parasite isolation by *in vitro* culture; molecular detection of parasite DNA; and, for VL, serologic testing (see VI–VIII and Table 2). Simultaneous testing for other diagnoses (e.g., by histopathology and culture) should be considered [Strong, low].
15. We recommend attempting parasite isolation with the assistance of reference laboratories. We recommend that clinicians contact their leishmaniasis reference laboratory before collecting specimens (Table 2). If *Leishmania* parasites are isolated in culture, reference laboratories can identify the species by DNA-based assays or isoenzyme analysis [Strong, low].
16. Molecular amplification assays typically should be performed because they are the most sensitive *Leishmania* tests currently available (see VII) [Strong, moderate].
17. *Leishmania* skin testing is not recommended or available in the United States or Canada; there are no standardized, approved, or commercially available skin-test products in North America [Strong, very low].

## VI. In a person with leishmaniasis, why could it be helpful to identify the infecting *Leishmania* species?

*Recommendation*

18. We suggest that identification of the infecting parasite to the species level be attempted in cases of suspected CL. Species identification may help inform clinical management decisions for individual persons (e.g., whether and how to treat) [Weak, moderate].

## VII. What is the role of DNA-based assays in the diagnosis of leishmaniasis?

*Recommendation*

19. DNA-based assays should be performed, especially if other diagnostic testing is unrevealing. They are emerging as the most sensitive assays for the diagnosis of leishmaniasis [Strong, moderate].

## VIII. What is the role of serologic testing in the diagnosis of leishmaniasis?

*Recommendations*

20. Serologic testing is recommended for persons with suspected VL in whom definitive diagnostic tests for the parasite (microscopic identification, culture, and molecular tests for parasite DNA) cannot be conducted or have negative results. The sensitivity and specificity of serologic tests depend on the assay and antigens used,

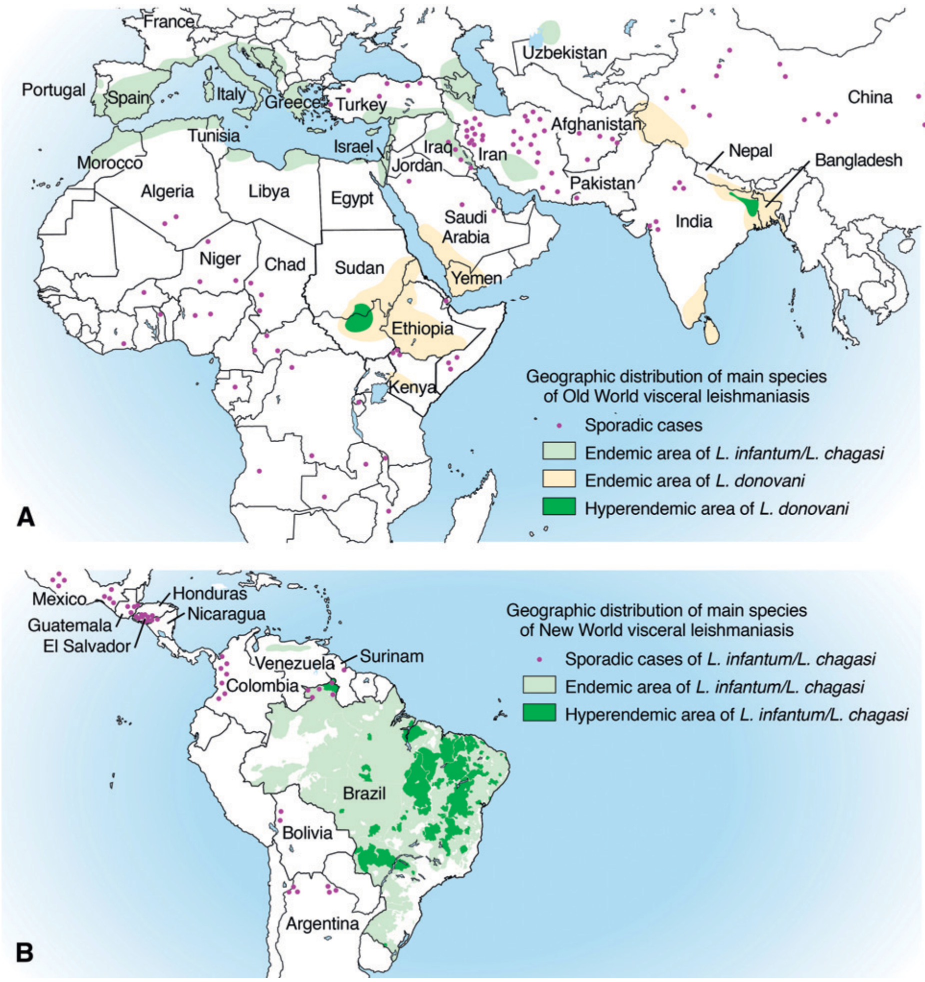


Figure 3. Maps of the Geographic Distribution of Visceral Leishmaniasis (VL)

as well as host factors. Serologic tests cannot be used to assess the response to treatment. Antileishmanial antibodies can be detected years after clinically successful therapy in some persons [Strong, moderate].

21. We suggest that tests for antileishmanial antibodies not be performed as the sole diagnostic assay. Antibodies may be undetectable or present at low levels in persons with VL who are immunocompromised because of concurrent HIV/AIDS or other reasons. The potential for false-negative test results limits the utility of serologic assays in this setting [Weak, low].
22. Serologic testing is not recommended as part of the diagnostic evaluation for CL. The currently available serologic assays are neither sensitive nor specific for the diagnosis of CL [Strong, moderate].

## RECOMMENDATIONS FOR THE TREATMENT OF LEISHMANIASIS

## CUTANEOUS LEISHMANIASIS

### IX. In a person with a consistent travel history and compatible skin lesion(s), is it necessary to obtain parasitologic confirmation of the diagnosis of leishmaniasis before starting treatment?

*Recommendation*

23. After a careful diagnostic evaluation in which neither leishmaniasis nor another diagnosis is confirmed, empiric treatment may be indicated on the basis of an individualized risk-benefit assessment [Weak, very low]. *Remark: This should be discussed with the patient and reevaluated periodically, taking into account the clinical evolution.*

### X. Is treatment of clinically manifest cutaneous infection (CL) always indicated?

*Recommendations*

24. We recommend that immunocompetent persons with skin lesions that are caused by infection with *Leishmania* species that are not associated with increased risk for ML, that are defined as clinically simple lesions (Table 1), and that are healing spontaneously may be observed without treatment if the patient concurs with this management [Strong, moderate].
25. For persons with CL when the *Leishmania* species is not known but the infection was not acquired in an increased ML-risk region (Table 1, Figure 2), treatment of clinically simple or healing skin lesions is not required in an immunocompetent patient who concurs

**Table 2: Leishmaniasis Reference Diagnostic Laboratories in North America**

| Laboratory[1] | Testing available | Submitting samples[2] | Point of Contact |
|---|---|---|---|
| McGill University, Montreal, CANADA | - Culture<br>- PCR (conventional and real time)<br>- Species determination (DNA sequencing, DNA probes)<br>- Antibody detection (DAT, rK39, ELISA Crude Antigen) | Shipment instructions provided on request. Shipment preferred using McGill transport medium.<br>In most cases, specimens should be sent to the relevant provincial public health laboratory, which will forward samples as appropriate. | Momar Ndao, DVM, MSc, PhD<br>National Reference Centre for Parasitology<br>Research Institute of the McGill University Health Centre<br>Room E03<br>5375 1001 Décarie Blvd<br>Montreal, QC H4A 3J1<br>Email: momar.ndao@mcgill.ca<br>Tel 1: +1-514-934-8347<br>Fax: +1-514-934-8261<br>https://www.mcgill.ca/tropmed/nrcp |
| Centers for Disease Control and Prevention (CDC), Atlanta, GA, USA | - Microscopic evaluation<br>- Culture<br>- PCR (conventional and real time)<br>- Species determination (DNA sequencing analysis; also cellulose acetate electrophoresis)<br>- Antibody detection r(K39 Rapid Test) | Shipment instructions provided on request. Shipment preferred using CDC transport medium. Clinicians are encouraged to notify their State Public Health Laboratory regarding specimen submission to CDC. | Marcos E. de Almeida, PhD<br>Centers for Disease Control and Prevention<br>Division of Parasitic Diseases and Malaria<br>1600 Clifton Road NE, Mailstop D-64<br>Building 23, 9th Floor, Room 439<br>Atlanta, GA 30329-4027<br>Tel: (404) 718- 4175/718-4126<br>Fax: (404) 718-4191<br>Email: bnz0@cdc.gov<br>http://www.cdc.gov/parasites/leishmaniasis/health_professionals/index.html#dx |
| Walter Reed Army Institute of Research (WRAIR), USA | - Microscopic evaluation<br>- Culture<br>- PCR (real time)<br>- Species determination (Cellulose acetate electrophoresis)<br>- Xenodiagnosis (mice and hamsters)<br>- Antibody detection (rK39) | Shipment instructions provided on request. Shipment preferred using WRAIR transport medium. Services restricted to samples from U.S. military beneficiaries and DoD civilian workers. | Sheila A. Peel, MSPH, PhD<br>Leishmania Diagnostic Laboratory<br>Walter Reed Army Institute of Research<br>503 Robert Grant Avenue<br>Silver Spring, MD 20910-7500<br>24-hour cell – (240) 595-7353<br>usarmy.detrick.medcom-wrair.mbx.leishmania-diagnostic@mail.mil<br>http://www.wrair.army.mil/OtherServices_LDL.aspx |

PCR = polymerase chain reaction  rK39 = recombinant K39 antigen
DAT = direct agglutination test  ELISA = enzyme-linked immunosorbent assay
DoD= Department of Defense
Note: Please visit http://apps.who.int/whocc/List.aspx?cc_subject=Leishmaniasis& to access additional laboratories that are World Health Organization (WHO) Collaborating Centers, http://www.who.int/leishmaniasis/collaborating_centres/en/
[1]Additional WHO leishmaniasis laboratories are listed in WHO Technical Report Series 949 "Control of the leishmaniasis" pages 162–3 [42].
[2]Recommend contact with reference laboratories in advance for instructions to optimize specimen collection and shipping. Tests performed in the above laboratories are provided free of charge.

with this management [Strong, low; E.C. dissents, recommending that all persons with NWCL receive treatment]. *Remark: See XXIV and XXV regarding the management of CL in immunocompromised persons*.

26. We suggest that systemic treatment be offered for persons even with healing/recently healed CL lesions caused by increased ML-risk species or when the species is unknown but the infection was acquired in an increased ML-risk region. Risks and benefits of such treatment should be discussed with the patient [Weak, low]. *Remark: In some cases, watchful waiting, with vigilance for signs and symptoms of ML, may be a reasonable approach.*
27. We recommend that any decision to observe a patient with CL without treatment should be reevaluated periodically, and the decision not to treat should be reconsidered if healing does not progress as anticipated [Strong, very low].
28. In all cases of CL, wound care, individualized documentation of lesion evolution, and patient education regarding the manifestations and detection of local therapeutic failure/relapse and ML should be routine components of management (see III and XV) [Strong, low].

## XI. In a person with CL, what could be the consequences of no treatment or suboptimal therapy, and how should persons who received no or suboptimal therapy be monitored?

*Recommendations*

29. Potential consequences of inadequate treatment include poor cosmetic outcome due to scarring or superinfection, the persistence of a chronic wound(s), and, with some *Leishmania* species, destructive and disfiguring

ML. In immunocompromised persons, cutaneous, mucosal, and visceral dissemination may occur [FACT, no grade].

30. Persons with CL should be actively monitored by clinical appearance, including by performing a careful nasal and oropharyngeal examination periodically up to 1 year, or at least 2 years if at increased risk for ML. They should be educated about the signs and symptoms of relapse and ML and instructed to seek medical attention anytime these appear [Strong, low].
31. Symptoms such as chronic nasal stuffiness, epistaxis, or hoarseness or findings such as septal perforation that occur anytime in a person with a prior or current diagnosis of CL or a scar consistent with prior CL should prompt evaluation for ML, including fiberoptic examination of the affected area if relevant (see II and III) [Strong, moderate].

**XII. In a person with CL, what factors should prompt consideration of use of a systemic (oral or parenteral) agent for initial therapy?**

*Recommendations*

32. Systemic treatment is recommended for persons with complex CL as defined in Table 1 [Strong, moderate].
33. Initial systemic therapy (see XIII) may be used in persons with CL in whom it is not practical to use local therapy or (possibly) if more rapid healing of large, cosmetically or functionally concerning lesions is preferred [Weak, very low].
34. Less common cutaneous syndromes, such as leishmaniasis recidivans (caused by *L. tropica* and occasionally other species), diffuse cutaneous leishmaniasis (caused by *L. mexicana, L. amazonensis*, and *L. aethiopica*), and disseminated cutaneous leishmaniasis (caused by *L.* [*V.*] *braziliensis*), usually require systemic therapy [Strong, low].

**XIII. What systemic treatment options are available in North America for CL, and what factors should be considered when selecting a medication for an individual patient?**

*Recommendations*

35. The parenteral options for systemic therapy currently available in North America include **conventional amphotericin B deoxycholate, lipid formulations of amphotericin B, pentavalent antimonial ($Sb^V$) compounds, and pentamidine (listed in alphabetical order). Oral options include miltefosine and the "azole" antifungal compounds, including ketoconazole (if potential benefits outweigh risks for hepatotoxicity and QT prolongation) and fluconazole [FACT, no grade].**
36. To maximize effectiveness and to minimize toxicity, the choice of agent, dose, and duration of therapy should be individualized [Strong, moderate]. *Remarks*: *No ideal or universally applicable therapy for CL has been identified. Some therapies/regimens appear highly effective only against certain Leishmania species/strains in certain areas of the world. Both the parasite species and host factors (e.g., comorbid conditions and immunologic status) should be considered.*
37. Factors that should be considered when selecting CL treatment for an individual patient include the risk for ML; the *Leishmania* strain/species and published response rates for antileishmanial agents in the pertinent geographic region; the potential for adverse events; age extremes; childbearing competence and pregnancy; obesity; hepatic, pancreatic, renal, and cardiac comorbid conditions; preference for and convenience of various routes of administration; the rapidity with which one wishes to control the infection; the impact of lesions on daily activities and patient self-confidence; the patient/provider comfort level with logistics (e.g., Investigational New Drug protocols); and other practical issues (e.g., drug availability, various types of cost, insurance reimbursement) (see XII and XXVI; Tables 3 and 4) [Strong, low].

**XIV. In which clinical settings can local therapy be used effectively in a person with CL?**

*Recommendations*

38. Local therapy is preferred for treatment of OWCL lesions defined as clinically simple (Table 1) and may be useful for localized NWCL caused by *Leishmania* species not associated with increased risk for ML [Strong, moderate]. *Remark: Local therapy includes heat and cryotherapy, topical ointments/creams with paromomycin and other ingredients, intralesional injections of pentavalent antimonial drugs (±cryotherapy), and photodynamic or laser treatment.*
39. Eschar(s) overlying ulcers should be debrided before administration of local therapy and any secondary infection managed to maximize treatment effect [Strong, very low].

**XV. What are the recommended timeframes and findings to assess response to treatment in a person with CL?**

*Recommendations*

40. Response to treatment is assessed by clinical criteria; repeat parasitologic testing is not recommended if the skin lesion appears to be healing [Strong, low]. *Remark: The healing process may continue after the treatment course is completed especially for large ulcerative lesions.*
41. Persons with CL should have their skin lesions monitored for 6–12 months after treatment for clinical evidence of therapeutic failure, which is initially seen at the border of a healed lesion [Strong, low]. *Remark: The first sign of healing is usually flattening of the skin lesion. By 4–6 weeks after treatment, the lesion size should have decreased by >50%, ulcerative lesions should be reepithelializing, and no new lesions should be appearing. Ulcerative lesions are generally fully reepithelialized and clinically healed by approximately 3 months after treatment.*

**XVI. What are the recommended approaches for additional management in a person with CL that does not respond to therapy?**

*Recommendations*

42. Additional therapy is recommended (but not necessarily always with a different agent or approach) when there is development of new skin lesions or worsening of existing lesions. Additional therapy is also recommended if there is incomplete healing by 3 months after completion of the treatment course [Strong, low].
43. We recommend that therapeutic failure be assessed by physical appearance. Relatively little improvement

**Table 3: Approach to Syndromic Treatment of Leishmaniasis in North America[1,2]**

| Syndrome | Treatment Classification | Drug/treatment | Proprietary Name | Source | Route of Administration | Regimen | FDA Approval and Availability | Comments |
|---|---|---|---|---|---|---|---|---|
| **Cutaneous** leishmaniasis (CL) | | | | | | | | |
| | Treatment of choice | There is no generally applicable treatment of choice; choice should be individualized. | | | | | | For cases of CL associated with increased risk for ML,[3] the choices include miltefosine, amphotericin B formulations, and pentavalent antimonials. |
| | Parenteral alternatives | | | | | | | |
| CL | | **Amphotericin B deoxycholate** | Fungizone® | Bristol Myers Squibb | IV | 0.5–1.0 mg/kg per dose daily or every other day for cumulative total of ~15–30 mg/kg | Yes, but not for CL; off-label use | |
| | | **Pentavalent antimonials**[4] | | | | | | In some settings, treatment for as few as 10 days has been effective. |
| CL | | Sodium stibogluconate | Pentostam® | GlaxoSmithKline, via CDC Drug Service or USAMMDA for military health care beneficiaries<br>via Special Access Program in Canada | IV, IM (IV preferred in North America[1]) | 20 mg $Sb^V$/kg/day for 20 days | No; but available in the US under a CDC-sponsored IND protocol. For military health care beneficiaries, contact Force Health Protection Division, US Army Medical Materiel Development Activity (USAMMDA).[13]<br>In Canada, via Special Access Program | Supplied as 100 mg $Sb^V$/mL. Dilute dose in D5W (~50–100 mL) for IV, ~10–30-minute infusion. Use of an in-line filter is recommended. |

*(continued)*

**Table 3**
Continued

| Syndrome | Treatment Classification | Drug/treatment | Proprietary Name | Source | Route of Administration | Regimen | FDA Approval and Availability | Comments |
|---|---|---|---|---|---|---|---|---|
| CL | | Meglumine antimoniate | Glucantime® | Sanofi<br>via Special Access Program in Canada | IV, IM (IV preferred in North America[1]) | As per Pentostam® | No; in US, would require investigator-sponsored IND protocol.<br>In Canada, via Special Access Program | Supplied as 81 mg $Sb^{V}$/mL. Dilute dose in D5W (~50–100 mL) for IV, ~10–30-minute infusion. |
| CL | | **Liposomal** amphotericin B | AmBisome® | Astellas | IV | 3 mg/kg/day on days 1–5 and 10 or on days 1–7 (total 18–21 mg/kg) | Yes, but not for CL; off-label use | No standard dosage regimens have been established; other regimens have been described in case reports/series from various settings. |
| CL | | **Pentamidine** isethionate | Pentam 300® | APP Pharmaceuticals | IV, IM (IV preferred in North America[1]) | 3–4 mg/kg every other day for 3 or 4 doses | Yes, but not for CL; off-label use | *L.* (*V.*) *panamensis*/ guyanensis: an alternative regimen is 2 mg/kg every other day for 7 doses. |
| | Oral alternatives | | | | | | | |
| | | **Azoles** | | | | | | |
| CL | | Fluconazole | Diflucan® | Pfizer | po | **Adults**: 200 mg daily for 6 weeks | Yes, but not for CL; off-label use | See XIII regarding preliminary data for therapy with higher daily doses. |
| CL | | Ketoconazole | Nizoral® | Janssen | po | **Adults**: 600 mg daily for 28 days | Yes, but not for CL; off-label use | Take with acidic drink (eg, coke or citric juice). |
| CL | | **Miltefosine** | Impavido® | In US: Knight Therapeutics, via Profounda, the US marketer.<br>In Canada: via Special Access Program | po | **FDA-approved regimen**: if 30–44 kg, 50 mg bid for 28 days; if ≥45 kg, 50 mg tid for 28 days | Yes, for CL caused by *Viannia* species; off-label use for other species | Target dose is ~2.5 mg/kg/day, but doses >150 mg/day have not been studied. GI side effects may limit higher doses. See Table 4 and XXVI. |

*(continued)*

**Table 3**
Continued

| Syndrome | Treatment Classification | Drug/treatment | Proprietary Name | Source | Route of Administration | Regimen | FDA Approval and Availability | Comments |
|---|---|---|---|---|---|---|---|---|
| | Intralesional alternatives | | | | | | | |
| | | **Pentavalent** antimonials[4] | | | | | | |
| CL | | Sodium stibogluconate | Pentostam® | GlaxoSmithKline, via CDC Drug Service or USAMMDA for military health care beneficiaries[13] | IL | Various regimens, eg, 0.2–5 mL per session every 3–7 days (or up to every 3 weeks) +/− cryotherapy for 5–8 sessions or until healing. 5 sites/lesion with a 25–27G needle intradermally for 0.1 mL/$cm^2$ until blanched. | Not currently covered by the CDC-sponsored IND protocol | Use undiluted drug. Consider premedication (eg, with EMLA: lidocaine/prilocaine). In children, sedation/anesthesia may be required. Avoid body sites as per heat therapy (see below). |
| CL | | Meglumine antimoniate | Glucantime® | Sanofi<br>via Special Access Program in Canada | IL | As per Pentostam® | No; in US, would require investigator-sponsored IND protocol.<br>In Canada, via Special Access Program | |
| | Topical alternatives | | | | | | | |
| | | **Paromomycin preparations** | | | | | | |
| CL | | 15% paromomycin and 12% MBCL ointment | Leshcutan® | Approximate with compounding pharmacy | Topical | Apply bid for 10 days, rest for 10 days, and reapply bid for 10 days | The capsule formulation of paromomycin is FDA approved for other indications; use of the capsules to compound antileishmanial ointment constitutes off-label use. | Local irritation (from MBCL) may lead some patients to discontinue therapy. Higher response rates noted for infection caused by *L. major* than *L. tropica*. |

*(continued)*

**Table 3**
Continued

| Syndrome | Treatment Classification | Drug/treatment | Proprietary Name | Source | Route of Administration | Regimen | FDA Approval and Availability | Comments |
|---|---|---|---|---|---|---|---|---|
| CL | | 15% paromomycin and 0.5% gentamicin cream | WR 279,396 | Expanded-access IND protocol; otherwise, approximate with compounding pharmacy | Topical | Apply once per day for 20 days | See above about paromomycin capsules. Treatment under expanded-access IND protocol currently is limited to US military health care beneficiaries. | Local erythema and/or mild pain are commonly noted. See section XIV for some similar drug compounding instructions and the US military point of contact. |
| CL | | **Heat therapy** | ThermoMed™ | TTI, Thermosurgery Technologies, Inc. | Locally applied to skin | Apply under local anesthesia for 30-sec doses in grid-like pattern extending 1–2 mm into surrounding normal-appearing skin. Usually one session (sometimes up to 3). | Yes, cleared for CL indication | Avoid applying over eyelids, tip of nose, lips, mucus membranes, cartilaginous structures, or superficial nerves. Use topical antibiotics for several days after the heat treatment. Keloids may be less common than with cryotherapy. |
| CL | | **Cryotherapy with liquid nitrogen** | | No special applicator required | Locally applied to skin | Multiple regimens, eg, freeze 15–20 sec until 1–2 mm of normal circumferential skin frozen, thaw 20–60 sec, and freeze again. Repeat every 3 weeks for up to 3 total applications (fewer, if healed sooner). | Yes, "grandfathered in" | Increased efficacy has been noted if used in combination with IL $Sb^V$. Avoid applying over eyelids, tip of nose, lips, mucus membranes, cartilaginous structures, or superficial nerves. |

*(continued)*

**Table 3**
Continued

| Syndrome | Treatment Classification | Drug/treatment | Proprietary Name | Source | Route of Administration | Regimen | FDA Approval and Availability | Comments |
|---|---|---|---|---|---|---|---|---|
| **Mucosal leishmaniasis (ML)** | | | | | | | | |
| | Treatment of choice | There is no treatment of choice; choice should be individualized. | | | | | | |
| | Alternatives | | | | | | | |
| ML | | **Amphotericin B deoxycholate** | Fungizone® | Bristol Myers Squibb | IV | 0.5–1.0 mg/kg per dose daily or every other day for cumulative total of ~20–45 mg/kg | Yes, but not for ML; off-label use | |
| ML | | **Liposomal amphotericin B** | AmBisome® | Astellas | IV | ~3 mg/kg/day for cumulative total of ~20–60 mg/kg | Yes, but not for ML; off-label use | |
| ML | | **Miltefosine** | Impavido® | In US: Knight Therapeutics, via Profounda, the US marketer.<br>In Canada: via Special Access Program | po | **FDA-approved regimen**: if 30–44 kg, 50 mg bid for 28 days; if ≥45 kg, 50 mg tid for 28 days | Yes, approved for ML caused by *L.* (*V.*) *braziliensis* | Target dose is ~2.5 mg/kg/day, but doses >150 mg/day have not been studied. GI side effects may limit higher doses. See Table 4 and XXVI. |
| | - | **Pentavalent antimonials**[4] | | | | | | |
| ML | | Sodium stibogluconate | Pentostam® | GlaxoSmithKline, via CDC Drug Service or USAMMDA for military health care beneficiaries[13] | IV, IM (IV preferred in North America[1]) | 20 mg $Sb^V$/kg/day for 28 days | No; but available in the US under a CDC-sponsored IND protocol. For military health care beneficiaries, available from USAMMDA.[13] | Supplied as 100 mg $Sb^V$/mL. Dilute dose in D5W (~50–100 mL) for IV, ~10–30-minute infusion. Use of an in-line filter is recommended. |
| ML | | Meglumine antimoniate | Glucantime® | Sanofi<br>via Special Access Program in Canada | IV, IM (IV preferred in North America[1]) | As per Pentostam® | No; in US, would require investigator-sponsored IND protocol.<br>In Canada, via Special Access Program | Supplied as 81 mg $Sb^V$/mL. Dilute dose in D5W (~50–100 mL) for IV, ~10–30-minute infusion. |

*(continued)*

**Table 3**
Continued

| Syndrome | Treatment Classification | Drug/treatment | Proprietary Name | Source | Route of Administration | Regimen | FDA Approval and Availability | Comments |
|---|---|---|---|---|---|---|---|---|
| ML | Lesser alternative | **Pentamidine isethionate** | Pentam 300® | APP Pharmaceuticals | IV, IM (IV preferred in North America[1]) | 2–4 mg/kg every other day or 3 times per week for 15 or more doses | Yes, but not for ML; off-label use | |
| **Visceral leishmaniasis (VL)**[5] | | | | | | | | |
| VL | Treatment of choice | **Liposomal amphotericin B**[6] | AmBisome® | Astellas | IV | **FDA-approved regimen**, if **immunocompetent**[5,7]: 3 mg/kg/day on days 1–5, 14, and 21 (total dose 21 mg/kg)<br>**FDA-approved regimen**, if **immunosuppressed**[8]: 4 mg/kg/day on days 1–5, 10, 17, 24, 31, and 38 (total dose 40 mg/kg) | Yes, for this indication | See XIX regarding other regimens that have been used in various settings. For treatment of VL in immunocompetent[7] persons with VL acquired in East Africa, regimens with total doses ≥40 mg/kg may be needed. |
| | Alternatives[9] | | | | | | | |
| VL | | **Miltefosine**[10] | Impavido® | In US: Knight Therapeutics, via Profounda, the US marketer.<br>In Canada: via Special Access Program | po | **FDA-approved regimen**: if 30–44 kg, 50 mg bid for 28 days; if ≥45 kg, 50 mg tid for 28 days[8] | Yes, for VL caused by *L. donovani* | On the basis of anecdotal experience in Europe and Brazil, not as effective for VL caused by *L. infantum-chagasi*. In general, target dose is ~2.5 mg/kg/day, but doses >150 mg/day have not been studied. GI side effects may limit higher doses. See Table 4 and XXVI. |

*(continued,*

**Table 3**
Continued

| Syndrome | Treatment Classification | Drug/treatment | Proprietary Name | Source | Route of Administration | Regimen | FDA Approval and Availability | Comments |
|---|---|---|---|---|---|---|---|---|
| | - | **Pentavalent antimonials[4,11]** | | | | | | |
| VL | | Sodium stibogluconate | Pentostam® | GlaxoSmithKline, via CDC Drug Service or USAMMDA for military health care beneficiaries[13] | IV, IM (IV preferred in North America[1]) | 20 mg $Sb^V$/kg/day for 28 days[8] | No; but available in the US under a CDC-sponsored IND protocol. For military health care beneficiaries, available from USAMMDA.[13] | Supplied as 100 mg $Sb^V$/mL. Dilute dose in D5W (~50–100 mL) for IV, ~10–30-minute infusion. Use of an in-line filter is recommended. |
| | | | | via Special Access Program in Canada | | | In Canada, via Special Access Program | |
| VL | | Meglumine antimoniate | Glucantime® | Sanofi | IV, IM (IV preferred in North America[1]) | As per Pentostam® | No; in US, would require investigator-sponsored IND protocol. In Canada, via Special Access Program | Supplied as 81 mg $Sb^V$/mL. Dilute dose in D5W (~50–100 mL) for IV, ~10–30-minute infusion. |
| | | | | via Special Access Program in Canada | | | | |
| VL | | **Amphotericin B deoxycholate[6]** | Fungizone® | Bristol Myers Squibb | IV | 1 mg/kg per dose daily or every other day for a total of 15–20 doses[8] | Yes, but not for VL; off-label use | |

*(continued)*

**Table 3**
Continued

| Syndrome | Treatment Classification | Drug/treatment | Proprietary Name | Source | Route of Administration | Regimen | FDA Approval and Availability | Comments |
|---|---|---|---|---|---|---|---|---|
| VL | | **Amphotericin B lipid complex** | Abelcet® | Sigma-Tau Pharmaceuticals | IV | **Immunocompetent**[5,7]: 2–3 mg/kg/day for 5–10 days<br>**Immunosuppressed**[8]: 3–5 mg/kg/day for 10 days[12] | Yes, but not for VL; off-label use | Liposomal amphotericin B (L-AmB) is the treatment of choice for VL. Bioequivalence between amphotericin B lipid complex (ABLC) and L-AmB for treatment of VL has not been established; ABCL has been less well studied in VL treatment trials and, anecdotally, may not be as effective as AmBisome® (rough conversion: 3 mg/kg of liposomal amphotericin B is about 5 mg/kg of ABLC). |
| VL | Lesser alternative | **Pentamidine isethionate** | Pentam 300® | APP Pharmaceuticals | IV, IM (IV preferred in North America[1]) | 4 mg/kg every other day or three times per week for ~15–30 doses[8] | Yes, but not for VL; off-label use | Considered second-line therapy because of toxicity (see Table 4) and lower efficacy. |

[1] For simplicity, the terminology North America is used to refer to the United States and Canada.
[2] All treatment-related decisions should be individualized. The lists of treatment approaches/drugs and regimens are not all inclusive. For the listed systemic drugs, see Table 4 regarding adverse events, monitoring for toxicity, and mitigation approaches. See XXIII-XXV regarding treatment considerations applicable to HIV-coinfected persons and to persons who are immunocompromised for other reasons. See XXVI for considerations for other special populations of patients (eg, young children).
[3] See Table 1 and X-XIII for additional perspective.
[4] The pentavalent antimonial drugs—sodium stibogluconate (Pentostam®) and meglumine antimoniate (Glucantime®)—are considered comparable when dosed on the basis of $Sb^V$ content. In general, the daily dose does not have an upper limit in mgs (ie, the daily dose no longer is limited to 850 mg); however, see XXVI for additional perspective and cautionary notes.
[5] Persons newly diagnosed with VL should be assessed for concurrent HIV/AIDS or other causes of cell-mediated immunosuppression.
[6] Liposomal amphotericin is approved by the U.S. Food and Drug Administration for the treatment of VL. The off-label use of amphotericin B deoxycholate is likely to be effective but is generally more toxic (see Table 4).
[7] An immunocompetent person is defined as someone without an identified congenital or acquired immune defect (eg, HIV/AIDS). In general, *L. donovani* (India) may be treated with a shorter course of ABLC, whereas *L. infantum* in Europe requires 10 days duration [300, 377].
[8] See XXIII regarding secondary prophylaxis in patients with HIV/AIDS-associated VL. Chronic maintenance therapy (secondary prophylaxis) should be given until the CD4 T-lymphocyte cell count consistently remains >200–350/$mm^3$ (see XXIII).
[9] See XIX and XX for additional perspective about treatment alternatives. Parenteral paromomycin appeared promising in clinical trials in India, but it is not available in North America.
[10] Miltefosine has been effective in treating VL in India and adjacent areas of South Asia where resistance to pentavalent antimonials is prevalent. There is some evidence to support the use of miltefosine for VL acquired in East Africa. There is less available evidence to support its use in southern Europe and Latin America.
[11] Resistance to pentavalent antimonials is well documented in India and has been reported from other areas. In general, pentavalent antimonial therapy should not be used for persons who acquired VL in India.
[12] Personal communication Pierre Buffet, on the basis of expert opinion.
[13] Contact information for use in military beneficiaries:
Force Health Protection Division's 24-hour cell phone: 301-401-2768
Force Health Protection Division's email: usarmy.detrick.medcom-usammda.mbx.force-health-protection@mail.mil
CDC Drug Service (telephone: 404-639-3670; email: drugservice@cdc.gov)
Canada's Special Access Program

**List of Abbreviations:** CDC (Centers for Disease Control and Prevention), CL (cutaneous leishmaniasis), D5W (5% dextrose in water), EMLA (lidocaine and prilocaine topical anesthetic), FDA (Food and Drug Administration), GI (gastrointestinal), IL (intralesional), IM (intramuscular), IND (Investigational New Drug), IV (intravenous), kg (kilogram), MBCL (methylbenzethonium chloride), mg (milligram), mL (milliliter), ML (mucosal leishmaniasis), mm (millimeter), po (by mouth), $Sb^V$ (pentavalent antimony), sec (second(s), US (United States), VL (visceral leishmaniasis).

**Table 4**: Drugs Used in North America for Systemic[a] Antileishmanial Therapy: Adverse Events, Monitoring for Toxicity, and Mitigation Approaches[b]

| Drug[c] | Route(s) of Administration | Adverse Events[d,e] | Laboratory Monitoring for Toxicity[d,f] | Mitigation and Management Approaches[d,f] | Pregnant Patients[f,g] | Breastfeeding Patients[f,h] | Comments |
|---|---|---|---|---|---|---|---|
| Parenteral | | | | | | | |
| Amphotericin B formulations | | | | | | | |
| Amphotericin B deoxycholate | IV | Infusion-related reactions[i] (eg, fever, rigors, headache, nausea, vomiting, hypotension, tachypnea), electrolyte abnormalities (eg, hypokalemia, hypomagnesemia), nephrotoxicity, anemia | Baseline and frequent (eg, once or twice weekly) serum chemistry values and CBC. More frequent and/or additional testing (eg, ECG, urinalysis) may be indicated or prudent for some patients. | Examples: premedication; saline loading; test dose; slow infusions (~2–6 h); electrolyte supplementation, increased intervals between doses, and/or drug holidays, if indicated. Avoid/ minimize use of other nephrotoxic agents (eg, nonsteroidal anti-inflammatory drugs). | FDA pregnancy category B[j] | Probably compatible (see text XXVI); interruption of breastfeeding may be prudent. | |
| Liposomal amphotericin B (*also other lipid-associated formulations of amphotericin B*) | IV | Usually better tolerated than amphotericin B deoxycholate but similar types of toxicity (eg, renal). Infusion-related reactions to liposomal amphotericin B also can be caused by liposome-induced complement activation-related pseudoallergy (CARPA; see text XIX and XX). | See above. | See above (eg, minimize use of other nephrotoxic agents) but modify as appropriate (eg, liposomal amphotericin B typically is infused over ~2 h; minimum of ~1 h). | FDA pregnancy category B[j] (see text XXVI) | See above. | |

*(continued)*

**Table 4**
Continued

| Drug[c] | Route(s) of Administration | Adverse Events[d,e] | Laboratory Monitoring for Toxicity[d,f] | Mitigation and Management Approaches[d,f] | Pregnant Patients[f,g] | Breastfeeding Patients[f,h] | Comments |
|---|---|---|---|---|---|---|---|
| Pentavalent antimonial ($Sb^V$) compounds—sodium stibogluconate (Pentostam®) and meglumine antimoniate (Glucantime®) | IV, IM[a] | Various symptoms (eg, myalgia, large-joint arthralgia, headache, malaise, fatigue, anorexia, nausea) commonly noted as treatment course progresses. Laboratory abnormalities usually reversible (during or after treatment), including elevated aminotransferase, lipase, and amylase values (see comments); also, ECG abnormalities (eg, nonspecific ST-T-wave changes; less often, clinically relevant QTc prolongation) and cytopenias (in VL, pretreatment cytopenias typically improve during therapy). | Baseline and weekly serum chemistry values (eg, aminotransferases, lipase/amylase, potassium, creatinine, BUN, glucose), CBC, and ECG. More frequent monitoring may be indicated or prudent for some patients (see text XXVI). | Avoid/minimize use of other agents (eg, drugs linked to QTc prolongation). Interrupt $Sb^V$ therapy if QTc prolongation (eg, if QTc >0.50 sec), concave ST-segments, clinically relevant arrhythmias, or moderate-to-severe clinical pancreatitis; thresholds for interrupting therapy if asymptomatic laboratory abnormalities (eg, elevated aminotransferase levels) should be individualized. Nonsteroidal anti-inflammatory drugs may be used for symptomatic therapy; avoid rigorous physical activity. | Not formally assigned to an FDA pregnancy category (see text XXVI) | Probably compatible (see text XXVI); interruption of breastfeeding may be prudent. | Patients with advanced immunosuppression (eg, AIDS) may have life-threatening pancreatitis or cardiotoxicity (see text XXIII). See XXVI regarding considerations for other special populations (eg, children). |

*(continued)*

**Table 4**
Continued

| Drug[c] | Route(s) of Administration | Adverse Events[d,e] | Laboratory Monitoring for Toxicity[d,f] | Mitigation and Management Approaches[d,f] | Pregnant Patients[f,g] | Breastfeeding Patients[f,h] | Comments |
|---|---|---|---|---|---|---|---|
| Pentamidine isethionate | IV, IM | Various symptoms (eg, nausea, vomiting, dysgeusia, headache); hypo/hyperglycemia, insulin-dependent diabetes mellitus (may e diagnosed up to several months posttreatment), pancreatitis, hypotension, QTc prolongation, nephrotoxicity, hyperkalemia, hypocalcemia, hepatoxicity, cytopenias (leukopenia/ thrombocytopenia > anemia). If IM: also pain and sterile abscesses at injection sites; rhabdomyolysis. | Assess before, during, and after therapy: serum chemistry values, CBC, and ECG. Monitor fasting \glucose level (and urinalysis) before each dose and ~3 weeks and ~2–3 months posttreatment. If indicated (if potential for rhabdomyolysis), check or monitor CPK level. | To minimize risk for hypotension, infuse drug over 1–2 h; keep patient supine; check vital signs before, during, and after infusion (or injection) until stable. Avoid/ minimize use of other agents, including nephrotoxic drugs. | Typically, not warranted or recommended for antileishmanial treatment during pregnancy | Selection of a different drug or interruption of breastfeeding may be prudent. | |
| Oral | | | | | | | |
| Azoles | Oral | GI symptoms (eg, nausea, vomiting, abdominal pain); headache; hepatotoxicity | Baseline and weekly assessment of hepatic function (eg, aminotransferase levels). More frequent and/or additional types of monitoring (eg, ECG, CBC) may be indicated or prudent for some patients. | Avoid/minimize use of other hepatotoxic agents (eg, acetaminophen). Hepatoxicity may warrant interrupting therapy. Both drugs listed below are associated with drug interactions that can be life threatening. | Typically, not warranted or recommended for antileishmanial treatment during pregnancy | | |
| Fluconazole | Oral | See above. Also: reversible hair loss and agranulocytosis | See above. | Can be taken with or without food. (Also see above.) | See above. | Generally considered compatible; on principle, interruption of breastfeeding may be prudent. | |

*(continued)*

**Table 4**
Continued

| Drug[c] | Route(s) of Administration | Adverse Events[d,e] | Laboratory Monitoring for Toxicity[d,f] | Mitigation and Management Approaches[d,f] | Pregnant Patients[f,g] | Breastfeeding Patients[f,h] | Comments |
|---|---|---|---|---|---|---|---|
| Ketoconazole[k] | Oral | See above. Risk for severe hepatoxicity (fatal or requiring transplantation) may be higher than with other azoles and may occur regardless of dose/ duration of therapy.[k] QTc prolongation may occur and lead to life-threatening ventricular arrhythmias.[k] High-dose therapy may be associated with decreased secretion of adrenal corticosteroids and/or reversible decreases in serum testosterone levels. | See above. | To minimize GI symptoms, take with food; gastric acidity required. (Also see above.) Avoid use of other drugs linked to QTc prolongation, including drugs metabolized by CYP3A4.[k] | See above. | Selection of a different drug or interruption of breastfeeding may be prudent. | |
| Miltefosine[k] | Oral | GI symptoms (nausea/ vomiting > diarrhea), mainly early in treatment course; dizziness/motion sickness; scrotal pain (decreased/absent ejaculate); nephrotoxicity and/or hepatoxicity | Baseline and weekly assessment of renal function; also (particularly, if VL) monitor hepatic function (aminotransferase and bilirubin levels) and CBC (platelet count). | To minimize GI symptoms, take with food and use divided daily dosing (see text XXVI). Encourage fluid intake if vomiting/diarrhea. | Female patients with reproductive potential[k] should have a negative pretreatment pregnancy test, should use effective contraception during and for 5 months after treatment, and should not rely on hormonal contraception if vomiting/diarrhea. | Breastfeeding not recommended during or for 5 months after treatment (see text XXVI). | Not FDA-approved for patients <12 years of age or <30 kg. See text (XXVI) regarding considerations for children and other special populations. Contraindicated in patients with Sjögren-Larsson Syndrome (congenital ichthyosis). |

**Abbreviations**: BUN, blood urea nitrogen; CBC, complete blood count; CPK, creatine phosphokinase; ECG, electrocardiogram; FDA, U.S. Food and Drug Administration; GI, gastrointestinal; IM, intramuscular; IV, intravenous; QTc, corrected QT interval (on ECG); $Sb^V$, pentavalent antimony (antimonial); VL, visceral leishmaniasis.
[a]See Table 3 and text (eg, XIV and XXVI) regarding nonsystemic drug therapies, including treatment with intralesional $Sb^V$ and topical paromomycin.
[b]To help ensure safe and effective therapy, see full prescribing information for additional details, including potential drug interactions. Expert consultation also is encouraged regarding such issues as whether to start, continue, or interrupt therapy with a particular antileishmanial agent; to adjust the dosage regimen; or to select a different agent if the patient has or develops laboratory abnormalities or comorbid conditions. On principle, minimize the use of other medications/supplements and avoid alcohol.
[c]In general, drugs are listed alphabetically in the parenteral and oral categories and in the subcategories (eg, azoles); however, “pentavalent antimonial compounds” are listed before “pentamidine isethionate.”
[d]Not all-inclusive.
[e]Selected examples are provided (eg, comparatively common or noteworthy adverse events); potential dermatologic effects and phlebitis (if IV) are not addressed. In general, symptoms are listed first. The types and rates of adverse events associated with a particular drug may vary, depending on interrelated factors such as the leishmanial syndrome, dosage regimen, and host characteristics (eg, immunologic status, comorbid conditions, concomitant/recent use of other medications).
[f]Should be individualized.
[g]Use during pregnancy only if clearly indicated (see text XXVI); expert consultation encouraged.
[h]The potential for risk to breastfeeding infants cannot be excluded; expert consultation encouraged.
[i]Some of the examples (eg, headache, GI symptoms) are not necessarily just infusion related.
[j]Reproduction studies in animals have not demonstrated fetal risk; however, data from adequate, controlled studies in pregnant women are not available.
[k]See boxed warning (also known as “black box warning”) in prescribing information.

or worsening while on therapy suggests an inadequate response and an alternate treatment approach should be planned [Strong, low]. *Remark: A paradoxical increase in the local inflammatory response may be seen in the first 2–3 weeks of treatment and can be difficult to differentiate from therapeutic failure.*

44. Consultation with a leishmaniasis expert about other treatment options is recommended for management of persons' lesions associated with therapeutic failure [Strong, very low].

## MUCOSAL LEISHMANIASIS

### XVII. What are the treatment options for American (New World) mucosal leishmaniasis (ML)?

*Recommendations*

45. All persons with clinically manifest, metastatic, American ML should receive systemic antileishmanial therapy, with the goals of preventing morbidity (e.g., disfigurement) and mortality (e.g., from aspiration pneumonia or respiratory obstruction) [Strong, low].
46. Before treatment is initiated, a complete examination of the naso-oropharyngeal/laryngeal mucosa should be conducted by a specialist to assess the anatomic extension and clinical severity of the mucosal disease, which have prognostic implications [Strong, moderate].
47. We recommend inpatient monitoring and prophylactic corticosteroid therapy for persons with laryngeal/pharyngeal disease and increased risk for respiratory obstruction, as indicated by symptoms and otolaryngologic/radiologic examinations, because of the potential for inflammatory reactions after initiation of antileishmanial therapy [Strong, low].
48. The choice of antileishmanial agent, dose, and duration of therapy for persons with ML should be individualized (Table 3) [Strong, moderate]. *Remarks: The traditional options for ML include treatment with a pentavalent antimonial ($Sb^V$) compound (20 mg $Sb^V$/kg daily, IV or IM, for 28–30 days) or with amphotericin B deoxycholate (0.5–1.0 mg/kg per dose, IV, daily or every other day, for a cumulative total of ~20–45 mg/kg). More recently, on the basis of comparatively limited data, the armamentarium has expanded to include lipid formulations of amphotericin B (typically, liposomal amphotericin B [L-AmB], with a cumulative total dose ranging widely from ~20–60 mg/kg), as well as the oral agent miltefosine (~2.5 mg/kg per day [maximum, 150 mg/day] for 28 days).*

## VISCERAL LEISHMANIASIS

### XVIII. In what circumstances should a person with visceral *Leishmania* infection be treated?

*Recommendations*

49. We recommend that persons with clinical abnormalities compatible with VL and laboratory evidence of VL be treated (Table 3) [Strong, moderate].
50. We suggest that clinicians closely monitor persons with asymptomatic visceral infection and generally initiate therapy only if clinical manifestations of VL develop [Weak, very low].

### XIX. What is the optimal treatment for VL in a symptomatic immunocompetent person (person without an identified immune defect) in North America?

*Recommendations*

51. For an immunocompetent person with VL, treatment with liposomal amphotericin B (L-AmB) is recommended. The FDA-approved dosage regimen is 3 mg/kg/day IV on days 1–5, 14, and 21 (total dose, 21 mg/kg) (Table 3) [Strong, high]. *Remarks: Multiple regimens in which the total L-AmB dose is 18–21 mg/kg have been used effectively in regions other than East Africa. Doses of 40 mg/kg or more may be necessary in persons with VL acquired in East Africa. Other lipid-associated formulations of amphotericin B, such as amphotericin B lipid complex and amphotericin B colloidal dispersion, are not generally recommended: they have not been approved by FDA for treatment of VL; and they have been less well studied in VL treatment trials (i.e., bioequivalence has not been established).*
52. For an immunocompetent person with VL caused by *L. donovani*, acquired in the Indian subcontinent (South Asia), who is ≥12 years of age, weighs ≥30 kg, and is not pregnant or breastfeeding, treatment with the oral agent miltefosine, 2.5 mg/kg per day (maximum, 150 mg, in 3 divided doses) for 28 days, is a possible alternative to L-AmB, particularly in persons weighing <75 kg (see XXVI and Table 3) [Strong, moderate].

### XX. What alternative agent(s) can be used for a person with VL who cannot tolerate liposomal amphotericin B or miltefosine or in whom these agents otherwise are contraindicated?

*Recommendations*

53. Pentavalent antimonial therapy (20 mg $Sb^V$/kg/day IV or IM for 28 days) is a recommended therapy for immunocompetent persons with VL acquired in areas where the prevalence of antimony-resistant *Leishmania* species is low (<10%) [Strong, high].
54. We do not recommend switching to amphotericin B deoxycholate in persons with contraindications to, or substantial toxicity with, L-AmB, with the exception of persons who develop liposome-induced complement activation-related pseudoallergy (CARPA). Amphotericin B lipid complex is a consideration in this situation [Strong, low].

### XXI. In persons with VL, what parameters should be used to assess the clinical response to treatment?

*Recommendations*

55. Clinical parameters correlate well with parasitologic responses to VL treatment and should be used to monitor the response [Strong, low].
56. Parasitologic confirmation of response (such as by repeat bone marrow aspiration for microscopy and culture after treatment) is not recommended in a patient showing a timely clinical response. Antibody levels fall but over many months or longer [Strong, moderate].

**XXII. How should a person with VL be treated who does not respond to initial therapy as assessed by these parameters [or who has a relapse]?**

*Recommendations*

57. Immunocompetent persons with VL who do not respond to therapy with L-AmB should be treated with an alternative drug or with a higher dose or a longer course of L-AmB [Strong, low].
58. Immunocompetent persons with VL who do not respond to initial therapy with miltefosine or a pentavalent antimonial compound should be treated with L-AmB or an alternative drug if L-AmB is unavailable [Strong, low].
59. Immunocompetent persons with VL who respond to initial therapy but subsequently have a relapse should be treated with an alternative drug or with another, potentially longer, course of therapy with the initial drug. If L-AmB was the drug used for initial therapy, use of a higher dose can be considered [Strong, low].
60. Combination therapies may be considered but have not been well studied after therapeutic failure in persons with VL [Weak, low].

## LEISHMANIASIS IN IMMUNOCOMPROMISED HOSTS

**XXIII. How should HIV/AIDS-associated visceral leishmaniasis (VL) be treated in persons in North America, and what other management issues should be considered?**

*Recommendations*

61. Liposomal amphotericin B (L-AmB) is recommended for the treatment of VL in immunocompromised persons in North America (Table 3) [Strong, low]. *Remark: The FDA-approved dosage regimen of L-AmB for such persons, including those with concurrent HIV/AIDS, is 4 mg/kg/day IV, on days 1–5, 10, 17, 24, 31, and 38 (10 doses over a 38-day period), for a total dose of 40 mg/kg*.
62. Combination therapy (e.g., L-AmB plus miltefosine) might be considered, especially for persons with refractory cases of VL [Weak, very low]. *Remark: The efficacy and optimal duration of miltefosine monotherapy (and combination therapy) for HIV/AIDS-associated VL have not been established.*
63. Because of the importance of effective immune reconstitution in HIV-VL-coinfected persons, antiretroviral therapy (ART) should be initiated or optimized as soon as the person is sufficiently stable to tolerate it (e.g., either during or soon after the initial course of therapy for VL) [Strong, low].
64. *Leishmania* infection that becomes clinically manifest or worsens after initiation of ART should be treated with antileishmanial (and, if indicated, corticosteroid) therapy; leishmaniasis-associated immune reconstitution inflammatory syndrome (IRIS) reactions after initiation of ART have been reported occasionally [Strong, very low].
65. We recommend administering secondary prophylaxis (chronic maintenance therapy) to decrease the risk for posttreatment relapse of VL in persons with HIV/AIDS-associated immunosuppression (e.g., CD4 T-lymphocyte cell counts <200 cells/$mm^3$) [Strong, moderate].
66. Persons with VL-HIV/AIDS coinfection should be monitored indefinitely (until effective immune reconstitution) for evidence of posttreatment relapse; ART and secondary prophylaxis provide only partial protection against relapse. Antileishmanial treatment is indicated for persons who have clinical and parasitologic evidence of recurrence [Strong, low].

**XXIV. How should HIV/AIDS-associated CL or ML be treated in persons in North America who do not have evidence of VL, and what other management issues should be considered?**

*Recommendations*

67. In HIV/AIDS-associated CL/ML, systemic antileishmanial therapy is recommended, particularly in persons who are moderately to severely immunosuppressed (e.g., have CD4+ T-lymphocyte cell counts <200–350 cells/$mm^3$), who may be at increased risk for suboptimal therapeutic responses, for posttreatment relapses, and for cutaneous, mucosal, or visceral dissemination [Strong, very low].
68. The systemic regimens used for CL/ML in otherwise comparable immunocompetent persons typically are recommended for the initial treatment of coinfected persons, taking into account the potentials for drug interactions and toxicity (Tables 3 and 4) [Strong, very low]. *Remark: Whether coinfected persons who experience multiple posttreatment relapses of CL/ML would benefit from secondary prophylaxis (chronic maintenance therapy) has not yet been established*.
69. Antiretroviral therapy (ART) should be initiated or optimized in accordance with standard practice for HIV/AIDS; no evidence-based, CL/ML-specific recommendations regarding ART have been established [Strong, low].

**XXV. What is the preferred treatment of visceral/cutaneous leishmaniasis in immunocompromised hosts with solid organ transplant, persons with lymphatic-hematologic malignancies, or persons receiving immunosuppressive therapy for other reasons?**

*Recommendations*

70. Liposomal amphotericin B (L-AmB) is recommended as the drug of choice for immunosuppressed persons with VL (Table 3) [Strong, low]. *Remarks: The FDA-approved regimen is 4 mg/kg/day IV on days 1–5, 10, 17, 24, 31, and 38 (total dose of 40 mg/kg). Higher doses and longer durations of therapy may be needed depending in part on the level of immunosuppression.*
71. Doses of immunosuppressive drugs should be decreased in persons with VL during antileishmanial therapy whenever possible [Strong, very low].
72. Secondary prophylaxis is not recommended for initial management in persons with VL who have not manifested a relapse [Weak, low]. *Remark*: *Immunosuppressed persons with VL who are not coinfected with HIV typically have higher response rates to initial treatment and lower recurrence rates than HIV-coinfected persons.*
73. Routine serologic screening of organ donors from leishmaniasis-endemic areas is not recommended. If an available donor is known to be seropositive, it is advisable to perform clinical and laboratory monitoring

of the recipient in the post-transplant period rather than to reject the organ for transplant [Strong, low].

74. We suggest that clinicians not routinely perform diagnostic testing to assess persons for evidence of asymptomatic visceral infection, including persons who have lived or traveled in leishmaniasis-endemic regions (Figure 3) and are considering organ transplantation or initiation of therapy with biologic immunomodulating agents. Immunosuppressed persons known or found to be asymptomatically infected and those with a history of VL warrant close monitoring. Neither preemptive treatment nor primary prophylaxis for VL in asymptomatically infected persons is suggested [Weak, very low].
75. Immunosuppressed persons with VL who are not coinfected with HIV should be monitored for a minimum of 1 year (ideally lifelong or until effective immune reconstitution) to assess for posttreatment relapse. During clinical follow-up, assess for symptoms and, if present, pursue parasitologic confirmation of relapse [Strong, very low].
76. Confirmed VL therapeutic failure typically can be managed by retreatment using L-AmB at the same or a higher total dose [Strong, very low]. *Remark: Pentavalent antimonials could be used in some persons with VL under close follow-up.*
77. We suggest that CL/ML associated with the use of tumor necrosis factor (TNF)-alpha antagonist therapy be managed with systemic therapy and standard drug regimens for the pertinent setting/species (e.g., geographic area where the infection was acquired) [Weak, very low]. *Remark: Withdrawal of TNF-α antagonists during antileishmanial therapy may be appropriate: the risks, benefits, and feasibility of this action should be assessed on a case-by-case basis.*

## SPECIAL POPULATIONS AND LEISHMANIASIS

**XXVI. How should the treatment of leishmaniasis be modified in persons who are pregnant or lactating; are young children or older adults; or have comorbidities such as renal, hepatic, or cardiac dysfunction?**

*Recommendations*

78. In general, clinically manifest cases otf VL and ML should be treated even in these special populations of persons because the benefits of treatment typically outweigh the risks. However, patient-specific factors, including the presence of comorbid conditions, should be considered in the selection of the antileishmanial therapy, dosage regimen, and monitoring approach (Table 4) [Strong, low].
79. Decisions regarding whether and how to treat cases of CL in persons with special characteristics or comorbid conditions should take into account the potential risks and benefits of various approaches, such as initially observing without antileishmanial therapy, deferring treatment (e.g., until after pregnancy/delivery), and using local (vs systemic) therapy as the sole approach or as a temporizing measure, if otherwise appropriate and feasible [Strong, very low].

## INTRODUCTION

In the first section, the Panel summarizes background information relevant to the topic. In the second section, the Panel poses questions regarding the diagnosis and treatment of leishmaniasis, evaluates applicable clinical trial and observational data, and makes recommendations using the Grading of Recommendations, Assessment, Development and Evaluation (GRADE) framework [1]. The following 26 clinical questions were answered:

I. In a person with a compatible skin lesion(s) and exposure history, what specimen(s) should be collected for diagnostic testing of CL?
II. In a person with manifestations suggestive of New World mucosal leishmaniasis (ML), what types of specimens should be obtained for diagnostic testing?
III. During the initial and subsequent evaluations of persons with CL acquired in Central or South America who may have or be at risk for mucosal leishmaniasis (ML), what should be done to assess the possibility of mucosal disease?
IV. In a person with a compatible clinical course and epidemiologic context, what types of samples should be collected to evaluate for the diagnosis of VL?
V. What laboratory tests should be used to diagnose leishmaniasis?
VI. In a person with leishmaniasis, why could it be helpful to identify the infecting *Leishmania* species?
VII. What is the role of DNA-based assays in the diagnosis of leishmaniasis?
VIII. What is the role of serologic testing in the diagnosis of leishmaniasis?
IX. In a person with a consistent travel history and compatible skin lesion(s), is it necessary to obtain parasitologic confirmation of the diagnosis of leishmaniasis before starting treatment?
X. Is treatment of clinically manifest cutaneous infection (CL) always indicated?
XI. In a person with CL, what could be the consequences of no treatment or suboptimal therapy, and how should persons who received no or suboptimal therapy be monitored?
XII. In a person with CL, what factors should prompt consideration of use of a systemic (oral or parenteral) agent for initial therapy?
XIII. What systemic treatment options are available in North America for CL, and what factors should be considered when selecting a medication for an individual patient?
XIV. In which clinical settings can local therapy be used effectively in a person with CL?
XV. What are the recommended timeframes and findings to assess response to treatment in a person with CL?
XVI. What are the recommended approaches for additional management in a person with CL that does not respond to therapy?
XVII. What are the treatment options for American (New World) mucosal leishmaniasis (ML)?
XVIII. In what circumstances should a person with visceral *Leishmania* infection be treated?
XIX. What is the optimal treatment for VL in a symptomatic immunocompetent person (person without an identified immune defect) in North America?

XX. What alternative agent(s) can be used for a person with VL who cannot tolerate liposomal amphotericin B or miltefosine or in whom these agents otherwise are contraindicated?

XXI. In persons with VL, what parameters should be used to assess the clinical response to treatment?

XXII. How should a person with VL be treated who does not respond to initial therapy as assessed by these parameters [or who has a relapse]?

XXIII. How should HIV/AIDS-associated visceral leishmaniasis (VL) be treated in persons in North America, and what other management issues should be considered?

XXIV. How should HIV/AIDS-associated CL or ML be treated in persons in North America who do not have evidence of VL, and what other management issues should be considered?

XXV. What is the preferred treatment of visceral/cutaneous leishmaniasis in immunocompromised hosts with solid organ transplant, persons with lymphatic-hematologic malignancies, or persons receiving immunosuppressive therapy for other reasons?

XXVI. How should the treatment of leishmaniasis be modified in persons who are pregnant or lactating; are young children or older adults; or have comorbidities such as renal, hepatic, or cardiac dysfunction?

## BACKGROUND

The term leishmaniasis refers to a diverse group of syndromes caused by protozoa of the genus *Leishmania*, in the *Leishmania* and *Viannia* subgenera. The clinical manifestations of infection are variable and reflect a complex interplay between the human host's cell-mediated immune responses and the virulence and tropism of the infecting *Leishmania* species, >20 of which are known to be pathogenic for humans. Each leishmaniasis-endemic region has particular combinations of parasite species/strains, sand fly species, mammalian reservoir hosts (in zoonotic transmission cycles), and human hosts with different genetic backgrounds. Although *Leishmania* infection can be subclinical, the three main clinical syndromes are cutaneous leishmaniasis (CL), mucosal leishmaniasis (ML), and visceral leishmaniasis (VL). Less common presentations include diffuse cutaneous leishmaniasis, disseminated cutaneous leishmaniasis, leishmaniasis recidivans, bubonic leishmaniasis, uveitis, and post kala-azar dermal leishmaniasis (PKDL).

CL is the most common syndrome worldwide and the one most likely to be encountered in patients in North America. Although autochthonous CL cases acquired in Texas and Oklahoma have been reported, almost all of the cases of CL evaluated in North America occur among immigrants, international travelers, expatriates, and military personnel exposed in leishmaniasis-endemic areas elsewhere in the world. The skin lesions typically are first noticed at the site(s) where *Leishmania* parasites were inoculated by an infected sand fly. The lesions enlarge slowly and typically ulcerate after weeks to months, although persistently nodular and other forms also occur. The natural history is usually slow spontaneous healing as cell-mediated immunity develops; healing may be accelerated with antileishmanial treatment.

A minority of persons infected with *L.* (*V.*) *braziliensis* and related *Viannia* species in Latin America, particularly in parts of South America, develop metastatic ML after healing of CL or concomitantly with a cutaneous lesion(s). ML can progress to cause destructive lesions of the naso-oropharyngeal/laryngeal mucosa. Leishmaniasis with mucosal lesions also has been reported in the Old World, where the pathogenesis and clinical manifestations of mucosal infection may be different.

VL is potentially life threatening and requires prompt evaluation and treatment. In VL, amastigotes (the tissue stage of the parasite) disseminate throughout the reticuloendothelial system and occasionally are found in other organ systems. VL, and less commonly CL or ML, may be opportunistic infections in persons who are immunocompromised because of HIV/AIDS or other reasons. Some experts consider all persons with symptomatic VL to be immunocompromised, some perhaps without an identified immune defect. In these guidelines, immunocompetent VL refers to persons with VL without an identified immune defect.

Treatment of leishmaniasis can be challenging. The primary goals of therapy for VL and CL are to prevent mortality and morbidity, respectively. The only FDA-approved medications for leishmaniasis are intravenous liposomal amphotericin B (L-AmB) for VL, and oral miltefosine for CL, ML, and VL caused by particular species. The potential for VL to be life threatening and the high response rate associated with L-AmB therapy justify its side effects and cost. Treatment recommendations for CL are less straightforward, in part because the data from randomized control trials are of variable quality and generalizability. Many CL infections may clinically resolve without treatment. Furthermore, treatment does not necessarily result in parasitologic cure, as evidenced by cases of relapse, especially in the context of immunosuppression. The objective of treatment is clinical healing, not parasitologic cure. For CL, the interrelated goals of treatment include minimizing local tissue damage and cosmetic or functional consequences, accelerating the rate of healing, reducing the likelihood of local recurrences, and decreasing the risk of developing mucosal disease caused by parasites in the *Viannia* subgenus. The therapeutic strategy depends in part on the infecting species or, as a proxy, whether the infection was acquired in the Americas (New World cutaneous leishmaniasis; NWCL) or elsewhere (Old World cutaneous leishmaniasis; OWCL). Local therapy may be an option for some cases of CL, and use of systemic therapy may be indicated or prudent for others.

Clinical resolution of leishmaniasis may not be associated with a parasitologic cure, a definitive method to document parasitologic cure is not available, and even asymptomatic persons may have low concentrations of blood/tissue parasites that could cause infection in a transfusion/transplant recipient. We suggest that persons with a history of leishmaniasis (particularly, but not only, VL) refrain from donating blood; if organ/tissue donation is contemplated pre- or postmortem, pertinent agencies should be informed of the patient's history of leishmaniasis. The practices and policies for screening/deferring potential North American donors for *Leishmania* infection not only may change over time but also may vary among different places or settings (e.g., in the military vs the civilian sector), as well as for different types of donations, which also might be processed in

ways that could decrease the number or the viability of residual parasites.

For prevention of leishmaniasis in travelers, no prophylactic medications or vaccines are currently available. Healing of CL may be associated with some protection from clinical disease with subsequent exposure to the same *Leishmania* species/strain; however, persons should be informed that clinically manifest reinfection is possible and that they should use personal protective measures (PPM) that minimize vector exposure whenever they are in leishmaniasis-endemic areas. These measures include protective clothing, insect repellents such as DEET applied to exposed skin, permethrin applied to clothing, window coverings, and insecticide-impregnated bed nets. Vector control with residual insecticides has been used in settings with peridomestic transmission. Reservoir control depends in part on the infecting *Leishmania* species.

## METHODS

### Panel Composition

The Infectious Diseases Society of America (IDSA) and the American Society of Tropical Medicine and Hygiene (ASTMH) convened experts in the diagnosis and management of leishmaniasis from the fields of diagnostic parasitology, pediatrics, public health, tropical medicine, and infectious diseases, including experts from leishmaniasis-endemic areas.

### Literature Review and Analysis

Subgroups were formed, each responsible for developing recommendations and evidence support in the specific areas of diagnostic testing; treatment of cutaneous, mucosal, and visceral leishmaniasis; and issues associated with immunocompromised hosts and other special populations. Separate computerized searches of MEDLINE (primarily English language) through 2014, with some updates in 2015, were performed for each clinical question.

The supplemental Appendix summarizes clinical trials regarding the treatment of cutaneous leishmaniasis, including studies analyzed in two Cochrane reviews [2, 3]. We also included clinical trials and pivotal observational data (particularly, if they involved new therapies) identified through English-language PubMed searches using the terms leishmaniasis and treatment, starting with a 1-year overlap with the Cochrane reviews and extending through January 2015. The Appendix is organized to facilitate search by country of exposure, *Leishmania* species, and treatment modality; it provides transparent assessment of the quality of each study.

Process Overview:

The evidence evaluation process was based on the IDSA Handbook on Clinical Practice Guideline Development (Figure 1). In evaluating the evidence regarding the clinical management of leishmaniasis, the Panel followed a process developed by the Grades of Recommendation, Assessment, Development, and Evaluation (GRADE) Working Group (http://www.gradeworkinggroup.org/). This process utilizes a systematic weighting of the quality of the evidence and an assessment of the strength of the corresponding recommendation.

### Consensus Development Based on Evidence

The Panel had several in-person meetings, and conducted most of its work though monthly teleconferences and electronically based discussion during 2011–2016. All members of the Panel participated in the preparation and review of the draft guidelines, which were reviewed by the entire Panel. Feedback via external peer review was also obtained. The content of the guidelines and the manuscript was reviewed and approved by the IDSA and ASTMH guideline steering committees and the respective Boards of Directors before dissemination.

The subgroup-completed recommendations were discussed by the Panel and were finalized by electronic survey in a two-phase process in which responses and justifications for responses were anonymously summarized, revisions were made, and a final survey vote reached consensus on the GRADE assigned. In general, data from randomized controlled trials begin as "high" quality, and data from observational studies begin as "low" quality. However, the Panel may judge that particular features of the data warrant decreasing or increasing the quality-of-evidence rating; GRADE provides guidance on how various factors should be weighed [4]. The strength assigned to a recommendation chiefly reflects the Panel's confidence that the benefits of following the recommendation are likely to outweigh potential harms. Although the quality of evidence is an important factor in assessing the strength of a recommendation, it is not prescriptive.

### Guidelines and Conflict of Interest

The Panel complied with the IDSA policy on conflicts of interest, which requires disclosure of any financial or other interest that may be construed as constituting an actual, potential, or apparent conflict. Panel members were provided IDSA's conflicts of interest disclosure statement and were asked to identify ties to companies developing products that may be affected by promulgation of the guidelines. Information was requested regarding employment, consultancies, stock ownership, honoraria, research funding, expert testimony, and membership on company advisory committees. Decisions were made by IDSA on a case-by-case basis as to whether an individual's role should be limited as a result of a conflict. Potential conflicts of interests are listed in the Acknowledgments section.

### Revision Dates

At annual intervals, the Standards and Practice Guidelines Committee (SPGC) will determine the need for revisions to the guidelines, on the basis of review of current literature. If necessary, a Panel will be convened (or reconvened) to discuss potential changes.

### Background Information about Leishmaniasis

#### What clinical manifestations are suggestive of cutaneous leishmaniasis (CL)?

CL occurs in an afebrile person with a history of residence or travel in a leishmaniasis-endemic area of the world (Figure 2)

who has one or more chronic skin lesions. The usually painless lesions may be small or large, and nodular or ulcerative (Figure 4). Induration of the lesions is typical but purulence is not, unless lesion(s) are superinfected. Many persons do not recall being bitten by a sand fly and do not know how to distinguish sand flies from other small flying insects, but they still should be asked about potential exposures to sand flies.

The morphologic characteristics and natural history of CL depend in part on the infecting *Leishmania* species and the host's immunoinflammatory response. Clinically compatible features of the lesions include well-defined often indurated borders, chronicity, single or clustered lesions, and occurrence in exposed skin areas. Lesions typically are painless unless secondarily infected or over a joint. There may be regional adenopathy, subcutaneous nodules in a lymphatic drainage ("sporotrichoid") pattern, and satellite papules. Single or multiple lesions may occur where the parasite was inoculated by the sand fly but also may occur distant to that site such as at the sites of trauma [5]. The incubation period from inoculation to clinical manifestations is usually at least several weeks [6]. The lesions typically begin as papules, progress in size, and often ulcerate. Lesions may be chronic ulcers, papules, nodules, verrucous lesions, or plaques (Figure 4). Over time (months to years), the lesions usually spontaneously heal, typically with residual scarring.

The clinical manifestations of CL in HIV-infected and HIV-uninfected persons may be comparable, especially, but not only, in coinfected persons with minimal immunosuppression [7–10]. However, in general, the likelihood of having or developing atypical, multifocal, diverse, persistent, progressive, and remitting-relapsing lesions increases in the context of progressively more severe immunosuppression [11, 12]. Lesions may be unusual in interrelated respects, such as their type/appearance (e.g., pleomorphic, nonulcerative, papulonodular lesions), size, number, and distribution on the skin and mucus membranes [7–9, 12–18].

The differential diagnosis includes cutaneous fungal and mycobacterial infections, cutaneous actinomycosis/nocardiosis, yaws, skin cancer, pyoderma gangrenosum, sarcoidosis, venous stasis ulcers, cutaneous myiasis, spider bites, tropical ulcers, prurigo nodularis, lichen simplex chronicus, fixed drug eruptions, and vasculitis. In the more acute stage, bacterial skin abscesses, infected arthropod bites, and impetigo may be considerations; in the appropriate epidemiologic and clinical context, lack of response to antibacterial therapy should prompt diagnostic testing for leishmaniasis.

In addition, a chronic syndrome (skin lesion, naso-oral symptoms, or subacute febrile illness) associated with a granulomatous inflammatory reaction on histopathology may suggest leishmaniasis in persons with the appropriate history (even if remote) [19].

### What clinical manifestations are suggestive of New World mucosal leishmaniasis (ML)?

The diagnosis of ML is a consideration in the appropriate epidemiologic context (Figure 2) in persons with compatible naso-oropharyngeal/laryngeal symptoms or signs, especially if they have evidence or a history of active or healed NWCL. However, ML can develop in persons without a history of symptomatic cutaneous infection or any physical evidence (e.g., scars) of prior CL. The interval from onset (or clinical resolution) of CL to clinical manifestations of ML typically is several years but may range from <30 days to decades.

Persistent nasal congestion/stuffiness is the most commonly reported symptom [20, 21]; associated and interrelated manifestations may include coryza, epistaxis, tissue/scab expulsion, pruritus, mass sensation, blockage/obstruction, and hyposmia [20, 22–27]. Persons with ML may have oral or pharyngeal lesions, bleeding, or pain; dysphagia/odynophagia; or dysphonia. Isolated laryngeal disease, without involvement of other mucosal sites, may occur but is relatively unusual [22, 26].

Although ML typically does not directly affect the ears, involvement of the rhinopharnyx may affect the orifice of the Eustachian tube and thereby lead to "chronic secreting otitis media," the sensation of having a blocked ear, dyacussis, and tinnitus [25, 28]. Abnormalities of the paranasal sinuses (e.g., detected via computed tomography) also have been reported [21].

The differential diagnosis of ML includes infectious diseases (e.g., paracoccidioidomycosis, histoplasmosis, rhinosporidiosis, rhinoscleroma, leprosy, tuberculosis, syphilis, tertiary yaws), neoplastic diseases, and various other etiologies (e.g., granulomatosis with polyangiitis, sarcoidosis, intranasal cocaine use) [20, 25, 29, 30].

### What clinical manifestations are suggestive of visceral leishmaniasis (VL)?

VL presents in a person who has a history of residence or travel in a leishmaniasis-endemic area of the world (Figure 3) and develops a compatible clinical syndrome, which commonly includes chronic fever, weight loss, splenomegaly, pancytopenia, eosinopenia, elevated liver enzymes, hypergammaglobulinemia, and variable hepatomegaly. There is a spectrum of severity; and atypical presentations are common, especially in persons who are immunocompromised (see XXIII and XXV). The onset and course of VL are usually subacute or chronic but can be acute. Risk factors for the acquisition of VL include the bite of an infected sand fly but also needle sharing, laboratory accident, or receipt of a blood transfusion or organ transplant from an infected donor; uncommonly, congenital/perinatal (and, rarely, sexual) transmission has been reported. VL is a consideration even if the likely infection was acquired years to decades earlier (latent infection can reactivate). Immunocompromised persons with AIDS, organ transplant recipients, and persons treated with biologic immunomodulating drugs (such as tumor necrosis factor [TNF]-alpha antagonists) are at increased risk for reactivation and disseminated infection.

The spectrum of infection with *Leishmania donovani* and *L. infantum-chagasi* ranges from asymptomatic to classic VL, or kala-azar, which is characterized by fever; other constitutional symptoms, including malaise, loss of appetite, and wasting; splenomegaly, which can become massive; hepatomegaly; and various laboratory abnormalities, including hypergammaglobulinemia, anemia, leukopenia, thrombocytopenia, hypoalbuminemia, elevated acute inflammatory markers, and liver enzyme abnormalities. In addition, hyperpigmentation may be observed in persons infected in India and Bangladesh. Lymphadenopathy is seen in some persons in East Africa and occasionally elsewhere. Fever may be intermittent; remittent, with twice daily temperature spikes; or, less commonly, continuous. Detailed clinical descriptions of VL are available elsewhere [31–33].

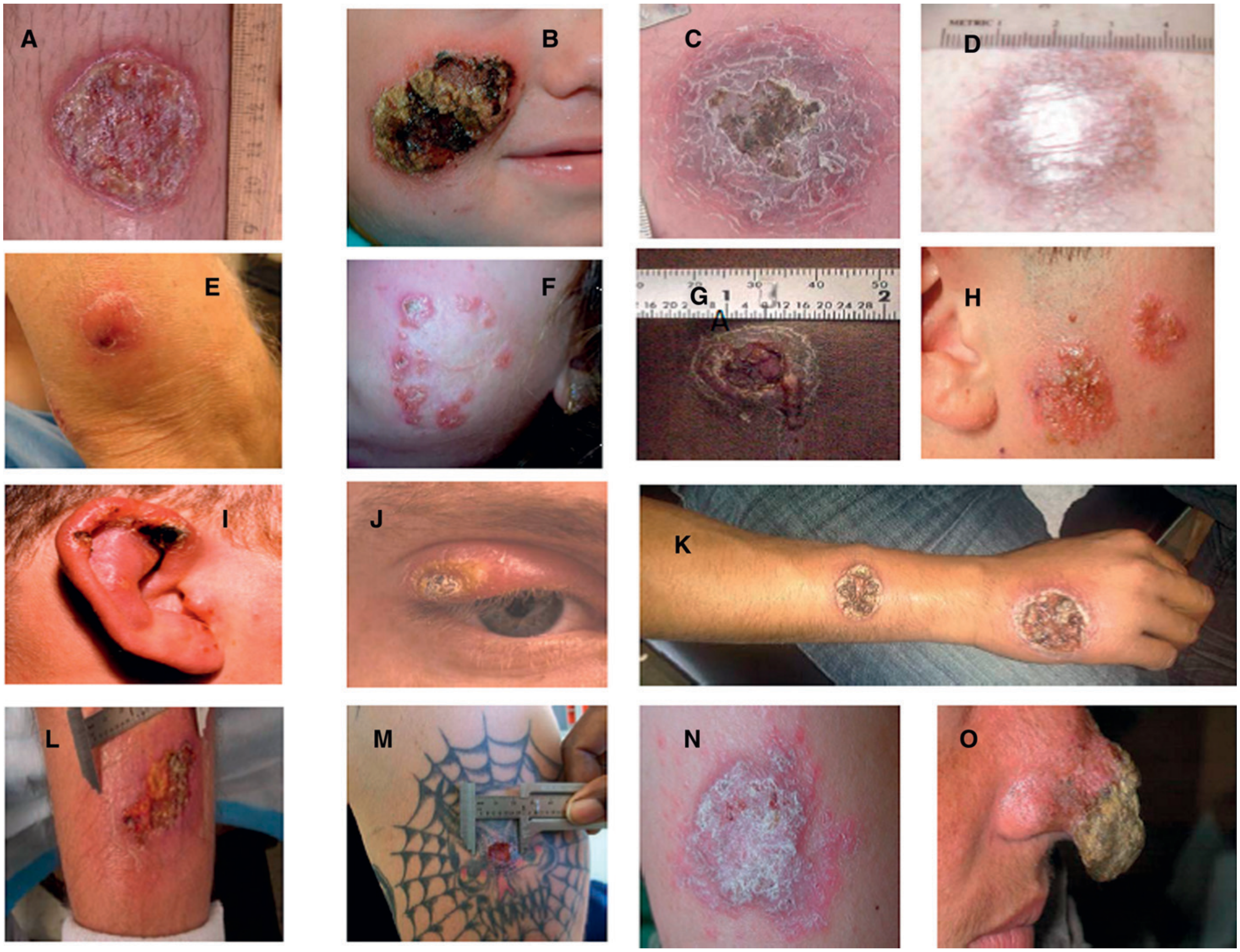


Figure 4. Clinical photographs of cutaneous Leishmaniasis (CL). A. Typical New World cutaneous leishmaniasis (NWCL) ulcerative lesion caused by *Leishmania* (*Viannia*) *braziliensis* infection acquired in Peru (the patient also had mucosal involvement). Photograph from Chris Ohl, Wake Forest Univ, NC. B. *L. tropica* CL, with thick crusted eschar that should be debrided before diagnostic testing or topical treatment. Photograph from Moshe Ephros. C and D: Before and after treatment of a *L. major* lesion, demonstrating the scarring nature of this infection. Photographs from Naomi Aronson. E. Nodular lesion caused by *L. infantum* infection, acquired in Sicily. Photograph from Christina Coyle, Albert Einstein University, NY. F. *L. tropica* leishmaniasis recidivans, with typical recurrence around the edge of a scar on the face. Photograph from Moshe Ephros. G and H. Secondary infection of CL lesions: G shows suppurative staphylococcal superinfection, and H shows impetiginous streptococcal superinfection. Purulence is not typical of CL unless secondarily infected. Photograph from Naomi Aronson. I. *L. mexicana* ulcerative lesion of the ear (Chiclero's ulcer), with a superficial necrotic appearance and edema. Photograph from Naomi Aronson. J. *L.* (*V.*) *panamensis* infection of the eyelid. Photograph from Naomi Aronson. K. Sporotrichoid NWCL; note the subcutaneous nodules along the lymphatic drainage and two large ulcerative lesions. Photograph from Peter Weina. L. Phlebitic change and large ragged ulcer caused by *L. major* infection acquired in northern Afghanistan. Photograph from Naomi Aronson. M. CL lesion over colored tattoo. Photograph from Naomi Aronson. N. Multiple small circumferential papules that formed soon after initiation of therapy for a plaque-like lesion caused by *L. major*. Photograph from Naomi Aronson. O. Verrucous CL on the tip of the nose of a patient in Afghanistan. Photograph from Peter Weina.

VL may be the first opportunistic infection in persons with AIDS, and it often complicates the terminal stage of HIV infection in *Leishmania*-endemic areas [34]. The clinical manifestations of VL in HIV-infected and HIV-uninfected persons often are qualitatively similar [11, 35–37], although some common manifestations of VL (kala-azar), such as splenomegaly, may be more subtle or absent in coinfected persons [38]. On the other hand, in coinfected persons, *Leishmania* parasites may be widely disseminated and found, often serendipitously, in atypical sites and cells, in essentially any organ system (e.g., the gastrointestinal tract and skin), with or without clinical manifestations or relevance [11, 38–42]. Persons with HIV-associated VL quite commonly have or develop dermatologic or mucosal involvement [11, 43–48], which may mimic other pathologies and may be localized or diffuse. Although PKDL most commonly is associated with *L. donovani* infection [37], in persons with concurrent HIV/AIDS, PKDL also has been associated with *L. infantum-chagasi* [49–54]. VL also occurs in persons who are or become immunocompromised for reasons other than HIV/AIDS (see XXV). The onset of clinical manifestations of VL may occur years or decades after the pertinent exposure in persons who become immunocompromised.

Although the clinical manifestations may be suggestive of VL in persons with exposure in a leishmaniasis-endemic area, they are not specific. The differential diagnosis is broad. When the onset is acute, it includes malaria, typhoid fever, typhus, acute Chagas disease (in Latin America), acute schistosomiasis, miliary tuberculosis, amebic liver abscess, mononucleosis, and viral hepatitis. In subacute or chronic cases, the differential diagnosis includes miliary tuberculosis, brucellosis, prolonged or recurrent *Salmonella* infections, subacute bacterial endocarditis, histoplasmosis or other disseminated fungal diseases, malaria with tropical splenomegaly syndrome (hyperreactive malarial splenomegaly syndrome), and hepatosplenic schistosomiasis with portal hypertension. Some noninfectious causes include lymphoma, leukemia, other myeloproliferative diseases, rheumatoid arthritis with Felty's syndrome, other autoimmune processes, and the hemophagocytic lymphohistiocytic syndrome (which is also associated with VL).

## RECOMMENDATIONS FOR THE DIAGNOSIS OF LEISHMANIASIS (CUTANEOUS, MUCOSAL, AND VISCERAL)

### I. In a person with a compatible skin lesion(s) and exposure history, what specimen(s) should be collected for diagnostic testing for CL?

*Recommendations*

1. Tissue specimens should be collected from a lesion(s) when a clinical suspicion for CL exists. Full-thickness skin biopsy specimens allow for simultaneous testing for other diagnoses, such as by histopathology and cultures [Strong, moderate].
2. Obtain a sample from a cleansed lesion, from which cellular debris and eschar/exudates have been removed [Strong, very low].

*Evidence Summary*

Samples for diagnosing CL should be collected from an active appearing (vs a nearly healed) skin lesion. Commonly used approaches for collecting samples include scraping or brushing the debrided ulcer base or edges, aspirating lesions, and obtaining skin snips or punch/shave biopsy specimens from an indurated border (see V). Additional details about specimen collection and diagnostic methodologies for various presentations of CL are provided elsewhere [55–57], including on the Centers for Disease Control and Prevention (CDC) website at http://www.cdc.gov/parasites/leishmaniasis/diagnosis.html. For collection of biopsy specimens (vs lesion aspirates or swabs), local anesthesia, such as with lidocaine plus epinephrine, typically is used (unless the lesions are on the face, genitalia, or digits, where epinephrine is not advised).

Which part of the skin lesion should be sampled to optimize the likelihood of diagnosing CL? The results of several comparisons of sampling the indurated edge vs the ulcer base in NWCL (Guatemala and Colombia) have varied; although the consensus, especially with more sensitive PCR-based diagnostics, is that the base has more parasites, this may not matter clinically. Obtaining samples from under the edge of the ulcer as well as from the base of the lesion has been suggested [58–61]. The optimal sampling site(s) for CL depends upon geographic variability; lesion age, location, and other characteristics; *Leishmania* species; and the sensitivity of the test procedure.

When initially evaluating a patient for CL, if laboratory support permits a quick assessment, it is useful to examine a scraping/aspirate/brushing or touch preparation from the lesion base, near the periphery. The best specimens are obtained from a well-cleaned, active-appearing lesion. Care should be taken to scrape without eliciting bleeding, and then to transfer the material onto a microscope slide for Giemsa staining and microscopy. The use of exudative material with minimal red blood cells on a smear makes identification of amastigotes (either extracellular or within macrophages) easier than in paraffin-fixed tissue sections. Alternatively, with the FDA clearance of the CL detect™ immunochromatographic assay (InBios International, Inc.), the tissue brushing from an ulcerative lesion can be processed with this rapid point-of-care assay (www.accessdata.fda.gov/cdrh_docs/reviews/k141341.pdf).

Molecular-based testing, such as polymerase chain reaction (PCR) analysis, is the most sensitive diagnostic approach [62]. Improvement in the ease and standardization of molecular techniques has led to increased use of such assays. We recommend collecting tissue for PCR analysis in 100% ethyl alcohol or by using the preferred collection method of your reference laboratory; sterility is not needed. For a diagnosis using PCR methodology, almost any tissue specimen may be acceptable. The handling of the material between sampling and testing is much more crucial. PCR analysis can identify *Leishmania* DNA in small tissue specimens, such as in the scab overlying the lesion, as well as from deep tissue (e.g., dermal) specimens. Lesions that are not ulcerative (a setting in which scraping may be less useful) or that are in cosmetically sensitive areas, such as the face, genitalia, or digits (where biopsy is less preferred), may be sampled by needle aspiration. A needle aspirate samples 3–5 areas, using a 1–3 milliliter (mL) syringe with a small needle (23–27G); some practitioners use a small amount of preservative-free sterile saline and inject then withdraw, whereas others use a dry syringe with a back-and-forth needle incursion into the lesion, simultaneously rotating and applying suction to the syringe for collection of a small drop of tissue fluid in the syringe. This fluid can be smeared on a slide and stained, and the aspiration procedure can be repeated for culture and PCR.

For *Leishmania* culture specimens, use sterile technique: avoid leaving residual iodine or alcohol on the lesion, which may interfere with culture yield. Full-thickness punch biopsy samples are usually obtained from the indurated edge of the skin lesion, where histology may have less degradation changes. In general, shave or punch biopsy specimens are recommended when the pre-test likelihood of leishmaniasis is lower (the differential diagnosis is broad) or when initial less-invasive sampling methods (such as brushing, scraping, or aspirating) do not identify an etiology.

### II. In a person with manifestations suggestive of New World mucosal leishmaniasis (ML), what types of specimens should be obtained for diagnostic testing?

*Recommendations*

3. The initial and most prominent mucosal manifestations typically are nasal (e.g., chronic unexplained congestion/secretions). Oral/palatal, pharyngeal, and laryngeal

involvement may develop as ML progresses or, in some persons, may be the first or the only noted abnormalities. The clinical signs, which may evolve over time, may include erythema, edema, hyperemia, infiltration, nodules, erosion, ulceration, and tissue destruction (e.g., perforation of the nasal septum) [FACT, no grade].

4. Mucosal areas that have macroscopic abnormalities are recommended for specimen collection; biopsy specimens, obtained by an otolaryngologist, are useful for confirming the diagnosis by molecular and traditional methods and for excluding other etiologies [Strong, low].

*Evidence Summary*

Many of the principles regarding specimen collection for diagnosis of CL also apply to ML; however, for ML, biopsy specimens typically should be collected by an otolaryngologist or other experienced specialist. In advance of the examination, the referring physician and otolaryngologist should discuss the differential diagnosis and the importance of assessing the anatomic extension and clinical severity of mucosal disease (if present), including the potential for respiratory obstruction. Details regarding specimen collection, handling, and testing for leishmaniasis and other potential etiologies should be discussed in advance with the pertinent laboratories.

In general, ML is a pauciparasitic syndrome [20,63,64], which underscores the utility of molecular amplification methods. However, obtaining mucosal specimens for *Leishmania* testing—even via relatively noninvasive means (e.g., via nasal swab or cytologic brush [24,30,65])—typically is not recommended for persons who do not have any macroscopic mucosal abnormalities. Because mucosal dissemination is more common than mucosal disease per se [24], detecting the parasite/DNA in naso-oropharyngeal mucosa does not suffice to diagnose ML. The risk factors for the development of ML are poorly understood (see clinical question XII), as are the factors that affect the progression and anatomic extension of ML over time. Investigational testing for the presence of *Leishmania* RNA virus (a purported virulence factor) is not readily available, nor, to date, has it been found useful for identifying persons who may have or be at risk for ML [66].

**III. During the initial and subsequent evaluations of persons with CL acquired in Central or South America who may have or be at risk for mucosal leishmaniasis (ML), what should be done to assess the possibility of mucosal disease?**

*Recommendations*

5. All persons at risk for ML—on the basis of the etiologic agent of the *Leishmania* infection, if known, and the region in the New World in which infection was acquired—should be questioned about and examined for mucosal symptoms and signs, respectively, even during the initial evaluation [Strong, low].
6. During all evaluations (i.e., initial and subsequent), persons at risk for ML should be questioned explicitly about the development, evolution, and other characteristics of mucosal symptoms; and they should have a thorough examination of the naso-oropharyngeal mucosa even if they do not have any mucosal symptoms [Strong, low].
7. Persons at risk for ML should be educated and provided personalized documentation about the importance of seeking medical attention for possible ML if they ever develop persistent, atypical (unusual for the person) naso-oropharyngeal/laryngeal manifestations that do not have a clear etiology [Strong, low].
8. Persons at risk for ML who have persistent mucosal symptom(s) or compatible abnormalities of the naso-oropharyngeal mucosa should be referred to a specialist for an otorhinolaryngologic examination, which typically should include fiberoptic endoscopy [Strong, low].
9. Clinicians might refer some at-risk persons without documented mucosal symptoms or signs to an otolaryngologist, especially if it was not possible to conduct a thorough review of systems and mucosal examination or if the assessments may not have been adequate or reliable [Weak, very low].

*Evidence Summary*

In New World ML, the anterior nasal septum is the most commonly involved area [22, 25, 63, 67], which, in contrast to the posterior nose, may be readily accessed even by non-otolaryngologists [20, 22]. Dried nasal secretions, if any, should be removed before beginning the examination. The external contour of the nose often is normal even in persons whose nasal septum has perforated, whereas the perforation may be palpated with the index and forefinger and may be visualized after lifting up the tip of the nose and shining a light at a diagonal angle inside the nares. The speculum examination of the nose may be facilitated by bending the nose from side to side. The oral cavity and pharynx should be inspected using a tongue depressor and a light; to visualize the entire hard palate, ask the patient to tip back his/her head [22].

The potential utility of otorhinolaryngologic examination for early detection of ML in persons with NWCL was evaluated in a *L.* (*V.*) *braziliensis*-endemic area of Bahia State, Brazil [68]. Among 220 consecutive persons with active CL who had a "careful" otorhinolaryngologic examination (anterior rhinoscopy, oropharyngeal examination, mirror laryngoscopy, and, if indicated, fiberoptic examination), concomitant ML was diagnosed in 6 persons (2.7%). All 6 persons were immunocompetent, and none had a history of CL or a compatible cutaneous scar; their cutaneous lesions had been present for 15–30 days (in 4 persons) or for 8 months (in 2 persons). ML was diagnosed during the persons' initial evaluation. Five of the 6 persons had mucosal disease that was restricted to the nose; the other patient, who was 1 of the 2 persons with an 8-month history of CL, had "pharyngeal and laryngeal involvement and had a nasal septal perforation." The publication about the study [68] did not address whether any of the identified cases of ML would have been missed altogether if the two-step approach described above in recommendations 5–8 had been followed—i.e., if complete otorhinolaryngologic examinations had been reserved for persons who had mucosal symptoms or signs that were detected via thorough assessments by nonspecialists.

The utility of a complete otorhinolaryngologic examination (anterior rhinoscopy, oropharyngeal examination, and a fiberoptic examination) for excluding ML was addressed by the same group of investigators in a study in Acre State, Brazil [29]. Among 44 persons with a clinical diagnosis of

ML—i.e., a nasal “clinical complaint” plus either a positive *Leishmania* skin test, a positive serologic result, or a previous diagnosis of CL—only 13 persons (29%) had evidence of active ML (10 persons) or healed/scarred mucosal disease (3 persons who already had been treated). Eight of the 10 previously untreated persons consented to having a biopsy specimen obtained; all 8 had positive *Leishmania* PCR results. Likely alternative diagnoses (e.g., allergic or atrophic rhinitis or chronic sinusitis) were identified for the persons who did not have evidence of ML.

A complete otorhinolaryngologic examination also may be warranted in persons who have disseminated CL—a syndrome distinct from localized and diffuse CL that has been reported primarily in northern and northeastern Brazil and that is associated with an increased risk for concomitant ML, particularly in persons with head or neck lesions [69, 70].

**IV. In a person with a compatible clinical course and epidemiologic context, what types of samples should be collected to evaluate for the diagnosis of VL?**

*Recommendations*

10. We recommend the collection of tissue aspirates or biopsy specimens for smears, histopathology, parasite culture, and molecular testing [Strong, low].
11. Bone marrow aspiration is the preferred first source of a diagnostic sample. Liver, enlarged lymph nodes, and whole blood (buffy coat) are other potential sources of tissue specimens [Strong, low].
12. Serum should be collected for detection of antileishmanial antibodies (see VIII) [Strong, moderate].
13. In immunocompromised persons, blood should be collected for buffy coat examination, *in vitro* culture, and molecular analyses [Strong, very low].

*Evidence Summary*

While there are several approaches to the diagnosis of VL, we recommend that the diagnosis be obtained by collection of tissue aspirates and/or biopsy specimens for microscopy (smears from aspirates and impression preparations from tissue), histopathology, parasite culture, and molecular testing (PCR) from venous blood and tissues [71, 72]. The specificity of microscopy for the diagnosis of VL is high, but its sensitivity varies among the tissues sampled, ranging from positive diagnoses in spleen (93%–99%), bone marrow (52%–85%), and lymph node aspirates (52%–58%) [73, 74]. Splenic aspiration is not recommended as part of the diagnostic evaluation for VL for persons in North America. Aspiration of the spleen is the most likely to yield a diagnosis but encumbers risk; life-threatening hemorrhages have been reported [75]. Bone marrow aspiration is less sensitive but safer and is the preferred first source of diagnostic sample. Liver, enlarged lymph nodes, and/or even whole blood are other potential sources of tissue specimens. In immunocompromised persons with VL, samples from atypical sites (e.g., gastrointestinal tract, bronchial alveolar lavage, pleural fluid, skin) may yield a diagnosis.

Serology with a rK39-based immunochromatographic test (ICT) [76, 77] may provide supportive evidence for a diagnosis of VL, but it is not recommended as a stand-alone VL diagnostic test; however, it may be useful to direct more invasive testing (see VIII).

A quantitative PCR is sensitive and specific when *Leishmania* parasitemia is above 10 parasites/mL in the venous blood (see VII) [78, 79]. Using a peripheral blood buffy coat sample for concentration before molecular testing is gaining widespread acceptance; a qualitative result can be obtained. However, because of the lack of standardization of the assay/target and undefined performance characteristics in North American reference laboratories (Table 2), we do not (currently) recommend using this evolving technique as first-line testing for VL.

**V. What laboratory tests should be used to diagnose leishmaniasis?**

*Recommendations*

14. We recommend using multiple diagnostic approaches to maximize the likelihood of a positive *Leishmania* result, using methods such as visualization of the characteristic amastigote in smears or tissue (histopathology); parasite isolation by *in vitro* culture; molecular detection of parasite DNA; and, for VL, serologic testing (see VI–VIII and Table 2). Simultaneous testing for other diagnoses (e.g., by histopathology and culture) should be considered [Strong, low].
15. We recommend attempting parasite isolation with the assistance of reference laboratories. We recommend that clinicians contact their leishmaniasis reference laboratory before collecting specimens (Table 2). If *Leishmania* parasites are isolated in culture, reference laboratories can identify the species by DNA-based assays or isoenzyme analysis [Strong, low].
16. Molecular amplification assays typically should be performed because they are the most sensitive *Leishmania* tests currently available (see VII) [Strong, moderate].
17. *Leishmania* skin testing is not recommended or available in the United States or Canada; there are no standardized, approved, or commercially available skin-test products in North America [Strong, very low].

*Evidence Summary*

There is currently no one, single “gold-standard” test for the diagnosis of leishmaniasis. Rather, a group of tests is typically performed: light-microscopic examination of tissue smears or sections (histopathology) for the presence of amastigotes, the tissue stage of the parasite; *in vitro* culture to isolate the parasite; DNA amplification assays; and, in VL, serologic testing. Many of these assays require reference laboratory support (Table 2). It is helpful to contact the leishmaniasis laboratory in advance to optimize specimen collection and transport.

Microscopy is the most widely available, but it does not allow for identification of the causative *Leishmania* species. Microscopy requires the visualization of *Leishmania* amastigotes (including cell membrane, cytoplasm, nucleus, and, in particular, the extranuclear rod-shaped kinetoplast) in tissue specimens by light microscopy with oil immersion at 1000X magnification [80]. Parasites can be seen using routine hematoxylin and eosin, Giemsa, or Wright-Giemsa stains. The morphologic identification of amastigotes is easier in smears than in tissue sections. The usual 3–5 um thickness

of tissue sections makes it more difficult to confirm the presence of a kinetoplast; thin sections are sometimes helpful. *Trypanosoma cruzi* amastigotes may also be seen in tissue specimens. Diagnostic confusion can result, particularly in immunosuppressed organ transplant recipients with skin lesions suggestive of CL that actually are caused by *T. cruzi* infection. *Histoplasma capsulatum* is of similar size but does not have a kinetoplast [55]. Diagnostic confusion can also result between VL and histoplasmosis in regions where histoplasmosis is endemic, a problem exacerbated by the response of both to empiric antifungal therapy (eg, with amphotericin B formulations). Although a relatively rapid method of discerning whether *Leishmania* parasites are present (histopathology takes 1–3 days to process, whereas smears and touch preparations take less time), microscopy requires substantial technical expertise. Biopsy quality and the usual 3–5 um thickness of tissue sections, may be factors that contribute to the relatively low sensitivity of histopathology, which has been estimated to be 50%–70% in aggregate for Old World species and 15%–30% for the New World species [81].

A new point-of-care rapid diagnostic test CL Detect™ (InBios International, Inc) has been FDA cleared for use in ulcerative CL skin lesions, with sampling of early (<4 months old) lesions. This qualitative immunochromatographic assay (test strip) reportedly has limits of detection of ~200 parasites for OWCL species and 1,000–1,440 parasites for NWCL species. In a study of OWCL in Tunisia, the sensitivity of the test was 96% and the specificity was 90.5% (www.accessdata.fda.gov/cdrh_docs/reviews/k141341.pdf).

If possible, parasite isolation (by culture) should be attempted because it provides parasitologic confirmation of the diagnosis and the isolate can be used for additional testing, if indicated. Some reference laboratories provide culture and transport media (Table 2). *Leishmania* are fastidious and it can take weeks for cultures to become positive; therefore, therapy is often initiated on the basis of other test results. Once a parasite is isolated, species identification is routinely performed at CDC (Centers for Disease Control and Prevention), Walter Reed Army Institute of Research (WRAIR), and other World Health Organization (WHO) reference laboratories by isoenzyme analysis and more recently DNA-based assays (Table 2). Despite providing a definitive diagnosis of *Leishmania* infection, culture is not a highly sensitive diagnostic method. Many specimens contain nonviable organisms or may become contaminated during collection or transport. An estimated 44%–60% of specimens yield parasites that can be expanded in culture and maintained in the laboratory [80]. Recently, MALDI-TOF (Matrix assisted laser desorption time of flight) has been used to assist with rapid species identification of cultured promastigotes [82, 83].

PCR analysis detects *Leishmania* DNA in tissue specimens (see VII and Table 2). There are many *Leishmania* molecular assays with different targets and performance characteristics. The advantage of this approach is that it can provide diagnostic results within 24 hours (although, in practice, it takes longer), is not dependent on having viable organisms, and typically has high sensitivity and specificity [62]. Depending on the primers and target sequence selected, one can identify the *Leishmania* genus, a subgenus complex, or a particular *Leishmania* species.

Immunologic diagnostic methods include serologic and delayed-type hypersensitivity testing (*Leishmania* skin test). Neither can distinguish past from current infection. Serologic testing (see VIII) is recommended for persons with suspected VL in whom definitive diagnostic tests for the parasite (microscopic identification, culture, and molecular tests for parasite DNA) cannot be conducted or have negative results. Serologic tests are not reliable for the diagnosis of CL. Skin testing is not recommended or available in the United States or Canada for any form of leishmaniasis. There are no standardized, approved, or commercially available products in North America. Delayed-type cutaneous hypersensitivity responses to leishmanial antigens are typically not observed in untreated persons with kala-azar and are variably seen with *L. infantum-chagasi*.

### VI. In a person with leishmaniasis, why could it be helpful to identify the infecting *Leishmania* species?

*Recommendation*

18. We suggest that identification of the infecting parasite to the species level be attempted in cases of suspected CL. Species identification may help inform clinical management decisions for individual persons (e.g., whether and how to treat) [Weak, moderate].

*Evidence Summary*

Although *Leishmania* species identification is not necessary to confirm the diagnosis of CL, ML, or VL, identifying the species can help inform clinical management decisions for individual persons. For example, species identification stratifies potential risk for associated ML, allows estimates of the natural history of the infection, and may help predict the response to a particular therapy (see XII and XIII). If the individual situation is such that none of these factors are assessed to be relevant, species identification may be foregone or treatment may be initiated before the species results are available. However, species identification should be pursued if a person has a complex residence/travel history (if more than 1 species is found in the pertinent region[s] and infection caused by those species have clinically relevant differences that affect choice of treatment, the dose/duration of therapy, the prognosis, and posttreatment monitoring), became infected in the "mucosal belt" of South America (Table 1), is immunocompromised, or has comorbidities that may affect the treatment risk-benefit assessment (e.g., young or old age, pregnancy or lactation, liver or renal disease; see XXVI and Table 4). Classically, species identification has been accomplished using multilocus enzyme electrophoresis (MLEE) with culture-adapted parasites. Molecular techniques often allow rapid species identification directly from tissue, from many different available specimen sources, with the caveat that the assays may not have been fully characterized and validated [84]. In addition, there may be irreconcilable discrepancies between the molecular and the MLEE results.

These recommendations are based on clinical experience and a summary of the limited available data as outlined in a recent review article about species-directed therapy [85]. Decisions regarding clinical management also must take into account the availability (or lack thereof) and feasibility of particular therapeutic options.

## VII. What is the role of DNA-based assays in the diagnosis of leishmaniasis?

*Recommendation*

19. DNA-based assays should be performed, especially if other diagnostic testing is unrevealing. They are emerging as the most sensitive assays for the diagnosis of leishmaniasis [Strong, moderate].

*Evidence Summary*

In North America, DNA-based molecular assays should be performed if direct visualization of the parasite is unsuccessful and the index of suspicion is high for any form of leishmaniasis. These assays include conventional PCR, real-time PCR, nucleic acid based amplification (NASBA), and loop-mediated isothermal amplification (LAMP). They are the preferred methodology where experienced microscopists are not readily available. They are currently the most sensitive assays for the detection of *Leishmania* species (spp.) and are particularly useful in situations where few parasites are present [62, 86–89]. A key point is that there is no single standard *Leishmania* PCR assay, and many assays differ with respect to parasite targets and performance characteristics. For example, current PCR targets at North American reference laboratories vary—e.g., glucose-6-phosphate isomerase (GPI), cathepsin L-like cysteine protease B gene, and leishmanial ribosomal RNA (rRNA).

Samples for PCR analysis do not have to be sterilely collected and can be inoculated onto filter paper and dried, allowing for specimen preservation and easy transport. Although the preferred sample is a fresh tissue specimen preserved in absolute alcohol, PCR assays are also useful in identifying the *Leishmania* species in paraffin-fixed tissue and cultured isolates. For persons with HIV/AIDS, quantitative *Leishmania* loads in the blood, buffy coat, and bone marrow are used in VL management [89, 90]. Although PCR-based assays are not commercially available in North America, molecular analyses are performed at reference laboratories such as at CDC and WRAIR in the United States and at the National Reference Centre for Parasitology in Montreal, Canada, as well as at other WHO leishmaniasis collaborating centers worldwide (Table 2). Quantitative PCR is not available in any North American reference laboratory as of the time of writing these guidelines; however, clinicians should contact reference laboratory directors for updated information about availability.

Potential limitations of these molecular methods include the requirement for technical training and the need to avoid specimen contamination [91]. Other issues include the infrequent validation of the assay for the geographic area where the infection was acquired and a need for contextual assurance that the assay result is consistent with the potential region of exposure [92]. Real-time PCR (RT-PCR) methodology has been developed, which uses fluorescent signal as an indicator of amplification products. This approach is an improvement over older PCR methods; it is highly specific, less labor intensive (which reduces the risk for contamination), and provides results in <1 hour (although, in practice, it takes longer) [93].

Multilocus enzyme typing (MLEE), which is technically complex and time-consuming, is a potentially more powerful methodology than molecular analysis for the identification of *Leishmania* spp., but it needs cultured parasites and lacks the discriminatory power of newer techniques like multilocus sequence typing [94]. MALDI-TOF—a technology that is being widely introduced into clinical microbiology laboratories in North America—recently was reported to assist in the diagnosis of leishmaniasis and to identify the *Leishmania* species [83].

## VIII. What is the role of serologic testing in the diagnosis of leishmaniasis?

*Recommendation*

20. Serologic testing is recommended for persons with suspected VL in whom definitive diagnostic tests for the parasite (microscopic identification, culture, and molecular tests for parasite DNA) cannot be conducted or have negative results. The sensitivity and specificity of serologic tests depend on the assay and antigens used, as well as host factors. Serologic tests cannot be used to assess the response to treatment. Antileishmanial antibodies can be detected years after clinically successful therapy in some persons [Strong, moderate].
21. We suggest that tests for antileishmanial antibodies not be performed as the sole diagnostic assay. Antibodies may be undetectable or present at low levels in persons with VL who are immunocompromised because of concurrent HIV/AIDS or other reasons. The potential for false-negative test results limits the utility of serologic assays in this setting [Weak, low].
22. Serologic testing is not recommended as part of the diagnostic evaluation for CL. The currently available serologic assays are neither sensitive nor specific for the diagnosis of CL [Strong, moderate].

*Evidence Summary*

A number of assays can be used to detect antileishmanial antibodies. The sensitivity and specificity vary depending on the leishmanial antigen(s) and platform used as well as the patient populations studied. Whole or solubilized promastigotes and recombinant antigens have been used (see Background information about leishmaniasis).

The best characterized is the recombinant K39 kinesin-like antigen expressed by amastigotes cloned from *L. infantum-chagasi* [95, 96]. In the United States, the immunochromatographic dipstick (Kalazar Detect,™ InBios International, Inc., Seattle, WA) is an FDA-cleared diagnostic test. In a meta-analysis of 13 publications [97], the sensitivity and specificity of the rK39 dipstick for symptomatic VL in populations unlikely to have HIV/AIDS were 94% and 91%, respectively. The sensitivity was higher in studies conducted in South Asia [98] and Latin America [99] than in East Africa [100, 101], where the sensitivity reportedly ranged from 75% to 85% and the specificity from 70% to 92% [102, 103]. The specificity tends to be highest in healthy control populations [97]. False-positive rK39 reactions have been reported in Latin America in persons with Chagas disease or CL [99]. On occasion, the rK39 results are positive in asymptomatic

persons infected with *L. donovani* or *L. infantum-chagasi*, including persons who may later progress to develop VL. Unfortunately, assays for antileishmanial antibodies are not helpful as a test of cure. In a study of 780 persons treated for VL in India, antibodies to rK39 were assessed using an ELISA format. The titers decreased rapidly during the first 12 months following treatment but then declined slowly, with 39% of patients still seropositive more than 15 years after treatment [104]. The presence of antileishmanial antibodies years after treatment for VL has been reported in cohort studies performed in India, South America, and East Africa [95].

Some North American laboratories perform indirect immunofluorescence assays (IFA) or enzyme-linked immunosorbent assays (ELISA) using crude promastigote or other characterized leishmanial antigens. The sensitivity and specificity vary with the platform and antigens used, as well as the threshold for considering a result positive [95, 105]. False-positive results have been reported in persons with CL, Chagas disease, leprosy, tuberculosis, typhoid fever, malaria, and other diseases [106, 107]. The National Reference Centre for Parasitology in Montreal, Canada, offers *Leishmania* IFA testing. There is also a direct agglutination test (DAT, Dutch TB Laboratory Partnership, Royal Tropical Institute, Amsterdam), using whole *Leishmania* promastigotes, that has been evaluated in a number of leishmaniasis-endemic areas. In a meta-analysis [97], the sensitivity and specificity were reported to be 95% and 86%, respectively. This assay is not available in North America.

Antileishmanial antibodies may be diminished or undetectable in persons who are coinfected with HIV/AIDS. In a recent meta-analysis, the sensitivity in persons coinfected with HIV/AIDS ranged from 51% to 84% and the specificity from 82% to 93%, depending on the assay [108]. In studies in Ethiopia and Brazil [109, 110], 77% and 82% of persons with concurrent VL and HIV/AIDS were seropositive using rK39-based assays. However, in solid organ transplant recipients [79] and a few cases of persons treated with TNF-α antagonists [101], serologic tests have not appeared to be of reduced sensitivity in persons with VL.

Kalazar Detect™ (InBios International, Inc) test for antibodies against rK39 is known to have poor sensitivity in persons with CL. In a cohort of otherwise healthy military personnel who had CL (mainly OWCL), the sensitivity of the rK39 dipstick assay was 10.2% and of the rK39 ELISA was 28.8% [111]. In a study of 242 persons with confirmed CL in a *L.* (*V.*) *braziliensis*-endemic area in Brazil, none tested positive with the dipstick assay [112]. A number of other studies of serologic responses in CL have used leishmanial antigens derived from cultured promastigotes. The *Leishmania* spp., the methods of antigen preparation, and the platforms have varied widely, as have the reported sensitivities and specificities [81]. On the basis of the available data, serologic testing is not recommended for the diagnostic evaluation of CL.

## RECOMMENDATIONS FOR THE TREATMENT OF LEISHMANIASIS

### Cutaneous leishmaniasis

**IX. In a person with a consistent travel history and compatible skin lesion(s), is it necessary to obtain parasitologic confirmation of the diagnosis of leishmaniasis before starting treatment?**

*Recommendation*

23. After a careful diagnostic evaluation in which neither leishmaniasis nor another diagnosis is confirmed, empiric treatment may be indicated on the basis of an individualized risk-benefit assessment [Weak, very low]. *Remark: This should be discussed with the patient and reevaluated periodically, taking into account the clinical evolution.*

*Evidence Summary*

This recommendation derives from opinion based on clinical experience. We strongly prefer to have a confirmed diagnosis to inform treatment and to provide prognostic information; otherwise, we manage the skin lesion(s) as a calculated risk-benefit decision. Clinical appearance (see Background) must be suggestive, although a variety of appearances are possible. Certainly, one should first ascertain that CL is endemic and plausible in the region of exposure (Figure 2). Knowing what species of CL are endemic there also may be helpful [85]. CL may occur in clusters of exposed persons (e.g., in travelers or military groups); if efforts to make a specific diagnosis (particularly in NWCL) fail to confirm the presence of parasites, the pretest likelihood that the clinical manifestations and epidemiology are compatible with CL should be considered—e.g., if fellow travelers have confirmed cases of CL, the likelihood that a person with skin lesions has CL is higher.

*L.* (*V.*) *braziliensis* CL and leishmaniasis recidivans (*L. tropica*) persisting for many months may have fewer parasites present, thus making diagnostic confirmation difficult [113, 114]. The availability of diagnostic molecular methods has increased the sensitivity of detection, such that negative test results are less likely (see VII). In this circumstance, obtaining a skin biopsy specimen to look for other etiologies may be helpful. The histopathologic milieu of CL may include well-formed granulomas but also lymphocytic/plasma cell infiltration; the stratum corneum may be hypertrophied and can also be ulcerated with necrosis [115].

If the exposure was south of Nicaragua (i.e., in Costa Rica or further south), particularly in the so-called mucosal belt in South America, concern about risk for mucosal involvement may prompt empiric treatment based on clinical impression. This, as well as the potential toxicities associated with particular medications (Table 4), should be considered in individualized treatment decisions. Oral miltefosine, which recently became available in the United States, may have a role in this circumstance, although it also can be associated with adverse effects. OWCL skin lesions can often be treated with nonspecific local measures, which also could be indicated for other similarly appearing conditions and are associated with less toxicity than available systemic agents.

**X. Is treatment of clinically manifest cutaneous infection (CL) always indicated?**

*Recommendations*

24. We recommend that immunocompetent persons with skin lesions that are caused by infection with

*Leishmania* species that are not associated with increased risk for ML, that are defined as clinically simple lesions (Table 1), and that are healing spontaneously may be observed without treatment if the patient concurs with this management [Strong, moderate].
25. For persons with CL when the *Leishmania* species is not known but the infection was not acquired in an increased ML-risk region (Table 1, Figure 2), treatment of clinically simple or healing skin lesions is not required in an immunocompetent patient who concurs with this management [Strong, low; E.C. dissents, recommending that all persons with NWCL receive treatment]. *Remark: See XXIV and XXV regarding the management of CL in immunocompromised persons*.
26. We suggest that systemic treatment be offered for persons even with healing/recently healed CL lesions caused by increased ML-risk species or when the species is unknown but the infection was acquired in an increased ML-risk region. Risks and benefits of such treatment should be discussed with the patient [Weak, low]. *Remark: In some cases, watchful waiting, with vigilance for signs and symptoms of ML, may be a reasonable approach.*
27. We recommend that any decision to observe a patient with CL without treatment should be reevaluated periodically, and the decision not to treat should be reconsidered if healing does not progress as anticipated [Strong, very low].
28. In all cases of CL, wound care, individualized documentation of lesion evolution, and patient education regarding the manifestations and detection of local therapeutic failure/relapse and ML should be routine components of management (see III and XV) [Strong, low].

*Evidence Summary*

These guidelines assume a setting with ready access to medical resources and availability of most relevant treatment modalities. Persons with CL will be involved in the assessment of risks and benefits associated with treatment, and potential legal liabilities for adverse outcomes will require consideration as well. These factors may lead to recommendations that differ from those in some leishmaniasis-endemic areas.

When deciding whether to treat a case of CL, one must consider the goals of treatment. CL can lead to morbidity but does not directly cause mortality. The primary goal is to accelerate healing of the lesion(s) and, thereby, to minimize tissue damage, scarring, and disfigurement; appropriate treatment is also thought to reduce the risk for subsequent therapeutic failure, including ML.

In some instances, at the time of diagnosis, lesions already will show evidence of spontaneous healing. Some lesions may also be considered “simple,” in the sense that they are uncomplicated (Table 1) and seem unlikely to lead to substantial morbidity. In particular, the lesions are small in size, are few in number, and are not localized on parts of the body with the potential for cosmetic or functional consequences.

When assessing whether treatment may be indicated, there are several patient categories to consider:

1) *Known species, low ML risk, clinically benign lesions*: The infection was acquired in the Old World or it was acquired in the New World but is known to be caused by a species not in the *Viannia* subgenus or is caused by a *Viannia* species from a region north of Costa Rica. In addition, the lesions are clinically benign or reportedly are spontaneously healing, and the patient is not immunocompromised. In these cases, the risks for ML and other complications are low and observation is a reasonable approach. However, persons with lesions that are not shown to heal subsequently may be offered treatment.
2) *Region of acquisition known, low ML risk, clinically benign lesions*: The infecting species is unknown, but infection was acquired in a region where ML is rare. In addition, the lesions are clinically benign or reportedly are spontaneously healing, and the patient is not immunocompromised. In these cases, the risks for ML or other complications are low, and observation is a reasonable approach. However, persons with lesions that do not subsequently heal should be offered treatment.
3) *Increased ML risk*: The infection is caused by a *Viannia* species from Costa Rica or further south or the species is not known but infection was acquired in a region where ML is endemic. Treatment should be offered even if the lesions are spontaneously or recently healed.

The natural history of CL has received limited study, although the placebo groups of clinical trials provide some information. There is a variable tendency for lesions to spontaneously heal within approximately 2–6 months (e.g., *L. major*), 3–9 months (e.g., *L. mexicana*), or 6–15 months (e.g., *L. tropica*, *L.* [*V.*] *braziliensis*, or *L.* [*V.*] *panamensis*) of disease onset [116]. On the basis of this knowledge, observation alone may be considered for lesions expected to heal in a short time (e.g., 1–3 months) and that are clinically simple as defined in Table 1. Observation may also be preferred when therapy, especially systemic therapy, would ideally be delayed, such as during pregnancy (see XXVI) or during treatment for other conditions that may interfere or interact with the antileishmanial agents. Anecdotally, observation is poorly accepted by many persons as a management approach. Unfortunately, there are no reliable clinical predictors of delayed healing or therapeutic failure, other than the demonstration that both phenomena are complex and are related to variability in the host immune response as well as strain factors [117, 118]. Some factors associated with slower healing are discussed in section XV. Whether lesions have healed spontaneously or after treatment, risk for therapeutic failure remains and it is difficult to quantify for an individual immunocompetent person.

The primary concern related to observation of CL without treatment is that, despite apparent spontaneous healing, metastatic infection in the form of mucosal involvement may occur. Mucosal disease may cause destructive lesions that are difficult to treat and may lead to severe sequelae (see II). A careful nasal-oropharyngeal examination should always be performed (see III). An additional concern is that apparently healing cutaneous disease may persist for long periods or relapse. Treatment can mitigate both of these potential complications [116].

The risks for ML in leishmaniasis-endemic areas have been summarized [119]. *L.* (*V.*) *braziliensis* is the New World species most often associated with ML, which can occur years, even decades, after acquisition of infection. The incidence of mucosal involvement associated with CL seems to

vary with geography and species/strain. Generally, it is highest in the Amazonian basin and adjacent lower altitudes of Andean South America, particularly Bolivia, Peru, and Brazil, and lower in Colombia, Venezuela, Argentina, and Central America. Systemic treatment should be offered for *L.* (*V.*) *braziliensis* infections and other cases in category 3, above. The ML risk is lower outside of South America and the Central American countries from Costa Rica southward. Importantly, case reports of disease imported into North America and Europe by travelers have suggested limited imported mucosal disease from outside these regions [120–132]. NWCL caused by non-*Viannia* species and OWCL are very rarely associated with ML, although cases have been reported in immunocompromised and some immuncompetent hosts [133–135]. Patient education regarding this possibility should be a standard component of care. The evidence for the effectiveness of systemic treatment in preventing ML is summarized in clinical question XII.

Another point of consideration in selecting a treatment plan includes that, although often considered a clinically less virulent pathogen, *L. mexicana* has rarely been associated with a more severe condition known as diffuse cutaneous leishmaniasis (DCL) [134]. DCL has been reported as caused by other species, including *L. amazonensis, L. venezuelensis*, and *L. pifanoi* [42, 136–138]. This syndrome appears to be a type of anergic, severe, and chronic nonulcerative, plaque-like form of CL, thought to be related to a host immunologic defect. Treatment is often unsatisfactory. Systemic treatment is usually given, but relapse is typical when treatment is stopped. Miltefosine may yield a better initial response rate than antimonials, but some type of chronic or intermittent therapy may be required [139].

Regardless of whether antileishmanial therapy is administered, standard wound care should be applied to ulcerative skin lesions until they have fully reepithelialized. Although there are few trials of particular interventions, clinical experience supports the importance of these common measures. They include control of secondary infection, gentle debridement of necrotic tissue if present, and moisturizing to promote tissue regeneration. Daily ulcer cleansing with mild soap and allowing water in the shower to run over the lesions is advised. A thin layer of a petroleum-based ointment (e.g., Aquaphor®, petroleum jelly, Vaseline®) should be applied after bathing; other moisturizing creams used in leishmaniasis include Aquaphilic® or Eucerin® products. Occlusion does not seem to be needed but can be used when wound drainage is present.

## XI. In a person with CL, what could be the consequences of no treatment or suboptimal therapy, and how should persons who received no or suboptimal therapy be monitored?

*Recommendations*

29. Potential consequences of inadequate treatment include poor cosmetic outcome due to scarring or superinfection, the persistence of a chronic wound(s), and, with some *Leishmania* species, destructive and disfiguring ML. In immunocompromised persons, cutaneous, mucosal, and visceral dissemination may occur [FACT, no grade].
30. Persons with CL should be actively monitored by clinical appearance, including by performing a careful nasal and oropharyngeal examination periodically up to 1 year, or at least 2 years if at increased risk for ML. They should be educated about the signs and symptoms of relapse and ML and instructed to seek medical attention anytime these appear [Strong, low].
31. Symptoms such as chronic nasal stuffiness, epistaxis, or hoarseness or findings such as septal perforation that occur anytime in a person with a prior or current diagnosis of CL or a scar consistent with prior CL should prompt evaluation for ML, including fiberoptic examination of the affected area if relevant (see II and III) [Strong, moderate].

*Evidence Summary*

When considering whether to treat CL in persons who have not been previously treated or who have received suboptimal treatment, the potential consequences of the infection should be considered. The most common morbidity relates to scarring of the lesions, which can be extensive. Scars and pigmentary changes, especially if on the face, can have substantial aesthetic consequences. Scars that are over joints, such as those in the fingers, can impair function. In the short term, optimal treatment accelerates healing and may reduce tissue destruction. Faster healing presumably also reduces the opportunity for bacterial superinfection and the associated complications. These benefits have been confirmed by clinical experience, although rigorous assessments of benefits are lacking. In the midterm, treatment reduces the likelihood of persisting lesions or relapse, whether local, regional (e.g., lymphatic spread), or more distant. In the long term, although the evidence is indirect, the probability of developing ML appears to be reduced, and there may be a reduction in late reactivation (e.g., in the context of immunosuppression).

Regardless of whether treatment is administered, careful objective documentation of the evolution of the skin lesions is important for guiding clinical management. Photographic records of lesions are often helpful; even photographs taken by patients using their own cameras or smart phones can be helpful. Observations should be made approximately every 2–4 weeks until lesions have reepithelialized and less frequently thereafter. The surface area and induration should be recorded, as well as the proportion of the ulcer base that has reepithelialized. The lesion(s) size, location, and associated subcutaneous nodules or adenopathy should be noted, especially new findings around the original lesions and along lymphatic drainage pathways. Evidence of secondary infection of any lesion and adjacent tissues should be described, including pain, purulence, and fluctuance. Superinfection may require debridement of eschar. The time course of healing is further discussed in XV. Careful examination of the mouth and nose should be included in all follow-up evaluations (see III); the indications for referral to a specialist for a complete otorhinolaryngologic examination are discussed in III. The likelihood of therapeutic success and failure has proven very difficult to predict in the individual patient. Healing rates appear to be influenced by host genetics and immune responses, the *Leishmania* strain, and even the strain of the vector sand fly [116]. There are few long-term studies monitoring defined cohorts of infected patients for therapeutic failure. Reappearance of lesions at the same site is generally presumed to represent relapse. Clinically manifest reinfection is rare with *L. major* and has not been well studied for other

species. The extent to which infection with one species/strain protects against infection (or disease) with another is also poorly understood [140].

Most relapses of cutaneous lesions occur within 1 year, although much longer intervals have been reported with extended follow-up periods [141, 142]; and viable parasites have been isolated from healed scars years later [143]. There are many case reports of later recurrence associated with acquired immunosuppression [118].

Similar to CL recurrence, the risk for ML following NWCL is highest within 2 years of the onset of the initial skin lesion (see III) [22]. Persons should be informed that even after routine follow-up has ended, they may still be at risk of developing ML. Severe sequelae from ML include destructive lesions causing nasal, oral, pharyngeal, and airway complications (see II and III); treatment of advanced ML is often difficult and unsatisfactory (see XVII). When planning patient follow-up, it can be challenging to balance the relatively low risk for late ML against the costs and discomfort of long-term surveillance for ML symptoms and signs. The risks associated with laryngoscopy and biopsy are minimal but should nonetheless be considered when discussing such surveillance [144]. Patient values and preferences are important when developing follow-up plans.

## XII. In a person with CL, what factors should prompt consideration of use of a systemic (oral or parenteral) agent for initial therapy?

*Recommendations*

32. Systemic treatment is recommended for persons with complex CL as defined in Table 1 [Strong, moderate].
33. Initial systemic therapy (see XIII) may be used in persons with CL in whom it is not practical to use local therapy or (possibly) if more rapid healing of large, cosmetically or functionally concerning lesions is preferred [Weak, very low].
34. Less common cutaneous syndromes, such as leishmaniasis recidivans (caused by *L. tropica* and occasionally other species), diffuse cutaneous leishmaniasis (caused by *L. mexicana, L. amazonensis*, and *L. aethiopica*), and disseminated cutaneous leishmaniasis (caused by *L.* [*V.*] *braziliensis*), usually require systemic therapy [Strong, low].

*Evidence Summary*

The most important reason for giving systemic therapy is the treatment of local or distant dissemination. Systemic (vs local) therapy involves the reliable and sustained delivery of the therapeutic agent to the infected tissues, including distant sites of potential spread. For this reason, systemic therapy has become the standard of care for all disseminated infection, and such cases are generally excluded from studies of local therapy. This principle also relates to prevention of ML, presumably by treating organisms that already have seeded the naso-oropharynx and, perhaps, by accelerating healing and thereby reducing the opportunity for metastatic spread.

Other infections defined as complex (Table 1) may also benefit from systemic therapy. These include large, multiple, or difficult-to-access lesions that make local treatment (see XIV) technically difficult or impossible. Data from comparative studies regarding simple (Table 1) leishmaniasis do not suggest that systemic therapy is superior to local therapy for uncomplicated infection (see XIV). Some experts think that systemic therapy may lead to more rapid healing of lesions compared with local treatment, with possible associated benefits in terms of scarring and superinfection, although we did not identify published data to support this impression.

Systemic treatment options include parenteral and oral therapies (Table 3). Choosing the optimal therapy for a particular case is difficult, given the limited information available from comparative clinical trials and the substantial methodologic or other limitations of many studies. The majority of treated CL persons in South America are treated with parenteral pentavalent antimonials ($Sb^V$). In several recent clinical trials, miltefosine and parenteral $Sb^V$ therapy were compared, yielding results that supported FDA approval of miltefosine for this indication. There are limited published data regarding other systemic agents, such as amphotericin B deoxycholate, liposomal amphotericin B (L-AmB), and the oral "azoles." Data from published clinical trials are summarized in Appendix 1. It remains unclear how the results of individual studies can be extrapolated to other species, geographic areas, and patient populations where data are unavailable.

Systemic therapy of NWCL (compared with no or suboptimal therapy) appears to reduce the risk for subsequent ML. If ML is considered to be a risk, treatment selection should be based on predicted efficacy, patient tolerance of risk for therapeutic failure, toxicity, practicality, availability, and cost (see XIII). ML can cause disfigurement and disabilities, especially if the diagnosis and treatment are delayed (see XVII).

No controlled clinical trials have compared the incidence of ML after treatment of NWCL with systemic versus local therapy (see XIV). Observational studies have generally found incidence rates of ML following CL caused by *L.* (*V.*) *braziliensis* to be 2%–10% [119] and close to 30% in some reports [145]. On the basis of retrospective estimates in an actively surveyed population of >3,000 CL persons in a *L.* (*V.*) *braziliensis* focus in Peru, the lifetime risk for ML was 12.8% [146]. Unfortunately, study methods have been highly variable and often poorly described. It is difficult to use individual studies to assess variations in risk associated with different geographic regions, *Leishmania* species/strains, and host factors; of note, the proportion of patients treated and the types of treatment used in the study population have not always been described, further complicating interpretation of the findings.

The evidence that systemic therapy (compared with no or suboptimal therapy) may reduce the risk of developing ML derives from 3 types of studies: 1) treatment trials of NWCL (presumed or proven *L.* [*V.*] *braziliensis*) with long-term follow-up showing rates of ML that appear to be substantially lower than those described above; 2) case series that suggest that most of the persons with ML had not received adequate treatment for their prior CL; and 3) clinical trials of NWCL in which subjects were observed over extended periods, such that the incidence of ML could be compared between treated subjects and those who received incomplete, ineffective, or no therapy.

There have been several studies of the first type. Among 658 persons with presumed *L.* (*V.*) *braziliensis* CL in Brazil (Rio de Janeiro State), only 0.4% of patients who received various dosage regimens of parenteral $Sb^V$ treatment went on to develop ML after follow-up for 1 to 11 years [147].

In a study in which a low-dose regimen of parenteral $Sb^V$ was used and the follow-up period was 5–10 years, no cases of ML were identified among 120 patients whose cases of CL were successfully treated; and no cases of ML were reported among 59 patients treated with intralesional $Sb^V$ [147, 148]. In a study in Brazil, a 3.2% rate (2/62 persons) of development of ML posttreatment was reported [142].

The second type of study suggests that the majority of cases of ML occur in persons who did not receive optimal therapy for their prior cutaneous lesions. This type of study includes a series of 78 Brazilian patients with ML, only 7 of whom reported having a history of treatment of CL [27]; and a series of 12 Brazilian patients, 4 of whom had been treated previously (3 with parenteral $Sb^V$ and 1 with pentamidine) [149]. However, among imported ML cases in travelers, there are reports of some who had received prior systemic treatment [132].

There have been a few small studies of the third type. In a study in Colombia (*L.* [*V.*] *panamensis* and *L.* [*V.*] *braziliensis*), none of 66 subjects with CL treated with parenteral $Sb^V$, 2 of 55 treated with allopurinol (generally not effective for CL), and 1 of 46 who received placebo developed ML within 1 year [150]. In a study in Salvador, Brazil, 2 of 18 patients treated with allopurinol developed ML within 1 year, compared with none of 16 who received parenteral $Sb^V$ [151].

In aggregate, these observations suggest that effective systemic treatment of NWCL caused by *Viannia* species can decrease the risk for ML but may not prevent all cases of ML.

## XIII. What systemic treatment options are available in North America for CL, and what factors should be considered when selecting a medication for an individual patient?

*Recommendations*

35. The parenteral options for systemic therapy currently available in North America include conventional amphotericin B deoxycholate, lipid formulations of amphotericin B, pentavalent antimonial ($Sb^V$) compounds, and pentamidine (listed in alphabetical order). Oral options include miltefosine and the “azole” antifungal compounds, including ketoconazole (if potential benefits outweigh risks for hepatotoxicity and QT prolongation) and fluconazole [FACT, no grade].
36. To maximize effectiveness and to minimize toxicity, the choice of agent, dose, and duration of therapy should be individualized [Strong, moderate]. *Remarks*: *No ideal or universally applicable therapy for CL has been identified. Some therapies/regimens appear highly effective only against certain Leishmania species/strains in certain areas of the world. Both the parasite species and host factors (e.g., comorbid conditions and immunologic status) should be considered.*
37. Factors that should be considered when selecting CL treatment for an individual patient include the risk for ML; the *Leishmania* strain/species and published response rates for antileishmanial agents in the pertinent geographic region; the potential for adverse events; age extremes; childbearing competence and pregnancy; obesity; hepatic, pancreatic, renal, and cardiac comorbid conditions; preference for and convenience of various routes of administration; the rapidity with which one wishes to control the infection; the impact of lesions on daily activities and patient self-confidence; the patient/provider comfort level with logistics (e.g., Investigational New Drug protocols); and other practical issues (e.g., drug availability, various types of cost, insurance reimbursement) (see XII and XXVI; Tables 3 and 4) [Strong, low].

*Evidence Summary*

The options for systemic treatment of CL in North America are partially limited by availability issues (Table 3), which are discussed for each individual agent below. In the United States, using those drugs that are available only under Investigational New Drug (IND) protocols implies somewhat reduced flexibility in treatment administration and the need for IND-associated record keeping and review. In Canada, agents not approved for use are available through the Special Access Program from Health Canada; but physicians must make their own arrangements for importation, and permission is usually given only if other approved agents can be clearly shown to be ineffective, inferior, or not tolerated. Associated costs may not be covered by public or private insurance.

There is no ideal systemic treatment, in the sense of being uniformly and highly effective, regardless of the parasite species or the patient’s immunologic status; safe, regardless of the patient’s age, pregnancy status, or comorbidities; and inexpensive, short-course oral therapy. Each available regimen lacks several of these desirable features. Individual persons and physicians will weigh these factors differently. No therapy has been shown to eradicate all parasites, and the risk for relapse cannot be entirely eliminated.

The choice of systemic agents will depend on several factors. Species-directed therapy has received much attention. The relevant data have been reviewed [85], and most available clinical trials are summarized in the Appendix. In general, the mainstay for systemic treatment has been the pentavalent antimonials ($Sb^V$), the majority of the data on treatment relates to the use of these agents, and these agents have been the reference against which other agents have been compared. The antimonials have issues with toxicity and availability, which provide the motivation for using alternative regimens and agents.

The reasons for choosing systemic therapy are discussed in XII. Modification of the risk for ML is often an important consideration, but the only available data involve use of $Sb^V$; other agents may be considered when the risk for ML is thought to be low or if geographically restricted therapeutic failure with $Sb^V$ has been reported. It is difficult to accurately determine whether an infection is truly localized or who is at risk for dissemination. When systemic treatment is chosen for cosmetic or practical reasons, factors related to toxicity, cost, and convenience become more relevant.

Factors in addition to the *Leishmania* species and the geographic region in which the infection was acquired may negatively affect response rates and may account for some of the heterogeneity in the results of clinical trials. Factors that may adversely affect outcome include markers of severity, such as higher number of skin lesions and coinfection with *Leishmania* RNA virus 1 [152, 153] Factors that, paradoxically, may increase the risk for poor outcome include shorter duration of exposure in a CL-endemic region (<72 months),

earlier initiation of therapy for CL, and younger patient age [153]. Treatment early in the course of the disease, including treatment of adenitis before skin ulceration is evident, appears to be a risk factor for poor response [154]. One hypothesis is that some or all of these factors may be proxies for a less-effective immune response [155].

Therapeutic failure with particular antileishmanial agents has not been well studied in CL. Unusually high antimonial therapeutic failure rates have been noted in tourists returning from Amazonian Bolivia, generally *L.* (*V.*) *braziliensis* acquired in the frequently visited Madidi National Park [156]. Anecdotally, this observation has also been associated with *L.* (*V.*) *braziliensis* acquired in Manu National Park and Puerto Maldonado in southeastern Peru. Miltefosine appeared to perform less well against *L.* (*V.*) *braziliensis* in Guatemala compared with other countries [157]. Research techniques for monitoring drug resistance *in vitro* have been described; and surveillance of response rates in specific regions, together with monitoring of clinical risk factors associated with treatment success or failure, may become available in the future.

It is possible that some therapeutic failures are related to our limited understanding of drug pharmacodynamics and weight-based dosing. On the basis of pharmacokinetic considerations, we assume that amphotericin B deoxycholate doses should be based on total body weight, whereas pentavalent antimonial dosing is more complex and, in some cases, might be better guided by ideal body weight (see XXVI). Anecdotal experience with miltefosine suggests that gastrointestinal toxicity tends to limit the ability to give daily doses above 150 mg. As mentioned above, drug toxicities as listed in Table 4 become more important considerations among those with relevant comorbidities and in the elderly in general. Some antileishmanial drugs are not recommended during pregnancy or breastfeeding (see XXVI and Table 4).

Convenience and adherence are important in some cases. For example, amphotericin compounds are typically given with saline loading and premedication, and the entire process may take 4 hours for each dose. Antimonials can be infused over a shorter time but may require more doses. Use of oral agents presumes adherence, in contrast to parenteral doses administered by a health worker. In some settings, services for giving IV infusions may not be available on weekends; and some home care services will not administer IND agents.

The systemic antileishmanial agents available in North America are discussed below and in Tables 3 and 4:

$Sb^V$ compounds have been the mainstay of systemic antileishmanial treatment for ~7 decades. They have shown reasonably good efficacy against almost all *Leishmania* species in most geographic regions. However, no $Sb^V$ drug is approved for commercial use in the United States or Canada. In the United States, the $Sb^V$ compound sodium stibogluconate (SSG; Pentostam®; GlaxoSmithKline; 100 mg $Sb^V$/mL) is available to U.S.-licensed physicians under IND protocols—for civilians, through the CDC Drug Service; and, for military beneficiaries, through the U.S. Army Medical Materiel Development Activity. At the time of this writing, the IND protocols cover intravenous (IV) and intramuscular (IM) but not intralesional administration. In North America, the most common route of administration is IV (vs IM), in part because the volume per dose is high (e.g., 14 mL for a 70-kg patient treated with SSG). The traditional regimen for CL is 20 mg of $Sb^V$ per kg daily for 20 days; 10 days may suffice in some settings. In Canada, both meglumine antimoniate (Glucantime®; Sanofi; 81 mg $Sb^V$/mL) and SSG are available to licensed physicians through the Special Access Program from Health Canada, generally free of charge (Tables 3 and 4).

The oral agent miltefosine was approved by FDA in March 2014 for use in the United States for treatment of CL caused by three particular species, *L.* (*V.*) *braziliensis*, *L.* (*V.*) *panamensis*, and *L.* (*V.*) *guyanensis,* in persons ≥12 years of age who weigh ≥30 kg (see XXVI). However, the effectiveness of miltefosine even for infection caused by these species has been variable in clinical trials—most notably, the cure rates for *L.* (*V.*) *braziliensis* infection 6 months posttreatment have ranged from 33% (5 of 15 persons) in Guatemala to 80% (32 of 40 persons) in Bolivia [157]. The FDA-approved regimen of miltefosine is 2.5 mg/kg per day (maximum, 150 mg, in 3 divided doses), for 28 days. The upper limit of 150 mg per day was established because of poor gastrointestinal tolerability and applies to all persons who weigh ≥45 kg. Limited information is available about the use of miltefosine in heavier persons, particularly in those who weigh ≥75 kg (see XXVI). However, some data suggest that doses <2 mg/kg per day are associated with lower response rates [158]. Miltefosine is not approved for use in Canada, and permission to import the drug is not always granted.

Conventional amphotericin B deoxycholate traditionally has been used as rescue therapy for CL. Some data suggest that amphotericin B is likely to be highly and broadly effective against a wide range of species. Lipid formulations of amphotericin B generally are better tolerated than conventional amphotericin B and may be better tolerated than $Sb^V$ (especially in HIV-coinfected persons). Essentially no controlled clinical trials of amphotericin B formulations have been successfully completed for CL; standard dosage regimens have not been established. The anecdotal experience using lipid formulations of amphotericin B, which are targeted to the reticuloendothelial system, has been mixed. Reasonably good response rates in the range of ~83%–85% have been reported for IV liposomal amphotericin B (L-AmB) in several case series: for Bolivian *L.* (*V.*) *braziliensis* CL in 34 Israeli tourists who were treated with a total L-AmB dose of 18 mg/kg (6 doses of 3 mg/kg/day); for *L. tropica* CL in 13 Israeli patients who also were treated with a total of 18 mg/kg; and for CL caused by various Old and New World species among 19 U.S. military health care beneficiaries whose initial L-AmB course (for purposes of the efficacy analyses) entailed a median total dose of 21 mg/kg (maximum of 30 mg/kg) [156, 159, 160]. Despite the lack of randomized controlled clinical data, off-label use of L-AmB is appealing to some clinicians because the drug is readily available in North America and they may be familiar with its use.

Pentamidine has had a unique niche in the treatment of *L.* (*V.*) *guyanensis* infection, largely on the basis of studies of individuals with high response rates, although most are retrospective observations. A single randomized controlled trial showed outcomes similar to parenteral antimony [161]. Two clinical trials have been conducted for *L.* (*V.*) *braziliensis* infection: the first showed inferiority to antimony, and the second showed outcomes similar to antimony [162, 163]. A more recent study from Surinam showed a lower therapeutic success rate than in previous studies, leading to the

question of whether resistance to pentamidine could be emerging; however, this study was of low quality due to a high loss to follow-up rate [164]. Pentamidine showed some efficacy in treatment of *L.* (*V.*) *panamensis* infection in Colombia [165, 166]. Pentamidine, which is available as an isethionate salt, has been used in doses lower than those used to treat *Pneumocystis* pneumonia (its most common indication in North America) and, consequently, with less associated toxicity (Table 4).

The "azoles" ketoconazole and fluconazole have been used with mixed results, in various settings. For example: ketoconazole (adult regimen: 600 mg daily for 28 days) showed modest activity against *L. mexicana* and *L.* (*V.*) *panamensis* infection in small studies in Guatemala and Panama, respectively [167, 168]. Use of fluconazole (adult regimen: 200 mg daily for 6 weeks) for treatment of *L. major* infection in various countries in the Old World has been associated with mixed results [169, 170]. Preliminary data from Iran suggest that a higher daily dose (400 vs 200 mg) for 6 weeks might be more effective against *L. major* infection [171]. Preliminary, uncontrolled data from northeastern Brazil suggest that a regimen of 8 mg/kg daily for 4–6 weeks might be effective against *L.* (*V.*) *braziliensis* infection in that region, at least in the short term [172]. There is little evidence for the efficacy of itraconazole, essentially no clinical data are available for posaconazole or voriconazole, and data comparing the "azole" agents with each other are not available. Thus, while ketoconazole is relatively toxic (Table 4), it is unknown whether other agents in this class have similar antileishmanial efficacy. Additionally, although substantial toxicity issues with ketoconazole have been described, such as serious hepatotoxicity in persons without apparent risk factors and QT prolongation that can result in life-threatening ventricular arrhythmias, these risks should be weighed against the toxicity of other commonly used antileishmanial agents, such as amphotericin and antimonial drugs.

Comparative and some recent selected noncomparative clinical trials of various available therapies are summarized in the Appendix, together with a general assessment of the methodology and conduct of each study. Studies are classified according to species and geographic region. The reader can use the Appendix to extract the studies relevant to a particular species and region of acquisition, and to obtain information about the quality of the treatment data. The marked heterogeneity among the studies suggests that data from one species/region cannot be extrapolated to other species or regions.

## XIV. In which clinical settings can local therapy be used effectively in a person with CL?

*Recommendations*

38. Local therapy is preferred for treatment of OWCL lesions defined as clinically simple (Table 1) and may be useful for localized NWCL caused by *Leishmania* species not associated with increased risk for ML [Strong, moderate]. *Remark: Local therapy includes heat and cryotherapy, topical ointments/creams with paromomycin and other ingredients, intralesional injections of pentavalent antimonial drugs (±cryotherapy), and photodynamic or laser treatment.*
39. Eschar(s) overlying ulcers should be debrided before administration of local therapy and any secondary infection managed to maximize treatment effect [Strong, very low].

*Evidence Summary*

The overarching concept is that localized and limited CL (simple CL) should, in general, be treated initially with local therapies, which typically can suffice for healing, and are less toxic and less expensive than systemic therapies. See Table 1 for a definition of complex CL, which usually is not treated solely with local therapy. A current constraint in North America is access to and experience with local therapies, which are more in the practice scope of dermatologists than infectious disease clinicians.

Types of local therapy include physical treatments (e.g., heat, liquid nitrogen, photodynamic therapy, $CO_2$ laser), intralesional injection (pentavalent antimony), and topical ointments/creams (paromomycin preparations). Most of the published experience using local therapies has been with OWCL, particularly in *L. major*/*L. tropica*-endemic regions, with limited published experience with CL caused by *L. infantum* or *L. aethiopica*. In the New World, local therapies have been reported in few studies; species represented include *L. infantum-chagasi, L. mexicana, L. amazonensis, L.* (*V.*) *braziliensis, L.* (*V.*) *guyanensis,* and *L.* (*V.*) *naiffi*.

Patients for whom local therapy may be a good option include those who have small, few lesions of localized CL that do not involve the nose, ears, eyelids, lips, or genitalia; patients who are pregnant for whom systemic therapy may be contraindicated because of potential toxicity; and patients who may benefit from mop-up treatment after an incomplete response to systemic therapy. Combination therapy (e.g., local therapy plus an "azole" or cryotherapy plus intralesional $Sb^V$) may be considered for some patients. Therapeutic failure of initial local treatment can be managed with oral or parenteral systemic therapy. Local therapy usually is not recommended for treatment of CL if the risk for mucosal dissemination is considered substantial. This includes infection acquired south of Nicaragua with species in the *Viannia* subgenus. Local therapy is also not usually recommended for treatment of CL lesions with associated nodular lymphangitis or in immunocompromised hosts (Table 1).

Before initiating local therapy, lesions with overlying eschar should be debrided down to a clean ulcer base; and secondary bacterial infection (e.g., manifest by suppuration or surrounding cellulitis), if present, should be treated.

### Physical methods for CL treatment

Heat treatment: Dermatotropic species, such as *L. major, L. tropica,* and *L. mexicana*, are thermosensitive [173]. The therapeutic effect of heat generated in tissue using radiofrequency waves has been studied in randomized controlled trials with efficacy rates ranging from 54%–81% (see Appendix) [174–179]. Efficacy rates with two devices have been reported: the Ellman RF device (Ellman International, Inc., Hicksville NY); and, more commonly, with the ThermoMed™ device (Thermosurgery Technologies Inc, Phoenix AZ), which is FDA-cleared for treatment of CL. In brief, the protocol is to initially disinfect the lesion and surrounding skin (such as with an iodine preparation), provide local anesthesia with lidocaine, moisten with sterile saline, and apply the heat at 50°C for 30-second doses using the

device prongs to cover the lesion in a grid-like pattern extending 1–2 mm into surrounding normal-appearing skin. A second-degree burn can be anticipated.

The cosmetic outcome with heat therapy generally has been good, although with repeated treatment sessions more scarring may be seen. Most recipients report mild pain afterwards for a day, redness, oozing, and an eventual crusted eschar. Secondary bacterial infection can occur; use of topical antibiotics and dressing(s) for several days after a heat treatment is recommended. Good candidates for heat therapy include persons with uncomplicated CL, with smaller (≤25 mm width) and fewer lesions, not directly over superficial veins, nerves, or cartilaginous tissue. Areas where scarring is an issue, such as eyelids, nose, and the lips, should be avoided. Heat therapy can be used during pregnancy/breastfeeding and may also have a role as follow-up management for residual lesions not healing after systemic treatment.

Cryotherapy (or cryosurgery) may be more readily available than heat therapy. Various regimens of cryotherapy have been published. An example of an approach is to apply liquid nitrogen with a cotton-tipped applicator for 15–20 seconds, until 1–2 mm of the circumferential skin around the lesion appears frozen; then thaw for 20–60 seconds; then repeat the freeze step; and repeat the entire process at 3-week intervals until healing occurs [180]. In some studies, more frequent applications were administered. No anesthesia is used, which reportedly is not helpful.

Combination therapy with cryotherapy, immediately followed by intralesional antimony, seems to have the best efficacy.

In two controlled clinical trials in Iran, persons with CL were randomized to receive intralesional (IL) $Sb^V$, cryotherapy, or a combination of both therapies, which were administered every 1–2 weeks for 6–8 weeks [181, 182]. In these studies, the therapeutic success rates for the combined treatment arms were higher, 89% and 91%. The most responsive lesions tended to be those that were small (<30 mm width), on the face or neck, dry, and present for <3 months. When cryotherapy is combined with IL $Sb^V$, a shorter application of liquid nitrogen is used (e.g., no second freeze step), and the whitened skin is allowed to normalize in color before injection of the $Sb^V$ a few seconds later. On the basis of limited data, cryotherapy has been effective for infection caused by *L. aethiopica* in Ethiopia, *L. donovani* in Sri Lanka, and *L. infantum* in the Mediterranean region [182, 183].

Data from several large (thousands of persons), noncomparative case series provide perspective about the toxicity that can be associated with cryotherapy. Immediate reactions that can be seen in and near the treated skin include vesicle formation, erythema, swelling, and burning pain. Both hypo- and hyperpigmentation result; they typically are worse and more persistent in patients with darkly pigmented skin but generally have improved by 6–12 months of follow-up. Overall in the series, the scar has been described as acceptable, although keloids may form [184–186]. Cryotherapy should be considered in smaller, recent onset, uncomplicated CL lesions, including nonulcerative forms. It has been well tolerated on the face but avoidance of eyelids, lips, nose, and ears is recommended. It can be used during pregnancy and breastfeeding. It also may be an option for treatment of small residual lesions that persist after systemic therapy.

The success of both heat and cryotherapy is highly dependent on the skill of the operator and complete, careful application. It is important to treat into normal-appearing tissue around the lesions. Persons who have evidence of potential dissemination (e.g., subcutaneous nodules or regional adenopathy) and are infected with NWCL *Leishmania* species that can cause ML should not be treated solely with physical methods, which may not control the infection. Experience in OWCL suggests that small subcutaneous nodules may respond to local therapy of the primary lesion alone [187].

### Other

Photodynamic therapy and carbon dioxide laser treatment have been studied in randomized clinical trials with some promising early results [188–190].

### Intralesional pentavalent antimonial treatment

The $Sb^V$ compounds sodium stibogluconate and meglumine antimoniate have been used for intralesional treatment of CL (see above, the Appendix, and Table 3). Most of the published clinical trials have involved OWCL, although recently a few studies of NWCL have been performed. The intralesional method uses the undiluted parenteral formulation of these drugs but at much smaller doses (see below), which results in fewer systemic adverse effects and less expense. Laboratory monitoring is not needed. The procedure is painful: local anesthesia should be given in advance (such as with EMLA cream, i.e., lidocaine plus prilocaine), and young children may need general anesthesia. Intralesional $Sb^V$ injections should not be used on fingers, nose, ears, eyelids, near the lips, or anywhere vascular compromise could be of concern. In addition to pain, adverse effects can include local allergic reactions, pruritus, edema, and transient erythema.

In general, referral of patients to practitioners who have experience with intralesional administration is suggested when this treatment approach is considered. The aim is to inject the $Sb^V$ into the dermis, using a small gauge needle (25–27G); subcutaneous injection would be innocuous but ineffective. The volume injected is determined as a function of lesion size and varies from 0.2–5 mL in up to 4–5 injection sites, with a total estimated dose of about 0.1 mL per $cm^2$. The WHO recommends repeating injections every 3–7 days until healing occurs [42]. First cleanse the lesion and surrounding skin (e.g., with betadine or soap and water), then approach the lesion with the needle at a right angle initially and infiltrate in a V-shaped pattern, injecting drug under pressure as the needle is advanced [191]. Initially, the dermal injection may be met with resistance; this tends to decrease with subsequent sessions. The entire lesion and 1–2 mm of surrounding normal-appearing skin must be infiltrated until blanching is seen. When combination therapy with cryotherapy is used, the $Sb^V$ should be injected after liquid nitrogen (no preanesthesia is used) until blanching of lesion borders and swelling of the entire base are noted. As with the physical methods, the skill of the operator and careful controlled application are key to a successful outcome.

Most of the clinical trials of intralesional $Sb^V$ therapy have been conducted in the Old World, in Iran/Afghanistan, in studies in which the infecting species was not identified but the possibilities (depending on the region) included primarily *L. major* and *L. tropica*. Many regimens have been used, ranging from one injection 2 to 3 times per week to one injection every other week for 5–8 weeks; response rates have ranged from 25% to 100% [175, 192–195]. Only

a few studies of intralesional $Sb^V$ therapy for NWCL have been reported, generally because the risk for metastatic infection associated with *L.* (*V.*) *braziliensis* and other *Viannia* species has dissuaded use of local therapy. In a randomized clinical trial of single ulcer (≤30 mm largest diameter) Bolivian CL (predominantly *L.* [*V.*] *braziliensis*), intralesional $Sb^V$ dosed on days 1, 3, and 5 was compared with two doses of cryotherapy and with placebo cream. The reported efficacies of treatments at 3 months (with only 6 months of follow-up) were 70%, 20%, and 17% [196]. As per the prior cryotherapy section, studies combining cryotherapy with intralesional antimony showed an improved response in OWCL compared with each method individually [181, 197]. This synergistic response has also been noted for *L. infantum, L. mexicana, L. amazonensis*, and *L.* (*V.*) *naiffi* infection [85].

Topical paromomycin preparations

The only topical preparation with good supportive evidence for use in the treatment of CL is topical paromomycin. This aminoglycoside has been studied primarily in ulcerative infections caused by *L. major* in the Old World and by *L.* (*V.*) *panamensis* (small studies with other NWCL spp.) in Colombia and Panama [198–200]. Note that the vehicle seems cardinally important for efficacy, and different formulations cannot be considered equivalent. In a recent meta-analysis [201], the response to topical paromomycin therapy was higher if the formulation included methylbenzethonium chloride (MBCL), which itself induces a local inflammatory response. For *L. major* infection, the efficacy was equivalent to that for intralesional $Sb^V$; but, in NWCL, it was inferior to parenteral $Sb^V$ therapy. Topical agents may have better absorption in ulcerative (vs nodular) skin lesions, which may partially explain why the reported efficacy for *L. tropica* and *L. aethiopica* infection has been poor; *L. tropica* seems inherently less responsive (39% cure rate) than *L. major* to paromomycin/MBCL [202]. More recently, a third-generation topical paromomycin and gentamicin cream WR 279,396 (without MBCL) has been found in several phase II studies and a phase III clinical trial (n = 375 patients) to be associated with response rates of 81%–94% (compared with 58%–71% for vehicle placebo) using a 20-day course; twice and once daily applications have been associated with comparable response rates for treatment of *L. major* infection [203, 204].

None of these preparations are commercially available or FDA approved in the United States. WR279,396 is available under an expanded-access treatment protocol for U.S. military health care beneficiaries; contact usarmy.detrick.medcom-usammda.list.leishmania-tx-protocol@mail.mil for details. Leshcutan™ ointment, Teva Pharmaceuticals Israel, similar to the El On topical paromomycin formulation [194], has been available on international mail order sites such as www.pharmacyworld.com, although there may be FDA restrictions for U.S. physicians prescribing it. Oral paromomycin capsules are available, and a compounding pharmacy can provide a topical preparation (with unknown performance characteristics) using 15% paromomycin and 12% MBCL in soft white paraffin [198]. WR 279,396 cream can be approximated using the following formula: Unibase ointment with 15% (based on free base) paromomycin sulfate, 0.5% gentamicin sulfate, 6.75% urea, and purified water 42.2% (personal communication Dr. Max Grogl).

## XV. What are the recommended timeframes and findings to assess response to treatment in a person with CL?

*Recommendations*

40. Response to treatment is assessed by clinical criteria; repeat parasitologic testing is not recommended if the skin lesion appears to be healing [Strong, low]. *Remark: The healing process may continue after the treatment course is completed especially for large ulcerative lesions.*
41. Persons with CL should have their skin lesions monitored for 6–12 months after treatment for clinical evidence of therapeutic failure, which is initially seen at the border of a healed lesion [Strong, low]. *Remark: The first sign of healing is usually flattening of the skin lesion. By 4–6 weeks after treatment, the lesion size should have decreased by >50%, ulcerative lesions should be reepithelializing, and no new lesions should be appearing. Ulcerative lesions are generally fully reepithelialized and clinically healed by approximately 3 months after treatment*.

*Evidence Summary*

The responses of individual persons to treatment of CL are assessed clinically by the physical appearance of their lesion(s). Therapeutic success is usually defined as complete epithelialization of ulcerative lesions and lack of inflammatory findings/induration for nonulcerative lesions. Scarring is common but can improve with remodeling over months to years. Parasitologic assessment is used in the context of clinical trials; but it does not correlate well with clinical healing, which is the patient-relevant outcome [205–207]. During the first 2 weeks of therapy, there can be a paradoxical increase in the perilesional inflammatory response, including new satellite lesions and more erythema/induration especially around the border [208]. This exacerbation does not portend a poor treatment response but may be concerning to the patient and physician; with adequate treatment, it will usually resolve within 3–4 weeks.

Treatment response can vary based on many factors. CL caused by some *Leishmania* spp. often spontaneously heals within months (e.g., *L. mexicana, L. major*), whereas CL caused by *L. tropica* and *Viannia* spp. may be very slow to resolve. Other parameters that affect the response to treatment include host factors (e.g., age, immune status); lesion appearance (e.g., ulcerative vs not), location (e.g., lesions on the lower legs or on cartilaginous areas, such as ears or nose, may be slow to heal), and severity; bacterial superinfection (may interfere with healing); and the treatment and route of administration used [209]. Clinical trials show that tissue repair will take weeks after the parasite is killed; therefore, typically, healing is seen by 6–9 weeks after the start of treatment (faster with *L. major* and *L. mexicana*; slower with *Viannia* spp.). Especially for large ulcerative lesions, the healing process may continue after the treatment course has been completed. Five clinical parameters of healing have been suggested for assessing endpoints in treatment trials: the size of the area of ulceration (using the two largest diameter measurements), the size of the area of induration (consider using the ball-point pen method to determine), an estimate of the thickness of the induration, the color of the lesion border, and the extent of scarring [209, 210].

A general timeline for assessing the adequacy of the treatment response was developed by a WHO expert consensus group [209]. Usually one should expect some degree of improvement by 42–63 days after the start of treatment and clinical "cure" by 3 months [211]. Patients should be monitored for 6–12 months to assess for longer-term therapeutic failure or relapse (most occur within the first 3 months posttreatment). The first sign of healing is usually flattening (decreasing induration) of the skin lesion. Other physical findings associated with progressive healing include increasing reepithelialization of ulcerative lesions, decreasing lesion size, the presence of more granulation tissue, and decreasing erythema. In contrast, enlarging lesions or new lesions (e.g., satellite lesions) or subcutaneous nodules along the draining lymphatics may be indicators of a lack of response. Therapeutic failure often starts with breakdown along the border of previously epithelialized ulcers; increasing size, induration, and erythema are also suggestive of reactivation.

## XVI. What are the recommended approaches for additional management in a person with CL that does not respond to therapy?

*Recommendations*

42. Additional therapy is recommended (but not necessarily always with a different agent or approach) when there is development of new skin lesions or worsening of existing lesions. Additional therapy is also recommended if there is incomplete healing by 3 months after completion of the treatment course [Strong, low].
43. We recommend that therapeutic failure be assessed by physical appearance. Relatively little improvement or worsening while on therapy suggests an inadequate response and an alternate treatment approach should be planned [Strong, low]. *Remark: A paradoxical increase in the local inflammatory response may be seen in the first 2–3 weeks of treatment and can be difficult to differentiate from therapeutic failure.*
44. Consultation with a leishmaniasis expert about other treatment options is recommended for management of persons' lesions associated with therapeutic failure [Strong, very low].

*Evidence Summary*

Limited published data are available that can inform clinical management decisions when CL therapeutic failure occurs. The factors considered with the initial therapy decision continue to be important, and the treatment history may influence the retreatment choices. Drug availability, as well as the experience of the treating provider and the wishes of the patient, will influence the approach taken.

When therapeutic failure has occurred, confirmation of the diagnosis and species identification of the parasite (by culture and molecular diagnostic testing), if not previously done, are recommended. When scraping or aspiration of the lesion does not yield sufficient diagnostic information, obtaining a full-thickness biopsy specimen should be considered.

Local wound care should be continued and bacterial superinfection, if any, should be treated since it has been associated with lower response rates to $Sb^V$ therapy [212]. Changing the treatment should be considered, such as selecting a different local therapy, a different systemic therapy, or a systemic instead of a local therapy. To mitigate adverse effects, a washout period between therapies may be required (e.g., between $Sb^V$ and amphotericin B formulations). Retreatment with more of the same drug may also work. If CL progresses during therapy or if there is no response at all by 4–6 weeks posttreatment, retreating with the same drug may not be the best option. For some patients, combination therapy may be an option—e.g., with two antiparasitic agents, an antiparasitic plus a physical modality (such as with IL $Sb^V$ plus cryotherapy), or an antiparasitic plus an immune modulator. Sometimes, discontinuation of therapy and observation may be the optimal approach.

Despite relatively high therapeutic failure rates even in immunocompetent persons (~10%–25%), the possibility of immune deficiency should be considered if CL is rapidly progressive, unresponsive to various therapeutic modalities, or highly atypical in clinical manifestations. If the patient is immunocompromised or if the *Leishmania* species/strain is associated with ML, systemic therapy should be continued either alone or in combination with another agent/modality unless associated with substantial toxicity.

Predictors of therapeutic failure include host as well as parasite factors. The host's general immunologic status (especially regarding cell-mediated immunity) plays an important role in treatment response [213, 214]. Corticosteroids, both topical and systemic [215–219], have been associated with recurrence of CL; similarly, immune modulators, especially TNF-α antagonists (e.g., infliximab), have also been implicated in therapeutic failure (see XXV) [220].

Several case-control studies in Peru assessed risk factors associated with CL $Sb^V$ therapeutic failure. Depending on the report, these included concomitant distant lesions, the species *L.* (*V.*) *braziliensis*, young patient age, short duration of skin lesions, prior treatment, incomplete treatment, and weight >68 kg [154, 155, 221–223]. Local trauma was also associated with CL recurrence [5,219].

In addition to host factors described above, intrinsic and acquired parasite resistance to antileishmanials has been described with $Sb^V$ [224–233], miltefosine [234], amphotericin B (deoxycholate or liposomal) [217, 231, 233, 235, 236], and pentamidine [231] in treatment of Old and New World CL. Therapeutic failure has been associated with CL caused by the following species: *L.* (*V.*) *braziliensis* [228, 234, 237, 238], *L.* (*V.*) *panamensis* [227, 234], *L. tropica*, *L. major* [239], and *L. aethiopica* [240]. Therapeutic failure with local physical or other non-drug modalities (e.g., cryotherapy, heat therapy, photodynamic therapy) has been reported [233], but this cannot strictly be considered parasite resistance or even a host-driven therapeutic failure. Success or failure with some of these modalities may also depend, in part, on operator expertise.

Therapeutic decision-making for persons with relapsed CL or CL treatment failure, including leishmaniasis recidivans, can be challenging and frustrating. We recommend consultation with a leishmaniasis treatment expert regarding management options. The evidence for these recommendations derives from case reports and modest-sized case series. Therapeutic choices are often driven by the availability of particular treatment modalities and the practitioner's experience. The published reports must be interpreted in the context that CL is a self-resolving disease in immunocompetent hosts, in whom the course and prognosis vary greatly by species/strain, geographic location, and various host factors.

Summarized below are treatments that have been reported as successful for CL with therapeutic failure. In NWCL, repeating a course of antimonials [216]; and treating with immunomodulators, such as imiquimod, pentoxifylline, or granulocyte macrophage colony stimulating factor (GmCSF), together with repeat courses of antimonial drugs or alone, have been associated with efficacy [233, 241, 242]. Amphotericin/liposomal amphotericin B are regularly used in lieu of $Sb^V$, including in retreatment for therapeutic failure [160, 221]. *L.* (*V.*) *guyanensis* infection can be treated with a repeat course of pentamidine [219]. For therapeutic failure of OWCL, repeating antimonial therapy IL or systemically, adding or using imiquimod [220], changing to liposomal amphotericin B [159], using combination treatments such as allopurinol and $Sb^V$ [243], or extending the duration of/ repeating a liposomal amphotericin course [160] have been reported anecdotally as successful therapeutic strategies.

## MUCOSAL LEISHMANIASIS

### XVII. What are the treatment options for American (New World) mucosal leishmaniasis (ML)?

*Recommendations*

45. All persons with clinically manifest, metastatic, American ML should receive systemic antileishmanial therapy, with the goals of preventing morbidity (e.g., disfigurement) and mortality (e.g., from aspiration pneumonia or respiratory obstruction) [Strong, low].
46. Before treatment is initiated, a complete examination of the naso-oropharyngeal/laryngeal mucosa should be conducted by a specialist to assess the anatomic extension and clinical severity of the mucosal disease, which have prognostic implications [Strong, moderate].
47. We recommend inpatient monitoring and prophylactic corticosteroid therapy for persons with laryngeal/ pharyngeal disease and increased risk for respiratory obstruction, as indicated by symptoms and otolaryngologic/ radiologic examinations, because of the potential for inflammatory reactions after initiation of antileishmanial therapy [Strong, low].
48. The choice of antileishmanial agent, dose, and duration of therapy for persons with ML should be individualized (Table 3) [Strong, moderate]. *Remarks: The traditional options for ML include treatment with a pentavalent antimonial ($Sb^V$) compound (20 mg $Sb^V$/kg daily, IV or IM, for 28–30 days) or with amphotericin B deoxycholate (0.5–1.0 mg/kg per dose, IV, daily or every other day, for a cumulative total of ~20–45 mg/ kg). More recently, on the basis of comparatively limited data, the armamentarium has expanded to include lipid formulations of amphotericin B (typically, liposomal amphotericin B [L-AmB], with a cumulative total dose ranging widely from ~20–60 mg/kg), as well as the oral agent miltefosine (~2.5 mg/kg per day [maximum, 150 mg/day] for 28 days).*

*Evidence Summary*

Many of the principles regarding treatment of NWCL caused by *L.* (*V.*) *braziliensis* and related species in the *Viannia* subgenus are applicable to persons with ML. However, in comparison with CL, the stakes are higher for ML (because of the risks for substantial morbidity and for mortality); the management issues are more complex, compounded by immunologic and anatomic factors; fewer prospective clinical trials have been conducted and even fewer randomized clinical trials [244]; and the trials that have been conducted have, of necessity, been comparatively small [245].

Response rates—even with the same drug regimen—vary widely, depending in part on interrelated factors such as the *Leishmania* species/strain; the geographic region in which infection was acquired [246, 247]; the particular, as well as the number of, anatomic locations involved (e.g., nasal mucosa, palate, pharynx, larynx/epiglottis/vocal cords) [70, 248]; the severity of the site-specific symptoms and signs; the duration of the mucosal involvement; and poorly understood immunologic and other variables. As broad generalizations, ML, compared with CL caused by the same species/strain in the same setting, is less responsive to antileishmanial treatment and posttreatment relapse is more common. However, if ML is detected early and is confined to the nasal (or oral) mucosa, the response rates for ML may approach those for CL. The lowest cure rates generally have been associated with laryngeal disease, especially if the vocal cords are involved [42, 248]. Persons with laryngeal/pharyngeal disease also may be at risk for respiratory obstruction, including after initiation of antileishmanial therapy, which may trigger a Jarisch Herxheimer-type reaction [22, 23, 126, 249, 250]. At-risk persons should be closely monitored; and prophylactic corticosteroid therapy should be considered [22, 126], taking into account potential benefits and risks [251, 252]. The potential need for corticosteroid therapy and the dose and duration thereof (before and during antileishmanial therapy) to prevent or treat laryngeal/ pharyngeal edema/obstruction need to be individualized in consultation with the otolaryngologist who performed the ENT examination. For example, even short courses of corticosteroid therapy can be associated with development of life-threatening strongyloidiasis [253]; therefore, laboratory screening for asymptomatic *Strongyloides stercoralis* infection (and, potentially, empiric ivermectin therapy) may be warranted [254].

In Latin America, $Sb^V$ compounds and amphotericin B deoxycholate generally are the most readily available and commonly used drugs for treatment of ML [246, 255]. Selected information and perspective about these and other options are provided below; the drugs are not necessarily discussed in order of preference for persons in North America.

In a *L.* (*V.*) *panamensis*-endemic area of Panama, a 28-day course of therapy with IV sodium stibogluconate (SSG; 20 mg $Sb^V$/kg/day) was evaluated in a prospective, noncomparative clinical trial among 16 persons with disease confined to the nasal mucosa [256]. At the last follow-up examination, up to 1 year posttherapy, 10 persons (63%) were classified as cured, all of whom had received a 28-day treatment course.

In a prospective clinical trial in Peru (*L.* [*V.*] *braziliensis* infection), using the same 28-day SSG regimen, six (75%) of eight persons whose ulcerative/infiltrative disease was confined to the nasal mucosa had sustained clinical cures, without relapse during the 12-month posttreatment follow-up period [257]. However, in the same study, only two (10%) of 21 persons whose disease involved at least one additional anatomic location (e.g., pharynx or larynx) were classified as cured. To assess whether a longer course of $Sb^V$

therapy would increase the cure rate for Peruvian persons with “multi-anatomic” ML, the same group of investigators randomized such persons to receive either 28 or 40 days of IV SSG (20 mg $Sb^V$/kg/day) [258]. The per-protocol cure rate was 63% in both treatment groups—i.e., 10 (63%) of 16 persons treated for 28 days and 12 (63%) of 19 persons treated for 40 days had sustained clinical cures, without relapse during the 12-month follow-up period. The investigators did not have an explanation for the much higher cure rate in the second compared with the first study (63% vs 10%) among persons with multi-anatomic disease.

Adjunctive therapy with pentoxifylline, which inhibits the production of TNF-α by mononuclear cells and modulates the immune response [259], has looked promising in clinical trials in Brazil [229, 230, 245]. In a double-blind, placebo-controlled clinical trial, conducted in a *L.* (*V.*) *braziliensis*-endemic area (Bahia State) among persons with nasal ML, all 11 persons who received a 30-day course of combination therapy with parenteral $Sb^V$ (20 mg/kg/day) plus oral pentoxifylline (400 mg thrice daily) were classified as cured, without relapse during ~2 years of follow-up, compared with seven (58%) of 12 persons who received $Sb^V$ therapy plus placebo [229]. The investigators previously had conducted an open-label study among persons with $Sb^V$-refractory ML [230]; nine of the 10 enrolled persons treated with $Sb^V$ plus pentoxifylline fulfilled the criteria for cure after 1 year of follow-up.

Amphotericin B deoxycholate has been the traditional alternative to $Sb^V$ therapy [260]; because it generally is considered more toxic (with a higher risk for irreversible toxicity), in some settings in Latin America, it has been used primarily for persons who did not respond to $Sb^V$ therapy [22]. In a randomized clinical trial in Bolivia and Peru, combination therapy with amphotericin B deoxycholate plus itraconazole was not better than monotherapy with amphotericin B [261]. In a prospective case series among Bolivian persons with ML of variable severity treated with amphotericin B deoxycholate (1 mg/kg every other day for a total of 45 doses), seven (50%) of 14 evaluable subjects and seven (37%) of 19 total persons were classified as cured [262].

Limited, uncontrolled data from case reports/series have been published regarding therapy with lipid formulations of amphotericin B, most often with L-AmB [126, 127, 131, 214, 263–268]. In two relatively large retrospective studies (16 and 29 patients, respectively) conducted by different groups of investigators in São Paulo, Brazil [263, 268], the results regarding L-AmB treatment of ML were promising, with the caveat that the studies were subject to the many limitations inherent to uncontrolled studies in which data are obtained by retrospective review of medical records.

Limited data regarding miltefosine therapy for ML have been published [262, 269, 270], the bulk of which are from two nonrandomized clinical trials conducted in a *L.* (*V.*) *braziliensis*-endemic region of Bolivia [262, 269]. In the studies in Bolivia, the enrolled adult subjects received a daily dose of either 100 or 150 mg. The mean weights of the persons in both studies were <60 kg, whereas North American adults typically are much heavier (see XXVI). In the first study [262], the duration of miltefosine therapy was 28 days. According to the published report [262], 78 persons were enrolled and 51 (71%) of the 72 persons considered evaluable were classified as cured after 12 months of follow-up, including 21 (58%) of 36 persons with palatal, pharyngeal, or laryngeal disease. In modified analyses [271], the intention-to-treat cure rate was 62% (49 of 79) and the per-protocol cure rate was 64.5% (49 of 76). In the second study [269], 21 persons not previously treated with miltefosine were treated for 6 weeks. Compared with persons in the first study, these 21 persons had had mucosal disease for a much shorter period (means of 5 years vs 14 months, respectively). Fifteen persons (71% of 21) fulfilled the criteria for cure. In addition, 17 of the persons from the first study who had been treated for 4 weeks and had not been classified as cured were located and retreated for 6 weeks, 11 (65%) of whom were considered cured thereafter. The investigators also assessed whether a longer follow-up period (i.e., 24 vs 12 months) could increase the relapse rate. Among the 45 persons classified as cured during the first study who were located and reevaluated, three (7%) definite or probable relapses were identified.

Limited published data are available regarding treatment of ML with pentamidine isethionate [214], an alternative, second-line agent [42]. Monotherapy with the parenteral formulation of paromomycin (not available in North America) is not recommended, on the basis of clinical trials in Peru and Brazil [272, 273].

Response to antileishmanial treatment of ML typically is assessed by clinical criteria. The majority of relapses occur within the first year, but they may occur later [22, 142, 269]. The risk factors for relapse are poorly understood [22, 274]. In persons whose mucosal disease improved or healed during or after the initial treatment course, relapses do not necessarily mean drug failure (drug resistance). In some settings, reinduction therapy with the agent initially used may be justified. Alternatives to consider for some persons/settings include monotherapy with a different medication or, potentially, combination therapy, such as with pentoxifylline.

## VISCERAL LEISHMANIASIS

### XVIII. In what circumstances should a person with visceral *Leishmania* infection be treated?

*Recommendations*

49. We recommend that persons with clinical abnormalities compatible with VL and laboratory evidence of VL be treated (Table 3) [Strong, moderate].
50. We suggest that clinicians closely monitor persons with asymptomatic visceral infection and generally initiate therapy only if clinical manifestations of VL develop [Weak, very low].

*Evidence Summary*

All persons with symptomatic visceral infection (i.e., VL) should be treated with antileishmanial drugs and ancillary measures. If untreated, advanced cases of VL can result in death, in association with progressive wasting, superinfection, or hemorrhage. Ancillary care includes nutritional support, treatment of other infectious diseases (e.g., tuberculosis, malaria, or bacterial or parasitic dysentery), and blood transfusions as needed. Persons newly diagnosed with VL should also be assessed for concurrent HIV/AIDS or other causes of cell-mediated immunosuppression.

The majority of asymptomatic *L. donovani* and *L. infantum-chagasi* infections in immunocompetent persons in VL-endemic areas are self-resolving, but prospective studies are lacking on how to manage them. Among a cohort of 32,529 persons monitored in *L. donovani*-endemic areas of India and Nepal, risk for progression to VL disease was associated with high serologic titer values and seroconversion during the study period [275]. If the person can be monitored closely, understands the situation, and is comfortable with delaying therapy, it is reasonable to follow and initiate therapy if signs and symptoms of VL develop. Quantitative measures of parasite load (e.g., qPCR) may eventually prove useful in predicting who will progress to symptomatic VL, but qPCR has not been adequately studied in immunocompetent asymptomatic persons and a standardized qPCR assay is not currently available in North America (see VII).

### XIX. What is the optimal treatment for VL in a symptomatic immunocompetent person (person without an identified immune defect) in North America?

*Recommendations*

51. For an immunocompetent person with VL, treatment with liposomal amphotericin B (L-AmB) is recommended. The FDA-approved dosage regimen is 3 mg/kg/day IV on days 1–5, 14, and 21 (total dose, 21 mg/kg) (Table 3) [Strong, high]. *Remarks*: *Multiple regimens in which the total L-AmB dose is 18–21 mg/kg have been used effectively in regions other than East Africa. Doses of 40 mg/kg or more may be necessary in persons with VL acquired in East Africa. Other lipid-associated formulations of amphotericin B, such as amphotericin B lipid complex and amphotericin B colloidal dispersion, are not generally recommended: they have not been approved by FDA for treatment of VL; and they have been less well studied in VL treatment trials (i.e., bioequivalence has not been established).*
52. For an immunocompetent person with VL caused by *L. donovani*, acquired in the Indian subcontinent (South Asia), who is >12 years of age, weighs >30 kg, and is not pregnant or breastfeeding, treatment with the oral agent miltefosine, 2.5 mg/kg per day (maximum, 150 mg, in 3 divided doses) for 28 days, is a possible alternative to L-AmB, particularly in persons weighing <75 kg (see XXVI and Table 3) [Strong, moderate].

*Evidence Summary*

The studies that served as the basis for the group's treatment recommendations have been summarized in review articles [276, 277] and in the FDA Briefing Document for the Anti-Infective Drugs Advisory Committee Meeting on the use of Miltefosine (Impavido®) for the Treatment of Visceral, Mucosal and Cutaneous Leishmaniasis [271].

For persons with no identifiable immune defect who have VL caused by either *L. donovani* or *L. infantum-chagasi*, we recommend treatment with L-AmB using the FDA-approved regimen. For many years, prolonged courses of amphotericin B deoxycholate were known to be effective VL treatment in India and elsewhere, but the drug's use was associated with substantial nephrotoxicity and other untoward effects (Table 4). Amphotericin B interacts with ergosterol precursors in *Leishmania*, disrupting the parasite plasma membrane [278]. Lipid formulations of amphotericin B couple liposome protection against renal and other toxic effects of amphotericin with deep tissue penetration into *Leishmania*-infected macrophages [279]. Therapeutic levels of L-AmB persist for ≥2 weeks in the liver/spleen after loading doses [280–282].

Liposomal amphotericin B (AmBisome®) was approved by FDA in 1997 for treatment of VL, on the basis of review of Mediterranean *L. infantum* dose-ranging, open-label treatment studies and case series, comprising 65% pediatric cases [283,284]. The FDA analysis found that the response rates among persons who received a total dose of >21 mg/kg or of 18 mg/kg were 100% and 97%, respectively. Data from a large case series of *L. donovani*-infected persons treated in a very rudimentary Sudanese field setting were also provided [285]. The regimen of L-AmB that the FDA approved for treatment of VL in immunocompetent persons (3 mg/kg/day on days 1–5, 14, and 21; total dose of 21 mg/kg) does not appear to have been used in any of the studies that were reviewed, but it has been shown to be effective in subsequent studies [276, 277]. A number of different dosage regimens have been studied, including regimens with shorter durations of therapy (e.g., even just a single infusion) and various total L-AmB doses; the FDA-approved regimen is recommended for persons treated in North America (Table 3).

There are geographic differences in VL treatment responses to L-AmB. In India, susceptibility to L-AmB has been observed in large, well-conducted randomized clinical trials demonstrating high therapeutic success rates among persons with parasitologically confirmed VL (*L. donovani*). A total L-AmB dose of 10 mg/kg administered over 5 days or given as a single infusion has been associated with an efficacy rate of 96% [277]. In a large cohort study in Bihar, India, L-AmB 5 mg/kg daily for 4 doses over 4–10 days resulted in a therapeutic success rate of 98% [286]. In the case of Mediterranean VL caused by *L. infantum*, L-AmB in a total dose of 18 mg/kg, administered in 6 doses over 10 days, also had an efficacy of 98% [287]. In another study, a pediatric regimen of L-AmB 10 mg/kg/day for 2 days was compared with a historical control group treated with meglumine antimoniate (20 mg $Sb^V$/kg/day for 30 days). The short course of L-AmB was at least as effective as $Sb^V$ treatment (97.6% vs 90%) [288]. L-AmB has also been used successfully to treat VL caused by *L. infantum-chagasi* in Latin America, but the published data are limited [289].

These results contrast with the treatment of VL caused by *L. donovani* in East Africa in which the therapeutic success rate at 6 months was 85% using the FDA-recommended 7-dose regimen (total dose 21 mg/kg) of L-AmB, but significantly lower with single-dose therapy of either 7.5 mg/kg (40%) or 10 mg/kg (58%) [290]. Furthermore, in eastern Sudan, L-AmB at a total dose of 30 mg/kg over 10 days resulted in an initial therapeutic success rate of 94% with 7% therapeutic failure [291]. In Uganda and Kenya, therapeutic failure was reported to be more common in persons with VL who received a total dose of L-AmB of 20 mg/kg (13%) than in those treated with a total dose of 30 mg/kg (3.9%) [292]. Finally, in an open trial of L-AmB to treat persons in Sudan who previously had an incomplete

parasitologic response or had relapsed after treatment with pentavalent antimony and aminosidine or had severe disease, a treatment regimen of 3–5 mg/kg on days 0, 3, and 10 cured 50% of 16 persons and 6 doses of 3–5 mg/kg on days 0, 3, 6, 8, 10, and 13 cured 88% of 16 persons [285].

Other lipid-associated formulations of amphotericin B, such as amphotericin B lipid complex (ABLC) and amphotericin B colloidal dispersion (ABCD), have different pharmacokinetic and toxicity profiles than L-AmB and have been less well-studied in VL treatment trials [277, 293–299]. In a randomized clinical trial among 153 persons with Indian VL, the therapeutic success rates with amphotericin B deoxycholate (1 mg/kg every other day for 15 doses), L-AmB (2 mg/kg/day for 5 days), and ABLC (2 mg/kg/day for 5 days) were 96%, 96%, and 92%, respectively; the study was not powered to show differences in efficacy. L-AmB was associated with the fewest adverse reactions per person (mean 0.6 ± 0.1): there were 14-fold more with amphotericin B deoxycholate, and >2 fold more in the ABLC group [300]. Finally, amphotericin B deoxycholate at 1 mg/kg dosing either every other day for 30 days or 15 days consecutively was 95%–100% effective for treatment of Indian VL; but there is substantially more renal toxicity, hypokalemia, and infusion-related toxicity than with L-AmB [300–306] (Table 4).

L-AmB has associated adverse events that may prompt selection of alternative therapy. CARPA (complement activation related pseudoallergy), caused by C3a/C5a anaphylaxitoxins triggering mast cells and basophils, yields hypersensitivity reactions that are not mediated by IgE [307]. Acute infusion reactions (occurring in the first 5 minutes and responsive to diphenhydramine) can include chest pain; hypoxia; dyspnea; severe leg, flank, or abdominal pain; and flushing with urticaria [308]. ABLC was used to treat fungal infections without severe adverse reactions in 34 of 40 persons who previously had experienced severe intolerance of L-AmB [309]. Although ABLC may be useful for treatment of selected cases of VL, its use in this setting is less well documented than that of L-AmB; a rough conversion factor is that 3 mg/kg L-AmB is similar to 5 mg/kg of ABLC. Even though, in comparison with amphotericin B deoxycholate, L-AmB is associated with less nephrotoxicity, 15% of persons treated with L-AmB have a two-fold increase in their baseline serum creatinine levels (AmBisome® package insert) (Table 4).

For persons with VL caused by *L. donovani* acquired in the Indian subcontinent who are at least 12 years of age, weigh at least 30 kg, and are not pregnant or breastfeeding, treatment with the oral agent miltefosine is an option (see XXVI and Table 3). Large randomized clinical trials in India suggest that miltefosine is effective against *L. donovani* infection, with cure rates of 82%–97% [310–318]. The response rate may be lower for VL caused by *L. donovani* infection acquired in East Africa. In a clinical trial in Ethiopia among HIV-uninfected persons with VL, the 6-month therapeutic success rate was 75.6% [319]. Evidence is sparse to support the use of miltefosine for VL caused by *L. infantum-chagasi* [320]. Miltefosine has frequent gastrointestinal side effects including transient vomiting and diarrhea; it is also teratogenic and requires birth control measures in women of child-bearing age, and it is associated with hepatotoxicity and rarely nephrotoxicity (see XXVI and Table 4). Development of miltefosine resistance has been a concern in India. The miltefosine $IC_{90}$ has been reported to be significantly higher in geographic areas where it had been used extensively in comparison with areas where its use had been limited [321]. In addition, the posttreatment $IC_{50}$ has been significantly higher than the pretreatment $IC_{50}$ in persons who have had a relapse [322]. Finally, decreased therapeutic success rates and increased failure rates have been observed in India after a decade of miltefosine use and increasing rates of therapeutic failure have been reported in Nepal [323, 324].

**XX. What alternative agent(s) can be used for a person with VL who cannot tolerate liposomal amphotericin B or miltefosine or in whom these agents otherwise are contraindicated?**

*Recommendations*

53. Pentavalent antimonial therapy (20 mg $Sb^V$/kg/day IV or IM for 28 days) is a recommended therapy for immunocompetent persons with VL acquired in areas where the prevalence of antimony-resistant *Leishmania* species is low (<10%) [Strong, high].
54. We do not recommend switching to amphotericin B deoxycholate in persons with contraindications to, or substantial toxicity with, L-AmB, with the exception of persons who develop liposome-induced complement activation-related pseudoallergy (CARPA). Amphotericin B lipid complex is a consideration in this situation [Strong, low].

*Evidence Summary*

The availability, use, and toxicities of the pentavalent antimonials sodium stibogluconate and meglumine antimoniate are discussed in reference to the treatment of CL in clinical question XIII and summarized in Tables 3 and 4. Sodium stibogluconate 20 mg $Sb^V$/kg/day IV or IM for 30 consecutive days (28 days in some *L. infantum-chagasi* studies) or meglumine antimoniate 20 mg $Sb^V$/kg/day were the first-line drugs for the treatment of VL for many decades. Reports of therapeutic failures (especially in Northeast India, Bangladesh, Nepal, and Bhutan) [316, 325 ] and concerns about toxicity have led to the emergence of L-AmB and miltefosine as first-line drugs for VL, but $Sb^V$ remains an option in some areas. Studies in East Africa, Brazil, and Greece suggest that the efficacy with $Sb^V$ remains in excess of 90%–95% for both *L. infantum-chagasi* and *L. donovani* infection [288, 326–328].

Pentamidine isethionate has been used to treat VL: in one study, 4 mg/kg IM given for 20 alternate day doses had an efficacy of 77% compared with 98% for amphotericin B deoxycholate [329]. Pentamidine toxicity has been substantial and has included sudden death (because of QT-prolongation effect), delayed symptomatic hypoglycemia, hypotension, allergic reactions, and insulin-dependent diabetes [330] (Table 4).

On the basis of experience in the treatment of fungal infections, we do not recommend switching to amphotericin B deoxycholate for persons with contraindications to or substantial toxicity with L-AmB. Amphotericin B deoxycholate is almost always associated with more toxicity. Other drugs

have been used for the treatment of VL, at times in multidrug regimens. There are published case reports and a small clinical series with imidazole antifungals (fluconazole or itraconazole) with/without allopurinol in persons with VL: the available data are insufficient to recommend their use [331, 332]. Parenteral paromomycin has looked promising in clinical trials in India, but it is not available in North America.

### XXI. In persons with VL, what parameters should be used to assess the clinical response to treatment?

*Recommendations*

55. Clinical parameters correlate well with parasitologic responses to VL treatment and should be used to monitor the response [Strong, low].
56. Parasitologic confirmation of response (such as by repeat bone marrow aspiration for microscopy and culture after treatment) is not recommended in a patient showing a timely clinical response. Antibody levels fall but over many months or longer [Strong, moderate].

*Evidence Summary*

Clinical parameters correlate well with parasitologic responses to VL treatment. Normalization of temperature; decreased liver and spleen size; rises in leukocytes, hemoglobin, and platelet values; and increasing appetite and weight suggest a clinical response [333, 334]. With effective treatment, fever typically abates in <1 week [288, 335, 336]. Organomegaly is slower to resolve (~3–6 months), but some decrease may be seen by 10 days after initiation of treatment, depending in part on whether physical examination or ultrasonography is used for assessment [288, 337]. Leukopenia and thrombocytopenia generally normalize within a month, but resolution of anemia may be slower (6–12 months) [287–289, 334, 338]. Therapeutic failure (return of clinical signs/symptoms in concert with parasitologic confirmation) can occur at least up to 12 months after treatment (often the longest duration of follow-up in clinical trials), but most therapeutic failure occurs within the first 6 months [287, 289, 337, 339–343]. Risk factors associated with death (often studied in VL populations with substantial comorbidities quite different than in North America) include severe anemia, prolonged illness, jaundice, malnutrition, age <1 year, concomitant infection, mucosal bleeding, gastrointestinal symptoms, neutrophils <500 cells/uL, and platelets <50,000 cells/uL [317, 344–347]. A prognostic scoring system for children with VL has shown a sensitivity of 88.7% and a specificity of 78.5% [347].

Parasitologic reassessment (such as by repeat bone marrow aspiration) typically is not needed in persons who have a timely clinical response and usually is reserved for subjects in clinical trials for definitive outcome information and for individual persons who remain symptomatic. Molecular analyses using semi-quantitative and real-time PCR assays show rapid clearance of *Leishmania* DNA from the peripheral blood during effective VL treatment of immunocompetent persons; but these tests are not standardized (various techniques, parasite targets, sensitivities), are not routinely available for clinical use in North America, and are not FDA approved [90, 348–353]. The results of serologic testing using the rK39 immunochromatographic assay (Kalazar Detect™, InBios) may remain reactive for >1 year after successful treatment, although early studies suggested that the ELISA (vs dipstick) format showed declining rK39 levels after treatment [354–356].

Post kala-azar dermal leishmaniasis (PKDL) has been identified during or after treatment of *L. donovani* VL in up to 10%–27.5% of patients in India and 1.5%–60% VL in Sudan; it uncommonly has been associated with *L. infantum-chagasi* VL in otherwise immunocompetent patients [357–361]. The skin lesions can include papules, nodules, and sometimes hypopigmented macules, which may coalesce into irregular patches. There is a predilection for the face, around the nose and mouth; but PKDL can extend to the trunk, arms, and legs. The skin of biopsied papular lesions is infiltrated with amastigotes, and persons with PKDL are a likely reservoir for VL infection and a source for sand fly infection in VL-endemic regions, perpetuating transmission.

### XXII. How should a person with VL be treated who does not respond to initial therapy as assessed by these parameters [or who has a relapse]?

*Recommendations*

57. Immunocompetent persons with VL who do not respond to therapy with L-AmB should be treated with an alternative drug or with a higher dose or a longer course of L-AmB [Strong, low].
58. Immunocompetent persons with VL who do not respond to initial therapy with miltefosine or a pentavalent antimonial compound should be treated with L-AmB or an alternative drug if L-AmB is unavailable [Strong, low].
59. Immunocompetent persons with VL who respond to initial therapy but subsequently have a relapse should be treated with an alternative drug or with another, potentially longer, course of therapy with the initial drug. If L-AmB was the drug used for initial therapy, use of a higher dose can be considered [Strong, low].
60. Combination therapies may be considered but have not been well studied after therapeutic failure in persons with VL [Weak, low].

*Evidence Summary*

There are insufficient data to formulate an evidence-based recommendation for a therapeutic regimen for those who initially fail to respond. They can be treated with an alternative drug, the same drug at higher doses or for longer periods, or a combination of drugs. The choice of drug (s) is based on the infecting *Leishmania* species, the prevalence of therapeutic failure in the geographic area of acquisition, the immune status of the host, and potential untoward effects of the regimen.

Therapeutic failure can occur in persons without documented immunodeficiency, most are within 6 to 12 months, but it is more likely in those with AIDS or compromised cell-mediated immunity for other reasons and often represent an immunologic failure rather than a drug failure (see XXIII and XXV).

## LEISHMANIASIS IN IMMUNOCOMPROMISED HOSTS

### XXIII. How should HIV/AIDS-associated visceral leishmaniasis (VL) be treated in persons in North America, and what other management issues should be considered?

*Recommendations*

61. Liposomal amphotericin B (L-AmB) is recommended for the treatment of VL in immunocompromised persons in North America (Table 3) [Strong, low]. *Remark: The FDA-approved dosage regimen of L-AmB for such persons, including those with concurrent HIV/AIDS, is 4 mg/kg/day IV, on days 1–5, 10, 17, 24, 31, and 38 (10 doses over a 38-day period), for a total dose of 40 mg/kg.*
62. Combination therapy (e.g., L-AmB plus miltefosine) might be considered, especially for persons with refractory cases of VL [Weak, very low]. *Remark: The efficacy and optimal duration of miltefosine monotherapy (and combination therapy) for HIV/AIDS-associated VL have not been established.*
63. Because of the importance of effective immune reconstitution in HIV-VL-coinfected persons, antiretroviral therapy (ART) should be initiated or optimized as soon as the person is sufficiently stable to tolerate it (e.g., either during or soon after the initial course of therapy for VL) [Strong, low].
64. *Leishmania* infection that becomes clinically manifest or worsens after initiation of ART should be treated with antileishmanial (and, if indicated, corticosteroid) therapy; leishmaniasis-associated immune reconstitution inflammatory syndrome (IRIS) reactions after initiation of ART have been reported occasionally [Strong, very low].
65. We recommend administering secondary prophylaxis (chronic maintenance therapy) to decrease the risk for posttreatment relapse of VL in persons with HIV/AIDS-associated immunosuppression (e.g., CD4 T-lymphocyte cell counts <200 cells/mm$^3$) [Strong, moderate].
66. Persons with VL-HIV/AIDS coinfection should be monitored indefinitely (until effective immune reconstitution) for evidence of posttreatment relapse; ART and secondary prophylaxis provide only partial protection against relapse. Antileishmanial treatment is indicated for persons who have clinical and parasitologic evidence of recurrence [Strong, low].

*Evidence Summary*

Nonsterile cure of *Leishmania* infection is considered the norm, even for immunocompetent persons. Residual parasites may be present indefinitely in tissue macrophages, typically held in check by T cell (predominantly, CD4 cell)–dependent immunoinflammatory responses, including Th1-type cytokine-mediated activation of macrophages [362]. However, HIV infection increases the risk for development and recurrence of clinically manifest VL; even visceral infection that was quiescent for years to decades may (re)activate in the context of immunosuppression. In HIV-infected persons, the first diagnosed episode of VL typically represents (re)activation of latent infection and subsequent episodes usually constitute posttreatment relapses. However, in leishmaniasis-endemic areas, newly acquired (re)infections may account for some VL episodes [363]. When VL initially is diagnosed in HIV-infected persons, the CD4 cell count typically is <200 cells/mm$^3$ (often, <100 cells/mm$^3$) [34, 36, 39, 42, 364]. The presence of other coinfections/comorbidities may complicate clinical management.

Many of the principles regarding treatment of VL in immuncompetent persons are applicable to persons with concurrent HIV/AIDS. However, the management issues are more complex, the evidence base is weaker, and the response to antileishmanial therapy (and ART) is suboptimal. Coinfected (vs HIV-uninfected) persons are less apt to respond to the initial treatment course, and posttreatment relapses are much more common.

L-AmB generally is the drug of choice for treatment of VL in North America, regardless of whether the patient is immunocompetent or immunocompromised. However, the FDA-approved dosage regimen of L-AmB for immunocompromised persons entails a higher daily dose (4 vs 3 mg/kg), number of doses (10 vs 7), and total dose (40 vs 21 mg/kg) [284]. The optimal L-AmB regimen for inducing clinical remission has not been established and undoubtedly varies by host as well as parasite factors, such as in different geographic regions [365–368]. Regimens including L-AmB (or, less commonly, other lipid formulations of amphotericin B)—on consecutive days or in an interrupted schedule (such as in the FDA-approved regimen)—to achieve a total cumulative dose of ~40 mg/kg (range, 20–60 mg/kg) have been used with variable response rates [42, 362, 365–372].

The particular L-AmB regimen that FDA approved in 1997 for immunosuppressed persons was selected on the basis of analysis of limited, pooled data from three nonrandomized clinical studies conducted in Europe during the pre-ART era [283, 287, 370]. The studies included 21 immunosuppressed persons, 17 of whom were infected with HIV. The first 10 of the 19 immunosuppressed persons who fulfilled FDA's inclusionary criteria [284] were treated with 100 mg of L-AmB daily for 21 days (total dose, 2.1 grams; 29–38.9 mg/kg). All 10 persons had clinical and parasitologic responses to therapy; however, eight persons (80%) experienced relapses during the follow-up period. The other nine evaluable persons [370], all of whom were infected with HIV, were treated with the dosing schedule that FDA ultimately approved (total dose, 40 mg/kg, with intermittent rather than daily dosing). All nine persons clinically improved, eight of whom had parasitologic responses (no parasites evident by light-microscopic examination or culture of bone marrow aspirates), one of whom defaulted during the follow-up period, and all of the other seven persons experienced relapses 2–7 months posttreatment. Only one patient was still alive 26 months posttreatment. Among the other six persons, the mean interval from the initial diagnosis of VL to death was 19 months (range, 5–40 months).

Evidence from a systematic review of data regarding therapy of HIV-associated VL—which were analyzed with indirect comparisons, constrained by the limitations and heterogeneity of the available data, including the paucity of randomized clinical trials—has underscored that treatment with a amphotericin B formulation (e.g., L-AmB) is “superior”

to pentavalent antimonial ($Sb^V$) therapy [373], in part because of the safety issues associated with $Sb^V$ treatment in coinfected persons (e.g., potentially life-threatening clinical pancreatitis and cardiotoxicity [319, 371, 374–377]). Amphotericin B deoxycholate also generally is relegated to second-line status, in the context of L-AmB's better safety profile. If a coinfected patient in North America needed to be treated with amphotericin B deoxycholate or a $Sb^V$ compound, standard dosage regimens for VL generally would be used; data regarding optimal regimens for coinfected persons are not available.

Limited data regarding treatment of HIV-associated VL with the oral agent miltefosine have been published [319, 378–380]. In a randomized clinical trial in a population in Ethiopia with a high prevalence of HIV infection, miltefosine (100 mg daily for 28 days) was safer but less effective than treatment with parenteral sodium stibogluconate (SSG; 20 mg $Sb^V$/kg daily for 30 days) [319]. Preliminary, uncontrolled data from Ethiopia suggest that the combination of L-AmB and miltefosine might be more effective than monotherapy for VL in HIV-infected persons in that setting [381]; retrospective data regarding L-AmB–miltefosine combination therapy for VL in coinfected persons in India also have been published [382].

In southern Europe, the incidence of VL (i.e., of symptomatic visceral infection) markedly decreased in the late 1990s, after the introduction and widespread use of ART [11, 41, 42, 383–388]. In addition, ART has been associated with improved survival among coinfected persons in southern Europe, as well as with longer intervals between induction therapy and relapse of VL [38, 42, 388]. However, benefits regarding treatment outcomes per se have not been clearly shown [34]. Standard regimens of ART should be used, pending identification or development of regimens optimized for persons with leishmaniasis. Although *in vitro* data suggest that certain HIV-1 protease inhibitors—particularly, if present at high concentrations—might have direct inhibitory effects against *Leishmania*, the potential clinical relevance of such data is not yet known [389].

Although the benefits of ART generally outweigh the risks, treated persons should be monitored for adverse reactions. Increased toxicity may be noted if particular antiretroviral agents are used in conjunction with $Sb^V$ or miltefosine therapy [390]; for example, coadministration of SSG and zidovudine could potentiate bone-marrow toxicity, and ritonavir-containing protease inhibitors could potentiate gastrointestinal symptoms in patients treated with miltefosine. Leishmaniasis-associated IRIS appears to be relatively uncommon [391, 392]. In a case series in southern Europe, none of 11 HIV-coinfected persons with documented subclinical visceral infection developed active VL after starting ART [386]. Case reports have described development of symptomatic VL in association with ART [393–395]. Other case reports have described predominantly dermatologic (or mucosal/uveal) manifestations, often in a diffuse pattern, resembling PKDL or disseminated/diffuse CL [348, 392, 396–398].

In a systematic review, the identified risk factors for relapse of VL in coinfected persons included a CD4 cell count <100 cells/mm$^3$ when VL initially was diagnosed, a poor incremental increase in the CD4 cell count in response to ART, lack of secondary prophylaxis, and history of a previous relapse [87]. Relapse within 1 year posttreatment is the norm, even among persons who were treated with L-AmB and who receive ART and secondary prophylaxis, which provide only partial protection against relapses [39, 87]. Patients should be monitored indefinitely (until immune reconstitution) for clinical evidence of relapse. If possible, the diagnosis should be parasitologically confirmed. A positive PCR result with a nonquantitative method would not confirm that the person is experiencing a relapse, and a negative result would not exclude relapse. Although testing serial blood specimens with a quantitative *Leishmania* PCR (qPCR) assay may help identify persons at risk for clinical relapse (i.e., persons with increasing parasitemia levels), such assays are not generally available for clinical use in North America.

Antileishmanial treatment is indicated for persons who have a documented recurrence of VL. At least for amphotericin formulations, relapses typically represent immunologic rather than drug failure (drug resistance) [363]. Therefore, reinduction therapy with L-AmB typically is justified. Alternatives to consider for some persons/settings include monotherapy with a different medication or, potentially, combination therapy (e.g., L-AmB plus miltefosine). Even if a clinical response is noted, coinfected persons may have serial relapses at progressively shorter intervals and may develop chronic refractory VL [399].

Secondary prophylaxis with an effective antileishmanial drug should be initiated after the end of the initial treatment course (i.e., to persons who respond to the induction therapy) and administered at periodic intervals thereafter. The optimal agent and regimen (e.g., dose and dosing interval) have not been defined, and comparative data regarding different regimens are not available [87]. The results of one small, randomized clinical trial of secondary prophylaxis have been published. Among the eight persons who received amphotericin B lipid complex (3 mg/kg IV) every 3 weeks, the 1-year relapse rate was 50%, compared with 78% among the nine persons who did not receive secondary prophylaxis [400]. In nonrandomized studies and case series, periodic doses of parenteral L-AmB (e.g., 3–5 mg/kg every 3–4 weeks) [38, 401], pentamidine isethionate (e.g., 4 mg salt/kg [up to 300 mg per dose] every 2–4 weeks) [402–404], and SSG (e.g., 20 mg $Sb^V$/kg every 3–4 weeks) [38, 405] have been associated with decreased relapse rates. Experience using miltefosine for secondary prophylaxis is limited [379, 380, 406].

Discontinuation of secondary prophylaxis can be considered in persons without evidence of active *Leishmania* infection whose CD4 cell counts on ART have been >200–350 cells/mm$^3$ for ≥6 months [11, 407]. However, relapses of VL have been reported in coinfected persons with CD4 cell counts >200 cells/mm$^3$ or undetectable HIV viral loads [42, 87, 372, 408, 409].

**XXIV. How should HIV/AIDS-associated CL or ML be treated in persons in North America who do not have evidence of VL, and what other management issues should be considered?**

*Recommendations*

67. In HIV/AIDS-associated CL/ML, systemic antileishmanial therapy is recommended, particularly in persons

who are moderately to severely immunosuppressed (e.g., have CD4+ T-lymphocyte cell counts <200–350 cells/mm$^3$), who may be at increased risk for suboptimal therapeutic responses, for posttreatment relapses, and for cutaneous, mucosal, or visceral dissemination [Strong, very low].

68. The systemic regimens used for CL/ML in otherwise comparable immunocompetent persons typically are recommended for the initial treatment of coinfected persons, taking into account the potentials for drug interactions and toxicity (Tables 3 and 4) [Strong, very low]. *Remark: Whether coinfected persons who experience multiple posttreatment relapses of CL/ML would benefit from secondary prophylaxis (chronic maintenance therapy) has not yet been established.*
69. Antiretroviral therapy (ART) should be initiated or optimized in accordance with standard practice for HIV/AIDS; no evidence-based, CL/ML-specific recommendations regarding ART have been established [Strong, low].

*Evidence Summary*

In HIV-coinfected persons, cutaneous, mucosal, and visceral dissemination can be caused by essentially any of the *Leishmania* species that infect humans [8, 11, 13], not just by species stereotypically associated with diffuse CL, disseminated CL, American ML, or VL, although the risks, pathophysiology, and clinical manifestations may differ (e.g., from those for classic New World ML [410–414]). Even in some coinfected persons in the Americas, clinically manifest ML has been the first identified leishmanial form, which sometimes has been followed by the development of cutaneous lesions [11, 12, 415].

Not surprisingly, the clinical management issues are more complex for persons with HIV/AIDS-associated CL/ML than they are for CL/ML in general. In coinfected persons, the spectrum of clinical manifestations is even broader and the evidence base is even weaker—constrained by the absence of data from randomized, controlled treatment trials and by the heterogeneity of the limited anecdotal data regarding the use of various antileishmanial agents in persons with HIV/AIDS-associated CL/ML [9–12, 16, 214, 413, 416–420].

In general, CL/ML-coinfected (vs HIV-uninfected) persons, particularly if severely immunosuppressed (CD4 cell count <200 cells/mm$^3$), may be less apt to respond to the initial treatment course or to have a durable, relapse-free response [11] and may be more likely to have drug-associated toxicity. In addition, in part because of the nonquantified risk for dissemination in such persons, use of systemic therapy generally is considered prudent, even for treatment of seemingly focal CL. Whether the systemic regimens used for the initial treatment of CL/ML in HIV-uninfected persons should be modified for coinfected persons is not yet clear [8, 11, 362].

On principle, ART should be initiated or optimized in accordance with standard practice for HIV/AIDS; relatively limited data regarding use of ART in persons who have CL/ML without VL have been published. Clinicians should be aware of the potential for increased toxicity if particular antiretroviral agents are used in conjunction with Sb$^V$ or miltefosine therapy [390], as well as the potential for interactions between antiretroviral agents and azole/triazole drugs (including ketoconazole, itraconazole, and fluconazole) [390]. Occasional cases of presentation or worsening of CL or mucosal/uveal involvement that might have represented IRIS reactions have been reported in the New and Old Worlds in persons without current or past evidence of VL [392, 421–427]. *Leishmania* infection that becomes manifest or worsens after initiation of ART should be treated with antileishmanial (and, if indicated, corticosteroid) therapy.

**XXV. What is the preferred treatment of visceral/cutaneous leishmaniasis in immunocompromised hosts with solid organ transplant, persons with lymphatic-hematologic malignancies, or persons receiving immunosuppressive therapy for other reasons?**

*Recommendations*

70. Liposomal amphotericin B (L-AmB) is recommended as the drug of choice for immunosuppressed persons with VL (Table 3) [Strong, low]. *Remarks: The FDA-approved regimen is 4 mg/kg/day IV on days 1–5, 10, 17, 24, 31, and 38 (total dose of 40 mg/kg). Higher doses and longer durations of therapy may be needed depending in part on the level of immunosuppression.*
71. Doses of immunosuppressive drugs should be decreased in persons with VL during antileishmanial therapy whenever possible [Strong, very low].
72. Secondary prophylaxis is not recommended for initial management in persons with VL who have not manifested a relapse [Weak, low]. *Remark*: *Immunosuppressed persons with VL who are not coinfected with HIV typically have higher response rates to initial treatment and lower recurrence rates than HIV-coinfected persons.*
73. Routine serologic screening of organ donors from leishmaniasis-endemic areas is not recommended. If an available donor is known to be seropositive, it is advisable to perform clinical and laboratory monitoring of the recipient in the post-transplant period rather than to reject the organ for transplant [Strong, low].
74. We suggest that clinicians not routinely perform diagnostic testing to assess persons for evidence of asymptomatic visceral infection, including persons who have lived or traveled in leishmaniasis-endemic regions (Figure 3) and are considering organ transplantation or initiation of therapy with biologic immunomodulating agents. Immunosuppressed persons known or found to be asymptomatically infected and those with a history of VL warrant close monitoring. Neither preemptive treatment nor primary prophylaxis for VL in asymptomatically infected persons is suggested [Weak, very low].
75. Immunosuppressed persons with VL who are not coinfected with HIV should be monitored for a minimum of 1 year (ideally lifelong or until effective immune reconstitution) to assess for posttreatment relapse. During clinical follow-up, assess for symptoms and, if present, pursue parasitologic confirmation of relapse [Strong, very low].
76. Confirmed VL therapeutic failure typically can be managed by retreatment using L-AmB at the same or

a higher total dose [Strong, very low]. *Remark: Pentavalent antimonials could be used in some persons with VL under close follow-up.*

77. We suggest that CL/ML associated with the use of tumor necrosis factor (TNF)-alpha antagonist therapy be managed with systemic therapy and standard drug regimens for the pertinent setting/species (e.g., geographic area where the infection was acquired) [Weak, very low]. *Remark: Withdrawal of TNF-α antagonists during antileishmanial therapy may be appropriate: the risks, benefits, and feasibility of this action should be assessed on a case-by-case basis.*

*Evidence Summary*

With the number of immunosuppressed persons increasing globally and travel to and from leishmaniasis-endemic regions becoming more common, imported cases in countries where leishmaniasis is not endemic may increase [128, 428, 429, 430, 431, 432]. In the context of immunosuppression, VL can result from reactivation of dormant infection or from *de novo* infection if the person lives or travels in a VL-endemic area. Therefore, protective measures to prevent exposure to sand fly bites are recommended, particularly for immunocompromised travelers going to leishmaniasis-endemic regions [433, 434].

During a 2009–2012 *L. infantum* outbreak in Madrid, Spain, many of the detected VL cases were in patients who were immunocompromised for reasons other than HIV infection [435]. Most of the cases in this setting are VL, but CL and ML have also been observed [213]. Among transplant recipients, VL has been most commonly reported in recipients of renal transplants (the most common type of solid organ transplant) but also has been reported in recipients of other types of solid organs, including heart, liver, and lung [86, 213, 436–439], as well as in hematopoietic stem cell/bone marrow recipients [213]. In VL-organ transplant cases, the median onset time was in the first year post transplant, and high-dose corticosteroid use during the previous 6-month period was a risk factor for development of VL [440]. Cases of VL and CL have been associated with the use of various immunosuppressive drugs, such as azathioprine, methotrexate, corticosteroids, cyclosporine, and cyclophosphamide [213, 441–443]. Reports of leishmaniasis manifesting after several months of treatment with a TNF-α antagonist suggest a higher risk in patients receiving infliximab or adalimumab than etanercept [444]. Several cancer-associated (mainly hematologic malignancies) cases of VL have been reported, in the context of the use of various chemotherapeutic agents or monoclonal antibodies [213]. Leishmaniasis rarely has been described in patients with primary immunodeficiency conditions [213].

During the 2009–2012 outbreak of leishmaniasis in a region of Madrid, Spain, in which 446 cases of leishmaniasis (286 CL and 160 VL) were identified (421 [94%] of which were defined as confirmed), 68 cases (18 CL and 50 VL) were in persons with at least one identified "intrinsic risk factor" [435]; only 18 (26%) of these 68 case-patients were infected with HIV, including 2 (11%) of the 18 who had CL and 16 (32%) of the 50 with VL. In hospitalized persons in Madrid with confirmed VL, 1% had associated solid tumors and 2.5% had hematopoietic malignancies [445]. Among 54 persons with CL in a retrospective study for the period 1992–2012 at a tertiary care hospital in Barcelona, Spain, 16 persons were classified as immunocompromised: 7 were infected with HIV, 4 had an autoimmune disease, 3 had a lymphoproliferative disorder, 9 received immunosuppressive therapy, and 2 received biologic immunomodulating therapy [446]. In an evidence base mainly comprised of case reports, the most common leishmaniasis-malignancy association has been between VL and acute lymphocytic leukemia (ALL); other malignancies reportedly associated with VL include acute myelogenous leukemia (AML), chronic lymphocytic leukemia, primary cutaneous T-cell lymphoma (Sezary syndrome), Hodgkins and Non-Hodgkins lymphoma, and multiple myeloma [213, 442, 447, 448].

VL is usually diagnosed months to years after initiation of chemotherapy, often with a prolonged regimen of multiple courses/complicated clinical course implying longer periods of immunosuppression. The agents associated with VL include corticosteroids, rituximab, and fludarabine; bortezomib has been mentioned in several case reports and bevacizumab in one report. In patients with leukemia, potential confounding factors include increased ascertainment of VL because of the increased frequency of obtaining bone marrow biopsy specimens (in the context of fever and cytopenias), frequent blood transfusions (which, in leishmaniasis-endemic regions, could be an additional risk factor for *Leishmania* infection), and common early use of amphotericin B for empiric therapy of fever in neutropenic patients (which could partially treat VL, depending on the dosage regimen). In general, chemotherapy was held or interrupted briefly during the course of antileishmanial therapy (usually with L-AmB) but then resumed because of the life-threatening malignancy; in some cases, this was associated with therapeutic failure. Decisions about the use of subsequent secondary prophylaxis for leishmaniasis should be individualized, depending in part on the level and the anticipated duration of the underlying immunosuppression.

The clinical presentation of VL in immunosuppressed patients who are not infected with HIV often resembles the syndrome described in immunocompetent patients (see Background information about leishmaniasis) [86,441]. However, as with HIV/AIDS-associated VL (see XXIII), atypical parasite dissemination (see below) and severe clinical forms have been reported in persons with advanced immunosuppression. Severely immunosuppressed patients with VL also may have or develop dermatologic manifestations suggestive of diffuse/disseminated CL or mucosal involvement, which can be caused *Leishmania* species not typically associated with dermatologic or mucosal dissemination [410]. VL can mimic systemic lupus erythematosus, rheumatoid arthritis, or hematologic malignancy, which can lead to diagnostic errors.

The evidence base regarding treatment of VL in patients who are immunocompromised for reasons other than HIV/AIDS consists of case reports and small case series. Response rates to initial treatment usually are higher and recurrence rates lower than in HIV-infected patients, although response rates typically are lower than those in immunocompetent patients. We suggest that VL treatment be similar to what is recommended for induction therapy (ie, L-AmB) for persons with HIV/AIDS-associated VL (see XXIII)

and that decisions about the patient's immunosuppressive therapy (if applicable) be individualized, taking into account the level and anticipated duration of immunosuppression. Despite a lack of systematic comparisons, L-AmB is recommended because of its safety profile and generally good efficacy [42, 362, 449, 450]. If an immunocompromised person with *L. infantum-chagasi* infection does not have therapeutic success with L-AmB, the administration of pentavalent antimonials can be effective [451].

As with HIV/AIDS-associated CL, in persons in whom cell-mediated immune responses are seriously compromised for other reasons, the response to treatment of CL can be suboptimal; we recommend systemic instead of local therapy for CL [362]. Species identification is important when treating CL in immunocompromised persons because the treatment recommendations or prognosis may vary (see VI) [452]. If the immunosuppression is drug induced (e.g., associated with treatment with corticosteroids, TNF-α antagonists, or methotrexate), we recommend decreasing the dose of the drug, if feasible, or, if pertinent, withdrawing TNF-α antagonists [452]. The evidence base for treatment of ML in immunocompromised persons is limited [411]. In general, we recommend that CL and ML be treated as per the recommendations for HIV-coinfected patients.

Screening transplant donors for evidence of *Leishmania* infection typically is not recommended. However, serologic screening of transplant recipients who have a history of potential exposure to *Leishmania* may be considered before transplantation. Iatrogenic transmission of VL via organ transplantation may occur [86, 453]. Immunosuppressed persons known to have asymptomatic infection or to have a history of VL warrant close clinical monitoring [433]. Primary prophylaxis is not recommended at present. When blood quantitative real-time PCR (qPCR) testing is available in North America, it may be helpful to detect early reactivation (see VII) [454]. In a Brazilian study, none of the liver recipients who had positive *Leishmania* PCR results at the time of transplantation and none of the recipients of a PCR-positive organ developed VL during a median follow-up period of 2 years; no prophylaxis was given [453, 455].

Secondary prophylaxis has been administered to patients with non-HIV-related immunosuppression only in exceptional cases, because many patients have therapeutic success without maintenance therapy regardless of the continued use of immunosuppressive medication. However, in a recent case-control study of solid organ transplant recipients (n = 36 cases) with VL, 26% had therapeutic failure, often in the first year post transplantation, raising the issue of whether secondary prophylaxis should be considered [440].

## SPECIAL POPULATIONS AND LEISHMANIASIS

### XXVI. How should the treatment of leishmaniasis be modified in persons who are pregnant or lactating; are young children or older adults; or have comorbidities such as renal, hepatic, or cardiac dysfunction?

*Recommendations*

78. In general, clinically manifest cases of VL and ML should be treated even in these special populations of persons because the benefits of treatment typically outweigh the risks. However, patient-specific factors, including the presence of comorbid conditions, should be considered in the selection of the antileishmanial therapy, dosage regimen, and monitoring approach (Table 4) [Strong, low].
79. Decisions regarding whether and how to treat cases of CL in persons with special characteristics or comorbid conditions should take into account the potential risks and benefits of various approaches, such as initially observing without antileishmanial therapy, deferring treatment (e.g., until after pregnancy/delivery), and using local (vs systemic) therapy as the sole approach or as a temporizing measure, if otherwise appropriate and feasible [Strong, very low].

*Evidence Summary*

Basic principles applicable to all persons with leishmaniasis —including the needs to ensure that the diagnosis is correct and thereafter to individualize all treatment decisions (e.g., whether, when, and how to treat)—are particularly important for persons who have special characteristics or comorbid conditions [42, 456]. However, the published information regarding special patient populations is even more limited than it is for leishmaniasis in general, which compounds the challenges inherent to individualizing patient care, such as assessing the probability and magnitude of the potential risks and benefits of various treatment and monitoring approaches. The heterogeneity encompassed not only by leishmaniasis but also by each of the special populations (e.g., children, older adults, persons with comorbidities) compounds the complexities; expert consultation and clinical judgment typically are needed. The discussion below focuses on selected topics, principles, and cautionary notes. In Table 4, additional pertinent details and perspective about antileishmanial medications are provided. Considerations applicable to HIV-coinfected persons and to persons who are immunocompromised for other reasons are addressed in clinical questions XXIII–XXV.

*Pregnant women*

*VL during pregnancy*: Among the special populations discussed in this section, the stakes are highest for persons who have clinically manifest VL during pregnancy—which, in case reports/series, has been associated with maternal deaths, abortions/miscarriages, preterm deliveries, small-for-gestational-age infants, and congenital transmission [42, 457–460]. In this context, the benefits of antileishmanial therapy during pregnancy typically outweigh the potential risks to the fetus—particularly if persons are treated with liposomal amphotericin B (L-AmB), which is classified in pregnancy category B. Although relatively limited data have been published regarding L-AmB therapy for VL in pregnant women [291, 458, 459, 461–465], good maternal/fetal outcomes typically have been reported; amphotericin B compounds also have been safely used for treatment of systemic fungal infections in pregnant women. However, as always, treated persons should be closely monitored for adverse events (Table 4).

Case reports and retrospective analyses of uncontrolled data suggest that $Sb^V$ therapy, especially during the first

(or second) trimester, may increase the risks for abortions/miscarriages and preterm deliveries [42, 459, 460]. Additional cautionary notes include the observations of embryotoxicity in laboratory animals [457, 466] and of genotoxicity in a murine model although not *in vitro* [467].

Miltefosine is contraindicated during pregnancy. Although human risk data are lacking, embryo-fetal toxicity (in rats and rabbits) and teratogenicity (in rats) have been observed in animals exposed to doses lower than those recommended for human persons [468, 469]. According to the FDA-approved product label [469], women with reproductive potential should have a negative pregnancy test before starting therapy and should use effective contraception both during the treatment course and for 5 months thereafter. Although the optimal duration of posttreatment contraception is not yet known [158, 470], miltefosine has a long terminal-elimination half-life and still can be detected, at low levels of uncertain relevance, in human plasma specimens collected 5–6 months after a 28-day treatment course [158]. Persons of both sexes also should be informed that the potential for adverse effects on human fertility has not been adequately evaluated but that reproductive effects have been noted in animal studies (impaired fertility in rats of both sexes and testicular atrophy in male rats) [469, 471, 472].

*CL during pregnancy*: General principles regarding whether and how to treat CL are applicable in the context of pregnancy. Not all persons who have CL (vs VL) need to be treated or need to receive (or to start with) drug therapy (e.g., a combination of wound care plus a physical modality, such as heat or cryotherapy [191, 473], might suffice or constitute a temporizing measure in some persons). Not all persons who may benefit from drug treatment need to receive systemic therapy or need to receive it promptly (e.g., in some persons, the therapy may be postponed until later in pregnancy or after delivery). Although pregnancy has been associated with development of atypically large or exuberant CL lesions in some persons [474–476], even those with exophytic lesions do not necessarily need to be treated during pregnancy [474].

Various cautionary notes also apply. The effects, if any, of immunologic, hormonal, or other pregnancy-associated changes on the risks for mucosal dissemination of *Viannia* parasites or on the risks for development or progression of ML are not known; the possibility of an association between untreated CL and adverse fetal outcomes [474] has not been excluded; the potential for maternal local drug therapy (e.g., with intralesional $Sb^V$ or topical paromomycin) to cause fetal toxicity has not been evaluated; miltefosine is contraindicated during pregnancy (as discussed above); and oral ”azole” compounds should not be viewed as safe alternatives for pregnant women.

### *Lactating women*

In general, the potential for drug-associated risks to breastfeeding infants cannot be excluded (Table 4). Amphotericin B probably is compatible with breastfeeding [477]: the concentration of drug (if present at all) in breast milk likely would be low; and its oral bioavailability is known to be low. $Sb^V$ therapy may be compatible with breastfeeding: on the basis of data in a case report, breastfeeding infants are unlikely to be exposed to detectable or toxic antimony levels [478]. Lactating women should be advised not to breastfeed during treatment with miltefosine or for 5 months thereafter [469]: miltefosine is presumed to be transferred into breast milk [468, 471]; and the possibility that a breastfeeding infant might experience toxic effects cannot be excluded on the basis of available data.

### *Children*

The morbidity that can be associated with CL, coupled with the lack of optimal treatment modalities for cases of CL that warrant therapy, can be especially problematic for children. Facial lesions/scars, in particular, can have long-lasting social and psychological consequences; however, not all persons with CL who would or could benefit from treatment are candidates for local therapy (see XIV); and even local (not just parenteral) therapies may be challenging to administer [192, 479, 480]. For example, some children may need to be sedated before intralesional injections of $Sb^V$ [479]; the need to retreat on multiple days compounds such issues [481]. As a broad generalization, otherwise healthy young children typically tolerate systemic antileishmanial therapies as well as, if not better than, adults—which, as discussed below regarding $Sb^V$ and miltefosine, might in part reflect lower drug exposures in some children. Pediatric cases of VL (and systemic fungal infections) have been treated with amphotericin B compounds, including L-AmB, without reports of unusual side effects [291, 482, 483] or of the need for pediatric-specific dosage regimens.

### *Dosing issues for systemic pentavalent antimonial ($Sb^V$) and miltefosine therapy*

Perspective is provided below regarding particular dosing issues of clinical relevance—e.g., some children may have suboptimal or subtherapeutic drug exposures when treated with parenteral $Sb^V$ or oral miltefosine according to conventional weight-based regimens.

*$Sb^V$*: In some studies/settings, young children (variably defined) have had lower initial response rates or less durable responses to parenteral $Sb^V$ therapy compared with older children or adults treated with the same weight-adjusted dosage regimen [484–487]. In a pharmacokinetic (PK) study in Colombians with CL treated with meglumine antimoniate (20 mg $Sb^V$/kg IM daily), children aged 3–6 years had a statistically significant (42%) lower plasma exposure to antimony (Sb) than adults aged 20–36 years, in the context of a significantly (75%) higher weight-adjusted renal clearance of Sb in young children [487]. To achieve Sb plasma exposures comparable to those in adults treated with a daily dose of 20 mg $Sb^V$ per kg of body weight, young children may need higher weight- (or otherwise-) adjusted doses. However, this PK study was not designed to assess exposure-response relationships or to assess the relationship between drug concentrations in plasma and at the intracellular target site; nor was it designed to evaluate the clinical need for (or the benefits/risks of) alternative dosing regimens/algorithms, either for this setting in Colombia or for pediatric leishmaniasis elsewhere [487]. As a broader point also applicable to adults, the optimal descriptor(s) of body size/physiologic function for $Sb^V$ dosing purposes has not been established. Adjustments in the $Sb^V$

dosage regimen (e.g., use of a lower daily dose in mg/kg) or selection of an alternative antileishmanial agent may be prudent for some persons who are elderly or obese or who have pertinent comorbid conditions (e.g., renal insufficiency); see below.

*Miltefosine*: As of 2014, the FDA-approved indications for miltefosine are limited to non-pregnant, non-breastfeeding persons infected with particular *Leishmania* species who are ≥12 years of age and weigh ≥30 kg [469]; and the FDA-approved capsule size is 50 mg, although 10-mg capsules also are manufactured. The conventional target dose is ~2.5 mg of miltefosine per kg of body weight per day, some data suggest that doses <2 mg/kg are associated with lower response rates [488], an upper limit of 150 mg per day was established in the past because of poor tolerability at higher doses, and divided dosing (in increments of 50 mg) is recommended to minimize gastrointestinal symptoms. In this context, the FDA-approved dosage regimen for persons who weigh 30 to 44 kg is one 50-mg capsule twice a day (total, 100 mg/day) for 28 consecutive days; and the maximum daily dose of 150 mg (in 3 divided doses) applies to all persons who weigh ≥45 kg (≥99 pounds) [469]. Using conventional weight-based dosing, persons who weigh >60 kg (>132 pounds)—as most North American adults do, in contrast to the majority of the persons in the clinical trials to date—receive <2.5 mg/kg per day (e.g., persons >75 kg receive <2 mg/kg per day).

In the United States, use of miltefosine in persons who are <12 years of age or who weigh <30 kg constitutes off-label use. However, younger children ≥2 years of age were included in some clinical trials of miltefosine for CL in South America [485, 489, 490] and for VL in South Asia [310, 312, 323, 471, 491–495]. Of particular note, in clinical trials of VL in South Asia and in associated PK analyses, young children (from 2 to 11 or 14 years of age), in comparison with adults, had lower cure rates (lower initial response rates or higher relapse rates) and lower plasma drug exposures; the maximum weight of the adults with VL (vs CL) whose data were included in the PK analyses was 58 kg [494]. To address the apparent difference in drug exposure between young children and adults, with the goal of improving response rates in children with VL, a dosing algorithm with nonlinear, allometric scaling based on fat-free mass has been proposed [494]; in the algorithm, the maximum daily dose remains unchanged at 150 mg. Clinical evaluations of the allometric regimen are pending [495], and the applicability of the proposed regimen to adults with comparatively high body weights remains to be established.

*Older adults and persons with pertinent comorbid conditions*

In general, as with other special populations, cases of VL should be treated even in older adults and persons with comorbid conditions, whereas not all cases of CL need to be treated or require systemic therapy (e.g., intralesional [vs parenteral] $Sb^V$ therapy may be an option for some persons [496]). Patient- and drug-specific characteristics (e.g., the likelihood and severity of various drug-associated toxicities; Table 4) should be considered when assessing the potential risks and benefits of various treatment and monitoring approaches. Some comorbid conditions (e.g., cardiac or renal dysfunction) or older age, as a proxy or surrogate marker, may increase the risk for certain drug-associated toxicities [42, 191, 456, 497–499]. Therefore, certain antileishmanial medications should not be used or should be used only with additional precautionary measures (e.g., expert consultation, dosage adjustments, and more frequent monitoring for toxicity) in persons with pertinent comorbid conditions or laboratory abnormalities.

Examples of issues and principles pertinent to $Sb^V$ therapy [497] are provided here for illustrative purposes. Baseline laboratory testing and periodic monitoring (at least weekly) of the electrocardiogram (ECG), serum chemistry values, and complete blood count are recommended (Table 4). Because laboratory manifestations of toxicity typically develop gradually, such testing/monitoring should help minimize the risk for life-threatening or irreversible adverse events [497, 500–502]. Because antimony is renally excreted, baseline renal insufficiency, depending in part on its severity and etiology, may warrant modifying the dosage regimen (decreasing the daily dose or increasing the dosing interval) or selecting an alternative therapy. Modifying the dosage regimen, monitoring more frequently (e.g., twice weekly or even more frequently), or selecting an alternative drug also may be prudent for some persons with baseline hepatic, pancreatic, or cardiac disease—e.g., for persons with arrhythmia-associated conditions, including persons who have baseline prolongation of the corrected QT interval (QTc) or who are receiving medications (besides $Sb^V$) that may prolong the QTc. $Sb^V$ therapy should be interrupted if concave ST segments or QTc prolongation develop, especially if the absolute value of the QTc is >0.50 sec [497, 503] (Table 4).

**Acknowledgments**: The Panel expresses its gratitude for thoughtful reviews of an earlier version by Anthony Bryceson, Pierre Buffet, James Maguire, Henry Murray, Abhay Satoskar, as well as Edward Ryan (Chair) and members of the ASTMH Standards and Treatment Guidelines Committee. The panel also thanks Dean Winslow, the liaison of the IDSA Standards and Practice Guidelines Committee, and Genet Demisashi for their efforts guiding us through the process.

***Financial Support.*** Support for these guidelines was provided by the Infectious Diseases Society of America and the American Society of Tropical Medicine and Hygiene.

**Potential Conflict of Interest:**

The following list is a reflection of what has been reported to IDSA. To provide thorough transparency, IDSA requires full disclosure of all relationships, regardless of relevancy to the guideline topic. Evaluation of such relationships as potential conflicts of interest (COI) is determined by a review process that includes assessment by the SPGC Chair, the SPGC liaison to the development Panel, the Board of Directors liaison to the SPGC, and, if necessary, the Conflict of Interest (COI) Task Force of the Board. This assessment of disclosed relationships for possible COI will be based on the relative weight of the financial relationship (i.e., monetary amount) and the relevance of the relationship (i.e., the degree to which an association might reasonably be interpreted by an independent observer as related to the topic or recommendation of consideration). The reader of these guidelines should be mindful of this when the list of disclosures is reviewed. For activities outside of the submitted work, N.A. has received payment from Up to Date.

For activities outside of the submitted work M.E. has received research grants from the Israel National Institute for Health Policy Research (Rotavirus). For activities outside of the submitted work, R.P. has received personal fees from Merck & Co, Inc. and has a patent Protozoacidal Activity of the Phenothiazines (U.S. patent number 4,407,800), 1983.issued. No conflicts: E.C., M.L., R.L., B.H., P.W., S.J., and A.M.

This is an open-access article distributed under the terms of the Creative Commons Attribution License, which permits unrestricted use, distribution, and reproduction in any medium, provided the original author and source are credited.

## References

1. Guyatt GH, Oxman AD, Vist GE, et al. GRADE: an emerging consensus on rating quality of evidence and strength of recommendations. BMJ (Clinical research ed) 2008; 336(7650): 924-6.
2. Gonzalez U. Cochrane reviews on neglected diseases: the case of cutaneous leishmaniasis. The Cochrane database of systematic reviews 2013; 3: Ed000055.
3. Gonzalez U, Pinart M, Reveiz L, Alvar J. Interventions for Old World cutaneous leishmaniasis. The Cochrane database of systematic reviews 2008; (4): Cd005067.
4. Guyatt GH, Oxman AD, Schunemann HJ, Tugwell P, Knottnerus A. GRADE guidelines: a new series of articles in the Journal of Clinical Epidemiology. J Clin Epidemiol 2011; 64(4): 380-2.
5. Wortmann GW, Aronson NE, Miller RS, Blazes D, Oster CN. Cutaneous leishmaniasis following local trauma: a clinical pearl. Clinical infectious diseases : an official publication of the Infectious Diseases Society of America 2000; 31(1): 199-201.
6. Sanchez JL, Diniega BM, Small JW, et al. Epidemiologic investigation of an outbreak of cutaneous leishmaniasis in a defined geographic focus of transmission. The American journal of tropical medicine and hygiene 1992; 47(1): 47-54.
7. Puig L, Pradinaud R. Leishmania and HIV co-infection: dermatological manifestations. Ann Trop Med Parasitol 2003; 97 Suppl 1: 107-14.
8. Foulet F, Cosnes A, Dellion S, et al. Leishmania major cutaneous leishmaniasis in HIV-positive patients does not spread to extralesional sites. Arch Dermatol 2006; 142(10): 1368-9.
9. Couppie P, Clyti E, Sobesky M, et al. Comparative study of cutaneous leishmaniasis in human immunodeficiency virus (HIV)-infected patients and non-HIV-infected patients in French Guiana. The British journal of dermatology 2004; 151(6): 1165-71.
10. Guerra JA, Coelho LI, Pereira FR, et al. American tegumentary leishmaniasis and HIV-AIDS association in a tertiary care center in the Brazilian Amazon. The American journal of tropical medicine and hygiene 2011; 85(3): 524-7.
11. Alvar J, Aparicio P, Aseffa A, et al. The relationship between leishmaniasis and AIDS: the second 10 years. Clin Microbiol Rev 2008; 21(2): 334-59, table of contents.
12. Lindoso JA, Barbosa RN, Posada-Vergara MP, et al. Unusual manifestations of tegumentary leishmaniasis in AIDS patients from the New World. The British journal of dermatology 2009; 160(2): 311-8.
13. Niamba P, Goumbri-Lompo O, Traore A, Barro-Traore F, Soudre RT. Diffuse cutaneous leishmaniasis in an HIV-positive patient in western Africa. Australas J Dermatol 2007; 48(1): 32-4.
14. Niamba P, Traore A, Goumbri-Lompo O, et al. [Cutaneous leishmania in HIV patient in Ouagadougou: clinical and therapeutic aspects]. Ann Dermatol Venereol 2006; 133(6-7): 537-42.
15. Ndiaye PB, Develoux M, Dieng MT, Huerre M. [Diffuse cutaneous leishmaniasis and acquired immunodeficiency syndrome in a Senegalese patient]. Bulletin de la Societe de pathologie exotique 1996; 89(4): 282-6.
16. Torrico F, Parrado R, Castro R, et al. Co-Infection of Leishmania (Viannia) braziliensis and HIV: report of a case of mucosal leishmaniasis in Cochabamba, Bolivia. The American journal of tropical medicine and hygiene 2009; 81(4): 555-8.
17. Mattos M, Caiza A, Fernandes O, et al. American cutaneous leishmaniasis associated with HIV infection: report of four cases. Journal of the European Academy of Dermatology and Venereology : JEADV 1998; 10(3): 218-25.
18. Mota Sasaki M, Matsumo Carvalho M, Schmitz Ferreira ML, Machado MP. Cutaneous Leishmaniasis Coinfection in AIDS Patients: Case Report and Literature Review. The Brazilian journal of infectious diseases : an official publication of the Brazilian Society of Infectious Diseases 1997; 1(3): 142-4.
19. Aoun J, Habib R, Charaffeddine K, Taraif S, Loya A, Khalifeh I. Caseating granulomas in cutaneous leishmaniasis. PLoS Neglected Tropical Diseases 2014; 8(10): e3255.
20. Ahluwalia S, Lawn SD, Kanagalingam J, Grant H, Lockwood DN. Mucocutaneous leishmaniasis: an imported infection among travellers to central and South America. BMJ (Clinical research ed) 2004; 329(7470): 842-4.
21. Camargo RA, Tuon FF, Sumi DV, et al. Mucosal leishmaniasis and abnormalities on computed tomographic scans of paranasal sinuses. The American journal of tropical medicine and hygiene 2010; 83(3): 515-8.
22. Marsden PD. Mucosal leishmaniasis ("espundia" Escomel, 1911). Transactions of the Royal Society of Tropical Medicine and Hygiene 1986; 80(6): 859-76.
23. Marsden PD. Mucocutaneous leishmaniasis. BMJ (Clinical research ed) 1990; 301(6753): 656-7.
24. Figueroa RA, Lozano LE, Romero IC, et al. Detection of Leishmania in unaffected mucosal tissues of patients with cutaneous leishmaniasis caused by Leishmania (Viannia) species. The Journal of infectious diseases 2009; 200(4): 638-46.
25. Lessa MM, Lessa HA, Castro TW, et al. Mucosal leishmaniasis: epidemiological and clinical aspects. Braz J Otorhinolaryngol 2007; 73(6): 843-7.
26. Jones TC, Johnson WD Jr., Barretto AC, et al. Epidemiology of American cutaneous leishmaniasis due to Leishmania braziliensis braziliensis. The Journal of infectious diseases 1987; 156(1): 73-83.
27. de Oliveira-Neto MP, Mattos MS, Perez MA, et al. American tegumentary leishmaniasis (ATL) in Rio de Janeiro State, Brazil: main clinical and epidemiologic characteristics. International Journal of Dermatology 2000; 39(7): 506-14.
28. Lessa HA, Carvalho EM, Marsden PD. Eustachian tube blockage with consequent middle ear infection in mucosal leishmaniasis. Revista da Sociedade Brasileira de Medicina Tropical 1994; 27(2): 103.
29. Boaventura VS, de Oliveira JG, Costa JM, et al. The value of the otorhinolaryngologic exam in correct mucocutaneous leishmaniasis diagnosis. The American journal of tropical medicine and hygiene 2009; 81(3): 384-6.
30. Boggild AK, Valencia BM, Veland N, et al. Non-invasive cytology brush PCR diagnostic testing in mucosal leishmaniasis: superior performance to conventional biopsy with histopathology. PLoS ONE 2011; 16(10): e26395.
31. Magill A. Leishmania species. Chapter 277. In: Principles and Practice of Infectious Diseases. 8th ed. Philadelphia, PA: Elsevier 2015: 3091–107.
32. Magill A. Leishmaniasischapter 99. In Hunters Tropical Medicine and Emerging Infectious Diseases 2013; 9th edition: 739-60.
33. Jeronimo S, de Queiroz Sousa A, Pearson R. Leishmaniasis. Principles, pathogen, and practice. In Tropical Infectious diseeases 2006; (2nd edition): 1095-113.
34. Jarvis JN, Lockwood DN. Clinical aspects of visceral leishmaniasis in HIV infection. Current Opinion in Infectious Diseases 2013; 26(1): 1-9.
35. Albuquerque LC, Mendonca IR, Cardoso PN, et al. HIV/AIDS-related visceral leishmaniasis: a clinical and epidemiological description of visceral leishmaniasis in northern Brazil. Revista da Sociedade Brasileira de Medicina Tropical 2014; 47(1): 38-46.
36. Hurissa Z, Gebre-Silassie S, Hailu W, et al. Clinical characteristics and treatment outcome of patients with visceral leishmaniasis and HIV co-infection in northwest Ethiopia. Tropical medicine & international health : TM & IH 2010; 15(7): 848-55.

37. Ritmeijer K, Veeken H, Melaku Y, et al. Ethiopian visceral leishmaniasis: generic and proprietary sodium stibogluconate are equivalent; HIV co-infected patients have a poor outcome. Transactions of the Royal Society of Tropical Medicine and Hygiene 2001; 95(6): 668-72.
38. Pintado V, Martin-Rabadan P, Rivera ML, Moreno S, Bouza E. Visceral leishmaniasis in human immunodeficiency virus (HIV)-infected and non-HIV-infected patients. A comparative study. Medicine (Baltimore) 2001; 80(1): 54-73.
39. Pasquau F, Ena J, Sanchez R, et al. Leishmaniasis as an opportunistic infection in HIV-infected patients: determinants of relapse and mortality in a collaborative study of 228 episodes in a Mediterreanean region. European journal of clinical microbiology & infectious diseases : official publication of the European Society of Clinical Microbiology 2005; 24(6): 411-8.
40. Lopez-Velez R, Perez-Molina JA, Guerrero A, et al. Clinicoepidemiologic characteristics, prognostic factors, and survival analysis of patients coinfected with human immunodeficiency virus and Leishmania in an area of Madrid, Spain. The American journal of tropical medicine and hygiene 1998; 58(4): 436-43.
41. Rosenthal E, Marty P, del Giudice P, et al. HIV and Leishmania coinfection: a review of 91 cases with focus on atypical locations of Leishmania. Clinical infectious diseases : an official publication of the Infectious Diseases Society of America 2000; 31(4): 1093-5.
42. World Health Organization. Control of the leishmaniases. World Health Organi-zation technical report series Geneva, Switzerland: WHO, 2010; 949:xii–xiii, 1–186, back cover.
43. Bosch RJ, Rodrigo AB, Sanchez P, de Galvez MV, Herrera E. Presence of Leishmania organisms in specific and non-specific skin lesions in HIV-infected individuals with visceral leishmaniasis. International Journal of Dermatology 2002; 41(10): 670-5.
44. Colebunders R, Depraetere K, Verstraeten T, et al. Unusual cutaneous lesions in two patients with visceral leishmaniasis and HIV infection. Journal of the American Academy of Dermatology 1999; 41(5 Pt 2): 847-50.
45. Alsina-Gibert M, Lopez-Lerma I, Martinez-Chamorro E, Herrero-Mateu C. Cutaneous manifestations of visceral leishmaniasis resistant to liposomal amphotericin B in an HIV-positive patient. Arch Dermatol 2006; 142(6): 787-9.
46. Ara M, Maillo C, Peon G, et al. Visceral leishmaniasis with cutaneous lesions in a patient infected with human immunodeficiency virus. The British journal of dermatology 1998; 139 (1): 114-7.
47. Perrin C, Taillan B, Hofman P, Mondain V, Lefichoux Y, Michiels JF. Atypical cutaneous histological features of visceral leishmaniasis in acquired immunodeficiency syndrome. Am J Dermatopathol 1995; 17(2): 145-50.
48. Gonzalez-Beato MJ, Moyano B, Sanchez C, et al. Kaposi's sarcoma-like lesions and other nodules as cutaneous involvement in AIDS-related visceral leishmaniasis. The British journal of dermatology 2000; 143(6): 1316-8.
49. Farooq U, Choudhary S, Chacon AH, et al. Post-kala-azar dermal leishmaniasis in HIV-infected patients with AIDS: A report of two cases diagnosed in the USA. International Journal of Dermatology 2013; 52(9): 1098-104.
50. Stark D, Pett S, Marriott D, Harkness J. Post-kala-azar dermal leishmaniasis due to Leishmania infantum in a human immunodeficiency virus type 1-infected patient. Journal of clinical microbiology 2006; 44(3): 1178-80.
51. Bittencourt A, Silva N, Straatmann A, Nunes VL, Follador I, Badaro R. Post-kala-azar dermal leishmaniasis associated with AIDS. The Brazilian journal of infectious diseases : an official publication of the Brazilian Society of Infectious Diseases 2002; 6(6): 313-6.
52. Rihl M, Stoll M, Ulbricht K, Bange FC, Schmidt RE. Successful treatment of post-kala-azar dermal leishmaniasis (PKDL) in a HIV infected patient with multiple relapsing leishmaniasis from Western Europe. J Infect 2006; 53(1): e25-7.
53. Catorze G, Alberto J, Afonso A, Vieira R, Cortes S, Campino L. [Leishmania infantum/HIV co-infection: cutaneous lesions following treatment of visceral leishmaniasis]. Ann Dermatol Venereol 2006; 133(1): 39-42.
54. Carnauba D Jr., Konishi CT, Petri V, Martinez IC, Shimizu L, Pereira-Chioccola VL. Atypical disseminated leishmaniasis similar to post-kala-azar dermal leishmaniasis in a Brazilian AIDS patient infected with Leishmania (Leishmania) infantum chagasi: a case report. Int J Infect Dis 2009; 13(6): e504-7.
55. Herwaldt BL. Leishmaniasis. Lancet 1999; 354(9185): 1191-9.
56. Boggild AK, Ramos AP, Valencia BM, et al. Diagnostic performance of filter paper lesion impression PCR for secondarily infected ulcers and nonulcerative lesions caused by cutaneous leishmaniasis. Journal of clinical microbiology 2011; 49 (3): 1097-100.
57. Boggild AK, Ramos AP, Espinosa D, et al. Clinical and demographic stratification of test performance: a pooled analysis of five laboratory diagnostic methods for American cutaneous leishmaniasis. The American journal of tropical medicine and hygiene 2010; 83(2): 345-50.
58. Robinson RJ, Agudelo S, Muskus C, et al. The method used to sample ulcers influences the diagnosis of cutaneous leishmaniasis. Transactions of the Royal Society of Tropical Medicine and Hygiene 2002; 96 Suppl 1: S169-71.
59. Ramirez JR, Agudelo S, Muskus C, et al. Diagnosis of cutaneous leishmaniasis in Colombia: the sampling site within lesions influences the sensitivity of parasitologic diagnosis. Journal of clinical microbiology 2000; 38(10): 3768-73.
60. Navin TR, Arana FE, de Merida AM, Arana BA, Castillo AL, Silvers DN. Cutaneous leishmaniasis in Guatemala: comparison of diagnostic methods. The American journal of tropical medicine and hygiene 1990; 42(1): 36-42.
61. Weigle KA, de Davalos M, Heredia P, Molineros R, Saravia NG, D'Alessandro A. Diagnosis of cutaneous and mucocutaneous leishmaniasis in Colombia: a comparison of seven methods. The American journal of tropical medicine and hygiene 1987; 36(3): 489-96.
62. Reithinger R, Dujardin JC. Molecular diagnosis of leishmaniasis: current status and future applications. Journal of clinical microbiology 2007; 45(1): 21-5.
63. Klotz O, Lindenberg H. The Pathology of Leishmaniosis of the Nose. Am J Trop Med Hyg 1923; 3: 117-41.
64. Jara M, Adaui V, Valencia BM, et al. Real-time PCR assay for detection and quantification of Leishmania (Viannia) organisms in skin and mucosal lesions: exploratory study of parasite load and clinical parameters. Journal of clinical microbiology 2013; 51(6): 1826-33.
65. Gomes CM, de Paula NA, Cesetti MV, Roselino AM, Sampaio RN. Mucocutaneous leishmaniasis: accuracy and molecular validation of noninvasive procedures in a L. (V.) braziliensis-endemic area. Diagnostic Microbiology and Infectious Disease 2014; 79(4): 413-8.
66. Hartley MA, Drexler S, Ronet C, Beverley SM, Fasel N. The immunological, environmental, and phylogenetic perpetrators of metastatic leishmaniasis. Trends in Parasitology 2014; 30(8): 412-22.
67. Osorio LE, Castillo CM, Ochoa MT. Mucosal leishmaniasis due to Leishmania (Viannia) panamensis in Colombia: clinical characteristics. The American journal of tropical medicine and hygiene 1998; 59(1): 49-52.
68. Boaventura VS, Cafe V, Costa J, et al. Concomitant early mucosal and cutaneous leishmaniasis in Brazil. The American journal of tropical medicine and hygiene 2006; 75(2): 267-9.
69. Turetz ML, Machado PR, Ko AI, et al. Disseminated leishmaniasis: a new and emerging form of leishmaniasis observed in northeastern Brazil. The Journal of infectious diseases 2002; 186(12): 1829-34.
70. Lessa HA, Lessa MM, Guimaraes LH, et al. A proposed new clinical staging system for patients with mucosal leishmaniasis. Transactions of the Royal Society of Tropical Medicine and Hygiene 2012; 106(6): 376-81.
71. Mary C, Faraut F, Drogoul MP, et al. Reference values for Leishmania infantum parasitemia in different clinical presentations: quantitative polymerase chain reaction for therapeutic monitoring and patient follow-up. The American journal of tropical medicine and hygiene 2006; 75(5): 858-63.
72. Sundar S, Rai M. Laboratory diagnosis of visceral leishmaniasis. Clin Diagn Lab Immunol 2002; 9(5): 951-8.

73. da Silva MR, Stewart JM, Costa CH. Sensitivity of bone marrow aspirates in the diagnosis of visceral leishmaniasis. Am J Trop Med Hyg 2005; 72(6): 811-4.
74. Thakur CP. A comparison of intercostal and abdominal routes of splenic aspiration and bone marrow aspiration in the diagnosis of visceral leishmaniasis. Transactions of the Royal Society of Tropical Medicine and Hygiene 1997; 91(6): 668-70.
75. Salam MA, Khan MG, Bhaskar KR, Afrad MH, Huda MM, Mondal D. Peripheral blood buffy coat smear: a promising tool for diagnosis of visceral leishmaniasis. Journal of clinical microbiology 2012; 50(3): 837-40.
76. WHO WHO. Visceral leishmaniasis rapid diagnostic test performance. Diagnostic Evaluation Series No. 4. Geneva: World Health Organization. Available: http://www.who.int/tdr/publications/documents/vl-rdt-evaluation.pdf. Accessed 11Jan2016. 2011.
77. Boelaert M, Verdonck K, Menten J, et al. Rapid tests for the diagnosis of visceral leishmaniasis in patients with suspected disease. The Cochrane database of systematic reviews 2014; 6: Cd009135.
78. van Griensven J, Diro E. Visceral leishmaniasis. Infectious disease clinics of North America 2012; 26(2): 309-22.
79. Vaish M, Mehrotra S, Chakravarty J, Sundar S. Noninvasive molecular diagnosis of human visceral leishmaniasis. Journal of clinical microbiology 2011; 49(5): 2003-5.
80. Weina PJ, Neafie RC, Wortmann G, Polhemus M, Aronson NE. Old world leishmaniasis: an emerging infection among deployed US military and civilian workers. Clinical infectious diseases : an official publication of the Infectious Diseases Society of America 2004; 39(11): 1674-80.
81. Goto H, Lindoso JA. Current diagnosis and treatment of cutaneous and mucocutaneous leishmaniasis. Expert Review of Anti-Infective Therapy 2010; 8(4): 419-33.
82. Cassagne C, Pratlong F, Jeddi F, et al. Identification of Leishmania at the species level with matrix-assisted laser desorption ionization time-of-flight mass spectrometry. Clinical microbiology and infection : the official publication of the European Society of Clinical Microbiology and Infectious Diseases 2014; 20(6): 551-7.
83. Mouri O, Morizot G, Van der Auwera G, et al. Easy identification of leishmania species by mass spectrometry. PLoS Neglected Tropical Diseases 2014; 8(6): e2841.
84. Van der Auwera G, Dujardin JC. Species typing in dermal leishmaniasis. Clin Microbiol Rev 2015; 28(2): 265-94.
85. Hodiamont CJ, Kager PA, Bart A, et al. Species-directed therapy for leishmaniasis in returning travellers: a comprehensive guide. PLoS Neglected Tropical Diseases 2014; 8(5): e2832.
86. Antinori S, Cascio A, Parravicini C, Bianchi R, Corbellino M. Leishmaniasis among organ transplant recipients. The Lancet Infectious diseases 2008; 8(3): 191-9.
87. Cota GF, de Sousa MR, Rabello A. Predictors of visceral leishmaniasis relapse in HIV-infected patients: a systematic review. PLoS Neglected Tropical Diseases 2011; 5(6): e1153.
88. Tavares CA, Fernandes AP, Melo MN. Molecular diagnosis of leishmaniasis. Expert Rev Mol Diagn 2003; 3(5): 657-67.
89. de Ruiter CM, van der Veer C, Leeflang MM, Deborggraeve S, Lucas C, Adams ER. Molecular tools for diagnosis of visceral leishmaniasis: systematic review and meta-analysis of diagnostic test accuracy. Journal of clinical microbiology 2014; 52(9): 3147-55.
90. Mary C, Faraut F, Lascombe L, Dumon H. Quantification of Leishmania infantum DNA by a real-time PCR assay with high sensitivity. Journal of clinical microbiology 2004; 42(11): 5249-55.
91. Wortmann G, Sweeney C, Houng HS, et al. Rapid diagnosis of leishmaniasis by fluorogenic polymerase chain reaction. The American journal of tropical medicine and hygiene 2001; 65(5): 583-7.
92. Odiwuor SO, Saad AA, De Doncker S, et al. Universal PCR assays for the differential detection of all Old World Leishmania species. European journal of clinical microbiology & infectious diseases : official publication of the European Society of Clinical Microbiology 2011; 30(2): 209-18.
93. Wortmann G, Hochberg LP, Arana BA, Rizzo NR, Arana F, Ryan JR. Diagnosis of cutaneous leishmaniasis in Guatemala using a real-time polymerase chain reaction assay and the Smartcycler. The American journal of tropical medicine and hygiene 2007; 76(5): 906-8.
94. Schonian G, Mauricio I, Cupolillo E. Is it time to revise the nomenclature of Leishmania? Trends in Parasitology 2010; 26(10): 466-9.
95. Welch RJ, Anderson BL, Litwin CM. Rapid immunochromatographic strip test for detection of anti-K39 immunoglobulin G antibodies for diagnosis of visceral leishmaniasis. Clinical and vaccine immunology : CVI 2008; 15(9): 1483-4.
96. Burns JM Jr., Shreffler WG, Benson DR, Ghalib HW, Badaro R, Reed SG. Molecular characterization of a kinesin-related antigen of Leishmania chagasi that detects specific antibody in African and American visceral leishmaniasis. Proceedings of the National Academy of Sciences of the United States of America 1993; 90(2): 775-9.
97. Chappuis F, Rijal S, Soto A, Menten J, Boelaert M. A meta-analysis of the diagnostic performance of the direct agglutination test and rK39 dipstick for visceral leishmaniasis. BMJ (Clinical research ed) 2006; 333(7571): 723.
98. Sundar S, Sahu M, Mehta H, et al. Noninvasive management of Indian visceral leishmaniasis: clinical application of diagnosis by K39 antigen strip testing at a kala-azar referral unit. Clinical infectious diseases : an official publication of the Infectious Diseases Society of America 2002; 35(5): 581-6.
99. Braz RF, Nascimento ET, Martins DR, et al. The sensitivity and specificity of Leishmania chagasi recombinant K39 antigen in the diagnosis of American visceral leishmaniasis and in differentiating active from subclinical infection. The American journal of tropical medicine and hygiene 2002; 67(4): 344-8.
100. Vallur AC, Reinhart C, Mohamath R, et al. Accurate Serodetection of Asymptomatic Leishmania donovani Infection by Use of Defined Antigens. Journal of clinical microbiology 2016; 54(4): 1025-30.
101. Bezuneh A, Mukhtar M, Abdoun A, et al. Comparison of point-of-care tests for the rapid diagnosis of visceral leishmaniasis in East African patients. Am J Trop Med Hyg 2014; 91(6): 1109-15.
102. Pattabhi S, Whittle J, Mohamath R, et al. Design, development and evaluation of rK28-based point-of-care tests for improving rapid diagnosis of visceral leishmaniasis. PLoS Neglected Tropical Diseases 2010; 4(9).
103. Ritmeijer K, Melaku Y, Mueller M, Kipngetich S, O'Keeffe C, Davidson RN. Evaluation of a new recombinant K39 rapid diagnostic test for Sudanese visceral leishmaniasis. The American journal of tropical medicine and hygiene 2006; 74(1): 76-80.
104. Gidwani K, Picado A, Ostyn B, et al. Persistence of Leishmania donovani antibodies in past visceral leishmaniasis cases in India. Clinical and vaccine immunology : CVI 2011; 18(2): 346-8.
105. Mandal J, Khurana S, Dubey ML, Bhatia P, Varma N, Malla N. Evaluation of direct agglutination test, rk39 Test, and ELISA for the diagnosis of visceral leishmaniasis. The American journal of tropical medicine and hygiene 2008; 79(1): 76-8.
106. Kar K. Serodiagnosis of leishmaniasis. Crit Rev Microbiol 1995; 21(2): 123-52.
107. Romero HD, Silva Lde A, Silva-Vergara ML, et al. Comparative study of serologic tests for the diagnosis of asymptomatic visceral leishmaniasis in an endemic area. The American journal of tropical medicine and hygiene 2009; 81(1): 27-33.
108. Cota GF, de Sousa MR, Demarqui FN, Rabello A. The diagnostic accuracy of serologic and molecular methods for detecting visceral leishmaniasis in HIV infected patients: meta-analysis. PLoS Neglected Tropical Diseases 2012; 6(5): e1665.
109. ter Horst R, Tefera T, Assefa G, Ebrahim AZ, Davidson RN, Ritmeijer K. Field evaluation of rK39 test and direct agglutination test for diagnosis of visceral leishmaniasis in a population with high prevalence of human immunodeficiency virus in Ethiopia. The American journal of tropical medicine and hygiene 2009; 80(6): 929-34.
110. Nascimento ET, Moura ML, Queiroz JW, et al. The emergence of concurrent HIV-1/AIDS and visceral leishmaniasis in Northeast Brazil. Transactions of the Royal Society of Tropical Medicine and Hygiene 2011; 105(5): 298-300.
111. Hartzell JD, Aronson NE, Weina PJ, Howard RS, Yadava A, Wortmann GW. Positive rK39 serologic assay results in US

servicemen with cutaneous leishmaniasis. The American journal of tropical medicine and hygiene 2008; 79(6): 843-6.
112. Molinet FJ, Ampuero JS, Costa RD, Noronha EF, Romero GA. Specificity of the rapid rK39 antigen-based immunochromatographic test Kalazar Detect(r) in patients with cutaneous leishmaniasis in Brazil. Memorias do Instituto Oswaldo Cruz 2013; 108(3).
113. Andrade-Narvaez FJ, Medina-Peralta S, Vargas-Gonzalez A, Canto-Lara SB, Estrada-Parra S. The histopathology of cutaneous leishmaniasis due to Leishmania (Leishmania) mexicana in the Yucatan peninsula, Mexico. Revista do Instituto de Medicina Tropical de Sao Paulo 2005; 47(4): 191-4.
114. Gutierrez Y, Salinas GH, Palma G, Valderrama LB, Santrich CV, Saravia NG. Correlation between histopathology, immune response, clinical presentation, and evolution in Leishmania braziliensis infection. Am J Trop Med Hyg 1991; 45(3): 281-9.
115. Craig C, Faust E. Clinical Parasitology. Lea and Febiger, Philadelphia PA 1943; 3rd edition.
116. Reithinger R, Dujardin JC, Louzir H, Pirmez C, Alexander B, Brooker S. Cutaneous leishmaniasis. The Lancet Infectious diseases 2007; 7(9): 581-96.
117. Weigle K, Saravia NG. Natural history, clinical evolution, and the host-parasite interaction in New World cutaneous Leishmaniasis. Clinics in Dermatology 1996; 14(5): 433-50.
118. Murray HW, Berman JD, Davies CR, Saravia NG. Advances in leishmaniasis. Lancet 2005; 366(9496): 1561-77.
119. Blum J, Lockwood DNJ, Visser L, et al. Local or systemic treatment for New World cutaneous leishmaniasis? Re-evaluating the evidence for the risk of mucosal leishmaniasis. International Health 2012; 4(3): 153-63.
120. Singer C, Armstrong D, Jones TC, Spiro RH. Imported mucocutaneous leishmaniasis in New York City. Report of a patient treated with amphotericin B. The American journal of medicine 1975; 59(3): 444-7.
121. Maguire GP, Bastian I, Arianayagam S, Bryceson A, Currie BJ. New World cutaneous leishmaniasis imported into Australia. Pathology 1998; 30(1): 73-6.
122. Lohuis PJ, Lipovsky MM, Hoepelman AI, Hordijk GJ, Huizing EH. Leishmania braziliensis presenting as a granulomatous lesion of the nasal septum mucosa. J Laryngol Otol 1997; 111(10): 973-5.
123. Rosbotham JL, Corbett EL, Grant HR, Hay RJ, Bryceson AD. Imported mucocutaneous leishmaniasis. Clinical and experimental dermatology 1996; 21(4): 288-90.
124. Blum J, Junghanss T, Hatz C. [Erroneous tracks in the diagnosis of cutaneous and mucocutaneous leishmaniasis]. Schweiz Rundsch Med Prax 1994; 83(37): 1025-9.
125. Scope A, Trau H, Bakon M, Yarom N, Nasereddin A, Schwartz E. Imported mucosal leishmaniasis in a traveler. Clinical infectious diseases : an official publication of the Infectious Diseases Society of America 2003; 37(6): e83-7.
126. Lawn SD, Whetham J, Chiodini PL, et al. New world mucosal and cutaneous leishmaniasis: an emerging health problem among British travellers. Qjm 2004; 97(12): 781-8.
127. Eichner S, Thoma-Uszynski S, Herrgott I, et al. Clinical complexity of Leishmania (Viannia) braziliensis infections amongst travelers. European journal of dermatology : EJD 2013; 23(2): 218-23.
128. Bart A, van Thiel PP, de Vries HJ, Hodiamont CJ, Van Gool T. Imported leishmaniasis in the Netherlands from 2005 to 2012: epidemiology, diagnostic techniques and sequence-based species typing from 195 patients. Euro surveillance : bulletin Europeen sur les maladies transmissibles = European communicable disease bulletin 2013; 18(30): 20544.
129. Lachaud L, Dedet JP, Marty P, et al. Surveillance of leishmaniases in France, 1999 to 2012. Euro surveillance : bulletin Europeen sur les maladies transmissibles = European communicable disease bulletin 2013; 18(29): 20534.
130. Neumayr ALC, Walter C, Stoeckle M, Braendle N, Glatz K, Blum JA. Successful treatment of imported mucosal leishmania infantum leishmaniasis with miltefosine after severe hypokalemia under meglumine antimoniate treatment. Journal of Travel Medicine 2012; 19(2): 124-6.
131. Lawn SD, Yardley V, Vega-Lopez F, Watson J, Lockwood DN. New World cutaneous leishmaniasis in returned travellers: treatment failures using intravenous sodium stibogluconate. Transactions of the Royal Society of Tropical Medicine and Hygiene 2003; 97(4): 443-5.
132. Schwartz E, Hatz C, Blum J. New world cutaneous leishmaniasis in travellers. The Lancet Infectious diseases 2006; 6(6): 342-9.
133. Andrade-Narvaez FJ, Vargas-Gonzalez A, Canto-Lara SB, Damian-Centeno AG. Clinical picture of cutaneous leishmaniases due to Leishmania (Leishmania) mexicana in the Yucatan peninsula, Mexico. Memorias do Instituto Oswaldo Cruz 2001; 96(2): 163-7.
134. Velasco O, Savarino SJ, Walton BC, Gam AA, Neva FA. Diffuse cutaneous leishmaniasis in Mexico. The American journal of tropical medicine and hygiene 1989; 41(3): 280-8.
135. Richter J, Hanus I, Haussinger D, Loscher T, Harms G. Mucosal Leishmania infantum infection. Parasitology Research 2011; 109(3): 959-62.
136. Velez I, Agudelo S, Robledo S, et al. Diffuse cutaneous leishmaniasis with mucosal involvement in Colombia, caused by an enzymatic variant of Leishmania panamensis. Transactions of the Royal Society of Tropical Medicine and Hygiene 1994; 88(2): 199.
137. Bonfante-Garrido R, Barroeta S, de Alejos MA, et al. Disseminated American cutaneous leishmaniasis. International Journal of Dermatology 1996; 35(8): 561-5.
138. Silveira FT, Lainson R, Corbett CE. Clinical and immunopathological spectrum of American cutaneous leishmaniasis with special reference to the disease in Amazonian Brazil: a review. Memorias do Instituto Oswaldo Cruz 2004; 99(3): 239-51.
139. Zerpa O, Ulrich M, Blanco B, et al. Diffuse cutaneous leishmaniasis responds to miltefosine but then relapses. The British journal of dermatology 2007; 156(6): 1328-35.
140. Khamesipour A. Therapeutic vaccines for leishmaniasis. Expert Opin Biol Ther 2014; 14(11): 1641-9.
141. Saravia NG, Weigle K, Segura I, et al. Recurrent lesions in human Leishmania braziliensis infection–reactivation or reinfection? Lancet 1990; 336(8712): 398-402.
142. Netto EM, Marsden PD, Llanos-Cuentas EA, et al. Long-term follow-up of patients with Leishmania (Viannia) braziliensis infection and treated with Glucantime. Transactions of the Royal Society of Tropical Medicine and Hygiene 1990; 84(3): 367-70.
143. Mendonca MG, de Brito ME, Rodrigues EH, Bandeira V, Jardim ML, Abath FG. Persistence of leishmania parasites in scars after clinical cure of American cutaneous leishmaniasis: is there a sterile cure? The Journal of infectious diseases 2004; 189(6): 1018-23.
144. Paul BC, Rafii B, Achlatis S, Amin MR, Branski RC. Morbidity and patient perception of flexible laryngoscopy. Ann Otol Rhinol Laryngol 2012; 121(11): 708-13.
145. Dimier-David L, David C, Munoz M, et al. [Epidemiological, clinical and biological features of mucocutaneous leishmaniasis in Bolivia after a 221 patient sample]. Bulletin de la Societe de pathologie exotique 1993; 86(2): 106-11.
146. Davies CR, Reithinger R, Campbell-Lendrum D, Feliciangeli D, Borges R, Rodriguez N. The epidemiology and control of leishmaniasis in Andean countries. Cad Saude Publica 2000; 16(4): 925-50.
147. Oliveira-Neto MP, Schubach A, Mattos M, da Costa SC, Pirmez C. Intralesional therapy of American cutaneous leishmaniasis with pentavalent antimony in Rio de Janeiro, Brazil–an area of Leishmania (V.) braziliensis transmission. International journal of dermatology 1997; 36(6): 463-8.
148. Oliveira-Neto MP, Schubach A, Mattos M, Goncalves-Costa SC, Pirmez C. A low-dose antimony treatment in 159 patients with American cutaneous leishmaniasis: extensive follow-up studies (up to 10 years). Am J Trop Med Hyg 1997; 57(6): 651-5.
149. Camuset G, Remy V, Hansmann Y, Christmann D, Gomes de Albuquerque C, Sena Casseb GA. [Mucocutaneous leishmaniasis in Brazilian Amazonia]. Med Mal Infect 2007; 37(6): 343-6.
150. Velez I, Agudelo S, Hendrickx E, et al. Inefficacy of allopurinol as monotherapy for Colombian cutaneous leishmaniasis. A randomized, controlled trial. Ann Intern Med 1997; 126(3): 232-6.

151. D'Oliveira Junior A, Machado PR, Carvalho EM. Evaluating the efficacy of allopurinol for the treatment of cutaneous leishmaniasis. International journal of dermatology 1997; 36(12): 938-40.
152. Bourreau E, Ginouves M, Prevot G, et al. Presence of Leishmania RNA Virus 1 in Leishmania guyanensis Increases the Risk of First-Line Treatment Failure and Symptomatic Relapse. The Journal of infectious diseases 2016; 213(1): 105-11.
153. Adaui V, Lye LF, Akopyants NS, et al. Association of the Endobiont Double-Stranded RNA Virus LRV1 With Treatment Failure for Human Leishmaniasis Caused by Leishmania braziliensis in Peru and Bolivia. The Journal of infectious diseases 2016; 213(1): 112-21.
154. Machado P, Araujo C, Da Silva AT, et al. Failure of early treatment of cutaneous leishmaniasis in preventing the development of an ulcer. Clinical infectious diseases : an official publication of the Infectious Diseases Society of America 2002; 234(12): E69-73.
155. Llanos-Cuentas A, Tulliano G, Araujo-Castillo R, et al. Clinical and parasite species risk factors for pentavalent antimonial treatment failure in cutaneous leishmaniasis in Peru. Clinical infectious diseases : an official publication of the Infectious Diseases Society of America 2008; 46(2): 223-31.
156. Solomon M, Pavlotzky F, Barzilai A, Schwartz E. Liposomal amphotericin B in comparison to sodium stibogluconate for Leishmania braziliensis cutaneous leishmaniasis in travelers. Journal of the American Academy of Dermatology 2013; 68(2): 284-9.
157. Soto J, Arana BA, Toledo J, et al. Miltefosine for new world cutaneous leishmaniasis. Clinical infectious diseases : an official publication of the Infectious Diseases Society of America 2004; 38(9): 1266-72.
158. Dorlo TP, van Thiel PP, Huitema AD, et al. Pharmacokinetics of miltefosine in Old World cutaneous leishmaniasis patients. Antimicrobial Agents and Chemotherapy 2008; 52(8): 2855-60.
159. Solomon M, Pavlotsky F, Leshem E, Ephros M, Trau H, Schwartz E. Liposomal amphotericin B treatment of cutaneous leishmaniasis due to Leishmania tropica. Journal of the European Academy of Dermatology and Venereology : JEADV 2011; 25(8): 973-7.
160. Wortmann G, Zapor M, Ressner R, et al. Lipsosomal amphotericin B for treatment of cutaneous leishmaniasis. Am J Trop Med Hyg 2010; 83(5): 1028-33.
161. Neves LO, Talhari AC, Gadelha EP, et al. A randomized clinical trial comparing meglumine antimoniate, pentamidine and amphotericin B for the treatment of cutaneous leishmaniasis by Leishmania guyanensis. Anais brasileiros de dermatologia 2011; 86(6): 1092-101.
162. Correia D, Macedo VO, Carvalho EM, et al. [Comparative study of meglumine antimoniate, pentamidine isethionate and aminosidine sulfate in the treatment of primary skin lesions caused by Leishmania (Viannia) braziliensis]. Revista da Sociedade Brasileira de Medicina Tropical 1996; 29(5): 447-53.
163. Andersen EM, Cruz-Saldarriaga M, Llanos-Cuentas A, et al. Comparison of meglumine antimoniate and pentamidine for peruvian cutaneous leishmaniasis. Am J Trop Med Hyg 2005; 72(2): 133-7.
164. van der Meide WF, Sabajo LO, Jensema AJ, et al. Evaluation of treatment with pentamidine for cutaneous leishmaniasis in Suriname. International Journal of Dermatology 2009; 48(1): 52-8.
165. Soto J, Buffet P, Grogl M, Berman J. Successful treatment of Colombian cutaneous leishmaniasis with four injections of pentamidine. The American journal of tropical medicine and hygiene 1994; 50(1): 107-11.
166. Soto-Mancipe J, Grogl M, Berman JD. Evaluation of pentamidine for the treatment of cutaneous leishmaniasis in Colombia. Clinical infectious diseases : an official publication of the Infectious Diseases Society of America 1993; 16(3): 417-25.
167. Navin TR, Arana BA, Arana FE, Berman JD, Chajon JF. Placebo-controlled clinical trial of sodium stibogluconate (Pentostam) versus ketoconazole for treating cutaneous leishmaniasis in Guatemala. The Journal of infectious diseases 1992; 165(3): 528-34.
168. Saenz RE, Paz H, Berman JD. Efficacy of ketoconazole against Leishmania braziliensis panamensis cutaneous leishmaniasis. The American journal of medicine 1990; 89(2): 147-55.
169. Alrajhi AA, Ibrahim EA, De Vol EB, Khairat M, Faris RM, Maguire JH. Fluconazole for the treatment of cutaneous leishmaniasis caused by Leishmania major. N Engl J Med 2002; 346(12): 891-5.
170. Morizot G, Delgiudice P, Caumes E, et al. Healing of Old World cutaneous leishmaniasis in travelers treated with fluconazole: drug effect or spontaneous evolution? The American journal of tropical medicine and hygiene 2007; 76(1): 48-52.
171. Emad M, Hayati F, Fallahzadeh MK, Namazi MR. Superior efficacy of oral fluconazole 400 mg daily versus oral fluconazole 200 mg daily in the treatment of cutaneous leishmania major infection: a randomized clinical trial. Journal of the American Academy of Dermatology 2011; 64(3): 606-8.
172. Sousa AQ, Frutuoso MS, Moraes EA, Pearson RD, Pompeu MM. High-dose oral fluconazole therapy effective for cutaneous leishmaniasis due to Leishmania (Vianna) braziliensis. Clinical infectious diseases : an official publication of the Infectious Diseases Society of America 2011; 53(7): 693-5.
173. Sacks DL, Barral A, Neva FA. Thermosensitivity patterns of Old vs. New World cutaneous strains of Leishmania growing within mouse peritoneal macrophages in vitro. Am J Trop Med Hyg 1983; 32(2): 300-4.
174. Reithinger R, Mohsen M, Wahid M, et al. Efficacy of thermotherapy to treat cutaneous leishmaniasis caused by Leishmania tropica in Kabul, Afghanistan: a randomized, controlled trial. Clinical infectious diseases : an official publication of the Infectious Diseases Society of America 2005; 40(8): 1148-55.
175. Safi N, Davis GD, Nadir M, Hamid H, Robert LL Jr., Case AJ. Evaluation of thermotherapy for the treatment of cutaneous leishmaniasis in Kabul, Afghanistan: a randomized controlled trial. Military medicine 2012; 177(3): 345-51.
176. Aronson NE, Wortmann GW, Byrne WR, et al. A randomized controlled trial of local heat therapy versus intravenous sodium stibogluconate for the treatment of cutaneous Leishmania major infection. PLoS neglected tropical diseases 2010; 4 (3): e628.
177. Sadeghian G, Nilfroushzadeh MA, Iraji F. Efficacy of local heat therapy by radiofrequency in the treatment of cutaneous leishmaniasis, compared with intralesional injection of meglumine antimoniate. Clinical and experimental dermatology 2007; 32 (4): 371-4.
178. Navin TR, Arana BA, Arana FE, de Merida AM, Castillo AL, Pozuelos JL. Placebo-controlled clinical trial of meglumine antimonate (glucantime) vs. localized controlled heat in the treatment of cutaneous leishmaniasis in Guatemala. Am J Trop Med Hyg 1990; 42(1): 43-50.
179. Lopez L, Robayo M, Vargas M, Velez ID. Thermotherapy. An alternative for the treatment of American cutaneous leishmaniasis. Trials 2012; 13: 58.
180. al-Majali O, Routh HB, Abuloham O, Bhowmik KR, Muhsen M, Hebeheba H. A 2-year study of liquid nitrogen therapy in cutaneous leishmaniasis. International journal of dermatology 1997; 36(6): 460-2.
181. Salmanpour R, Razmavar MR, Abtahi N. Comparison of intralesional meglumine antimoniate, cryotherapy and their combination in the treatment of cutaneous leishmaniasis. International journal of dermatology 2006; 45(9): 1115-6.
182. Negera E, Gadisa E, Hussein J, et al. Treatment response of cutaneous leishmaniasis due to Leishmania aethiopica to cryotherapy and generic sodium stibogluconate from patients in Silti, Ethiopia. Transactions of the Royal Society of Tropical Medicine and Hygiene 2012; 106(8): 496-503.
183. Ranawaka RR, Weerakoon HS, Opathella N. Liquid nitrogen cryotherapy on Leishmania donovani cutaneous leishmaniasis. The Journal of dermatological treatment 2011; 22(4): 241-5.
184. Al-Gindan Y, Kubba R, Omer AH, el-Hassan AM. Cryosurgery in old world cutaneous leishmaniasis. The British journal of dermatology 1988; 118(6): 851-4.
185. Gurel MS, Ulukanligil M, Ozbilge H. Cutaneous leishmaniasis in Sanliurfa: epidemiologic and clinical features of the last four years (1997-2000). International journal of dermatology 2002; 41(1): 32-7.

186. Uzun S, Uslular C, Yucel A, Acar MA, Ozpoyraz M, Memisoglu HR. Cutaneous leishmaniasis: evaluation of 3,074 cases in the Cukurova region of Turkey. The British journal of dermatology 1999; 140(2): 347-50.
187. Thomaidou E, Horev L, Jotkowitz D, et al. Lymphatic Dissemination in Cutaneous Leishmaniasis Following Local Treatment. The American journal of tropical medicine and hygiene 2015.
188. Asilian A, Davami M. Comparison between the efficacy of photodynamic therapy and topical paromomycin in the treatment of Old World cutaneous leishmaniasis: a placebo-controlled, randomized clinical trial. Clinical and experimental dermatology 2006; 31(5): 634-7.
189. Enk CD, Nasereddin A, Alper R, Dan-Goor M, Jaffe CL, Wulf HC. Cutaneous leishmaniasis responds to daylight-activated photodynamic therapy: proof of concept for a novel self-administered therapeutic modality. The British journal of dermatology 2015; 172(5): 1364-70.
190. Meymandi S, Zandi S, Aghaie H, A. H. Efficacy of CO(2) laser for treatment of anthroponotic cutaneous leishmaniasis, compared with combination of cryotherapy and intralesional meglumine antimoniate. 2011.
191. Blum J, Buffet P, Visser L, et al. LeishMan recommendations for treatment of cutaneous and mucosal leishmaniasis in travelers, 2014. Journal of travel medicine 2014; 21(2): 116-29.
192. Reithinger R, Aadil K, Kolaczinski J, Mohsen M, Hami S. Social impact of leishmaniasis, Afghanistan. Emerging infectious diseases 2005; 11(4): 634-6.
193. Ranawaka RR, Weerakoon HS. Randomized, double-blind, comparative clinical trial on the efficacy and safety of intralesional sodium stibogluconate and intralesional 7% hypertonic sodium chloride against cutaneous leishmaniasis caused by L. donovani. The Journal of dermatological treatment 2010; 21(5): 286-93.
194. Alkhawajah AM, Larbi E, al-Gindan Y, Abahussein A, Jain S. Treatment of cutaneous leishmaniasis with antimony: intramuscular versus intralesional administration. Ann Trop Med Parasitol 1997; 91(8): 899-905.
195. Harms G, Chehade AK, Douba M, et al. A randomized trial comparing a pentavalent antimonial drug and recombinant interferon-gamma in the local treatment of cutaneous leishmaniasis. Transactions of the Royal Society of Tropical Medicine and Hygiene 1991; 85(2): 214-6.
196. Soto J, Rojas E, Guzman M, et al. Intralesional antimony for single lesions of bolivian cutaneous leishmaniasis. Clinical infectious diseases : an official publication of the Infectious Diseases Society of America 2013; 56(9): 1255-60.
197. Asilian A, Sadeghinia A, Faghihi G, Momeni A. Comparative study of the efficacy of combined cryotherapy and intralesional meglumine antimoniate (Glucantime) vs. cryotherapy and intralesional meglumine antimoniate (Glucantime) alone for the treatment of cutaneous leishmaniasis. International journal of dermatology 2004; 43(4): 281-3.
198. El-On J, Livshin R, Even-Paz Z, Hamburger D, Weinrauch L. Topical treatment of cutaneous leishmaniasis. The Journal of investigative dermatology 1986; 87(2): 284-8.
199. Soto JM, Toledo JT, Gutierrez P, et al. Treatment of cutaneous leishmaniasis with a topical antileishmanial drug (WR279396): phase 2 pilot study. Am J Trop Med Hyg 2002; 66(2): 147-51.
200. Sosa N, Capitan Z, Nieto J, et al. Randomized, double-blinded, phase 2 trial of WR 279,396 (paromomycin and gentamicin) for cutaneous leishmaniasis in Panama. Am J Trop Med Hyg 2013; 89(3): 557-63.
201. Kim DH, Chung HJ, Bleys J, Ghohestani RF. Is paromomycin an effective and safe treatment against cutaneous leishmaniasis? A meta-analysis of 14 randomized controlled trials. PLoS neglected tropical diseases 2009; 3(2): e381.
202. Shani-Adir A, Kamil S, Rozenman D, et al. Leishmania tropica in northern Israel: a clinical overview of an emerging focus. Journal of the American Academy of Dermatology 2005; 53(5): 810-5.
203. Ben Salah A, Buffet PA, Morizot G, et al. WR279,396, a third generation aminoglycoside ointment for the treatment of Leishmania major cutaneous leishmaniasis: a phase 2, randomized, double blind, placebo controlled study. PLoS neglected tropical diseases 2009; 3(5): e432.
204. Ben Salah A, Ben Messaoud N, Guedri E, et al. Topical paromomycin with or without gentamicin for cutaneous leishmaniasis. N Engl J Med 2013; 368(6): 524-32.
205. Schubach A, Marzochi MC, Cuzzi-Maya T, et al. Cutaneous scars in American tegumentary leishmaniasis patients: a site of Leishmania (Viannia) braziliensis persistence and viability eleven years after antimonial therapy and clinical cure. The American journal of tropical medicine and hygiene 1998; 58(6): 824-7.
206. Oster CN, Chulay JD, Hendricks LD, et al. American cutaneous leishmaniasis: a comparison of three sodium stibogluconate treatment schedules. Am J Trop Med Hyg 1985; 34(5): 856-60.
207. Herwaldt BL, Arana BA, Navin TR. The natural history of cutaneous leishmaniasis in Guatemala. The Journal of infectious diseases 1992; 165(3): 518-27.
208. Akilov OE, Khachemoune A, Hasan T. Clinical manifestations and classification of Old World cutaneous leishmaniasis. International Journal of Dermatology 2007; 46(2): 132-42.
209. Olliaro P, Vaillant M, Arana B, et al. Methodology of clinical trials aimed at assessing interventions for cutaneous leishmaniasis. PLoS neglected tropical diseases 2013; 7(3): e2130.
210. Bouros D, Zeros G, Panaretos C, Vassilatos C, Siafakas N. Palpation vs pen method for the measurement of skin tuberculin reaction (Mantoux test). Chest 1991; 99(2): 416-9.
211. Gonzalez U, Pinart M, Reveiz L, et al. Designing and reporting clinical trials on treatments for cutaneous leishmaniasis. Clinical infectious diseases : an official publication of the Infectious Diseases Society of America 2010; 51(4): 409-19.
212. Sadeghian G, Ziaei H, Bidabadi LS, Baghbaderani AZ. Decreased effect of glucantime in cutaneous leishmaniasis complicated with secondary bacterial infection. Indian Journal of Dermatology 2011; 56(1): 37-9.
213. Van Griensven J, Carrillo E, Lopez-Velez R, Lynen L, *Moreno J*. Leishmaniasis in immunosuppressed individuals. Clinical Microbiology and Infection 2014.
214. Amato VS, Tuon FF, Imamura R, Abegao de Camargo R, Duarte MI, Neto VA. Mucosal leishmaniasis: description of case management approaches and analysis of risk factors for treatment failure in a cohort of 140 patients in Brazil. Journal of the European Academy of Dermatology and Venereology : JEADV 2009; 23(9): 1026-34.
215. Casero R, Laconte L, Fraenza L, Iglesias N, Quinteros Greco C, Villablanca M. [Recidivant laryngeal leishmaniasis: an unusual case in an immunocompetent patient treated with corticosteroids]. Rev Argent Microbiol 2010; 42(2): 118-21.
216. Tuon FF, Sabbaga Amato V, Floeter-Winter LM, et al. Cutaneous leishmaniasis reactivation 2 years after treatment caused by systemic corticosteroids - first report. International Journal of Dermatology 2007; 46(6): 628-30.
217. Marovich MA, Lira R, Shepard M, et al. Leishmaniasis recidivans recurrence after 43 years: a clinical and immunologic report after successful treatment. Clinical infectious diseases : an official publication of the Infectious Diseases Society of America 2001; 33(7): 1076-9.
218. Kanj LF, Kibbi AG, Zaynoun S. Cutaneous leishmaniasis: an unusual case with atypical recurrence. Journal of the American Academy of Dermatology 1993; 28(3): 495-6.
219. Gangneux JP, Sauzet S, Donnard S, et al. Recurrent American cutaneous leishmaniasis. Emerging infectious diseases 2007; 13(9): 1436-8.
220. Mueller MC, Fleischmann E, Grunke M, Schewe S, Bogner JR, Loscher T. Relapsing cutaneous leishmaniasis in a patient with ankylosing spondylitis treated with infliximab. The American journal of tropical medicine and hygiene 2009; 81(1): 52-4.
221. Valencia C, Arevalo J, Dujardin JC, Llanos-Cuentas A, Chappuis F, Zimic M. Prediction score for antimony treatment failure in patients with ulcerative leishmaniasis lesions. PLoS neglected tropical diseases 2012; 6(6): e1656.
222. Unger A, O'Neal S, Machado PR, et al. Association of treatment of American cutaneous leishmaniasis prior to ulcer development with high rate of failure in northeastern Brazil. The

American journal of tropical medicine and hygiene 2009; 80(4): 574-9.
223. Rodrigues AM, Hueb M, Santos TA, Fontes CJ. [Factors associated with treatment failure of cutaneous leishmaniasis with meglumine antimoniate]. Revista da Sociedade Brasileira de Medicina Tropical 2006; 39(2): 139-45.
224. Mohammadzadeh M, Behnaz F, Golshan Z. Efficacy of glucantime for treatment of cutaneous leishmaniasis in Central Iran. Journal of infection and public health 2013; 6(2): 120-4.
225. Torres DC, Ribeiro-Alves M, Romero GA, Davila AM, Cupolillo E. Assessment of drug resistance related genes as candidate markers for treatment outcome prediction of cutaneous leishmaniasis in Brazil. Acta Tropica 2013; 126(2): 132-41.
226. Ait-Oudhia K, Gazanion E, Vergnes B, Oury B, Sereno D. Leishmania antimony resistance: what we know what we can learn from the field. Parasitology Research 2011; 109(5): 1225-32.
227. Rojas R, Valderrama L, Valderrama M, Varona MX, Ouellette M, Saravia NG. Resistance to antimony and treatment failure in human Leishmania (Viannia) infection. The Journal of infectious diseases 2006; 193(10): 1375-83.
228. Arevalo I, Tulliano G, Quispe A, et al. Role of imiquimod and parenteral meglumine antimoniate in the initial treatment of cutaneous leishmaniasis. Clinical infectious diseases : an official publication of the Infectious Diseases Society of America 2007; 44(12): 1549-54.
229. Machado PR, Lessa H, Lessa M, et al. Oral pentoxifylline combined with pentavalent antimony: a randomized trial for mucosal leishmaniasis. Clinical infectious diseases : an official publication of the Infectious Diseases Society of America 2007; 44(6): 788-93.
230. Lessa HA, Machado P, Lima F, et al. Successful treatment of refractory mucosal leishmaniasis with pentoxifylline plus antimony. The American journal of tropical medicine and hygiene 2001; 65(2): 87-9.
231. de Oliveira-Neto MP, Mattos Mda S. An alternative antimonial schedule to be used in cutaneous leishmaniasis when high doses of antimony are undesirable. Revista da Sociedade Brasileira de Medicina Tropical 2006; 39(4): 323-6.
232. Almeida RP, Brito J, Machado PL, et al. Successful treatment of refractory cutaneous leishmaniasis with GM-CSF and antimonials. The American journal of tropical medicine and hygiene 2005; 73(1): 79-81.
233. Hervas JA, Martin-Santiago A, Hervas D, et al. Old world Leishmania infantum cutaneous leishmaniasis unresponsive to liposomal amphotericin B treated with topical imiquimod. The Pediatric infectious disease journal 2012; 31(1): 97-100.
234. Obonaga R, Fernandez OL, Valderrama L, et al. Treatment failure and miltefosine susceptibility in dermal leishmaniasis caused by leishmania subgenus viannia species. Antimicrobial Agents and Chemotherapy 2014; 58(1): 144-52.
235. Gunduz K, Afsar S, Ayhan S, et al. Recidivans cutaneous leishmaniasis unresponsive to liposomal amphotericin B (AmBisome). Journal of the European Academy of Dermatology and Venereology : JEADV 2000; 14(1): 11-3.
236. Purkait B, Singh R, Wasnik K, et al. Up-regulation of silent information regulator 2 (Sir2) is associated with amphotericin B resistance in clinical isolates of Leishmania donovani. The Journal of antimicrobial chemotherapy 2015; 70(5): 1343-56.
237. Pimentel MI, Baptista C, Rubin EF, et al. American cutaneous leishmaniasis caused by Leishmania (Viannia) braziliensis resistant to meglumine antimoniate, but with good response to pentamidine: a case report. Revista da Sociedade Brasileira de Medicina Tropical 2011; 44(2): 254-6.
238. Arevalo J, Ramirez L, Adaui V, et al. Influence of Leishmania (Viannia) species on the response to antimonial treatment in patients with American tegumentary leishmaniasis. The Journal of infectious diseases 2007; 195(12): 1846-51.
239. Hadighi R, Mohebali M, Boucher P, Hajjaran H, Khamesipour A, Ouellette M. Unresponsiveness to Glucantime treatment in Iranian cutaneous leishmaniasis due to drug-resistant Leishmania tropica parasites. PLoS Med 2006; 3(5): e162.
240. Utaile M, Kassahun A, Abebe T, Hailu A. Susceptibility of clinical isolates of Leishmania aethiopica to miltefosine, paromomycin, amphotericin B and sodium stibogluconate using amastigote-macrophage in vitro model. Experimental parasitology 2013; 134(1): 68-75.
241. Bafica A, Oliveira F, Freitas LA, Nascimento EG, Barral A. American cutaneous leishmaniasis unresponsive to antimonial drugs: successful treatment using combination of N-methilglucamine antimoniate plus pentoxifylline. International Journal of Dermatology 2003; 42(3): 203-7.
242. Arevalo I, Ward B, Miller R, et al. Successful treatment of drug-resistant cutaneous leishmaniasis in humans by use of imiquimod, an immunomodulator. Clinical infectious diseases : an official publication of the Infectious Diseases Society of America 2001; 33(11): 1847-51.
243. Baum KF, Berens RL. Successful treatment of cutaneous leishmaniasis with allopurinol after failure of treatment with ketoconazole. Clinical infectious diseases : an official publication of the Infectious Diseases Society of America 1994; 18(5): 813-5.
244. Gonzalez U, Pinart M, Rengifo-Pardo M, Macaya A, Alvar J, Tweed JA. Interventions for American cutaneous and mucocutaneous leishmaniasis. The Cochrane database of systematic reviews 2009; (2): CD004834.
245. Machado PR, Ho J, Bang H, Guimarães LH, Carvalho EM. Reply to Ojha et al. Clinical Infectious Diseases 2007; 45(8): 1.
246. Amato VS, Tuon FF, Bacha HA, Neto VA, Nicodemo AC. Mucosal leishmaniasis. Current scenario and prospects for treatment. Acta Tropica 2008; 105(1): 1-9.
247. Oliveira-Neto MP, Mattos M, Pirmez C, et al. Mucosal leishmaniasis ("espundia") responsive to low dose of N-methyl glucamine (Glucantime) in Rio de Janeiro, Brazil. Revista do Instituto de Medicina Tropical de Sao Paulo 2000; 42(6): 321-5.
248. Llanos-Cuentas A, Echevarria J, Cruz M, et al. Efficacy of sodium stibogluconate alone and in combination with allopurinol for treatment of mucocutaneous leishmaniasis. Clinical infectious diseases : an official publication of the Infectious Diseases Society of America 1997; 25(3): 677-84.
249. Costa JM, Netto EM, Marsden PD. Acute airway obstruction due to oedema of the larynx following antimony therapy in mucosal leishmaniasis. Revista da Sociedade Brasileira de Medicina Tropical 1986; 19(2): 109.
250. Rocha RA, Sampaio RN, Guerra M, et al. Apparent Glucantime failure in five patients with mucocutaneous leishmaniasis. J Trop Med Hyg 1980; 83(4): 131-9.
251. Keith PJ, Wetter DA, Wilson JW, Lehman JS. Evidence-based guidelines for laboratory screening for infectious diseases before initiation of systemic immunosuppressive agents in patients with autoimmune bullous dermatoses. The British journal of dermatology 2014; 171(6): 1307-17.
252. Shafran DM, Bunce PE, Gold WL. Reducing the risk of infection in a 74-year-old man who is to receive prednisone. CMAJ : Canadian Medical Association journal = journal de l'Association medicale canadienne 2014; 186(16): 1239-40.
253. Ghosh K. Strongyloides stercoralis septicaemia following steroid therapy for eosinophilia: report of three cases. Transactions of the Royal Society of Tropical Medicine and Hygiene 2007; 101(11): 1163-5.
254. Mejia R, Nutman TB. Screening, prevention, and treatment for hyperinfection syndrome and disseminated infections caused by Strongyloides stercoralis. Current Opinion in Infectious Diseases 2012; 25(4): 458-63.
255. Amato VS, Tuon FF, Siqueira AM, Nicodemo AC, Neto VA. Treatment of mucosal leishmaniasis in Latin America: systematic review. The American journal of tropical medicine and hygiene 2007; 77(2): 266-74.
256. Saenz RE, de Rodriguez CG, Johnson CM, Berman JD. Efficacy and toxicity of pentostam against Panamanian mucosal leishmaniasis. The American journal of tropical medicine and hygiene 1991; 44(4): 394-8.
257. Franke ED, Wignall FS, Cruz ME, et al. Efficacy and toxicity of sodium stibogluconate for mucosal leishmaniasis. Ann Intern Med 1990; 113(12): 934-40.
258. Franke ED, Llanos-Cuentas A, Echevarria J, et al. Efficacy of 28-day and 40-day regimens of sodium stibogluconate (Pentostam) in the treatment of mucosal leishmaniasis. The

American journal of tropical medicine and hygiene 1994; 51(1): 77-82.
259. Brito G, Dourado M, Polari L, et al. Clinical and immunological outcome in cutaneous leishmaniasis patients treated with pentoxifylline. The American journal of tropical medicine and hygiene 2014; 90(4): 617-20.
260. Sampaio SA, Castro RM, Dillon NL, Martins JE. Treatment of mucocutaneous (American) leishmaniasis with amphotericin B: report of 70 cases. International Journal of Dermatology 1971; 10(3): 179-81.
261. Rodriguez LV, Dedet JP, Paredes V, Mendoza C, Cardenas F. A randomized trial of amphotericin B alone or in combination with itraconazole in the treatment of mucocutaneous leishmaniasis. Memorias do Instituto Oswaldo Cruz 1995; 90(4): 525-8.
262. Soto J, Toledo J, Valda L, et al. Treatment of Bolivian mucosal leishmaniasis with miltefosine. Clinical infectious diseases : an official publication of the Infectious Diseases Society of America 2007; 44(3): 350-6.
263. Rocio C, Amato VS, Camargo RA, Tuon FF, Nicodemo AC. Liposomal formulation of amphotericin B for the treatment of mucosal leishmaniasis in HIV-negative patients. Transactions of the Royal Society of Tropical Medicine and Hygiene 2014; 108(3): 176-8.
264. Amato VS, Tuon FF, Camargo RA, Souza RM, Santos CR, Nicodemo AC. Can we use a lower dose of liposomal amphotericin B for the treatment of mucosal American leishmaniasis? The American journal of tropical medicine and hygiene 2011; 85(5): 818-9.
265. Amato VS, Tuon FF, Campos A, et al. Treatment of mucosal leishmaniasis with a lipid formulation of amphotericin B. Clinical infectious diseases : an official publication of the Infectious Diseases Society of America 2007; 44(2): 311-2.
266. Lambertucci JR, Silva LC. Mucocutaneous leishmaniasis treated with liposomal amphotericin B. Revista da Sociedade Brasileira de Medicina Tropical 2008; 41(1): 87-8.
267. Sampaio RN, Marsden PD. [Treatment of the mucosal form of leishmaniasis without response to glucantime, with liposomal amphotericin B]. Revista da Sociedade Brasileira de Medicina Tropical 1997; 30(2): 125-8.
268. Cunha MA, Leao AC, de Cassia Soler R, Lindoso JA. Efficacy and Safety of Liposomal Amphotericin B for the Treatment of Mucosal Leishmaniasis from the New World: A Retrospective Study. The American journal of tropical medicine and hygiene 2015; 93(6): 1214-8.
269. Soto J, Rea J, Valderrama M, et al. Efficacy of extended (six weeks) treatment with miltefosine for mucosal leishmaniasis in Bolivia. The American journal of tropical medicine and hygiene 2009; 81(3): 387-9.
270. Garcia Bustos MF, Barrio A, Parodi C, Beckar J, Moreno S, Basombrio MA. [Miltefosine versus meglumine antimoniate in the treatment of mucosal leishmaniasis]. Medicina (B Aires) 2014; 74(5): 371-7.
271. Briefing Document for the Anti-Infective Drugs Advisory Committee Meeting F. Impavido® (miltefosine) capsules for the treatment of visceral, mucosal and cutaneous leishmaniasis. Meeting date, 18 Oct 2013. Accessed 2 January 2016 at: http://www.fda.gov/downloads/advisorycommittees/committeesmeetingmaterials/drugs/anti-infectivedrugsadvisorycommittee/ucm371075.pdf. Anti-infective Drugs Advisory Committee Meeting Materials Advisory Committee Briefing Book 2013.
272. Llanos-Cuentas A, Echevarria J, Seas C, et al. Parenteral aminosidine is not effective for Peruvian mucocutaneous leishmaniasis. The American journal of tropical medicine and hygiene 2007; 76(6): 1128-31.
273. Romero GA, Lessa HA, Orge MG, Macedo VO, Marsden PD. [Treatment of mucosal leishmaniasis with aminosidine sulfate: results of two year follow-up]. Revista da Sociedade Brasileira de Medicina Tropical 1998; 31(6): 511-6.
274. Tuon FF, Gomes-Silva A, Da-Cruz AM, Duarte MI, Neto VA, Amato VS. Local immunological factors associated with recurrence of mucosal leishmaniasis. Clin Immunol 2008; 128(3): 442-6.
275. Hasker E, Malaviya P, Gidwani K, et al. Strong association between serological status and probability of progression to clinical visceral leishmaniasis in prospective cohort studies in India and Nepal. PLoS neglected tropical diseases 2014; 8(1): e2657.
276. Monge-Maillo B, Lopez-Velez R. Therapeutic options for visceral leishmaniasis. Drugs 2013; 73(17): 1863-88.
277. Berman J. Amphotericin B formulations and other drugs for visceral leishmaniasis. The American journal of tropical medicine and hygiene 2015; 92(3): 471-3.
278. Saha AK, Mukherjee T, Bhaduri A. Mechanism of action of amphotericin B on Leishmania donovani promastigotes. Molecular and biochemical parasitology 1986; 19(3): 195-200.
279. Janknegt R, de Marie S, Bakker-Woudenberg IA, Crommelin DJ. Liposomal and lipid formulations of amphotericin B. Clinical pharmacokinetics. Clin Pharmacokinet 1992; 23(4): 279-91.
280. Adler-Moore J, Proffitt RT. Effect of tissue penetration on AmBisome efficacy. Curr Opin Investig Drugs 2003; 4(2): 179-85.
281. Gangneux JP, Sulahian A, Garin YJ, Farinotti R, Derouin F. Therapy of visceral leishmaniasis due to Leishmania infantum: experimental assessment of efficacy of AmBisome. Antimicrobial Agents and Chemotherapy 1996; 40(5): 1214-8.
282. Gradoni L, Davidson RN, Orsini S, Betto P, Giambenedetti M. Activity of liposomal amphotericin B (AmBisome) against Leishmania infantum and tissue distribution in mice. Journal of Drug Targeting 1993; 1(4): 311-6.
283. Davidson RN, Di Martino L, Gradoni L, et al. Liposomal amphotericin B (AmBisome) in Mediterranean visceral leishmaniasis: a multi-centre trial. Q J Med 1994; 87(2): 75-81.
284. Meyerhoff A. U.S. Food and Drug Administration approval of AmBisome (liposomal amphotericin B) for treatment of visceral leishmaniasis. Clinical infectious diseases : an official publication of the Infectious Diseases Society of America 1999; 28(1): 42-8; discussion 9-51.
285. Seaman J, Boer C, Wilkinson R, et al. Liposomal amphotericin B (AmBisome) in the treatment of complicated kala-azar under field conditions. Clinical infectious diseases : an official publication of the Infectious Diseases Society of America 1995; 21(1): 188-93.
286. Burza S, Sinha PK, Mahajan R, et al. Post Kala-Azar Dermal Leishmaniasis following Treatment with 20 mg/kg Liposomal Amphotericin B (Ambisome) for Primary Visceral Leishmaniasis in Bihar, India. PLoS Neglected Tropical Diseases 2014; 8(1): 49.
287. Davidson RN, di Martino L, Gradoni L, et al. Short-course treatment of visceral leishmaniasis with liposomal amphotericin B (AmBisome). Clinical infectious diseases : an official publication of the Infectious Diseases Society of America 1996; 22(6): 938-43.
288. Syriopoulou V, Daikos GL, Theodoridou M, et al. Two doses of a lipid formulation of amphotericin B for the treatment of Mediterranean visceral leishmaniasis. Clinical infectious diseases : an official publication of the Infectious Diseases Society of America 2003; 36(5): 560-6.
289. Freire M, Badaro F, Avelar ME, et al. Efficacy and Tolerability of Liposomal Amphotericin B (Ambisome) in the Treatment of Visceral Leishmaniasis in Brazil. The Brazilian journal of infectious diseases : an official publication of the Brazilian Society of Infectious Diseases 1997; 1(5): 230-40.
290. Khalil EAG, Weldegebreal T, Younis BM, et al. Safety and Efficacy of Single Dose versus Multiple Doses of AmBisome (registered trademark) for Treatment of Visceral Leishmaniasis in Eastern Africa: A Randomised Trial. PLoS Neglected Tropical Diseases 2014; 8(1): 21.
291. Salih NAW, van Griensven J, Chappuis F, et al. Liposomal amphotericin B for complicated visceral leishmaniasis (kala-azar) in eastern Sudan: How effective is treatment for this neglected disease? Tropical Medicine and International Health 2014; 19(2): 146-52.
292. Mueller YK, Kolaczinski JH, Koech T, et al. Clinical epidemiology, diagnosis and treatment of visceral leishmaniasis in the Pokot endemic area of Uganda and Kenya. American Journal of Tropical Medicine and Hygiene 2014; 90(1): 33-9.
293. Dietze R, Milan EP, Berman JD, et al. Treatment of Brazilian kala-azar with a short course of amphocil (amphotericin B cholesterol dispersion). Clinical infectious diseases : an official

publication of the Infectious Diseases Society of America 1993; 17(6): 981-6.
294. Sundar S, Agrawal NK, Sinha PR, Horwith GS, Murray HW. Short-course, low-dose amphotericin B lipid complex therapy for visceral leishmaniasis unresponsive to antimony. Ann Intern Med 1997; 127(2): 133-7.
295. Gaeta GB, Maisto A, Di Caprio D, et al. Efficacy of amphotericin B colloidal dispersion in the treatment of Mediterranean visceral leishmaniasis in immunocompetent adult patients. Scandinavian journal of infectious diseases 2000; 32(6): 675-7.
296. Dietze R, Fagundes SM, Brito EF, et al. Treatment of kala-azar in Brazil with Amphocil (amphotericin B cholesterol dispersion) for 5 days. Transactions of the Royal Society of Tropical Medicine and Hygiene 1995; 89(3): 309-11.
297. Sundar S, Goyal AK, Mandal AK, Makharia MK, Singh VP, Murray HW. Amphotericin B lipid complex in the management of antimony unresponsive Indian visceral leishmaniasis. J Assoc Physicians India 1999; 47(2): 186-8.
298. Sundar S, Goyal AK, More DK, Singh MK, Murray HW. Treatment of antimony-unresponsive Indian visceral leishmaniasis with ultra-short courses of amphotericin-B-lipid complex. Ann Trop Med Parasitol 1998; 92(7): 755-64.
299. Sundar S, Mehta H, Chhabra A, et al. Amphotericin B colloidal dispersion for the treatment of Indian visceral leishmaniasis. Clinical infectious diseases : an official publication of the Infectious Diseases Society of America 2006; 42(5): 608-13.
300. Sundar S, Mehta H, Suresh AV, Singh SP, Rai M, Murray HW. Amphotericin B treatment for Indian visceral leishmaniasis: conventional versus lipid formula-tions. Clin Infect Dis 38: 377–83.
301. Thakur CP, Narayan S. A comparative evaluation of amphotericin B and sodium antimony gluconate, as first-line drugs in the treatment of Indian visceral leishmaniasis. Ann Trop Med Parasitol 2004; 98(2): 129-38.
302. Thakur CP, Sinha GP, Pandey AK, Barat D, Singh RK. Daily versus alternate-day regimen of amphotericin B in the treatment of kala-azar: a randomized comparison. Bull World Health Organ 1994; 72(6): 931-6.
303. Olliaro PL, Guerin PJ, Gerstl S, Haaskjold AA, Rottingen JA, Sundar S. Treatment options for visceral leishmaniasis: a systematic review of clinical studies done in India, 1980-2004. The Lancet Infectious diseases 2005; 5(12): 763-74.
304. Mishra M, Biswas UK, Jha AM, Khan AB. Amphotericin versus sodium stibogluconate in first-line treatment of Indian kala-azar. Lancet 1994; 344(8937): 1599-600.
305. Sundar S, Chakravarty J, Rai VK, et al. Amphotericin B treatment for Indian visceral leishmaniasis: response to 15 daily versus alternate-day infusions. Clinical infectious diseases : an official publication of the Infectious Diseases Society of America 2007; 45(5): 556-61.
306. Sundar S, Jha TK, Thakur CP, Sinha PK, Bhattacharya SK. Injectable paromomycin for Visceral leishmaniasis in India. N Engl J Med 2007; 356(25): 2571-81.
307. Szebeni J. Complement activation-related pseudoallergy: a new class of drug-induced acute immune toxicity. Toxicology 2005; 216(2-3): 106-21.
308. Roden MM, Nelson LD, Knudsen TA, et al. Triad of acute infusion-related reactions associated with liposomal amphotericin B: analysis of clinical and epidemiological characteristics. Clinical infectious diseases : an official publication of the Infectious Diseases Society of America 2003; 36(10): 1213-20.
309. Farmakiotis D, Tverdek FP, Kontoyiannis DP. The safety of amphotericin B lipid complex in patients with prior severe intolerance to liposomal amphotericin B. Clinical infectious diseases : an official publication of the Infectious Diseases Society of America 2013; 56(5): 701-3.
310. Bhattacharya SK, Sinha PK, Sundar S, et al. Phase 4 trial of miltefosine for the treatment of Indian visceral leishmaniasis. The Journal of infectious diseases 2007; 196(4): 591-8.
311. Singh UK, Prasad R, Mishra OP, Jayswal BP. Miltefosine in children with visceral leishmaniasis: a prospective, multicentric, cross-sectional study. Indian J Pediatr 2006; 73(12): 1077-80.
312. Sundar S, Jha TK, Sindermann H, Junge K, Bachmann P, Berman J. Oral miltefosine treatment in children with mild to moderate Indian visceral leishmaniasis. The Pediatric infectious disease journal 2003; 22(5): 434-8.
313. Monge-Maillo B, Lopez-Velez R. Miltefosine for visceral and cutaneous leishmaniasis: drug characteristics and evidence-based treatment recommendations. Clinical infectious diseases : an official publication of the Infectious Diseases Society of America 2015; 60(9): 1398-404.
314. Sundar S, Jha TK, Thakur CP, et al. Oral miltefosine for Indian visceral leishmaniasis. N Engl J Med 2002; 347(22): 1739-46.
315. Sundar S, Rosenkaimer F, Makharia MK, et al. Trial of oral miltefosine for visceral leishmaniasis. Lancet 1998; 352(9143): 1821-3.
316. Sundar S, More DK, Singh MK, et al. Failure of pentavalent antimony in visceral leishmaniasis in India: report from the center of the Indian epidemic. Clinical infectious diseases : an official publication of the Infectious Diseases Society of America 2000; 31(4): 1104-7.
317. Collin S, Davidson R, Ritmeijer K, et al. Conflict and kala-azar: determinants of adverse outcomes of kala-azar among patients in southern Sudan. Clinical infectious diseases : an official publication of the Infectious Diseases Society of America 2004; 38(5): 612-9.
318. Jha TK, Sundar S, Thakur CP, et al. Miltefosine, an oral agent, for the treatment of Indian visceral leishmaniasis. N Engl J Med 1999; 341(24): 1795-800.
319. Ritmeijer K, Dejenie A, Assefa Y, et al. A comparison of miltefosine and sodium stibogluconate for treatment of visceral leishmaniasis in an Ethiopian population with high prevalence of HIV infection. Clinical infectious diseases : an official publication of the Infectious Diseases Society of America 2006; 43(3): 357-64.
320. Carnielli JB, de Andrade HM, Pires SF, et al. Proteomic analysis of the soluble proteomes of miltefosine-sensitive and -resistant Leishmania infantum chagasi isolates obtained from Brazilian patients with different treatment outcomes. J Proteomics 2014; 108: 198-208.
321. Prajapati VK, Mehrotra S, Gautam S, Rai M, Sundar S. In vitro antileishmanial drug susceptibility of clinical isolates from patients with Indian visceral leishmaniasis–status of newly introduced drugs. Am J Trop Med Hyg 2012; 87(4): 655-7.
322. Bhandari V, Kulshrestha A, Deep DK, et al. Drug susceptibility in Leishmania isolates following miltefosine treatment in cases of visceral leishmaniasis and post kala-azar dermal leishmaniasis. PLoS neglected tropical diseases 2012; 6(5): e1657.
323. Rijal S, Ostyn B, Uranw S, et al. Increasing failure of miltefosine in the treatment of kala-azar in nepal and the potential role of parasite drug resistance, reinfection, or noncompliance. Clinical Infectious Diseases 2013; 56(11): 1530-8.
324. Sundar S, Singh A, Rai M, et al. Efficacy of miltefosine in the treatment of visceral leishmaniasis in India after a decade of use. Clinical Infectious Diseases 2012; 55(4): 543-50.
325. Rijal S, Chappuis F, Singh R, et al. Treatment of visceral leishmaniasis in south-eastern Nepal: decreasing efficacy of sodium stibogluconate and need for a policy to limit further decline. Transactions of the Royal Society of Tropical Medicine and Hygiene 2003; 97(3): 350-4.
326. Musa A, Khalil E, Hailu A, et al. Sodium stibogluconate (ssg) & paromomycin combination compared to ssg for visceral leishmaniasis in east africa: A randomised controlled trial. PLoS Neglected Tropical Diseases 2012; 6(6).
327. Brustoloni YM, Cunha RV, Consolo LZ, Oliveira AL, Dorval ME, Oshiro ET. Treatment of visceral leishmaniasis in children in the Central-West Region of Brazil. Infection 2010; 38(4): 261-7.
328. de Melo EC, Fortaleza CM. Challenges in the therapy of visceral leishmaniasis in Brazil: a public health perspective. Journal of Tropical Medicine 2013; 2013: 319234.
329. Mishra M, Biswas UK, Jha DN, Khan AB. Amphotericin versus pentamidine in antimony-unresponsive kala-azar. Lancet 1992; 340(8830): 1256-7.
330. Thakur CP, Kumar M, Pandey AK. Comparison of regimes of treatment of antimony-resistant kala-azar patients: a

randomized study. The American journal of tropical medicine and hygiene 1991; 45(4): 435-41.
331. Sundar S, Singh VP, Agrawal NK, Gibbs DL, Murray HW. Treatment of kala-azar with oral fluconazole. Lancet 1996; 348(9027): 614.
332. Bechade D, Seurat L, Discamps G, Taniere PH, Du Bourguet F. [Multiple digestive involvement in visceral leishmaniasis in a patient with HIV infection: favourable course with itraconazole]. Rev Med Interne 1996; 17(3): 234-7.
333. Maru M. Clinical and laboratory features and treatment of visceral leishmaniasis in hospitalized patients in Northwestern Ethiopia. The American journal of tropical medicine and hygiene 1979; 28(1): 15-8.
334. Kager PA, Rees PH, Manguyu FM, et al. Clinical, haematological and parasitological response to treatment of visceral leishmaniasis in Kenya. A study of 64 patients. Trop Geogr Med 1984; 36(1): 21-35.
335. Zijlstra EE. The treatment of kala-azar: old and new options. Trop Geogr Med 1992; 44(3): 288.
336. Caldas AJ, Costa J, Aquino D, Silva AA, Barral-Netto M, Barral A. Are there differences in clinical and laboratory parameters between children and adults with American visceral leishmaniasis? Acta Tropica 2006; 97(3): 252-8.
337. Cascio A, di Martino L, Occorsio P, et al. A 6 day course of liposomal amphotericin B in the treatment of infantile visceral leishmaniasis: the Italian experience. The Journal of antimicrobial chemotherapy 2004; 54(1): 217-20.
338. Berman JD, Badaro R, Thakur CP, et al. Efficacy and safety of liposomal amphotericin B (AmBisome) for visceral leishmaniasis in endemic developing countries. Bull World Health Organ 1998; 76(1): 25-32.
339. Sundar S, Chakravarty J, Agarwal D, Rai M, Murray HW. Single-dose liposomal amphotericin B for visceral leishmaniasis in India. N Engl J Med 2010; 362(6): 504-12.
340. di Martino L, Davidson RN, Giacchino R, et al. Treatment of visceral leishmaniasis in children with liposomal amphotericin B. The Journal of pediatrics 1997; 131(2): 271-7.
341. Nyakundi PM, Wasunna KM, Rashid JR, et al. Is one year follow-up justified in kala-azar post-treatment? East Afr Med J 1994; 71(7): 453-9.
342. Sundar S, Agrawal G, Rai M, Makharia MK, Murray HW. Treatment of Indian visceral leishmaniasis with single or daily infusions of low dose liposomal amphotericin B: randomised trial. BMJ (Clinical research ed) 2001; 323(7310): 419-22.
343. Sundar S, Rai M, Chakravarty J, et al. New treatment approach in Indian visceral leishmaniasis: single-dose liposomal amphotericin B followed by short-course oral miltefosine. Clinical infectious diseases : an official publication of the Infectious Diseases Society of America 2008; 47(8): 1000-6.
344. Werneck GL, Batista MS, Gomes JR, Costa DL, Costa CH. Prognostic factors for death from visceral leishmaniasis in Teresina, Brazil. Infection 2003; 31(3): 174-7.
345. Seaman J, Mercer AJ, Sondorp HE, Herwaldt BL. Epidemic visceral leishmaniasis in southern Sudan: treatment of severely debilitated patients under wartime conditions and with limited resources. Ann Intern Med 1996; 124(7): 664-72.
346. Mueller Y, Mbulamberi DB, Odermatt P, Hoffmann A, Loutan L, Chappuis F. Risk factors for in-hospital mortality of visceral leishmaniasis patients in eastern Uganda. Tropical medicine & international health : TM & IH 2009; 14(8): 910-7.
347. Sampaio MJ, Cavalcanti NV, Alves JG, Filho MJ, Correia JB. Risk factors for death in children with visceral leishmaniasis. PLoS neglected tropical diseases 2010; 4(11): e877.
348. Antinori S, Longhi E, Bestetti G, et al. Post-kala-azar dermal leishmaniasis as an immune reconstitution inflammatory syndrome in a patient with acquired immune deficiency syndrome. The British journal of dermatology 2007; 157(5): 1032-6.
349. Maurya R, Singh RK, Kumar B, Salotra P, Rai M, Sundar S. Evaluation of PCR for diagnosis of Indian kala-azar and assessment of cure. Journal of clinical microbiology 2005; 43(7): 3038-41.
350. Cascio A, Calattini S, Colomba C, et al. Polymerase chain reaction in the diagnosis and prognosis of Mediterranean visceral leishmaniasis in immunocompetent children. Pediatrics 2002; 109(2): E27.
351. Fisa R, Riera C, Ribera E, Gallego M, Portus M. A nested polymerase chain reaction for diagnosis and follow-up of human visceral leishmaniasis patients using blood samples. Transactions of the Royal Society of Tropical Medicine and Hygiene 2002; 96 Suppl 1: S191-4.
352. Disch J, Oliveira MC, Orsini M, Rabello A. Rapid clearance of circulating Leishmania kinetoplast DNA after treatment of visceral leishmaniasis. Acta tropica 2004; 92(3): 279-83.
353. Nuzum E, White F 3rd, Thakur C, et al. Diagnosis of symptomatic visceral leishmaniasis by use of the polymerase chain reaction on patient blood. The Journal of infectious diseases 1995; 171(3): 751-4.
354. Houghton RL, Petrescu M, Benson DR, et al. A cloned antigen (recombinant K39) of Leishmania chagasi diagnostic for visceral leishmaniasis in human immunodeficiency virus type 1 patients and a prognostic indicator for monitoring patients undergoing drug therapy. The Journal of infectious diseases 1998; 177(5): 1339-44.
355. Bern C, Jha SN, Joshi AB, Thakur GD, Bista MB. Use of the recombinant K39 dipstick test and the direct agglutination test in a setting endemic for visceral leishmaniasis in Nepal. Am J Trop Med Hyg 2000; 63(3-4): 153-7.
356. De Almeida Silva L, Romero HD, Prata A, et al. Immunologic tests in patients after clinical cure of visceral leishmaniasis. The American journal of tropical medicine and hygiene 2006; 75(4): 739-43.
357. Zijlstra EE, Musa AM, Khalil EA, el-Hassan IM, el-Hassan AM. Post-kala-azar dermal leishmaniasis. The Lancet Infectious diseases 2003; 3(2): 87-98.
358. Mueller YK, Nackers F, Ahmed KA, et al. Burden of visceral leishmaniasis in villages of eastern Gedaref State, Sudan: an exhaustive cross-sectional survey. PLoS Neglected Tropical Diseases 2012; 6(11): e1872.
359. Ramesh V, Kaushal H, Mishra AK, Singh R, Salotra P. Clinico-epidemiological analysis of Post kala-azar dermal leishmaniasis (PKDL) cases in India over last two decades: a hospital based retrospective study. BMC Public Health 2015; 15: 1092.
360. Ganguly S, Saha P, Chatterjee M, et al. PKDL–A Silent Parasite Pool for Transmission of Leishmaniasis in Kala-azar Endemic Areas of Malda District, West Bengal, India. PLoS Neglected Tropical Diseases 2015; 9(10): e0004138.
361. WHO. The Post Kala-azar Dermal Leishmaniasis (PKDL) Atlas A Manual for Health Workers. Accessed on February 16, 2016 at http://apps.who.int/iris/bitstream/10665/101164/1/9789241504102_eng.pdf. 2011.
362. Murray HW. Leishmaniasis in the United States: treatment in 2012. Am J Trop Med Hyg 2012; 86(3): 434-40.
363. Lachaud L, Bourgeois N, Plourde M, Leprohon P, Bastien P, Ouellette M. Parasite susceptibility to amphotericin B in failures of treatment for visceral leishmaniasis in patients coinfected with HIV type 1 and Leishmania infantum. Clinical infectious diseases : an official publication of the Infectious Diseases Society of America 2009; 48(2): e16-22.
364. Alexandrino-de-Oliveira P, Santos-Oliveira JR, Dorval ME, et al. HIV/AIDS-associated visceral leishmaniasis in patients from an endemic area in Central-west Brazil. Memorias do Instituto Oswaldo Cruz 2010; 105(5): 692-7.
365. Ritmeijer K, ter Horst R, Chane S, et al. Limited effectiveness of high-dose liposomal amphotericin B (AmBisome) for treatment of visceral leishmaniasis in an Ethiopian population with high HIV prevalence. Clinical infectious diseases : an official publication of the Infectious Diseases Society of America 2011; 53(12): e152-8.
366. Sinha PK, van Griensven J, Pandey K, et al. Liposomal amphotericin B for visceral leishmaniasis in human immunodeficiency virus-coinfected patients: 2-year treatment outcomes in Bihar, India. Clinical infectious diseases : an official publication of the Infectious Diseases Society of America 2011; 53(7): e91-8.
367. Burza S, Sinha PK, Mahajan R, et al. Five-Year Field Results and Long-Term Effectiveness of 20 mg/kg Liposomal Amphotericin B (Ambisome) for Visceral Leishmaniasis in Bihar, India. PLoS Neglected Tropical Diseases 2014; 8(1): 46.
368. Burza S, Mahajan R, Sinha PK, et al. Visceral leishmaniasis and HIV co-infection in Bihar, India: long-term effectiveness

and treatment outcomes with liposomal amphotericin B (AmBisome). PLoS Neglected Tropical Diseases 2014; 8(8): e3053.
369. Laguna F, Torre-Cisneros J, Moreno V, Villanueva JL, Valencia E. Efficacy of intermittent liposomal amphotericin B in the treatment of visceral leishmaniasis in patients infected with human immunodeficiency virus. Clinical infectious diseases : an official publication of the Infectious Diseases Society of America 1995; 21(3): 711-2.
370. Russo R, Nigro LC, Minniti S, et al. Visceral leishmaniasis in HIV infected patients: treatment with high dose liposomal amphotericin B (AmBisome). J Infect 1996; 32(2): 133-7.
371. Laguna F. Treatment of leishmaniasis in HIV-positive patients. Ann Trop Med Parasitol 2003; 97 Suppl 1: 135-42.
372. Bern C, Adler-Moore J, Berenguer J, et al. Liposomal amphotericin B for the treatment of visceral leishmaniasis. Clinical infectious diseases : an official publication of the Infectious Diseases Society of America 2006; 43(7): 917-24.
373. Cota GF, de Sousa MR, Fereguetti TO, Rabello A. Efficacy of anti-leishmania therapy in visceral leishmaniasis among HIV infected patients: a systematic review with indirect comparison. PLoS Neglected Tropical Diseases 2013; 7(5): e2195.
374. Delgado J, Macias J, Pineda JA, et al. High frequency of serious side effects from meglumine antimoniate given without an upper limit dose for the treatment of visceral leishmaniasis in human immunodeficiency virus type-1-infected patients. The American journal of tropical medicine and hygiene 1999; 61(5): 766-9.
375. Diro E, Lynen L, Mohammed R, Boelaert M, Hailu A, van Griensven J. High parasitological failure rate of visceral leishmaniasis to sodium stibogluconate among HIV co-infected adults in Ethiopia. PLoS Neglected Tropical Diseases 2014; 8(5): e2875.
376. Laguna F, Lopez-Velez R, Pulido F, et al. Treatment of visceral leishmaniasis in HIV-infected patients: a randomized trial comparing meglumine antimoniate with amphotericin B. Spanish HIV-Leishmania Study Group. Aids 1999; 13(9): 1063-9.
377. Laguna F, Videla S, Jimenez-Mejias ME, et al. Amphotericin B lipid complex ver-sus meglumine antimoniate in the treatment of visceral leishmaniasis in patients infected with HIV: a randomized pilot study. J Antimicrob Chemother 2003; 52:464–8.
378. Sindermann H, Engel KR, Fischer C, Bommer W. Oral miltefosine for leishmaniasis in immunocompromised patients: compassionate use in 39 patients with HIV infection. Clinical infectious diseases : an official publication of the Infectious Diseases Society of America 2004; 39(10): 1520-3.
379. Troya J, Casquero A, Refoyo E, Fernandez-Guerrero ML, Gorgolas M. Long term failure of miltefosine in the treatment of refractory visceral leishmaniasis in AIDS patients. Scandinavian journal of infectious diseases 2008; 40(1): 78-80.
380. Marques N, Sa R, Coelho F, Oliveira J, Saraiva Da Cunha J, Melico-Silvestre A. Miltefosine for visceral leishmaniasis relapse treatment and secondary prophylaxis in HIV-infected patients. Scandinavian journal of infectious diseases 2008; 40(6-7): 523-6.
381. Diro E, Lynen L, Ritmeijer K, Boelaert M, Hailu A, van Griensven J. Visceral Leishmaniasis and HIV coinfection in East Africa. PLoS neglected tropical diseases 2014; 8(6): e2869.
382. Mahajan R, Das P, Isaakidis P, et al. Combination Treatment for Visceral Leishmaniasis Patients Coinfected with Human Immunodeficiency Virus in India. Clinical infectious diseases : an official publication of the Infectious Diseases Society of America 2015; 61(8): 1255-62.
383. Lopez-Velez R. The impact of highly active antiretroviral therapy (HAART) on visceral leishmaniasis in Spanish patients who are co-infected with HIV. Ann Trop Med Parasitol 2003; 97 Suppl 1: 143-7.
384. Russo R, Laguna F, Lopez-Velez R, et al. Visceral leishmaniasis in those infected with HIV: clinical aspects and other opportunistic infections. Ann Trop Med Parasitol 2003; 97 Suppl 1: 99-105.
385. del Giudice P, Mary-Krause M, Pradier C, et al. Impact of highly active antiretroviral therapy on the incidence of visceral leishmaniasis in a French cohort of patients infected with human immunodeficiency virus. The Journal of infectious diseases 2002; 186(9): 1366-70.
386. de la Rosa R, Pineda JA, Delgado J, et al. Influence of highly active antiretroviral therapy on the outcome of subclinical visceral leishmaniasis in human immunodeficiency virus-infected patients. Clinical infectious diseases : an official publication of the Infectious Diseases Society of America 2001; 32(4): 633-5.
387. de La Rosa R, Pineda JA, Delgado J, et al. Incidence of and risk factors for symptomatic visceral leishmaniasis among human immunodeficiency virus type 1-infected patients from Spain in the era of highly active antiretroviral therapy. Journal of clinical microbiology 2002; 40(3): 762-7.
388. Tortajada C, Perez-Cuevas B, Moreno A, et al. Highly active antiretroviral therapy (HAART) modifies the incidence and outcome of visceral leishmaniasis in HIV-infected patients. J Acquir Immune Defic Syndr 2002; 30(3): 364-6.
389. van Griensven J, Diro E, Lopez-Velez R, et al. HIV-1 protease inhibitors for treatment of visceral leishmaniasis in HIV-co-infected individuals. The Lancet infectious diseases 2013; 13(3): 251-9.
390. Dooley KE, Flexner C, Andrade AS. Drug interactions involving combination antiretroviral therapy and other anti-infective agents: repercussions for resource-limited countries. The Journal of infectious diseases 2008; 198(7): 948-61.
391. Lawn SD. Immune reconstitution disease associated with parasitic infections following initiation of antiretroviral therapy. Current Opinion in Infectious Diseases 2007; 20(5): 482-8.
392. Badaro R, Goncalves LO, Gois LL, Maia ZP, Benson C, Grassi MF. Leishmaniasis as a Manifestation of Immune Reconstitution Inflammatory Syndrome (IRIS) in HIV-Infected Patients: A Literature Review. J Int Assoc Provid AIDS Care 2015; 14(5): 402-7.
393. Albrecht H. Leishmaniosis–new perspectives on an underappreciated opportunistic infection. Aids 1998; 12(16): 2225-6.
394. Jimenez-Exposito MJ, Alonso-Villaverde C, Sarda P, Masana L. Visceral leishmaniasis in HIV-infected patients with non-detectable HIV-1 viral load after highly active antiretroviral therapy. Aids 1999; 13(1): 152-3.
395. Berry A, Abraham B, Dereure J, Pinzani V, Bastien P, Reynes J. Two case reports of symptomatic visceral leishmaniasis in AIDS patients concomitant with immune reconstitution due to antiretroviral therapy. Scandinavian journal of infectious diseases 2004; 36(3): 225-7.
396. Belay AD, Asafa Y, Mesure J, Davidson RN. Successful miltefosine treatment of post-kala-azar dermal leishmaniasis occurring during antiretroviral therapy. Ann Trop Med Parasitol 2006; 100(3): 223-7.
397. Tadesse A, Hurissa Z. Leishmaniasis (PKDL) as a case of immune reconstitution inflammatory syndrome (IRIS) in HIV-positive patient after initiation of anti-retroviral therapy (ART). Ethiop Med J 2009; 47(1): 77-9.
398. Gilad J, Borer A, Hallel-Halevy D, Riesenberg K, Alkan M, Schlaeffer F. Post-kala-azar dermal leishmaniasis manifesting after initiation of highly active anti-retroviral therapy in a patient with human immunodeficiency virus infection. Isr Med Assoc J 2001; 3(6): 451-2.
399. Bourgeois N, Bastien P, Reynes J, Makinson A, Rouanet I, Lachaud L. 'Active chronic visceral leishmaniasis' in HIV-1-infected patients demonstrated by biological and clinical long-term follow-up of 10 patients. HIV Med 2010; 11(10): 670-3.
400. Lopez-Velez R, Videla S, Marquez M, et al. Amphotericin B lipid complex versus no treatment in the secondary prophylaxis of visceral leishmaniasis in HIV-infected patients. The Journal of antimicrobial chemotherapy 2004; 53(3): 540-3.
401. Molina I, Falco V, Crespo M, et al. Efficacy of liposomal amphotericin B for secondary prophylaxis of visceral leishmaniasis in HIV-infected patients. The Journal of antimicrobial chemotherapy 2007; 60(4): 837-42.
402. Perez-Molina JA, Lopez-Velez R, Montilla P, Guerrero A. Pentamidine isethionate as secondary prophylaxis against visceral leishmaniasis in HIV-positive patients. Aids 1996; 10(2): 237-8.

403. Patel TA, Lockwood DN. Pentamidine as secondary prophylaxis for visceral leishmaniasis in the immunocompromised host: report of four cases. Tropical medicine & international health : TM & IH 2009; 14(9): 1064-70.
404. Diro E, Ritmeijer K, Boelaert M, et al. Use of Pentamidine As Secondary Prophylaxis to Prevent Visceral Leishmaniasis Relapse in HIV Infected Patients, the First Twelve Months of a Prospective Cohort Study. PLoS Neglected Tropical Diseases 2015; 9(10): e0004087.
405. Ribera E, Ocana I, de Otero J, Cortes E, Gasser I, Pahissa A. Prophylaxis of visceral leishmaniasis in human immunodeficiency virus-infected patients. The American journal of medicine 1996; 100(5): 496-501.
406. Cojean S, Houze S, Haouchine D, et al. Leishmania resistance to miltefosine associated with genetic marker. Emerging infectious diseases 2012; 18(4): 704-6.
407. Berenguer J, Cosin J, Miralles P, Lopez JC, Padilla B. Discontinuation of secondary anti-leishmania prophylaxis in HIV-infected patients who have responded to highly active antiretroviral therapy. Aids 2000; 14(18): 2946-8.
408. ter Horst R, Collin SM, Ritmeijer K, Bogale A, Davidson RN. Concordant HIV infection and visceral leishmaniasis in Ethiopia: the influence of antiretroviral treatment and other factors on outcome. Clinical infectious diseases : an official publication of the Infectious Diseases Society of America 2008; 46(11): 1702-9.
409. Villanueva JL, Alarcon A, Bernabeu-Wittel M, et al. Prospective evaluation and follow-up of European patients with visceral leishmaniasis and HIV-1 coinfection in the era of highly active antiretroviral therapy. European journal of clinical microbiology & infectious diseases : official publication of the European Society of Clinical Microbiology 2000; 19(10): 798-801.
410. Faucher B, Pomares C, Fourcade S, et al. Mucosal Leishmania infantum leishmaniasis: specific pattern in a multicentre survey and historical cases. J Infect 2011; 63(1): 76-82.
411. Aliaga L, Cobo F, Mediavilla JD, et al. Localized mucosal leishmaniasis due to Leishmania (Leishmania) infantum: clinical and microbiologic findings in 31 patients. Medicine (Baltimore) 2003; 82(3): 147-58.
412. Cascio A, Antinori S, Campisi G, Mancuso S. Oral leishmaniasis in an HIV-infected patient. European journal of clinical microbiology & infectious diseases : official publication of the European Society of Clinical Microbiology 2000; 19(8): 651-3.
413. Madeddu G, Fiori ML, Ena P, et al. Mucocutaneous leishmaniasis as presentation of HIV infection in Sardinia, insular Italy. Parasitology International 2014; 63(1): 35-6.
414. Miralles ES, Nunez M, Hilara Y, Harto A, Moreno R, Ledo A. Mucocutaneous leishmaniasis and HIV. Dermatology 1994; 189(3): 275-7.
415. Machado ES, Braga Mda P, Da Cruz AM, et al. Disseminated American muco-cutaneous leishmaniasis caused by Leishmania braziliensis braziliensis in a patient with AIDS: a case report. Memorias do Instituto Oswaldo Cruz 1992; 87(4): 487-92.
416. Sampaio RN, Salaro CP, Resende P, de Paula CD. [American cutaneous leishmaniasis associated with HIV/AIDS: report of four clinical cases]. Revista da Sociedade Brasileira de Medicina Tropical 2002; 35(6): 651-4.
417. Amato VS, Nicodemo AC, Amato JG, Boulos M, Neto VA. Mucocutaneous leishmaniasis associated with HIV infection treated successfully with liposomal amphotericin B (AmBisome). The Journal of antimicrobial chemotherapy 2000; 46(2): 341-2.
418. Amato VS, Rabello A, Rotondo-Silva A, et al. Successful treatment of cutaneous leishmaniasis with lipid formulations of amphotericin B in two immunocompromised patients. Acta Tropica 2004; 92(2): 127-32.
419. Perez C, Solias Y, Rodriguez G. [Diffuse cutaneous leishmaniasis in a patient with AIDS]. Biomedica 2006; 26(4): 485-97.
420. Schraner C, Hasse B, Hasse U, et al. Successful treatment with miltefosine of disseminated cutaneous leishmaniasis in a severely immunocompromised patient infected with HIV-1. Clinical infectious diseases : an official publication of the Infectious Diseases Society of America 2005; 40(12): e120-4.
421. Posada-Vergara MP, Lindoso JA, Tolezano JE, Pereira-Chioccola VL, Silva MV, Goto H. Tegumentary leishmaniasis as a manifestation of immune reconstitution inflammatory syndrome in 2 patients with AIDS. The Journal of infectious diseases 2005; 192(10): 1819-22.
422. Chrusciak-Talhari A, Ribeiro-Rodrigues R, Talhari C, et al. Tegumentary leishmaniasis as the cause of immune reconstitution inflammatory syndrome in a patient co-infected with human immunodeficiency virus and Leishmania guyanensis. The American journal of tropical medicine and hygiene 2009; 81(4): 559-64.
423. Sarazin E, Nacher M, Toure Y, et al. [Dermatologic manifestations associated with immune reconstitution syndrome in HIV+ patients starting HAART: a retrospective study in French Guiana]. Bulletin de la Societe de pathologie exotique 2005; 98(3): 187-92.
424. Sinha S, Fernandez G, Kapila R, Lambert WC, Schwartz RA. Diffuse cutaneous leishmaniasis associated with the immune reconstitution inflammatory syndrome. International Journal of Dermatology 2008; 47(12): 1263-70.
425. Kerob D, Bouaziz JD, Sarfati C, et al. First case of cutaneous reconstitution inflammatory syndrome associated with HIV infection and leishmaniasis. Clinical infectious diseases : an official publication of the Infectious Diseases Society of America 2006; 43(5): 664-6.
426. Blanche P, Gombert B, Rivoal O, Abad S, Salmon D, Brezin A. Uveitis due to Leishmania major as part of HAART-induced immune restitution syndrome in a patient with AIDS. Clinical infectious diseases : an official publication of the Infectious Diseases Society of America 2002; 34(9): 1279-80.
427. Gois L, Badaro R, Schooley R, Grassi MF. Immune response to Leishmania antigens in an AIDS patient with mucocutaneous leishmaniasis as a manifestation of immune reconstitution inflammatory syndrome (IRIS): a case report. BMC Infectious Diseases 2015; 15*:* 38.
428. Pavli A, Maltezou HC. Leishmaniasis, an emerging infection in travelers. Int J Infect Dis 2010; 14(12): e1032-9.
429. Gautret P, Cramer JP, Field V, et al. Infectious diseases among travellers and migrants in Europe, EuroTravNet 2010. Euro surveillance : bulletin Europeen sur les maladies transmissibles = European communicable disease bulletin *2012*; 17(26).
430. Flores-Figueroa J, Okhuysen PC, von Sonnenburg F, et al. Patterns of illness in travelers visiting Mexico and Central America: the GeoSentinel experience. Clinical infectious diseases : an official publication of the Infectious Diseases Society of America 2011; 53(6): 523-31.
431. Perez-Ayala A, Norman F, Perez-Molina JA, Herrero JM, Monge B, Lopez-Velez R. Imported leishmaniasis: a heterogeneous group of diseases. Journal of Travel Medicine 2009; 16(6): 395-401.
432. Buonomano R, Brinkmann F, Leupin N, et al. Holiday souvenirs from the Mediterranean: three instructive cases of visceral leishmaniasis. Scandinavian journal of infectious diseases 2009; 41(10): 777-81.
433. Martin-Davila P, Fortun J, Lopez-Velez R, et al. Transmission of tropical and geographically restricted infections during solid-organ transplantation. Clin Microbiol Rev 2008; 21(1): 60-96.
434. Coster LO. Parasitic infections in solid organ transplant recipients. Infectious Disease Clinics of North America 2013; 27(2): 395-427.
435. Arce A, Estirado A, Ordobas M, et al. Re-emergence of leishmaniasis in Spain: community outbreak in Madrid, Spain, 2009 to 2012. Euro surveillance : bulletin Europeen sur les maladies transmissibles = European communicable disease bulletin 2013; 18(30): 20546.
436. Larocca L, La Rosa R, Montineri A, et al. Visceral leishmaniasis in an Italian heart recipient: first case report. J Heart Lung Transplant 2007; 26(12): 1347-8.
437. Frapier JM, Abraham B, Dereure J, Albat B. Fatal visceral leishmaniasis in a heart transplant recipient. J Heart Lung Transplant 2001; 20(8): 912-3.
438. Golino A, Duncan JM, Zeluff B, et al. Leishmaniasis in a heart transplant patient. J Heart Lung Transplant 1992; 11(4 Pt 1): 820-3.

439. Morales P, Torres JJ, Salavert M, Peman J, Lacruz J, Sole A. Visceral leishmaniasis in lung transplantation. Transplant Proc 2003; 35(5): 2001-3.
440. Clemente W, Vidal E, Girao E, et al. Risk factors, clinical features and outcomes of visceral leishmaniasis in solid-organ transplant recipients: a retrospective multicenter case-control study. Clinical microbiology and infection : the official publication of the European Society of Clinical Microbiology and Infectious Diseases 2015; 21(1): 89-95.
441. Pittalis S, Nicastri E, Spinazzola F, et al. Leishmania infantum leishmaniasis in corticosteroid–treated patients. BMC Infectious Diseases 2006; 6: 177.
442. Fletcher K, Issa R, Lockwood DN. Visceral leishmaniasis and immunocompromise as a risk factor for the development of visceral leishmaniasis: a changing pattern at the hospital for tropical diseases, london. PLoS ONE 2015; 10(4): e0121418.
443. Fernandez-Guerrero ML, Aguado JM, Buzon L, et al. Visceral leishmaniasis in immunocompromised hosts. The American journal of medicine 1987; 83(6): 1098-102.
444. Zanger P, Kotter I, Kremsner PG, Gabrysch S. Tumor necrosis factor alpha antagonist drugs and leishmaniasis in Europe. Clinical microbiology and infection : the official publication of the European Society of Clinical Microbiology and Infectious Diseases 2012; 18(7): 670-6.
445. Herrador Z, Gherasim A, Jimenez BC, Granados M, San Martin JV, Aparicio P. Epidemiological changes in leishmaniasis in Spain according to hospitalization-based records, 1997-2011: raising awareness towards leishmaniasis in non-HIV patients. PLoS Neglected Tropical Diseases 2015; 9(3): e0003594.
446. Giavedoni P, Iranzo P, Fuertes I, Estrach T, Alsina Gibert M. Cutaneous leishmaniasis: 20 years' experience in a Spanish tertiary care hospital. Actas Dermosifiliogr 2015; 106(4): 310-6.
447. Kopterides P, Mourtzoukou EG, Skopelitis E, Tsavaris N, Falagas ME. Aspects of the association between leishmaniasis and malignant disorders. Transactions of the Royal Society of Tropical Medicine and Hygiene 2007; 101(12): 1181-9.
448. Fernandez-Guerrero ML, Robles P, Rivas P, Mojer F, Muniz G, de Gorgolas M. Visceral leishmaniasis in immunocompromised patients with and without AIDS: a comparison of clinical features and prognosis. Acta tropica 2004; 90(1): 11-6.
449. Schwartz BS, Mawhorter SD. Parasitic infections in solid organ transplantation. Am J Transplant 2013; 13 Suppl 4: 280-303.
450. AIDS I. Guidelines for Prevention and Treatment of Opportunistic Infections in HIV-Infected Adults and Adolescents. 2013.
451. Morizot G, Jouffroy R, Faye A, et al. Antimony to Cure Visceral Leishmaniasis Unresponsive to Liposomal Amphotericin B. PLoS Neglected Tropical Diseases 2016; 10(1): e0004304.
452. Neumayr ALC, Morizot G, Visser LG, et al. Clinical aspects and management of cutaneous leishmaniasis in rheumatoid patients treated with TNF-(alpha) antagonists. Travel Medicine and Infectious Disease 2013; 11(6): 412-20.
453. Veroux M, Corona D, Giuffrida G, et al. Visceral leishmaniasis in the early post-transplant period after kidney transplantation: clinical features and therapeutic management. Transpl Infect Dis 2010; 12(5): 387-91.
454. Simon I, Wissing KM, Del Marmol V, et al. Recurrent leishmaniasis in kidney transplant recipients: report of 2 cases and systematic review of the literature. Transpl Infect Dis 2011; 13(4): 397-406.
455. Clemente WT, Rabello A, Faria LC, et al. High prevalence of asymptomatic Leishmania spp. infection among liver transplant recipients and donors from an endemic area of Brazil. Am J Transplant 2014; 14(1): 96-101.
456. Silva JSF, Galvao TF, Pereira MG, Silva MT. Treatment of American tegumentary leishmaniasis in special populations: A summary of evidence. Revista da Sociedade Brasileira de Medicina Tropical 2013; 46(6): 669-77.
457. Figueiro-Filho EA, Duarte G, El-Beitune P, Quintana SM, Maia TL. Visceral leishmaniasis (kala-azar) and pregnancy. Infect Dis Obstet Gynecol 2004; 12(1): 31-40.
458. Figueiro-Filho EA, El Beitune P, Queiroz GT, et al. Visceral leishmaniasis and pregnancy: analysis of cases reported in a central-western region of Brazil. Arch Gynecol Obstet 2008; 278(1): 13-6.
459. Mueller M, Balasegaram M, Koummuki Y, Ritmeijer K, Santana MR, Davidson R. A comparison of liposomal amphotericin B with sodium stibogluconate for the treatment of visceral leishmaniasis in pregnancy in Sudan. The Journal of antimicrobial chemotherapy 2006; 58(4): 811-5.
460. Adam GK, Abdulla MA, Ahmed AA, Adam I. Maternal and perinatal outcomes of visceral leishmaniasis (kala-azar) treated with sodium stibogluconate in eastern Sudan. Int J Gynaecol Obstet 2009; 107(3): 208-10.
461. Pagliano P, Carannante N, Rossi M, et al. Visceral leishmaniasis in pregnancy: a case series and a systematic review of the literature. The Journal of antimicrobial chemotherapy 2005; 55(2): 229-33.
462. Gradoni L, Gaeta GB, Pellizzer G, Maisto A, Scalone A. Mediterranean visceral leishmaniasis in pregnancy. Scandinavian journal of infectious diseases 1994; 26(5): 627-9.
463. Caldas AJ, Costa JM, Gama ME, Ramos EA, Barral A. Visceral leishmaniasis in pregnancy: a case report. Acta Tropica 2003; 88(1): 39-43.
464. Topno RK, Pandey K, Das VN, et al. Visceral leishmaniasis in pregnancy - the role of amphotericin B. Ann Trop Med Parasitol 2008; 102(3): 267-70.
465. Thakur CP, Sinha GP, Sharma V, Barat D. The treatment of kala-azar during pregnancy. The National medical journal of India 1993; 6(6): 263-5.
466. Paumgartten FJ, Chahoud I. Embryotoxicity of meglumine antimoniate in the rat. Reprod Toxicol 2001; 15(3): 327-31.
467. Lima MI, Arruda VO, Alves EV, de Azevedo AP, Monteiro SG, Pereira SR. Genotoxic effects of the antileishmanial drug Glucantime. Arch Toxicol 2010; 84(3): 227-32.
468. Sindermann H, Engel J. Development of miltefosine as an oral treatment for leishmaniasis. Transactions of the Royal Society of Tropical Medicine and Hygiene 2006; 100 Suppl 1: S17-20.
469. FDA. Miltefosine. Highlights of prescribing information. Revised March 2014. Accessed 18 August 2015 at: http://www.accessdata.fda.gov/drugsatfda_docs/label/2014/204684s000lbl.pdf. 2014.
470. Dorlo TPC, Balasegaram M, Lima MA, De Vries PJ, Beijnen JH, Huitema ADR. Translational pharmacokinetic modelling and simulation for the assessment of duration of contraceptive use after treatment with miltefosine. Journal of Antimicrobial Chemotherapy 2012; 67(8): 1996-2004.
471. Dorlo TP, Balasegaram M, Beijnen JH, de Vries PJ. Miltefosine: a review of its pharmacology and therapeutic efficacy in the treatment of leishmaniasis. The Journal of antimicrobial chemotherapy 2012; 67(11): 2576-97.
472. van Thiel PP, Leenstra T, Kager PA, et al. Miltefosine treatment of Leishmania major infection: an observational study involving Dutch military personnel returning from northern Afghanistan. Clinical infectious diseases : an official publication of the Infectious Diseases Society of America 2010; 50(1): 80-3.
473. Valesky EM, Thaci D, Meissner M, et al. Cutaneous leishmaniasis: clinical report of two cases and review of the recent literature. J Dtsch Dermatol Ges 2007; 5(9): 770-2.
474. Morgan DJ, Guimaraes LH, Machado PR, et al. Cutaneous leishmaniasis during pregnancy: exuberant lesions and potential fetal complications. Clinical infectious diseases : an official publication of the Infectious Diseases Society of America 2007; 45(4): 478-82.
475. Guimaraes LH, Machado PR, Lago EL, et al. Atypical manifestations of tegumentary leishmaniasis in a transmission area of Leishmania braziliensis in the state of Bahia, Brazil. Transactions of the Royal Society of Tropical Medicine and Hygiene 2009; 103(7): 712-5.
476. Conceicao-Silva F, Morgado FN, Pimentel MIF, et al. Two Women Presenting Worsening Cutaneous Ulcers during Pregnancy: Diagnosis, Immune Response, and Follow-up. PLoS Neglected Tropical Diseases 2013; 7(12).
477. Ilett KF, Kristensen JH. Drug use and breastfeeding. Expert Opin Drug Saf 2005; 4(4): 745-68.
478. Berman JD, Melby PC, Neva FA. Concentration of Pentostam in human breast milk. Transactions of the Royal Society of Tropical Medicine and Hygiene 1989; 83(6): 784-5.

479. Solomon M, Schwartz E, Pavlotsky F, Sakka N, Barzilai A, Greenberger S. Leishmania tropica in children: a retrospective study. Journal of the American Academy of Dermatology 2014; 71(2): 271-7.
480. Burden-Teh E, Wootton CI, Williams HC. Can a simple outpatient-based treatment be used to treat cutaneous leishmaniasis in young children? A Critically Appraised Topic. The British journal of dermatology 2015; 172(4): 861-6.
481. Esfandiarpour I, Farajzadeh S, Rahnama Z, Fathabadi EA, Heshmatkhah A. Adverse effects of intralesional meglumine antimoniate and its influence on clinical laboratory parameters in the treatment of cutaneous leishmaniasis. International journal of dermatology 2012; 51(10): 1221-5.
482. Singh UK, Prasad R, Jaiswal BP, Singh PK, Thakur CP. Amphotericin B therapy in children with visceral leishmaniasis: daily vs. alternate day, a randomized trial. J Trop Pediatr 2010; 56(5): 321-4.
483. Minodier P, Robert S, Noel G, Blanc P, Retornaz K, Garnier JM. [First-line liposomal amphotericin B for pediatric visceral leishmaniasis in southern France]. Arch Pediatr 2005; 12(7): 1102-8.
484. Palacios R, Osorio LE, Grajalew LF, Ochoa MT. Treatment failure in children in a randomized clinical trial with 10 and 20 days of meglumine antimonate for cutaneous leishmaniasis due to Leishmania viannia species. The American journal of tropical medicine and hygiene 2001; 64(3-4): 187-93.
485. Rubiano LC, Miranda MC, Muvdi Arenas S, et al. Noninferiority of miltefosine versus meglumine antimoniate for cutaneous leishmaniasis in children. The Journal of infectious diseases 2012; 205(4): 684-92.
486. Layegh P, Rahsepar S, Rahsepar AA. Systemic meglumine antimoniate in acute cutaneous leishmaniasis: children versus adults. The American journal of tropical medicine and hygiene 2011; 84(4): 539-42.
487. Cruz A, Rainey PM, Herwaldt BL, et al. Pharmacokinetics of antimony in children treated for leishmaniasis with meglumine antimoniate. The Journal of infectious diseases 2007; 195(4): 602-8.
488. FDA. Miltefosine (Impavido®) for the treatment of visceral, mucosal and cutaneous leishmaniasis. FDA briefing document for the Anti-infective Drugs Advisory Committee Meeting. Meeting date, 18 Oct 2013. Accessed 9 March 2016 at: http://www.fda.gov/downloads/advisorycommittees/committeesmeeting materials/drugs/anti-infectivedrugsadvisorycommittee/ucm 371074.pdf. 2013.
489. Machado PR, Ampuero J, Guimaraes LH, et al. Miltefosine in the treatment of cutaneous leishmaniasis caused by Leishmania braziliensis in Brazil: a randomized and controlled trial. PLoS Neglected Tropical Diseases 2010; 4(12): e912.
490. Chrusciak-Talhari A, Dietze R, Chrusciak Talhari C, et al. Randomized controlled clinical trial to access efficacy and safety of miltefosine in the treatment of cutaneous leishmaniasis Caused by Leishmania (Viannia) guyanensis in Manaus, Brazil. The American journal of tropical medicine and hygiene 2011; 84(2): 255-60.
491. Bhattacharya SK, Jha TK, Sundar S, et al. Efficacy and tolerability of miltefosine for childhood visceral leishmaniasis in India. Clinical infectious diseases : an official publication of the Infectious Diseases Society of America 2004; 38(2): 217-21.
492. Rahman M, Ahmed BN, Faiz MA, et al. Phase IV trial of miltefosine in adults and children for treatment of visceral leishmaniasis (kala-azar) in Bangladesh. The American journal of tropical medicine and hygiene 2011; 85(1): 66-9.
493. Ostyn B, Hasker E, Dorlo TP, et al. Failure of miltefosine treatment for visceral leishmaniasis in children and men in South-East Asia. PLoS ONE 2014; 9(6): e100220.
494. Dorlo TPC, Huitema ADR, Beijnen JH, De Vries PJ. Optimal dosing of miltefosine in children and adults with visceral leishmaniasis. Antimicrobial Agents and Chemotherapy 2012; 56(7): 3864-72.
495. Dorlo TP, Rijal S, Ostyn B, et al. Failure of miltefosine in visceral leishmaniasis is associated with low drug exposure. The Journal of infectious diseases 2014; 210(1): 146-53.
496. Vasconcellos EC, Pimentel MI, Schubach AO, et al. Intralesional meglumine antimoniate for treatment of cutaneous leishmaniasis patients with contraindication to systemic therapy from Rio de Janeiro (2000 to 2006). The American journal of tropical medicine and hygiene 2012; 87(2): 257-60.
497. Herwaldt BL, Berman JD. Recommendations for treating leishmaniasis with sodium stibogluconate (Pentostam) and review of pertinent clinical studies. The American journal of tropical medicine and hygiene 1992; 46(3): 296-306.
498. Chappuis F, Alirol E, Worku DT, Mueller Y, Ritmeijer K. High mortality among older patients treated with pentavalent antimonials for visceral leishmaniasis in East Africa and rationale for switch to liposomal amphotericin B. Antimicrobial Agents and Chemotherapy 2011; 55(1): 455-6.
499. Diniz DS, Costa AS, Escalda PM. The effect of age on the frequency of adverse reactions caused by antimony in the treatment of American tegumentary leishmaniasis in Governador Valadares, State of Minas Gerais, Brazil. Revista da Sociedade Brasileira de Medicina Tropical 2012; 45(5): 597-600.
500. Oliveira LF, Schubach AO, Martins MM, et al. Systematic review of the adverse effects of cutaneous leishmaniasis treatment in the New World. Acta Tropica 2011; 118(2): 87-96.
501. Lawn SD, Armstrong M, Chilton D, Whitty CJ. Electrocardiographic and biochemical adverse effects of sodium stibogluconate during treatment of cutaneous and mucosal leishmaniasis among returned travellers. Transactions of the Royal Society of Tropical Medicine and Hygiene 2006; 100(3): 264-9.
502. Wise ES, Armstrong MS, Watson J, Lockwood DN. Monitoring toxicity associated with parenteral sodium stibogluconate in the day-case management of returned travellers with New World cutaneous leishmaniasis [corrected]. PLoS neglected tropical diseases 2012; 6(6): e1688.
503. Chulay JD, Spencer HC, Mugambi M. Electrocardiographic changes during treatment of leishmaniasis with pentavalent antimony (sodium stibogluconate). The American journal of tropical medicine and hygiene 1985; 34(4): 702-9.
